# Individualized funding interventions to improve health and social care outcomes for people with a disability: A mixed‐methods systematic review

**DOI:** 10.4073/csr.2019.3

**Published:** 2019-07-19

**Authors:** Pádraic Fleming, Sinead McGilloway, Marian Hernon, Mairead Furlong, Siobhain O'Doherty, Fiona Keogh, Tim Stainton

**Affiliations:** ^1^ Department of Psychology, Centre for Mental Health and Community Research, John Hume Building National University of Ireland Maynooth Maynooth Co. Kildare Ireland; ^2^ Health Sciences Centre, School of Public Health, Physiotherapy and Population Science University College Dublin, Belfield Dublin Ireland; ^3^ Centre for Economic and Social Research in Dementia National University of Ireland Galway Galway Ireland; ^4^ UBC School of Social Work & Centre for Inclusion and Citizenship Vancouver BC Canada

## 1 PLAIN LANGUAGE SUMMARY

### 1.1 Individualized funding has positive effects on health and social care outcomes

Individualized funding provides personal budgets for people with disabilities, to increase independence and quality of life. The approach has consistently positive effects on overall satisfaction, with some evidence also of improvements in quality of life and sense of security. There may also be fewer adverse effects. Despite implementation challenges, recipients generally prefer this intervention to traditional supports.

### 1.2 What is this review about?

Individualized funding is an umbrella term for disability supports funded on an individual basis. It aims to facilitate self‐direction, empowerment, independence and self‐determination. This review examines the effects and experiences of individualized funding.

**What is the aim of this review?**

This Campbell systematic review examines the effects of individualized funding on a range of health and social care outcomes. It also presents evidence on the experiences of people with a disability, their paid and unpaid supports and implementation successes and challenges from the perspective of both funding and support organizations.

### 1.3 What are the main findings of this review?

#### 1.3.1 What studies are included?

This study is a review of 73 studies of individualized funding for people with disabilities. These include four quantitative studies, 66 qualitative and three based on a mixed‐methods design. The data refer to a 24‐year period from 1992 to 2016, with data for 14,000 people. Studies were carried out in Europe, the US, Canada and Australia.

Overall, the evidence suggests positive effects of individualized funding with respect to quality of life, client satisfaction and safety. There may also be fewer adverse effects. There is less evidence of impact for physical functioning, unmet need and cost effectiveness. The review finds no differences between approaches for the Adult Social Care Outcomes Toolkit (ASCOT), self‐perceived health and community participation.

Recipients particularly value: flexibility, improved self‐image and self‐belief; more value for money; community integration; freedom to choose ‘who supports you; ‘social opportunities’; and needs‐led support. Many people chose individualized funding due to previous negative experiences of traditional, segregated, group‐orientated supports.

Successful implementation is supported by strong, trusting and collaborative relationships in their support network with both paid and unpaid individuals. This facilitates processes such as information sourcing, staff recruitment, network building and support with administrative and management tasks. These relationships are strengthened by financial recognition for family and friends, appropriate rates of pay, a shift in power from agencies to the individual or avoidance of paternalistic behaviour.

Challenges include long delays in accessing and receiving funds, which are compounded by overly complex and bureaucratic processes. There can be a general lack of clarity (e.g., allowable budget use) and inconsistent approaches to delivery as well as unmet information needs. Hidden costs or administrative charges can be a source of considerable concern and stress.

Staff mention involvement of local support organizations, availability of a support network for the person with a disability and timely relevant training as factors supporting implementation. Staff also highlight logistical challenges in support needs in an individualized way including, for example, responding to individual expectations and socio‐demographic differences.

### 1.4 What do the findings of this review mean?

This review provides an up‐to‐date and in‐depth synthesis of the available evidence over 25 years. It shows that there are benefits of the individualized funding model. This finding suggests that practitioners and funders should consider moving away from skepticism, towards opportunity and enthusiasm. Policy makers need to be aware of the set‐up and transitionary costs involved. Investment in education and training will facilitate deeper understanding of individualized funding and the mechanisms for successful implementation.

Future studies should incorporate longer follow‐ups at multiple points over a longer period. The authors of the review encourage mixed‐methods approaches in further systematic reviews in the field of health and social care, to provide a more holistic assessment of the effectiveness and impact of complex ‘real‐world’ interventions.

### 1.5 How up‐to‐date is this review?

The review authors searched for studies up to the end of 2016. This Campbell systematic review was published in January 2019.

## 2 EXECUTIVE SUMMARY/ABSTRACT

### 2.1 Background

The World Health Organisation estimates that 15% of the world's population live with a disability and that this number will continue to grow into the future, but with the attendant challenge of increasing unmet need due to poor access to health and social care (WHO, 2013). Historically, the types of supports available to people with a disability were based on medical needs only. More recently, however, the importance of social care needs, such as keeping active and socializing, has been recognized (Malley et al., 2012). There is now an international policy imperative for people with a disability to live autonomous, self‐determined lives whereby they are empowered and as independent as possible, choosing their supports and self‐directing their lives (Perreault & Vallerand, 2007; Saebu, Sørensen, & Halvari, 2013).

One way to achieve self‐determination is by means of a personal budget (United Nations, 2006). Personal budgets are just one example of many terms used to describe individualized funding – a mechanism to provide personalized and self‐directed supports for people with a disability, which places them at the center of decision‐making around how and when they are supported (Carr, 2010). Individualized funding –which is rooted in the Independent Living Movement (Glasby & Littlechild, 2009)– has evolved to take many forms. These include, for example, direct‐payments, whereby funds are given directly to the person with a disability who then self‐manages this money to meet their individual needs, capabilities, life circumstances and aspirations (Áiseanna Tacaíochta, 2014a). Alternatively, a microboard, brokerage model, or ‘managed’ personal budget provide a similar amount of freedom for the person with a disability, but an intermediary service assumes responsibility for administrative tasks, while sometimes also providing support, guidance and information to enable the person to successfully plan, arrange and manage their supports or care plans (Carr, 2010). Other types of models also exist, largely guided by country‐specific contexts, such as social benefits systems.

#### 2.1.1 The intervention

For the purposes of this review, the intervention included any form of individualized funding regardless of the name given, provided it met the following criteria: (a) it must be provided by the state as financial support for people with a lifelong physical, sensory, intellectual, developmental disability or mental health problem; (b) the recipient must be able to freely choose how this money is spent in order to meet their individual needs; (c) the individual can avail of ‘intermediary’ services or any equivalent service which supports them in terms of planning and managing how the money is used over the lifetime of the funding period; (d) the recipient can also independently manage the individualized fund, in whatever way is feasible; and (e) the individualized fund may be provided as a ‘once‐off’ pilot intervention for a defined period of time (minimum 6 months), or it can be a permanent move from more traditional forms of funding arrangements that exist nationally or regionally.

Commentators have indicated that strategic and policy decisions appear to be evolving on the basis of locally sourced or anecdotal evidence, due mainly to a lack of high quality experimental studies in the area (Harkes, Brown, & Horsburgh, 2014; Webber, Treacy, Carr, Clark, & Parker, 2014). While previous literature reviews exist (Carter Anand et al., 2012; Webber et al., 2014), we are not aware of any systematic review that focuses on the effectiveness of individualized funding in relation to people with a disability of any kind. Given the new policy imperative around individualized funding and the growing pool of studies in this area, there is now a need for a systematic review of these models across a spectrum of disabilities, in order to assess their effectiveness in relation to health and social care outcomes.

### 2.2 Objectives

The objectives of this review are to: (a) examine the effectiveness of individualized funding interventions for adults with a lifelong disability (physical, sensory, intellectual, developmental or mental disorder), in terms of improvements in their health and social care outcomes when compared to a control group in receipt of funding from more traditional sources; and (b) to critically appraise and synthesise the qualitative evidence relating to stakeholder perspectives and experiences of individualized funding, with a particular focus on the stage of ‘initial implementation’ as described by Fixsen, Naoom, Blase, Friedman, and Wallace (2005).

### 2.3 Search methods

In line with the study protocol (Fleming, Furlong, et al., 2016), ten academic databases and nine other grey literature databases/search engines were utilized. The terms used to customize the search string for specific databases were based on the ‘population’ and ‘intervention’ of interest. ‘Disability’ and all possible variations including mental health, disorders and autism was the first keyword. ‘Budget’ and all variations of same was the second keyword. Database specific conventions were followed to ‘explode’ or ‘truncate’ key terms as appropriate. A list of free‐text terms which were identified in the literature supplemented the syntax developed. Study design and outcomes were not included as part of the search strategy as it was anticipated that this would potentially lead to the omission of relevant literature. Bibliographies from included and some excluded studies (e.g., literature reviews) were used to guide forward citation searching. Conference proceedings, manual browsing of key journals and other online materials guided hand‐searching.

### 2.4 Selection criteria

The population of interest included: adults aged 18 years and over receiving a personal budget, with any form or level of lifelong disability (physical, sensory, intellectual or developmental disability, level of mental health problem, disorder or illness, or dementia), residing in any country and any type of residential setting (own home, group home, residential care setting, nursing home, hospital, institution). Studies in any language were included.

Minors and older people without a lifelong disability (i.e., no disability in 10 years prior to reaching the age of 65) were excluded, as were privately funded individualized funding interventions.

### 2.5 Data collection and analysis

Due to the very large search results (*n* = 82,274 after duplicates and non‐relevant grey literature excluded), an extensive, thorough and transparent ‘results refinement process’ was developed in order to filter these results. Following this refinement process, a screening of studies, based on the inclusion/exclusion criteria, was undertaken in two stages. The first stage involved title and abstract screening; the second involved full text documents. Three independent researchers were involved at each stage. Risk of bias and quality of research was evaluated using a range of tools (depending on study design) by one reviewer (PF). Further quality screening took place, during full text screening, by two second reviewers (MH & SOD).

A very high level of irregularity was observed across studies making them unamenable to metasynthesis, mainly based on the use of inconsistent, unstandardized, and often invalidated outcome measures as well as the selection of control groups. With regard to the latter, some control group participants were randomly assigned, some did not wish to leave traditional services, whilst others were on a waiting list to avail of individualized funding. Furthermore, the study designs were heavily influenced by country‐specific, and changing economic and policy landscapes. Therefore, a narrative analysis of quantitative data was considered the approach which would best represent the results. Narrative systematic reviews serve several functions including reporting the effects of interventions and also the factors impacting their implementation (Popay et al., 2006). A meta‐synthesis of qualitative data was undertaken to build upon the latter point, based on the experiences of intervention participants, in addition to outlining the key facilitators and challenges associated with implementation, from the perspective of multiple stakeholders. Key themes were identified, which were conceptually folded together across studies.

### 2.6 Results

Of the 82,274 potentially relevant titles originally identified, 7,158 were independently double screened based on ‘title / abstract’ and a subsequent 328 full‐text articles were doubled screened. In total, 73 studies met the inclusion criteria and were included in the review, 66 (90%) of which were qualitative in nature.

#### 2.6.1 Quantitative

Seven unique studies contained eligible quantitative data (including three mixed methods) and were included in the review, representing 19 titles in total. One of the studies was an unpublished report (available online), while the remaining six were reported in both unpublished reports and published peer‐reviewed journal articles. All studies were English language and the majority were based in the United States (*n* = 5). One study was a ‘quasi‐experimental controlled longitudinal survey’, three were ‘randomized, controlled cross‐sectional surveys’ and three were ‘randomized controlled before and after studies’. A total of 4,834 adults were represented in the narrative synthesis, with a collective response rate of 73%. The risk of bias was high or unclear for majority of studies, while the quality rating was fair to good. Five studies reported one or both primary outcomes of interest.

Two of the four studies which reported quality of life outcomes showed positive effects for those receiving individualised funding (two showed no difference):

- Site 1 (I: 43.4 / C: 22.9, MD = 20.5 (*p* < 0.001)); Site 2 (I: 63.5 / C: 50.2, MD = 13.3 (*p* < .01)); and Site 3 (I: 37.5 / C: 21.0, MD = 16.5 (*p* < .001)) (Brown et al., 2007);
- (I: M = 10.12, *SD* = 6.93 / C: M = 13.28, *SD* = 7.37, MD =−3.16, (*p* < .001) (95% CI: −4.65, −1.67)) (Woolham & Benton, 2013).

All five studies reporting client satisfaction showed positive effects for those receiving the intervention:

- (I: 61.4,: 9.7 / C: 52.1, *SD* = 10.9, MD = 9.3, (*p* < .001), (CI 95%: 4.80–13.80)) (Beatty, Richmond, Tepper, & DeJong, 1998);
- satisfaction with:
  ○ technical quality ‐ (I: 20.90, *SD* = 3.31 / C: 20.07, *SD* = 3.82, MD = 0.83, (*p* < .001), (CI 95%: 0.41–1.25);
  ○ service impact ‐ (I: 8.09, *SD* = 1.98 / C: 7.63, *SD* = 1.96, MD = 0.46, (*p* < .001), (CI 95%: 0.23– 0.69));
  ○ general satisfaction (I: 9.06, *SD* = 1.65 / C: 8.66, *SD* = 2.07, MD = 0.40, (*p* < .001), (CI 95%:0.18– 0.62)); and
  ○ interpersonal manner (I: 7.45, *SD* = 1.80 / C: 6.43, *SD* = 1.92, MD = 1.02, (*p* <  0.001), (CI 95%: 0.80–1.24)) (Benjamin, Matthias, & Franke, 2000);
- satisfaction with:
  ○ caregiver help
    ▪ Site 1 (I: 90.4 / C: 64.0, MD = 26.4, (*p* < .001));
    ▪ Site 2 (I: 85.4 / C: 70.9, MD = 14.5, (*p* < .01)); and
    ▪ Site 3 (I: 84.4 / C: 66.0, MD = 18.4, (*p* < .001));
  ○ and overall care arrangements
    ▪ Site 1 (I: 71.0 / C: 41.9, MD = 29.2, (*p* < .001));
    ▪ Site 2 (I: 68.2 / C: 48.0, MD = 20.2, (*p* < .01)); and
    ▪ Site 3 (I: 51.9 / C: 35.0, MD = 16.9, (*p* < .001))(Brown et al., 2007);
- (I: M = 3.89, *SD* = 0.85 / C: M = 2.82, *SD* = 1.25, MD = 1.07, (CI 95%: 0.63 – 1.51) (*p* < .001)) (Caldwell, Heller, & Taylor, 2007);
- and (I: *n* = 478, C: *n* = 431, proportion satisfied I: 0.78, C: 0.70, x^2^ = 7.54, (*p* < 0.01)) (Glendinning et al., 2008).

Secondary outcomes included physical functioning, costs and adverse effects. Only one study reported physical functioning, with no difference detected between intervention and control groups.

Two studies reported cost effectiveness data. One showed no difference between groups, while the other suggested that individualized funding was less cost‐effective than traditional supports (in one of two measures). Personal Care/HCBS alone − (Arkansas I: M = 5,435 / C: M = 2,430, MD = 3,005, (*p* < .001), Florida I: M = 22,017 / C: M = 18,321, MD = 3,696, (*p* < .001), New Jersey I: M = 11,166, C: M = 9,220, MD = 1,946, (*p* < 0.001)) (Brown et al., 2007, Table V.1; Dale & Brown, 2005).

Five studies reported adverse effects with two reporting no difference between intervention and control. One study reported two measures of ‘unmet need’, with one favoring the control group (I: M = 5.07, *SD* = 1.54, C: M = 5.38, *SD* = 1.21, MD = −0.31, *p* < .001, (CI 95%: −0.48 to −0.14;Benjamin et al., 2000), the second showing no difference. For the remaining two studies, those receiving individualized funding reported fewer:

- adverse effects: (I: M = 3.11, *SD* = 3.30 / C: M = 7, *SD* = 5.31, MD = −3.89, (*p* < .001), (CI 95%: −5.71 to −2.07)) (Caldwell et al., 2007); and
- unmet needs with daily living activities –
  ○ Site 1 (I: 25.8 / C: 41.0, MD = −15.2, (*p* < .01));
  ○ Site 2 (I: 26.7 / C: 33.8, MD = −7.1, (*p* < .05)); and
  ○ Site 3 (I: 46.1 / C: 54.5, MD = −8.4, (*p* < .05)) (Brown et al., 2007).

The remaining five measures of unmet need, in the last study, varied between study sites – some reporting no difference, whilst others favoured the intervention group.

Other relevant health and social care outcomes were also reported in three of the four quantitative studies. Safety/sense of security was the only outcome on which a significant difference was reported and in favor of the intervention group (I: M = 9.18, *SD* = 1.57, C: 8.96, *SD* = 1.65, MD = 0.22, *p* < .05 (CI 95%: 0.03– 0.41)) (Benjamin et al., 2000).

#### 2.6.2 Qualitative

##### Implementation facilitators

1. People with a disability and their carers/representatives consistently report many perceived benefits of individualized funding. This strongly suggests that implementation is well received and often advocated for, among people with a disability. Benefits that are particularly valued include: flexibility, improved self‐image and self‐belief; more value for money; community integration; freedom to choose ‘who supports you; ‘social opportunities’; and needs‐led support.
2. There are many mechanisms of success discussed, including the importance of strong, trusting and collaborative relationships. These extend to both paid and unpaid individuals, often forming the person's network of support which, in turn, plays an integral role in facilitating processes such as information sourcing, staff recruitment, network building, and support with administrative and management tasks. Factors that strengthen these relationships include: financial recognition for family and friends, appropriate rates of pay, a shift in power from agencies to the individual or avoidance of paternalistic behaviour.
3. Implementation facilitators from the perspective of staff, include the involvement of local support organizations, and the availability of a network of support for the person with a disability. Timely relevant training for practitioners, coordinators, and other frontline staff is also seen as an important facilitator, as are sufficient support and other human resources available to people with a disability, such as intermediary services, community integration, and innovative/creative supporters.

##### Implementation challenges

1. Perceived challenges for participants include agency involvement and lack of trusting working‐relationships due to previous negative experiences. Participants often experience long delays in accessing and receiving funds, which are compounded by overly complex, rigid, and bureaucratic assessment, administrative and review processes. A general lack of clarity (e.g., allowable budget use) and inconsistent approaches to delivery as well as unmet information needs are other major concerns, as are difficulties with finding and retaining suitable staff. Various internal factors (e.g., managing personal issues and negative emotions) and external factors (e.g., weak network of support) are mentioned as additional challenges to the process of implementation.
2. A number of barriers, whilst viewed as generally manageable in the short term, were considered potentially problematic in the longer term. These include: inaccurate or inaccessible information sometimes due to an unclear understanding of individualized funding (compounded by an absence of practitioner training); cumbersome systems that duplicate work and are framed within the directive medical model (i.e., based on a perception that staff inappropriately focus on targets and costs rather than quality of support provided); and a lack of resources/available support, exacerbated by an inaccurate estimation of need and subsequent delay in reviewing /adjusting budgets. This, amongst other things, can lead to conflict and tensions in working relationships, which are also hampered by disabling practices (e.g., exclusion from decision‐making). Lastly, financial hardship is commonly cited, with hidden costs or administrative charges widely identified as a source of considerable concern and stress for participants.
3. Other challenges to implementation, from the perspective of, or related to, staff/organizations include: risk aversion rooted in fears associated with perceived vulnerability of people with a disability and potential for abuse or exploitation; fear of misuse or fraud (by people with a disability); and concerns related to the long‐term sustainability of individualized funding, the quality of available supports and the impact on the traditional service providers/workforce. Staff also highlight logistical challenges in accommodating a wide range of support needs in an individualized way including, for example, responding to individual expectations and socio‐demographic differences.

### 2.7 Authors’ conclusions

Due to the considerable and growing interest in individualized funding as a means to improve the lived experience of people with a disability and their wider network of support (paid and unpaid), this review provides a comprehensive synthesis of evidence for future governments, funders, and policy makers. Commentators have previously criticized governments for proceeding with individualized funding initiatives without carefully considering the evidence. This review, therefore, provides an up‐to‐date repository of such evidence, particularly for countries at the early stages of planning or implementation. Not only does it present the most robust effectiveness data available, but it also specifically highlights implementation successes and challenges.

The evidence suggests that practitioners and funders need to shift their focus from one of skepticism, often grounded in fears, to one of opportunity and enthusiasm. Many of the fears, such as fraud/misuse of funds, job losses, recipients flooding the system, are not based on evidence. Funders and practitioners should be guided by the many examples of good practice outlined in this review, whilst working collaboratively toward, and appreciating the consistently reported benefits of, individualized funding. Greater investment is needed in education and training in order to facilitate stakeholder buy‐in and generate a better understanding of individualized funding and the philosophy and ethos and the associated mechanisms required for its successful implementation. Finally, policy makers need to be cognizant of the inevitable set‐up and transitionary costs involved such as capital funding for education and training, as well as redevelopment of assessment, review and other governance systems. In order to facilitate this spending, policy need to be put in place to allow the release of funds from block grants, if implementation is to be cost‐effective in the longer term.

This review clearly highlights and synthesizes the extensive and rich qualitative evidence from studies conducted in many countries – across changing social, political, economic, social care and healthcare landscapes – and over a considerable period of time. It also points to the inherent difficulties associated with collecting quantitative data on complex social interventions of this nature, with a subsequent lack of robust effectiveness data. The complexities around set‐up and attendant delays, highlighted in the qualitative data, suggest necessary changes in any future collection of quantitative outcomes. For example, future researchers should consider (resources permitting) conducting studies which incorporate longer follow‐ups (minimum 9 months), and ideally at multiple time‐points over a longer period of time. Finally, the authors of this review would encourage the adoption of mixed‐methods approaches in further systematic reviews when assessing the effectiveness of complex ‘real‐world’ interventions in the field of health and social care.

## 3 BACKGROUND

### 3.1 The problem

More than a billion people – or about 15% of the world's population – are estimated to live with some form of disability, and these rates are increasing over time (WHO, 2013). The International Classification of Functioning, Disability and Health, defines disability as an umbrella term for impairments, activity limitations, and participation restrictions. According to the WHO, disability is the interaction between individuals with a health condition (e.g., cerebral palsy, Down syndrome, and depression) and personal and environmental factors (e.g., negative attitudes, inaccessible transportation and public buildings, and limited social supports; WHO, 2013). The WHO (2013) recognizes that disability is extremely diverse, but that generally, rates of disability are increasing due to population ageing and a greater prevalence of more chronic health conditions, whilst people with disabilities also have less access to health care services and, therefore, more unmet needs than ever before. There is further evidence to suggest that people with disabilities have lower life expectancies (Patja, Iivanainen, Vesala, Oksanen, & Ruoppila, 2000).

The many different needs of people with a disability, learning difficulty or mental health problems tend to be met through a range of activities, which may be described, collectively, as ‘social care’. These include help with personal hygiene, dressing and feeding, or general life skills such as shopping, keeping active, and socializing (Malley et al., 2012). In recent years, the disability and mental health sectors have witnessed a significant shift towards community‐based health and social care services that attempt to place the service user at the centre of decision‐making and service delivery. A growing body of policy now describes how people with all disabilities should be autonomous and self‐determined members of society.

The concept of self‐determination has its roots in self‐determination theory, which is based on human motivation, development and wellness. According to Deci and Ryan (2008), the theory focuses on the type and quality of motivation as a predictor of performance and well‐being outcomes, as well as social conditions that are improved by such motivations. Autonomous motivation, in particular (compared to controlled motivation) – whereby intrinsic and extrinsic motivation allows individuals to identify with an activity's value and integrate it into their sense of self – can lead to better psychological health, performance and a shift toward healthier behaviors. While controlled motivation – when compared to a motivation – ‘can lead to improvements, these are limited because individuals feel a pressure to think, feel and behave in certain ways (in order to avoid shame or to gain approval from the external regulation), when functioning under a system of reward or punishment. Self‐determination theory also examines the impact of self‐determination on life goals and aspirations and can be applied to a wide range of domains, including relationships, work, education and health care (Deci & Ryan, 2008). The findings of a recent meta‐analysis of 184 studies – based on self‐determination theory in health care and health promotion contexts – showed positive relationships between the satisfaction of psychological needs, autonomous motivation and positive health outcomes (Ng. et al., 2012). A number of more specific studies that have examined self‐determination in a sample of people with a disability found similarly positive outcomes (Perreault & Vallerand, 2007; Saebu et al., 2013).

One way to achieve self‐determination is by means of a personal budget (United Nations, 2006). Individualized funding is rooted in the Independent Living Movement and the associated Independent Living Fund, whereby people with a disability self‐directed their support by hiring a ‘personal assistant’ (PA) to gain more control over their lives and services. While the concept of independent living varies internationally, all approaches emphasize choice and control whilst acknowledging that personal budgets are just one way to achieve their goals (Glasby & Littlechild, 2009). A personal budget, also known as ‘individualized funding’, is an umbrella term for various funding mechanisms that aim to provide personalized and individualized support services for people with a disability. Whilst the terminology may vary, the principles are similar and are based on self‐determination, choice and, very often, person centred planning. Thus, individualized funding aims to place the service user at the centre of the decision making process, thereby recognizing their strengths, preferences and aspirations and empowering them to shape public services, social care and support by allowing the service user to identify their needs, and to make choices about how and when they are supported (Carr, 2010). As a result, many international governments are recommending individualized funding as a means to empower individual service users or their advocates, whilst ensuring transparency in the allocation and use of resources.

For example, in Ireland, there are several key policy goals (e.g., enshrined in the Value for Money and Policy Review of Disability Services (Department of Health, 2012)) which promote the use of ‘individual needs assessments’. These assessments can lead to a personal budget which can then be used to purchase services from within existing (limited) resources (Keogh, 2011). In the UK, personal budgets are common and are facilitated by standardized resource allocation systems that include a robust needs assessment. Furthermore, a social care outcomes framework is in place to monitor how well social care services are delivering the most meaningful outcomes for people with disabilities whilst also addressing any shortcomings therein (Department of Health, 2013). The monitoring process is supported by tools such as the ASCOT which was used, for example, in an evaluation of personal budgets commissioned by the UK Department of Health (Forder et al., 2012). This tool comprises eight conceptually distinct attributes or domains including: personal cleanliness and comfort; food and drink; control over daily life; personal safety; accommodation cleanliness and comfort; social participation and involvement; occupation; and dignity (Malley et al., 2012).

There are several types of personal budget which can be used to address these kinds of health and social care needs; the two most common involve either a direct payment model or an intermediary service.

A direct payment involves the funds being given directly to the person with a disability, who then self‐manages this money to meet their individual needs, capabilities, life circumstances and aspirations (Áiseanna Tacaíochta, 2014a). This may include the employment of a personal assistant to help with everyday tasks and/or the purchase of services from private, voluntary or community service provider organizations (Carter Anand et al., 2012). Direct payments often involve considerable administrative duties for the person with a disability and are more likely, therefore, to be utilized by people with a physical or sensory disability and less so by those with an intellectual or developmental disability. However, in some cases, a person with a mild intellectual disability may have the skills to manage the direct payment, with or without the support of family members or other natural supports (or informal care). More severe intellectual disabilities would most likely require some kind of family/natural support – this having been the driving force behind microboards in Canada, for example. A micro board is a small non‐profit group of informal supports (family and friends) who assist persons with disabilities to develop individualized housing and support options (Malette, 1996). This review endeavors to determine whether the benefits of direct payments are affected by the type and degree of disability, or indeed the involvement of third parties whether paid or unpaid.

A microboard, brokerage model, or ‘managed’ personal budget, whilst it provides a similar amount of freedom (as a direct payment) for the person with a disability around choice and control of services utilized, it involves a third‐party assuming responsibility for administrative tasks and providing support, guidance and information to enable the person to successfully plan, arrange and manage their support services or care plans (Carr, 2010). A ‘managed’ personal budget tends to focus more on administration and financial management, with the budget held centrally by an organisation. This service is often referred to as a fiscal intermediary (Carter Anand et al., 2012). The tasks of a broker, on the other hand, include working with the person with a disability to develop an individual action plan, as well as researching options within the community to fulfil the goals in the action plan. The broker can also assist in negotiating costs with service providers and are available for support of the individual when necessary (PossibilitiesPlus, 2014). Brokerage models tend to have a far reaching impact across service provision and local authority purchasing by encouraging more flexible and innovative solutions for user‐orientated services, whilst also influencing the development of payment schemes (Zarb, 1995).

Whilst the involvement of brokers is ongoing, their presence in the life of the individual tends to be more intensive in the initial transition (i.e., from traditional services) and set‐up stages. During this period, the broker will help to develop the ‘circle of support’, either from scratch when none currently exists, or by expanding an existing support structure to include extended family members, such as aunts, uncles, cousins, friends and members of the wider community. During this initial period, the broker may also assist in the recruitment of staff for day‐to‐day support. For this reason, this review seeks to determine whether or not these intervention effects differ based on the level and quality of support available, both paid and unpaid. Some research suggests that the circle of support is integral to the successful implementation of such an intervention (Curryer, Stancliffe, & Dew, 2015; Fleming, McGilloway, & Barry, 2015c). Furthermore, the quality of paid support may also affect outcomes since the provision of broker/facilitator training has been found to be a successful element of individualized models of support (Fleming et al., 2015c; Lord & DeVidi, 2015).

A third type of model, the Cash and Counselling model, is found predominantly in the US and allows the user the flexibility to choose between a self‐managed and a professionally managed/assisted account. This represents a combination of the direct payment and intermediary models described above (NRCPDS, 2014). In many jurisdictions, the brokerage/support function which facilitates planning and implementation, is separated from the ‘fiscal management’ supports which handle the accounting and human resource issues, but not the personal planning/support/monitoring element. While these can be conflated in some cases, it is generally considered important to maintain the independence of the brokerage/planning function from the fiscal dimension to avoid conflict of interest. The separation of the two allows individuals or advocates who do not wish to have any planning support to secure the ‘payroll’ services required without any obligation to avail of planning and monitoring supports.

While ‘individualized funding’ is emerging as an umbrella term for the various funding mechanisms, the terminology remains unclear. A decade ago, ‘cash‐for‐care’ or ‘cash and care’ were predominant umbrella terms when reviewing evidence over several decades from the US, UK and EU (Glendinning & Kemp, 2006; Ungerson & Yeandle, 2007). These early studies highlighted the risks associated with the marketization and indirect privatization of care services whereby ‘consumers of care’ increasingly act as employers without necessarily having the human resource skills or knowledge of available care choices (Woods, 2008). In contrast, evidence suggests that people availing of individualized funding are capable of acquiring the necessary skills, or indeed able to outsource certain tasks in order to successfully bypass the service providers and contract their support services directly (Fleming et al., 2015c). Thus, there exists a tension between individuals with a disability, who can secure potential cost savings while having more autonomy, and traditional service providers who need to maintain contractual agreements with staff members within their organizations.

Further tensions may also exist for frontline staff between their ethical obligations to promote empowerment and self‐determination whilst honouring their legal obligations to limit access to individualized funding (Ellis, 2007). Another challenge for staff relates to risk management. A balancing act is required to facilitate positive risk‐taking whilst ensuring that the individualized funding‐specific risks, such as financial abuse, neglect or physical/emotional abuse, are avoided. This requires careful consideration and planning, but risk management can vary considerably. For example, during the piloting of personal budgets in the UK, local authorities conducted risk assessments but in some cases relied on annual reviews, thereby placing the onus of responsibility on individuals or families in the interim (Glendinning et al., 2008). Carr and Robbins (2009) also highlight the region‐specific contextual factors, such as culture and policy, which can influence implementation of individualized funding. For example, in certain jurisdictions in Canada, the US and the Netherlands, it is compulsory to use an independent support broker, whilst in the UK and US, ‘personal assistants’ are the preferred option for those receiving personal budgets. The eligibility criteria may also differ at initial implementation depending on the region. For example, in Canada, the focus was on younger people with learning disabilities whereas the Swedes focused on adults with physical disabilities; furthermore, very few regions accommodated people with mental health problems. Objectives also differed; for example, Australia initially focussed on tackling fragmented service provision, particularly in rural areas, while the US concentrated on solving staff shortages in long‐term care facilities (Carr & Robbins, 2009).

All of the above interventions, regardless of delivery mode, involve a transitionary period which can present challenges for individuals and families, particularly when national systems of allocating resources are not in place and families have to negotiate the release of funds from a regional disability manager, as is the case, for example, in Ireland (Fleming et al., 2015c). This period of transition can also be a time of great uncertainty for individuals and their families (where applicable) who have left a form of service provision to which they have been accustomed, often for many years. As a result, the length of time that the intervention has been in place may considerably affect its real or perceived effects. Furthermore, socio‐demographic factors may have a similar impact; for example, an older person may have been using traditional forms of services for much longer than a young adult transitioning from mainstream school or another form of secondary education. Thus, past experiences, such as institutionalization, may dramatically affect an older person's ability to adapt to this new model of service provision. Equally, more people living in rural areas have been found to avail of day services when compared to urban dwellers, potentially due to a lack of alternatives within the community (Fleming, McGilloway, & Barry, 2016a). This dependence on traditional day services may impact an individual's ability to adjust to the new model, or could limit the potential for community integration due to a lack of community services for the general population. Therefore, this review took such confounding factors into consideration, both in the inclusion/exclusion criteria and in the subgroup analysis.

### 3.2 The intervention

For the purposes of this review, the intervention included any form of personal budget, regardless of the name given to the model of delivery. As indicated above and outlined in Table 1, these models may be described in many different ways. For example, Webber et al. (2014) identified the following terms: ‘Individual Budgets’; ‘Recovery Budgets’; ‘Personal Budgets’; ‘Direct Payments’; ‘Direct Health Budgets’; and ‘Cash and Counselling’. Others include ‘third party managed’ personal budgets, direct payments managed by an appointed person and individual service funds. However, a personal budget, to be included in this review, must have the following fundamental characteristics: (a) it must be provided by the state as financial support for people with a lifelong physical, sensory, intellectual, developmental disability or mental health problem; (b) the recipient must be able to freely choose how this money is spent in order to meet their individual needs; (c) the individual can avail of ‘brokerage/intermediary’ services or any equivalent service which supports them in terms of planning and managing how the money is used over the lifetime of the funding period; (d) the recipient can also independently manage the personal budget, in whatever way is feasible, such as setting up a ‘Company Limited by Guarantee’ as is the case in Ireland (Áiseanna Tacaíochta, 2014b); and (e) the personal budget may be provided as a ‘once‐off’ pilot intervention for a defined period of time (minimum 6 months), or it can be a permanent move from more traditional forms of funding arrangements that exist nationally or regionally.

**Table 1:** Examples of terminology used globally

Country	Terms used		Source of money	Support / care mechanism
U.S.A	Self‐Determination programs		Medicaid waivers at State level	Independent consultant
	Cash and Counseling			Fiscal intermediary services
	Consumer Directed Care/Support			
U.K.	Direct Payments		Local Authority	Personal assistant
	Individual Budget		Local Authority	Package of care from multiple sources
	Block funding from the Social Care budget		Social Care budget	Residential costs and associated care costs
	Independent Living Fund		Department for Social Security	Care from agency OR personal assistant
Other terms used in UK	Recovery Budget			
	Personal Budget			
	Personal Health Budget			
	Microboard			
Other UK funding sources:	Supporting People fund			
	○ Access to work funding			
	○ Disabled Facilities Grants			
Netherlands	Person‐centred budget		Dutch Welfare State	Package of self‐determined care. Assisted by employed care worker (Often Informal (family) carers)
Ireland	Independent Support Broker/Brokerage		Innovation funding for pilot	Package of care from multiple sources/residential costs
			Ongoing funding from HSE	
	Direct payments		Innovation funding for pilot Ongoing funding from HSE	Package of care from multiple sources/residential costs
	Self‐management model		Innovation funding for pilot	Community Connector
Canada	Direct Payment/Direct Funding		Community Living British Columbia (CLBC)	Supports and services for the individual as agreed to by the individual, agent and CLBC facilitators and CLBC analysts
	Host Agency Funding		CLBC	
Other terms used in Canada	○ Self‐managed care			
	○ Individualised funding program			
	○ Support for Interdependent living			
Australia	○ Local Area Co‐ordination Program			Microboard
				Self‐directed funding
	○ Shared management model			
				Consumer‐directed care
	○ Self‐management (direct payments)			
	○ National Disability Insurance Scheme (NDIS)			
Other terms used internationally	Indicative allocation; Individual service fund; Managed account; Managed budget; Notional budget; Personalized care; Pooled budget; Self‐directed care; Self‐directed support; Virtual budget; Cash‐for‐care			

*Note*: International data sourced from: (Carter Anand et al., 2012; A. Power, 2010; Webber et al., 2014).

Individualized funding interventions are implemented with a view to delivering a range of positive health and social care outcomes over time. It is expected that a persons’ quality of life will improve (e.g., socially, personally, environmentally and in terms of their physical/psychological health) as a result of their increased autonomy, choice and control over daily life decisions and greater social integration and interaction. Client satisfaction is also expected to increase due to greater self‐determination, whilst the same is true for physical functioning which may improve due to better independent life skills (i.e., taking on more responsibilities such as shopping and household chores).

Many of these quality of life outcomes, if improved, could arguably generate cost benefits, although the evidence in this respect is very limited. The small pool of evidence would suggest that individualized funding can be cost effective, ranging from 7% to 16% in the US (Conroy, Fullerton, Brown, & Garrow, 2002) and 30% to 40% in the UK (Zarb & Nadash, 1994). Conversely, one UK study suggested that individualized funding may not result in cost savings, but does represent value for money (Glasby & Littlechild, 2002). Stainton, Boyce and Phillips (2009) support these more conservative findings showing relative cost neutrality for individualized funding when compared to independent service providers; however, individualized funding was more cost effective than traditional in‐house service provision. Furthermore the authors reported higher levels of user satisfaction for those availing of individualized funding, thereby highlighting the link between client satisfaction, quality of life and cost benefits.

### 3.3 Why it is important to do the review

The international move towards individualized funding has led, in turn, to a growing interest in identifying methods, more generally, that might offer the most potential in terms of informing effective and efficient resource allocation, particularly in the context of recent economic reforms. However, these strategic and policy decisions would appear to be evolving on the basis of locally sourced or anecdotal evidence, since there appears to be a lack of high quality experimental studies in the area (Webber et al., 2014). Nonetheless, current international evidence suggests many benefits of individualized funding, such as increased choice and control, a positive impact on quality of life (QoL), reduced service use and potential for cost effectiveness (Field, 2015; Webber et al., 2014). Thus, it is important to explore the pathways/mechanisms that lead to change (in this case positive change) and to determine the links between activities, outputs and outcomes (Taplin, Clark, Collins, & Colby, 2013).

In the case of individualized funding, it is intended that people with disabilities have more autonomy over their lives which, in turn, acts as a mechanism to enhance self‐determination, something that most people without a disability take for granted. A mantra that resonates globally within the disability sector is ‘Nothing about us, without us’ (Charlton, 1998). This aptly illustrates the fundamental need to place the person with a disability at the centre of decision making. Thus, individualized funding and attendant services are designed as a vehicle/mechanism for potentially improved health and social care outcomes. Such individualized funding arrangements are also important in shifting the power dynamic from service providers and placing it in the hands of individuals with a disability (or their families).

Glendinning et al. (2008) reported mixed findings in their RCT on the impact of a personal budget on health, social care and personal outcomes within their subgroup analyses. Outcomes varied according to age or mental health status, whilst the type of disability did not appear to play an important role (Glendinning et al., 2008). Furthermore, health outcomes may vary across various jurisdictions where different rules exist on what can or cannot be funded from a personal budget – particularly health services which may have different eligibility rules by region. Importantly, international evidence on individualized funding models suggests that there is no ‘one size fits all’ approach for everyone; hence, there is considerable variation with regard to: levels of choice and control given to service users; the professionals involved; the type of funder; and the limitations in both the services available for purchase and administrative structures/ processes (Carter Anand et al., 2012).

It is notable that the type of study design also varies considerably in the evaluation of individualized funding. Studies include, but are not limited to: RCTs (Glendinning et al., 2008; Shen et al., 2008); quasi‐experimental trials with controls (Forder et al., 2012; Foster, Brown, Phillips, & Schore, 2003; Teague & Boaz, 2003); and without controls (Spaulding‐Givens, 2011); cross‐sectional surveys (Hatton & Waters, 2011; Lawson, Pearman, & Waters, 2010); and qualitative studies (Coyle, 2009; Homer & Gilder, 2008; Maglajlic, Brandon, & Given, 2000).

#### 3.3.1 Prior reviews

We are aware of only two reviews, to date, which have specifically examined individualized funding for people with a disability or mental health problem. Both of these included quantitative and qualitative data. The first, by Carter Anand et al. (2012) (25 studies), was a rapid evidence assessment rather than a rigorous systematic review. As a result, the search strategy had some major limitations, such as the exclusion of non‐English studies and a geographical restriction to seven countries: the US, Australia, Germany, Great Britain, Ireland, The Netherlands and New Zealand. The authors acknowledged that the search strategy had resulted in a limited evidence base, which precluded the possibility of drawing strong conclusions about the implementation and impact of individualized funding. However, they also indicated that the qualitative evidence derived from service users tended to reflect positive views about the initiatives. The review did not report on the characteristics of included studies, or on study results in any detail. Furthermore, there was no detail about whether or not a meta‐analysis was conducted, or the methods by which the qualitative data were synthesized. In addition, no subgroup analyses were conducted despite an apparent broad definition of disability (e.g., various types and level of physical and intellectual disabilities, inclusion of older people and those with mental health problems). Finally, while quality was assessed, no information was provided on any assessment of bias.

The second more recent review by Webber et al. (2014) closely followed the EPPI‐Centre methodology for conducting a systematic review, appraising methodology and assessing the research quality and reliability (Gough, Oliver, & Thomas, 2012). Once again however, non‐English studies were excluded, but more importantly, the focus of this systematic review was on mental health only; other physical or learning disabilities were included only if they co‐existed with mental health problems. Fifteen studies were included in the review and the main findings showed that individualized funding can have positive outcomes for people with mental health problems in terms of choice and control, impact on QoL, service use and cost‐effectiveness (Coyle, 2009; Davidson et al., 2012; Glendinning et al., 2008; Spandler & Vick, 2004). However, methodological shortcomings, such as variation in study design, sample size and outcomes assessed, were reported to limit the extent to which the study findings could be accurately interpreted or generalized. This was compounded by considerable variation in the support models included, but without any attempt to undertake a sub‐group analysis (e.g., ‘Personal Budget’ versus ‘Direct Payment’ versus ‘Recovery Budget’ versus ‘Cash and Counselling’). Consequently, the authors concluded that more large, high quality, experimental studies were required before any definitive conclusions could be reached (Webber et al., 2014).

#### 3.3.2 Contribution of this review

We are not aware of any systematic review that focuses on the effectiveness of individualized funding in relation to people with a disability of any kind, including mental health problems. Given the new policy imperative around individualized funding and the growing pool of studies in this area, there is now a need for a systematic review of these models (when compared to a control) across a spectrum of disabilities, in order to assess their effectiveness in relation to health and social care outcomes. A supplementary synthesis of the non‐controlled evaluations and qualitative studies was also included in order to capture these findings in an area that is relatively new. Due to the complex nature of implementing novel initiatives that challenge the status quo, many qualitative studies have been undertaken to capture important perspectives, successes and challenges and these cannot, therefore, be overlooked in this review.

This review: (a) assesses the effectiveness of individualized funding interventions; (b) reports subgroup differences in order to explore how effects may differ by various client and intervention parameters; and (c) appraises and synthesizes the experiences of key stakeholders. The ultimate aim of this review is to provide useful, robust and timely data to inform service providers/organizations working in the field of disability and to provide a rigorous evidence base on which decisions by policy makers (and drivers) can be made around different resource allocation/individualized funding models to support greater choice and control by individuals in their daily lives.

## 4 OBJECTIVES

### 4.1 Objectives of the review

The objectives of this review are to: (a) examine the effectiveness of individualized funding interventions for adults with a lifelong disability (physical, sensory, intellectual, developmental or mental disorder), in terms of improvements in their health and social care outcomes when compared to a control group in receipt of funding from more traditional sources; and (b) to critically appraise and synthesize the qualitative evidence relating to stakeholder perspectives and experiences of individualized funding, with a particular focus on the stage of ‘initial implementation’ as described by Fixsen et al. (2005).

Most interventions included in the synthesis, at a minimum, should have reached initial implementation. Unsurprisingly, this is often the most challenging stage of implementation. Fixsen et al. (2005) describe initial implementation as complex process, requiring ongoing/multi‐level change (e.g., individual, environmental and organizational) that is not necessarily linear and which is influenced by external administrative, educational, economic and community factors. As a result, it is during this stage that stakeholders can encounter/experience the most fear of change or inertia. The next stage of implementation, ‘full operation’, cannot be initiated until the challenges associated with initial implementation are overcome and associated learnings are integrated into policy and practice.

Key questions include:

- What model of personal budget (e.g., direct payment or facilitated) is relatively more effective at improving health and social care outcomes?
- Do support structures such as resource allocation systems, needs assessments, support planning and review affect intervention effectiveness?
- How is the intervention effect linked to length/intensity of intervention?
- Is the intervention effect linked to type and/or severity of presenting disability (e.g., physical, sensory, intellectual, developmental or mental disorder)?
- Is the effect linked to implementation fidelity (e.g., does level of staff knowledge, access to independent information, advice, training and support affect intervention effectiveness)?
- Does the effect differ depending on the level of support available from non‐paid advocates (e.g., friends and family)?
- Do socio‐demographic factors, (e.g., age, race/ethnicity, sexual orientation, gender, religious beliefs, household income, urban/rural setting) impact on intervention effectiveness?
- What are the experiences, barriers and facilitators associated with the implementation of individualized funding initiatives for people with a disability or mental health problem?
- What is the economic impact of the intervention from both a service user and public service perspective?

## 5 METHODS

### 5.1 Criteria for considering studies for this review

#### 5.1.1 Types of studies

Eligible study designs for questions relating to the effectiveness of the individualized funding intervention included randomized, quasi‐randomized and cluster‐randomized controlled trials. Due to the complex nature of the intervention and attendant ethical constraints, randomization may not be possible since the aim of individualized funding is to increase choice and control, and randomization limits this option. Therefore, non‐randomized studies (e.g., controlled before and after studies, cross‐sectional surveys, longitudinal studies or cohort studies) were considered in this part of the review. Randomized and non‐randomized studies are reported separately. A key feature across the studies was the presence of a control group, in order to ascertain differences across a set of health and social care outcomes. As such, single‐case designs, pre‐post studies without a control group, non‐matched control groups, or groups matched in a post‐hoc way after results were known, were excluded from the review.

For the qualitative synthesis, eligible studies included: ethnographic research; phenomenology; grounded theory; participatory action research; case studies; or mixed methods studies in which qualitative approaches were used to gather data. Methods used to collect the qualitative data in primary studies included: interviews; focus groups; observation; open‐ended survey questions; and documentary analysis.

#### 5.1.2 Types of participants

##### Population Inclusion criteria

- Adults aged 18 years and over receiving a personal budget
- Where the study has categorized the person as having:
  ○ any form or level of physical, sensory, intellectual or developmental disability
  ○ any form or level of mental health problem, disorder or illness
  ○ dementia
- Residing in any country
  ○ Residing in any type of residential setting (own home, group home, residential care setting, nursing home, hospital, institution)

##### Population Exclusion criteria

- Minors under the age of 18 since the decisions around their daily lives are ultimately made by a parent or legal guardian.
- Older people (>= 65) who have a disability, but where it was not present for at least ten years of their working‐adult life. Such disabilities would generally be age‐related, such as frailty or difficulty with completing Activities of Daily Living, and are not the focus of this review
- Privately funded individualized funding interventions.

#### 5.1.3 Types of interventions

Any form of personal budget or individualized funding which is state funded directly or indirectly.

For the quantitative element of this review, where a control group exists, support services may take two forms: (a) traditional ‘services as usual’ (e.g., predetermined group activities, provided in a congregated setting and financed through block funding to service providers whereby previous annual spend for a service provider is used to estimate the required funding for the upcoming year (NDA, 2011); or (b) a different type of personalized support which does not include a personal budget where, for example, a service user might access services through a congregated setting where finances are centralized, but where an individualized plan is used to determine service user needs and preferred activities. However, the individualization of planned responses may be limited, for example, by majority preferences within the group, staffing limitations or pre‐existing service options.

Individualized funding interventions were excluded where the budget was provided to families, guardians/other carers (only), or where the person with a disability did not have an active role in the decision making and planning process and could not exercise control over the use of funds. However, studies were included where an advocate was managing the funds after an individual assessment of need took place and provided that the funds were being used to meet the needs identified during the assessment.

A personal budget provided by the person's family or by another private means was not included, as this review focuses on use of public funds for people with a disability. Furthermore, private sources of funding introduce confounding factors which would lead to uncontrollable bias.

#### 5.1.4 Types of outcome measures

##### Primary outcomes

The primary outcomes of interest (i.e. pertaining to the quantitative studies) are ‘Quality of Life’ and ‘Client Satisfaction’. Each is described in more detail below.

- **Quality of life**, including: physical health; psychological health; well‐being; social relationships; personal and life satisfaction; and environment or disability‐specific QoL including: choice; control over daily living; autonomy; social acceptance; social network and interaction; social inclusion and contribution; future prospects; communication ability; safety and personal potential. Typical measures include the WHO Quality of Life Disability module (M.J.Power & Green, 2010) and the ASCOT (Malley et al., 2012).
- **Client satisfaction**, as measured by access to and continuity of care, shared decision making, level of choice, control and self‐determination, planning, coordination and review of care, respect shown, information provided, staff attitudes and responsiveness, physical and emotional comfort; encouragement, opportunities for positive risk‐taking, risk management, availability of services, staff training and management, cost and administrative burden. The Consumer Assessment of Healthcare Providers is an example of a set of satisfaction scales which measure and evaluate various aspects of consumers’ experiences of health care, including a tool for measuring: health plans; group and individual service providers; hospitals; nursing homes; and behavioural health services (Kane & Radosevich, 2011b).

##### Secondary outcomes

- **Physical functioning,** measured by Activities of Daily Living (ADL), such as: bathing; dressing; feeding; transfer; toileting or advanced independent living activities such as: shopping; doing chores; and cleaning. These can be measured using, for example, the Katz Index of ADLs (Katz, Ford, Moskowitz, Jackson, & Jaffee, 1963 as cited in Kane & Radosevich, 2011a).
- **Costs data**, measured for example by: size of personal financial package available; brokerage/management fees; cost of individual services; and cost of recruiting staff (for self‐managed).

##### Adverse outcomes

- **Adverse psychological impact**, as measured by symptoms of depression, anxiety, stress, social dysfunction, and feelings of isolation. Depression can be measured as clinical (e.g., the Hamilton Rating Scale) or non‐clinical depression (e.g., Carroll Rating Scale;Kane & Radosevich, 2011a) or can be disability specific (e.g., Glasgow Depression Scale for people with a Learning Disability) (Cuthill, Espie, & Cooper, 2003). Anxiety may have been measured for example by general anxiety scales such as the Anxiety Adjective Checklist or Zung's Self‐Rating Anxiety Scale (Kane & Radosevich, 2011a) or the Glasgow Anxiety Scale for people with a learning disability (Hermans, van der Pas, & Evenhuis, 2011).

##### Qualitative data

- For the qualitative synthesis, outcomes or phenomena of interest involved the experiences of stakeholders in receiving and implementing a personal budget. Stakeholders include the client, family members, advocates, personal assistants/key workers, professional staff such as occupational therapists or physiotherapists and other members of the community involved in the process.

#### 5.1.5 Duration of follow‐up

The intervention should be in place for at least 6 months before follow‐up. This does not apply in the case of qualitative studies.

### 5.2 Search methods for identification of studies

The Campbell Collaboration policy brief for searching studies and information retrieval, informed the search strategy as presented below (Hammerstrøm, Wade, Hanz, & Klint Jørgensen, 2009). In addition, an information retrieval specialist within Maynooth University was consulted during the preparation of search strings, while several search retrieval specialists provided recommendations during the peer‐reviewing process (of the study protocol). Padraic Fleming, the lead author, conducted the searches once the protocol had been peer‐reviewed and approved by Campbell Collaboration. The searches were conducted during the period February 19th and March 9th, 2016. At the end of the screening process, key journals were searched using key‐terms up to the end of January 2017. Studies in any language and from any country were included, provided the abstract was in English.

Searches were completed, as per protocol with a number of minor additions. In some cases the search string could be copied and pasted directly from the protocol, whilst other databases required the search string to be manually populated. As recommended by Higgins and Green (2011), the search strategy is reported in Appendix 1, with any changes to protocol highlighted in bold text. The search strategy is reported (exactly) for each database utilized. This ensures that all searches are reproducible. Furthermore, details of additional grey literature databases are included (highlighted in bold), as recommended by Campbell Collaboration information retrieval specialists.

#### 5.2.1 Electronic searches

A selection of electronic search databases relevant to the area of study was searched. Where available, database thesauri were used to identify database specific terms for inclusion. These terms were ‘exploded’ to encompass all narrower terms when appropriate to do so. These terms also helped in the identification and inclusion of all possible synonyms. In addition to these database specific terms, free text terms which were identified from within the current literature were used to further broaden the search.

The follow databases/search engines were searched:

1. CINAHL (Cumulative Index of Nursing and Allied Health Literature)
2. EMBASE
3. Medline First Search
4. ASSIA (Applied Social Sciences Index and Abstracts) (Centre for Reviews and Dissemination, 2009)
5. PsycInfo
6. SCOPUS
7. Sociological Abstracts
8. Worldwide Political Science Abstracts
9. EconLit with Full text
10. Business Source Complete
11. Greylit
12. OpenGrey.eu
13. ProQuest Dissertations and Theses
14. Google Scholar
15. Google
16. Australian Policy Online
17. VHL Regional Portal – Latin America database
18. NORART (Norwegian and Nordic index to periodical articles)
19. Theses Canada

##### Search terms

The terms used to customize the search string for specific databases were based on the ‘population’ and ‘intervention’ of interest. ‘Disability’ and all possible variations including mental health, disorders and autism was the first keyword. Where available, database‐specific terms were used, encompassing all types of disability (see extensive list for PsychInfo – Appendix 1). Any overarching terms, encompassing all disabilities – when available – were exploded (see Embase search string in Appendix 1). ‘Budget’ and all its variations was the second keyword. The following truncations: ‘person*’; ‘individ*’; and ‘self‐direct*’ were used to refine the results pertaining to the main keywords, linking them when necessary to the main keywords with, for example, ‘near/n’ or ‘w/n’, where possible. All other keywords were connected with ‘or’/‘and’ when searching titles and abstracts. Search terms were also truncated, when appropriate, to allow for variations in word endings and spellings. Truncation conventions were specific to the database searched. A list of free‐text terms identified in the literature was used to supplement the syntax developed. The term ‘self‐determination’ (‘self‐determin*’) was added to the free‐text terms in addition to the terms outlined in the protocol. Individual studies and systematic reviews already known to the authors were used to check the sensitivity of search strings developed (Carter Anand et al., 2012; Webber et al., 2014).

Study design and outcomes were not included as part of the search strategy as it was anticipated that this would potentially lead to the omission of relevant studies. Furthermore, the mixed methods approach on which this review is based, led to broad inclusion criteria for study designs (Appendix 2 – methods paper).

All search strings are provided in Appendix 1. A sample search string is outlined below:

‘intellectual impairment’/exp OR ‘disability’/exp OR handicap OR ((people OR person* OR individ*) NEAR/3 (disabil* OR disable*)):ab,ti OR insanity OR (mental NEAR/1 (instability OR infantilism OR deficiency OR disease OR abnormality OR change OR confusion OR defect* OR disorder* OR disturbance OR illness OR insufficiency)):ab,ti OR (psych* NEAR/1 (disease OR disorder* OR illness OR symptom OR disturbance)):ab,ti AND (‘financial management’/exp OR ((budget OR finance* OR fund* OR resource OR money OR income OR purchas* OR broker* OR salary OR capital OR investment OR profit) NEAR/3 (individual* OR person*)):ab,ti) OR ‘cash for care’:ab,ti OR ‘consumer directed care’:ab,ti OR ‘direct payment’:ab,ti OR ‘indicative allocation’:ab,ti OR ‘individual budget’:ab,ti OR ‘individual service fund’:ab,ti OR ‘managed account’:ab,ti OR ‘managed budget’:ab,ti OR ‘notional budget’:ab,ti OR ‘personal budget’:ab,ti OR ‘personal health budget’:ab,ti OR personalisation:ab,ti OR ‘personalised care’:ab,ti OR personalization:ab,ti OR ‘person centred’:ab,ti OR ‘pooled budget’:ab,ti OR ‘recovery budget’:ab,ti OR ‘resource allocation system’:ab,ti OR ‘self‐directed assessment’:ab,ti OR ‘self‐directed care’:ab,ti OR ‘self‐directed support’:ab,ti OR ‘support plan’:ab,ti OR ‘virtual budget’:ab,ti OR ‘disability living allowance’:ab,ti OR ‘self‐determin*’:ab,ti AND [1985–2015]/py

##### Grey literature

An international list of grey literature databases published by the Campbell Collaboration (Hammerstrøm et al., 2009) was consulted in the first instance. A US electronic database, run by The New York Academy of Medicine and dedicated to specifically searching grey literature in public health, was also employed (www.greylit.org). Opengrey.eu was used to search grey literature in Europe. Other international grey literature databases utilised, as recommended by Hammerstrøm et al (2009) included: VHL Regional Portal for Latin American databases; NORART capturing Norwegian and Nordic articles; and Australian Policy Online. Boolean operators are not supported by these databases; therefore keywords, based on the database searches of published work, were searched separately (Appendix 1). Similar search strategies were employed for other country/region specific sites.

Timelines and other restrictions were not imposed in order to maximize the results from grey literature. Reference lists from relevant studies and previous systematic reviews were visually scanned to identify any unpublished literature not previously identified. Google Scholar, the popular internet search engine, was also used to search the terms developed for the academic databases in order to identify any relevant web materials or organizational/governmental reports which are unpublished or not accessible through electronic databases. ProQuest Dissertations and Theses was used to search for relevant theses at doctoral and masters level. Finally, Google search engine was searched to identify any relevant conference proceedings and government documents in addition to relevant NGOs that may have potentially useful research materials unpublished elsewhere. In total, 1,000 Google results and almost 6,000 Google Scholar titles were scanned (Appendix 1).

#### 5.2.2 Cross‐referencing of bibliographies

The references of each of the final studies included in the review were scanned to identify any additional potentially relevant studies. Literature reviews and other non‐eligible studies were also scanned for relevant titles. This forward citation searching led to the addition of 40 additional the full‐text screen. The bibliographies from the two previous reviews were also cross‐referenced (Carter Anand et al., 2012; Webber et al., 2014).

#### 5.2.3 Conference proceedings and experts in the field

Conference proceedings such as the extensive syllabus from the recent international conference hosted by The University of British Columbia's Centre for Inclusion and Citizenship (‘entitled Claiming Full Citizenship: Self Determination, Personalization, Individualized funding) were consulted. This syllabus provided slides from over 100 presentations and contact details for research and practice experts from around the world who specialize in the delivery of individualized funding, self‐determination and personalization of services for people with a disability. This syllabus was used as a reference point for identifying and sourcing data from unpublished or ongoing studies and guided the hand‐searching. Such hand searching led to the addition of 63 to the full‐text screen.

Corresponding authors as listed on published works were contacted, when necessary, to request access to primary data, and/or to provide clarification during the data extraction process on, for example, demographic information and timelines to follow‐up.

#### 5.2.4 Timeframe (and other filters)

According to Leece & Leece (2011), the origins of personalized brokerage schemes and individualized funding can be traced back to the mid‐1980s in to the USA. Around the same time (1988), legislation in Western Australia introduced a form of personal budget known as the Local Area Coordination charter which facilitated a mechanism for ‘Direct Consumer Funding’ (Carter Anand et al., 2012). Thus, individualized funding appears to have emerged for the first time, around the mid‐eighties. For this reason, the searches of published literature were limited to the period 1985 – quarter 1 of 2016. For example, date filters were applied to the Scopus search results (Appendix 1). Other filters were also applied where necessary to refine the search, such as exclusion of non‐relevant subject areas (See Embase search string Appendix 1).

#### 5.2.5 Manually browsing key journals

Toward the end of the data retrieval process, the most recent issues of key journals (i.e., those that produced the most studies in the meta‐analysis) were searched manually to capture any relevant work published since the searches were last run. Seven journals were searched including: (a) British Journal of Social Work; (b) Disability and Society; (c) Health and Social Care in the Community; (d) Health Services Research, Journal of Integrated Care; (e) Journal of Health Services Research & Policy; (f) International Journal of Mental Health Systems; and (g) International Journal of Mental Health Systems. Key terms were used to search these journals resulting in the addition of two titles to full‐text screen (Appendix 1).

### 5.3 Data collection and analysis

#### 5.3.1 Data extraction and study coding procedures

As outlined in the protocol, titles were reviewed initially in Endnote by the lead author to remove any studies which were clearly irrelevant (e.g., non‐human or pharmaceutical studies). However, due to the very large number of search results (*n* = 82,274 after duplicates and non‐relevant grey literature excluded), an extensive, thorough and transparent ‘results refinement process’ was developed. In summary, this included a three‐part process of (a) automatic text mining; (b) a failsafe check (to catch any studies inadvertently removed); and (c) a manual title screen. This process is detailed in Appendix 2. Excluded studies can be seen in Appendix 5.

Following this, the screening of studies in relation to inclusion/exclusion was undertaken in two stages. The first stage involved citation and abstract; the second involved full text documents. Three independent researchers (PF, MH, SOD) were involved at each stage. Both PF & MH were co‐authors of the protocol, but all three had a deep understanding of the research questions and outcomes of interest. SOD was recruited as a third screener, due to the intensive nature of the screening process. PF screened all titles and MH/SOD acted as second screeners. Prior to data extraction and coding, the three independent reviewers met to discuss and pilot the extraction and coding procedures on a sample of abstracts. While PF reviewed all materials, MH and SOD acted (alternately) as intermediaries to resolve any disagreements between PF and the second reviewer in question (e.g., MH acted as an intermediary for, the albeit very small number of, disagreements between PF and SOD, where a resolution could not be agreed through discussion and consensus). This occurred on approximately 20 occasions (0.3%). Inter‐rater reliability was calculated for the full sample of full‐text papers screened using kappa statistic (as recommended). Values of kappa between 0.40 and 0.59 reflect a fair level of agreement between reviewers, whilst values from 0.60 to 0.74 reflect good agreement; 0.75 indicates excellent agreement (Higgins & Green, 2011; Chapter 7.2.6). The inter‐rater reliability score is reported in the results section below.

To pre‐empt such disagreements, both reviewers discussed the inclusion/exclusion criteria, as set out in the protocol, and the various tools used to assess study quality and risk of bias. Any potential differences in interpretation were discussed and resolved insofar as possible. A number of known studies were used to pilot the data extraction and coding procedures in order to support this process.

##### Stage one: citation and abstract

Citations and abstracts which passed the first stage were retrieved in full text for a more comprehensive review. In order to pass stage one the citation or abstract must answer ‘Yes’ or ‘Unsure’ to all the questions below:

1. Has an individualized funding intervention been utilized?
2. Is the study population aged over 18 years of age?
3. Does the study population have any form of physical, sensory, intellectual or developmental disability, dementia or mental health problem, disorder or illness?
4. Does the personal budget originate from public funds, directly or indirectly?
5. Has a study design been adopted which collected and analyzed empirical data?

If reviewers were unsure, full text articles were retrieved to clarify and, if necessary, the corresponding author was contacted.

##### Stage two: full‐text

Full text documents were retrieved for all documents that passed stage one. Two reviewers independently evaluated all studies. Studies had to meet all of the inclusion/exclusion criteria set out previously in order to advance to full review. It should be noted that not all studies precisely met the inclusion criteria; for example, the study population may have included minors, adults and older people without lifelong disabilities. Where this occurred, studies were included if the eligible population represented the majority of respondents (>50%) and where it was possible to disaggregate the findings. Reasons for exclusion were independently reported by both reviewers in the ‘research notes’ field within endnote reference manager. For studies that were included in the review, a standard set of data are reported such as: publication details; study design; participant demographics, intervention and control descriptors; and outcome measures and related statistical differences between intervention and control groups (see Tables 2, 3, 4, 5 in results section).

**Table 2:** Characteristics of included quantitative studies

Characteristic	*19 titles (%) [7 studies]*	Characteristic	*19 titles (%) [7 studies]*
Publication Year		**Intervention Type**	
1992–1999	3 (16) [1]	Consumer‐directed	14 (74) [4]
2000–2005	7 (37) [2]	Self‐determination	1 (5) [1]
2006–2010	6 (32) [3]	Individual Budget	3 (16) [1]
2010–2016	3 (16) [1]	Personal Budget	1 (5) [1]
Characteristic	**7 Studies N (%)**	**Characteristic**	**7 Studies N (%)**
Geographic Region		**Disability Type (primary)**	
Australia/NZ	0	Physical/Sensory	2 (29)
Europe	2 (29)	Learning/Developmental	0
Canada	0	Mental Health	1 (14)
United States	5 (71)	Various	4 (57)
Study Design		**Sample Size**	
Randomised/random sampling	6 (86)	<1000	3 (43)
Non‐randomised	1 (14)	1001 ‐ 2000	3 (43)
Language		>= 2000	1 (14)
English	7 (100)		
Non‐English	0		

**Table 3:** Quality scores for quantitative studies

Study	Score from ‘Quality Assessment Tool for Observational Cohort and Cross‐Sectional Studies’
1^st^ Author (year)	
Beatty (1998)	6/10 (4 NA) = 60% (Fair)
Benjamin (2000)	7/10 (4 NA) = 70% (Good)
Conroy (2002)	6/10 (4 NA) = 60% (Fair)
Brown (2007)	7/11 (3 NA) = 64% (Good)
Caldwell (2007)	6/10 (4 NA) = 60% (Fair)
Glendinning (2008)	8/11 (3 NA) = 73% (Good)
Woolham (2013)	3/10 (4 NA) = 30% (Poor)
	The aims of the study are not clearly stated. While random assignment was used, the definition of the control group is ill‐defined. There is no discussion of statistical power in relation to sample size. The two groups were considered broadly comparable on a number of demographic factors but no statistical data are presented.

*Note*: Poor ‐ <40%, Fair – 40% ‐ 60%, Good – 61% ‐ 80%, Excellent ‐ >80%.

**Table 4:** Summary of outcomes across 7 included studies

Study – 1st Author	Outcome					
	Quality of life	Client satisfaction	Physical functioning	Cost effectiveness	Adverse	Other
Beatty (1998)	NR	+++	NR	NR	NR	NR
Benjamin (2000)	NR	m1: +++	NR	NR	m1: ‐‐‐	Safety/Security: +
		m2: +++				
					m2: ND	
		m3: +++				
					m3: ND	
		m4: +++				
		m5: ND				
Conroy (2002)	ND	NR	NR	NR	ND	ID
Brown (2007)	+++ (x2)	m1: +++ (x2)	NR	m1: ND (x2)	m1: ++ (x1)	NR
					m1: + (x2)	
					m2: ++ (x2)	
		m1: ++ (x1)				
					m2: ND (x1)	
				m1: ‐ ‐ (x1)		
		m2: +++ (x2)				
					m3: ++ (x1)	
					m3: ND (x2)	
	++ (x1)					
		m2: ++ (x1)				
				m2: ‐ ‐ (x3)		
					m4: + (x1)	
					m4: ND (x2)	
					m5: + (x1)	
					m5: ND (x2)	
					m6: + (x1)	
					m6: ND (x2)	
Caldwell (2007)	NR	+++	NR	NR	++	Comm. Participation: ND
Glendinning (2008)	m1: ND	+	NR	m1: ND	ND	Self‐perceived health: ND
	m2: ND					
				m2: ND		ASCOT: ND
Woolham (2013)	+++	NR	ND	m1: ID	NR	NR
				m2: ID		

*Note*: ‘+’, ‘++’, ‘+++’: Significant differences in favour of the intervention group representing significance level <.05, <.01, and <.001 (respectively).

‘‐’, ‘‐‐’, ‘‐‐‐’: Significant differences in favour of the control group representing significance level <.05, <.01, and <.001 (respectively).

m1/m2: Different measures of each outcome, within the same study.

(x2)/(x3): Multiple study sites, within the same study.

Abbreviations: ND, No difference between intervention and control groups; NR, Not reported; ID: Insufficient data.

**Table 5:** Characteristics of included qualitative studies

Characteristic	*69 studies (%)*	Characteristic	*69 studies (%)*
Publication Year		**Geographic Region**	
1992–1999	6 (9)	UK	41 (59)
2000–2005	16 (23)	United States/Canada	17 (25)
2006–2010	23 (33)	Australia	7 (10)
2010–2016	24 (35)	Other European	4 (6)
Intervention Type		**Disability Type**	
Direct/In‐direct payment	21 (30)	Various	41 (59)
Self‐directed/determination/managed	12 (17)	Mental Health/Dementia	10 (14)
Personal Budget	12 (17)	Physical/Sensory	7 (10)
Individual Budget	7 (10)	Learning	5 (7)
Mixed/Other	17 (25)	Not specified	6 (9)
Study Design		**Sample Size**	
In‐depth interviews	20 (29)	<25	24 (35)
Mixed qualitative	19 (28)	26–50	16 (23)
Case study (mixed methods)	18 (26)	51–100	16 (23)
Survey (8 primarily quant.)	9 (13)	>= 101	13 (19)
Other	3 (4)		
Language			
English	68 (99)	Non‐English	1 (1)

#### 5.3.2 Risk of bias

Risk of bias and quality of research were evaluated using a range of tools (depending on study design) by one reviewer (PF), except for a single paper (co‐authored by PF), which was reviewed by a second independent reviewer (MH). While a detailed assessment was conducted by one reviewer, the intensive screening process involved a quality assessment – with the screening tool adapted to solicit feedback on study quality, particularly in relation to methodological considerations (see 5.3.5). These assessments were discussed among the team of reviewers. The main areas of bias include: selection bias; performance bias; detection bias; attrition bias; and reporting bias (Higgins & Green, 2011; Chapter 8).

‘The Cochrane Collaboration's tool for assessing risk of bias’ was used to appraise randomized, quasi‐randomized and cluster‐randomized controlled trials. The protocol specified that all nonrandomized study designs would be appraised for quality and risk of bias using the appropriate tool from the Critical Appraisal Skills Programme (Critical Appraisal Skills Programme, 2014) However, upon application of the CASP, the criteria for measurement of quality did not seem appropriate or well matched to the study designs utilised in the included quantitative studies. For example, CASP does not have a specific tool for before and after studies or controlled cross‐sectional surveys, (the most common designs utilised by eligible studies). Consequently, various tools were researched, sourced and piloted before selecting the “Quality Assessment Tool for Observational Cohort and Cross‐Sectional Studies” (NHLBI, 2014). This tool was chosen due to its flexibility in terms of application, with clear guidance provided for how to treat criterion not relevant to the study design (e.g., measurement of exposure is not relevant for this intervention, but can be marked as ‘No’ or ‘NA’). It should also be noted that the use of the Grading of Recommendations Assessment, Development and Evaluation (GRADE) for quantitative data was deemed inappropriate since the fundamentally diverse nature of the available studies/data precluded the possibility of a meta‐analysis.

As per the protocol, CASP tools were used to assess quality and risk of bias for qualitative studies. The results of such assessments are presented under the CerQual headings of: ‘methodological limitations’, ‘relevance’, ‘adequacy of data’ and ‘coherence’ (Lewin et al., 2015). Risk of bias is discussed in detail for both quantitative and qualitative studies in the results section to follow. (Figures 2 and 3 and Appendixes 6 and 7).

#### 5.3.3 Synthesis procedures and statistical analysis

The interventions included in this review, whilst very diverse, successfully met the eligibility criteria outlined in the protocol as did the population of interest, comparison groups and outcomes. However, as data extraction progressed, it became apparent to the screeners that a wide range of economic, social and political factors, identified across different geographical contexts and jurisdictions, had hugely impacted design and delivery of the interventions. A number of examples will be described below to illustrate these differences.

- The population and comparison groups were very disparate, across the seven quantitative studies. For example, two quantitative studies focused solely on people with physical disabilities, whilst another was investigating only people with mental health problems. The remaining four studies represented people with various types of disabilities.
- Another example related to the comparison groups. For two studies, control group members wanted to avail of individualized funding, but were on a waiting list. For other studies, people were ‘happily in receipt of similar agency based services, rather than self‐directed, while a third approach involved random assignment to intervention or control group.
- Finally, the ways in which people accessed funding were vastly different, whereby participants in the US studies had to meet pre‐defined ‘Medcaid’ eligibility criteria, while UK participants often had multiple funding sources available to them, such as ‘Disability Living Allowance’, ‘Independent Living Fund’, and ‘Direct Payment’. The disparities in funding availability and allocation directly impacted the design and evaluation of included studies.

The differences documented above inherently affected the design and delivery of the intervention. Furthermore, study designs and data collection tools were vastly inconsistent, with most outcome measurement tools designed specifically for the study in question. As a result, it became apparent that the planned synthesis of quantitative data, as per the protocol, was not going to be feasible or meaningful. The above factors also affected the risk of bias (Figure 2), although quality scores remained reasonably good (Appendix 6). Therefore, a narrative analysis of quantitative data, as described below, was undertaken to best represent the results. Summary statistics for all studies are reported in Table 4.

##### Narrative analysis

Narrative systematic reviews serve several functions including reporting the effects of interventions as well as the factors impacting the implementation of interventions (Popay et al., 2006). Therefore, such an approach was well suited to the current review which aimed to examine quantitatively, the effects/impact of individualized funding whilst also qualitatively assessing factors related to implementation. The use of a narrative analysis viz‐à‐viz the quantitative data allowed for a coherent blending of findings within the mixed methods approach, particularly given the emerging contextual diversity. Specifically, this involved the following four main elements as identified by Popay et al. (2006): (a) developing a theory of how the intervention works, why, and for whom; (b) developing a preliminary synthesis; (c) exploring relationships in the data; and (d) assessing the robustness of the synthesis.

###### Continuous data

Ultimately, the original data were reported for each study in relation to the primary, secondary, adverse and other health and social care outcomes of interest. In those cases where no data were available, *p*‐values were calculated using the RevMan tTest calculator.

###### Dichotomous data

In cases where binary or categorical data were used to compare intervention and control groups, Upton *χ*² was employed to test for significant between group differences (i.e., between the proportions of the two independent groups), as recommended by Campbell (I. Campbell, 2007). WinPepi (Abramson, 2011) was used for such calculations.

#### 5.3.4 Treatment of qualitative research

##### Meta‐synthesis

Two complementary approaches were utilized sequentially in this review in order to manage the qualitative data. Firstly, a meta‐aggregation or metasynthesis was conducted, involving a comprehensive and systematic search, data appraisal, and extraction process using standardized tools where appropriate. Secondly, a standard thematic analysis was conducted to aggregate the findings from several studies. This involved four stages as recommended by Clark (2015), each of which is described below.

*Reading and coding the studies.* Each eligible study included in the systematic review was read carefully and in detail. The main study characteristics are reported in Table 5. A thematic analysis was conducted for each individual study, at this stage, in order to identify the main themes reported. Line‐by‐line coding of the results was undertaken using MAXQDA, followed by an organization of the codes into descriptive themes (MAXQDA, 2014; Thomas & Harden, 2008).

*Determining relations.* Having identified the main themes reported in the results of individual studies, relationships between studies were explored. Common and recurring themes were categorized, leading to the development of analytical themes (Thomas & Harden, 2008). At this point, the CerQual score was also determined, (Appendix 7).

*Translating the studies.* Having read all the studies at least once, each study was re‐read to examine similarities and differences between the concepts.

*Synthesizing translations.* The studies were conceptually folded together, using the concepts from individual studies and the emergent analytical themes as a lens to understand the whole body of work, thereby producing new understandings and conceptual development (Clark, 2015).

#### 5.3.5 Methodological changes to the study protocol

A number of changes to the protocol were required for two main reasons: (a) the unexpected scope and resource intensive nature of the review (despite recruiting an additional screener); and (b) the inconsistency in study design, analysis and reporting which was further compounded by the lack of eligible quantitative data. Further information is provided below.

*Changes relating to the resource‐intensive nature of the review.*

- A ‘results refinement’ process was developed and agreed to manage and filter the unexpectedly high number of search results (Appendix 2).
- Data extraction was conducted by only one review member for the qualitative data only rather than the anticipated two, due to resource constraints. However, during full‐text screening, the second screeners indicated where data were not relevant (e.g., data related to minors/older people without a life‐long disability). These notes were captured in the screening form and were used to guide data extraction. Quantitative data were double extracted as per protocol.
- A detailed quality assessment was conducted by only one reviewer, rather than the anticipated two, due to resource constraints. The screening of data was prioritized and, in fact, the thorough screening process did, in part, assess the quality of studies with the exclusion of those that did not have sufficient methodological detail to assess eligibility. These decisions were discussed among the review team, based on data captured in the screening tool. To facilitate these discussions, changes were made to the screening tool (compared to that published in protocol), in order to capture more detail, particularly regarding outcomes and methodology (Appendix 8).

- The use of GRADE for quantitative data was deemed unnecessary due to the lack of data and meta‐analysis.

*Changes due to complex nature of study designs.*

- Eligibility criteria were amended (i.e., tightened or loosened) as deemed necessary given the complex nature of social interventions. This led to the exclusion of older people who did not have a ‘lifelong’ disability, but instead required age‐related support. It was felt that including an older population without a life‐long disability would add uncontrollable confounding factors to the analysis. In addition, the eligibility criteria were not always applied in a strict/absolute fashion; for example, studies involving minors were only included where data could be disaggregated and where the majority of respondents (>50%) met the eligibility criteria.
- The quality assessment using the CASP toolkit did not seem appropriate or well matched to the study designs utilized in the included quantitative studies. Therefore, various alternative tools were researched, sourced and piloted before selecting the ‘Quality Assessment Tool for Observational Cohort and Cross‐Sectional Studies’.
- Given that a narrative synthesis of quantitative data was deemed most appropriate, many of the intended analyses were not conducted (e.g., examining the impact of sensitivity analysis or publication bias).
- As a point of clarification, the minimum intervention time of 6 months was not imposed for qualitative studies because the focus of this aspect of the study was on early implementation.

## 6 RESULTS

The search strategy which guided this review was purposely broad in order to identify all eligible quantitative and qualitative studies. This focused on: (a) the population of interest (itself expansive), including adults (18 and over) with any form of lifelong disability, mental health problem or dementia; and (b) the intervention, of which there were many terms used to describe the funding of disability supports on an individual basis (using state funds). Study design, comparator groups or outcomes of interest were not included at search stage. A wide range of academic databases (including general, psychological, medical, social, economic, business and policy), regional specific databases, sources of grey literature and search engines, were employed to gather the data.

### 6.1 Results of search

Due to the breadth of the search strategy, 82,274 potentially relevant titles were identified. For this reason – and as agreed by the two lead authors – an additional refinement process was necessary in order to reach a manageable number for title/abstract screening. This robust and transparent three‐part refinement process is detailed in Appendix 2. In summary, it included: (a) automatic text mining; (b) failsafe check – for potentially relevant titles that may have inadvertently been removed; and (c) manual title screen for clearly irrelevant titles.

**Figure 1:**
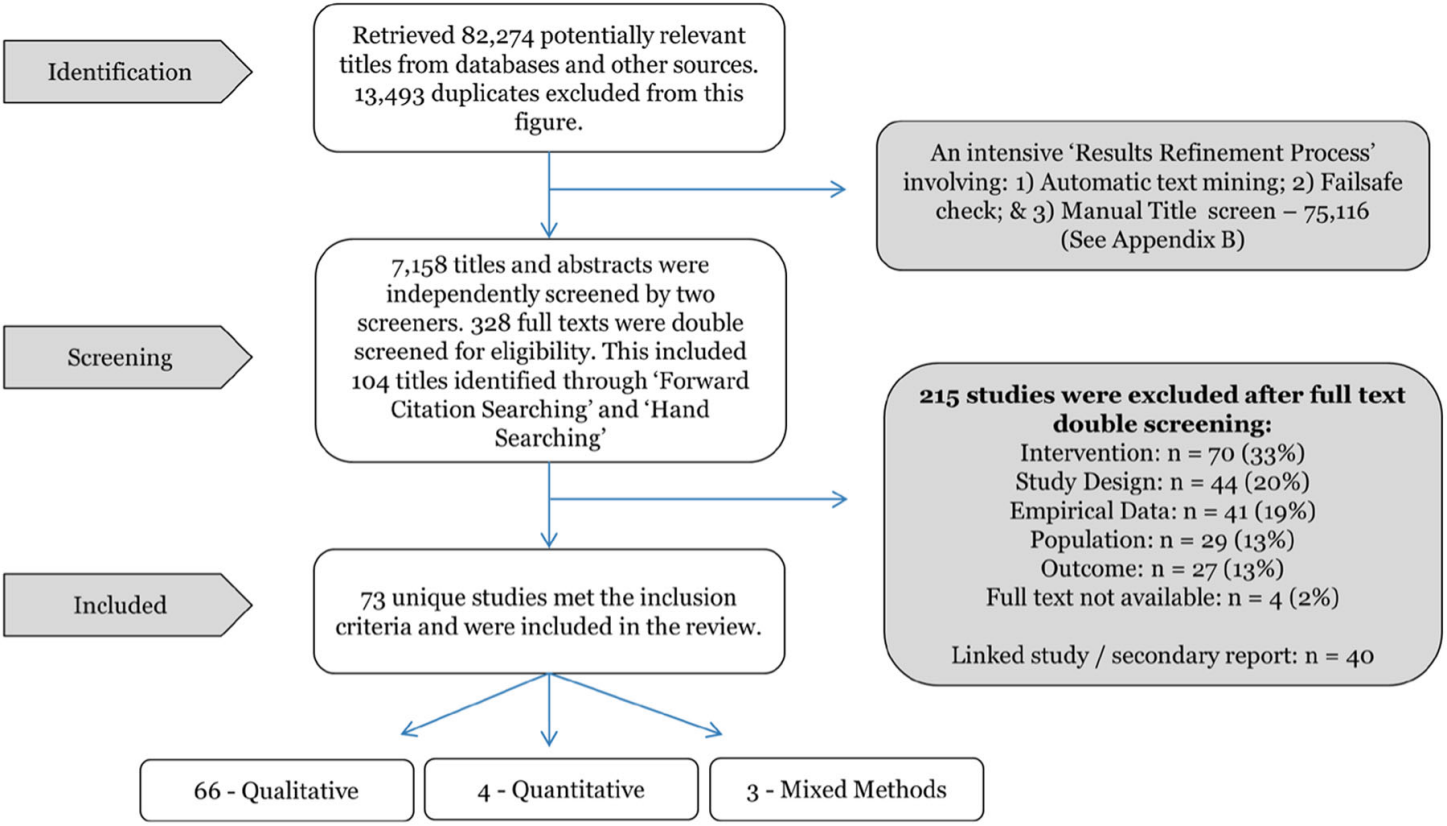
Flow chart of study selection process

After this search refinement process was complete, 7,158 titles and abstracts were double screened for relevance. A total of 6,934 were excluded, as they did not meet the inclusion criteria. The full texts of 224 titles were double screened as well as 104 titles identified through ‘forward citation searching’ and ‘hand searching’. In total, 328 full texts were double screened. Appendix 5 outlines the reasons for exclusion. A total of 73 studies met the inclusion criteria and were included in the review, 66 (90%) of which were qualitative in nature. See Figure 1 for a summary of the study selection process.

#### 6.1.1 Included studies

As indicated above, a total of 73 studies were included, 66 (90%) of which contained eligible qualitative data only. A further three, had employed a mixed methods design whereby both the quantitative and qualitative data were eligible. Only four studies (4/73) were solely quantitative in nature.

A number of country‐specific contextual factors impacted considerably on how the interventions of interest were described and implemented. For example, in some cases, both children and adults participated in the study. Where it was possible to disaggregate these data, the study was included, but the ineligible data were excluded. Therefore, the eligibility of many studies was unclear at first, resulting in an inter‐rater reliability score of 0.6 for the double screening of full‐texts (according to the criteria outlined by Higgins and Green (2011), whereby values from 0.60 to 0.74 reflect good agreement). In essence, this meant that 76 studies (23% of full‐text screen) required in‐depth discussion between two reviewers to resolve disagreements, while 21 (6%) were referred to a third reviewer.

#### 6.1.2 Qualitative data

Almost half (45%, 31/69) of the eligible studies containing qualitative data were solely qualitative in nature and had collected/accessed qualitative data in a number of ways, including in‐depth interviews, focus groups, workshops, telephone discussions, case studies, documentary analysis and open‐ended survey responses. The remaining 38 studies contained both qualitative and quantitative data but only three met the eligibility criteria for inclusion in the quantitative element of the review (*n* = 7). Six (primarily) quantitative studies also contained open‐ended responses providing eligible qualitative data. Thus, the text‐based data available for analysis varied considerably with a mean word count of c.9, 500 (ranging from c.556 to c.134, 260). Characteristics of the included studies containing eligible qualitative data (*n* = 69), can be seen in Appendix 3.

#### 6.1.3 Quantitative

A total of seven studies contained eligible quantitative data – four were based on solely quantitative designs and three on mixed methods approaches. The common methodological feature across the seven studies was the presence of a control group, in order to determine causal inference of an individualized funding intervention on a set of health or social care outcomes (outlined in 6.4). The study designs varied however. One was a ‘quasi‐experimental nonrandomized controlled longitudinal survey’, three were ‘controlled cross‐sectional surveys of random sample’ and three were ‘randomized controlled before and after studies’. A meta‐analysis was not possible due to the fundamental incompatibility of variables across all seven studies (e.g., inconsistent and unstandardized measurement and reporting of data). Therefore, a narrative review was undertaken based on the outcomes of interest. Characteristics of the studies containing eligible quantitative data (*n* = 7) are provided in Appendix 4.

#### 6.1.4 Mixed methods studies

As indicated above, mixed methods approaches were used in 38 of the 69 studies containing eligible qualitative data, only three of which contained quantitative data which were eligible for inclusion in the review.

#### 6.1.5 Excluded studies

In total, 215 studies were excluded during full‐text screen while a further 40 were identified as a secondary title linked to a study already captured within the review. Unique data from these 40 studies were included in the data synthesis. The largest proportion of excluded studies (33%, 70/215) were excluded primarily because they did not meet the definition of the intervention as described earlier. The remaining studies were excluded for a number of reasons including issues related to: study design (i.e., not a controlled study or unrelated qualitative focus; *n* = 44); empirical data (i.e., reporting data from previously published studies; *n* = 41); population (i.e., involving minors or older people without a lifelong disability or dementia; *n* = 29); and outcome (i.e., not measuring an outcome of interest; *n* = 27). A full list of excluded studies, including the reason for exclusion, can be seen in Appendix 5.

### 6.2 Description of included quantitative studies

Seven studies with unique quantitative data, representing 19 titles, were included in the review. One was an unpublished report (available online), while the remaining five included both unpublished reports and published peer‐reviewed journal articles. All studies were written in English and the majority (71%) were based in the United States (*n* = 5). Two (29%) were conducted in England. Sample sizes ranged from 92to 1,966, with a mean sample size of 761. The studies measured one or more outcomes of interest including quality of life (*n* = 4), satisfaction (*n* = 5); some level of physical functioning (*n* = 4); adverse outcomes (e.g., unmet needs or psychological risk) (*n* = 5); and costs data (*n* = 3). Other outcomes of interest included community participation/integration (*n* = 2 studies), selfperceived health, safety (*n* = 1), choice‐making (*n* = 1), challenging behaviour (*n* = 1), and person‐centred planning process. Table 2 provides a summary of study characteristics for all seven included studies.

#### 6.2.1 Participant characteristics

A total of 4,834 adults are included in the narrative synthesis, representing a collective response rate of 73%. Of the five studies that reported average age, those in the intervention and control groups were of a similar age (43 and 42 years old respectively). However, one study (Benjamin et al., 2000) reported that 54% of the intervention group and 50% of the control group were over 65. In another study (Brown et al., 2007), 48% were aged 18–39 years. In the latter, the older age groups (over 64 in 2 sites and over 59 in 1 site) were excluded from the narrative synthesis as there was no way to determine if a life‐long disability was present for the older cohort. All studies reported gender differences and ethnic/racial minority status; overall 61% were female (*n* = 2,963) and 28% were from an ethnic or racial minority group (*n* = 1,372). A mix of disabilities was represented in the sample including physical, cognitive/intellectual, mental health, developmental, and/or multiple/secondary disabilities (Table 2). Breakdown by intervention and control group (where available) can be seen in Appendix 4.

#### 6.2.2 Intervention characteristics

The included studies examined the effectiveness of a number of individualized funding models. These included four ‘consumer‐directed’ services, one ‘self‐determination’ programme, one ‘individual budgets’ programme and one ‘personal budgets’ programme. Six of the seven models permitted the purchase of a wide range of services/supports including, amongst others, payment of workers, home modifications, assistive equipment, and transport. In one of these studies, the services had to involve ‘in‐home’ supports. The remaining seventh study limited purchases to ‘personal assistance services’, although the scope of these services was broad. All the interventions were financed by State funds.

The time period between baseline/commencement of the intervention and follow‐up data collection, ranged from at least 6 months to 9 years. The monthly allocation of cash received by participants was presented differently for each study, with the monthly median payment (for two studies) ranging (between study sites) from £405 to £929 or between $313 and $1,097. Mean monthly payments for three other studies ranged from £1,288 to $1,656. These figures are based on best available data and do not take into consideration differences in, for example, exchange rates. Six of the seven studies involved the collection of data directly from people with disabilities, five of which reported the use of proxy respondents where necessary. Only the study by Caldwell (2007) was based on data collected from the primary caregivers of people with a disability.

### 6.3 Risk of bias in included quantitative studies

**Figure 2:**
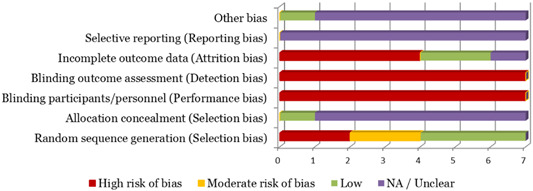
Risk of bias across studies [Color figure can be viewed at wileyonlinelibrary.com]

Only one study within the review was reported as a Randomised Controlled Trial and, as expected, this study was designed to assess the effectiveness of a social intervention. When assessed using the Cochrane risk of bias tool, this study by Glendinning (2008) was rated as ‘low’. As with most social interventions, however, it is often not ethically or practically possible to adhere strictly to the parameters that affect risk of bias. This is reflected in the low score above. As such, the Glendinning study was reassessed using the “Quality Assessment Tool for Observational Cohort and Cross Sectional Studies” (as with the other six studies) and, as a result, the rating increased to ‘good’. Nevertheless, as set out in the protocol, each of the domains used to assess risk of bias is discussed below. Using these criteria, the overall risk of bias across the seven studies was high (Figure 2). The use of the “Quality Assessment Tool for Observational Cohort and Cross Sectional Studies" (NHLBI, 2014) yielded a rating of ‘good’ for three of the included studies, ‘fair’ for three studies and ‘poor’ for one. Appendix 6 provides complete quality and risk of bias tables for each study. *Note* – both assessments are available for Glendinning (2008).

**Figure 3:**
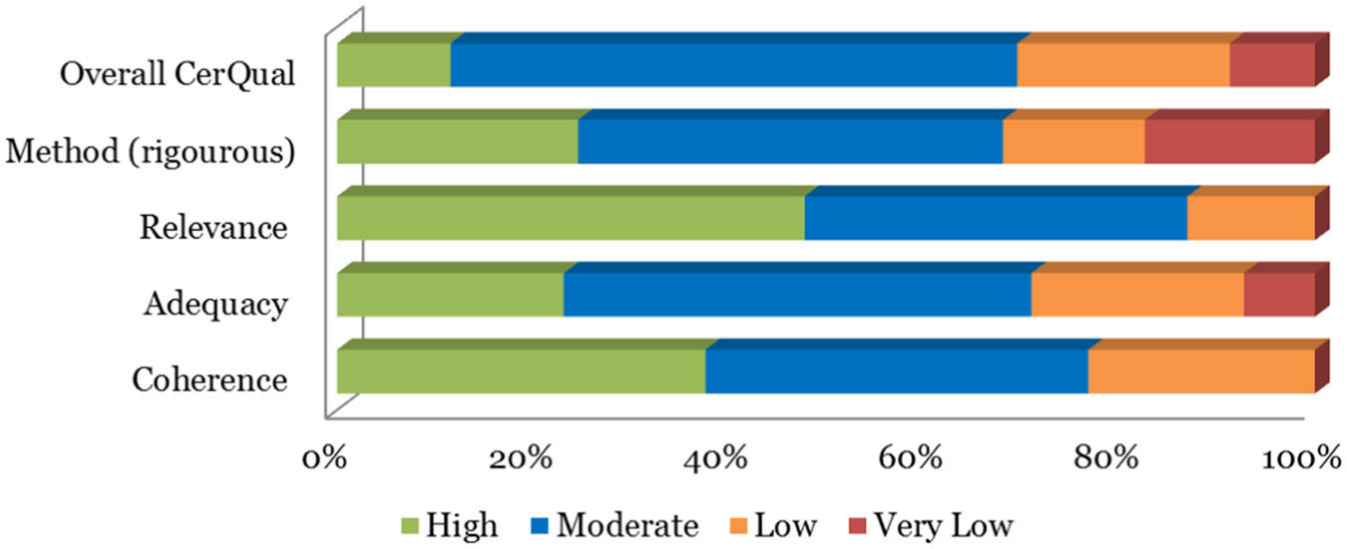
Confidence in individual studies based on CerQual headings [Color figure can be viewed at wileyonlinelibrary.com]

#### 6.3.1 Selection bias

Selection bias is based on random sequence generation and allocation concealment. Two of the 7 studies (Brown et al., 2007; Glendinning et al., 2008) involved random allocation of participants to either the intervention or control group, whilst a third used stratified randomization across study sites for various reasons (e.g., age, level of service need, ethnicity, residential setting or geographic spread) (Conroy, Brown, et al., 2002). In three studies, the sample was selected randomly from a larger pool of potential participants, while participant recruitment in the final remaining study was organized through a gatekeeper (Beatty, Adams, & O'Day, 1998).

Only one study reported efforts to conceal allocation. This is not unusual in social interventions but nevertheless, studies that did not report allocation concealment were considered unclear in terms of risk. Random sampling was considered medium risk, except in one case where people with a severe cognitive disability were excluded from the sampling frame and case manager discretion was reported to have potentially biased intervention/control assignment (Benjamin et al., 2000). This, together with the final study (in which no random allocation or selection was used), were rated as high risk (Figure 2).

#### 6.3.2 Performance and detection bias

As with most social interventions, it is not possible to blind either participants or personnel in terms of the type of intervention received. Likewise, with individualized funding compared to traditional service provision, it was impossible to blind participants or outcome assessors and, therefore, these domains were inherently high risk for all studies. In fact, the inability to blind personnel led to negative feedback about the selection process in two of the three randomized studies, with staff questioning the acceptability of withholding the intervention from interested parties and control participants – pressing their care manager for the intervention immediately rather than in six months’ time (Glendinning et al., 2008), while others suggested that a purposeful sampling process would have been more appropriate (Conroy, Brown, et al., 2002). This kind of problem is commonplace in community‐based trials and especially when the intervention is viewed positively by those delivering it.

#### 6.3.3 Attrition bias

Two of the studies were cross‐sectional and therefore attrition was low (Benjamin et al., 2000; Woolham & Benton, 2013). One of these studies excluded 10% of the original sample due to gatekeepers wrongly identifying participants who did not match the inclusion criteria or, had moved away, been hospitalized or passed away (Woolham & Benton, 2013). Beatty et al. (1998) carried out a longitudinal survey, but it is unclear whether there were multiple data collection points; however, approximately half (48%) of the original control group were excluded from the study as they were not in receipt of any service with which to compare the intervention.

For the remaining four included studies, the risk of bias was considered high as all four had attrition/exclusion levels exceeding 20%. For the Glendinning et al. (2008) study, the total loss at follow‐up was 29%. A total of 129 (10% of 1,356 original sample) were not approached because: they no longer received social services support; had passed away, were not contactable, or had moved away. A total of 221 (16%) did not complete a six‐month follow‐up interview for various reasons including illness and no longer wishing to participate. An additional 47 (3%) were also excluded post‐interview because the randomization group could not be validated. Finally proxy interviews were excluded for certain measures, where self‐completion is intended (e.g., GHQ‐12 and ASCOT) and for single item outcome measures, if a proxy completed the interview on behalf of the individual with a disability or when the proxy assisted that individual in answering the question.

The ‘Cash and Counseling’ pilots involved three study sites and a total eligible intervention group of 1,139 individuals. A significant minority (21 –34%) had withdrawn from the intervention at the 12 month follow‐up. Those who had withdrawn at the 9‐month data collection point were excluded from the analysis. The most common reasons for drop‐out included: a perception that the allowance was too low; that traditional agency services were meeting the needs of the person with a disability; or the individual with a disability had problems with employer responsibilities. Furthermore, where it was not appropriate for proxy respondents to answer questions (on, for example, perceived quality of life), these questions were not asked of proxies. In addition, it should be noted that only 81%, 67% and 68% of the three intervention groups respectively had received an allowance by the 9‐month follow‐up point. However, due to the intent‐to‐treat approach, all responses were reported which may have skewed the findings (Brown et al., 2007). With regard to the 9‐year longitudinal study, a second intake of participants was included in the time 3 data, representing a total sample of 135 families in the intervention. Only 38 were available after 9 years, representing a 72% attrition rate at time three. Available data for the attrition group were reportedly limited, with the authors acknowledging unknown factors that may have biased the longitudinal group. Finally, Conroy et al. (2002) reported an overall 31% attrition rate at follow‐up; furthermore, costs data was only available for 26% of respondents (due to limitations with data access).

#### 6.3.4 Reporting bias

None of the included studies incorporated a study protocol. Therefore, is it unclear whether a priori outcomes were identified, or whether all outcomes of interest under investigation were reported. Therefore, reporting bias was considered unclear for the studies. The lack of study protocol, and general incongruent and complex nature of the included studies, also precluded formal assessment of publication bias. Having said that, it appears that the measured outcomes are in line with the aims, as set out in the study results. However, not all studies reported the outcomes of interest for this review and, therefore, it cannot be determined if, for example, adverse effects data were collected for the four studies that did not report any.

#### 6.3.5 Other biases

None of the studies reported any conflicts of interest. In terms of funding, two did not receive any funding (Conroy, Brown, et al., 2002; Woolham & Benton, 2013), three were government funded (Beatty et al., 1998; Benjamin et al., 2000; Glendinning et al., 2008) and two were a combination of government funding and other funding sources including: the Robert Wood Johnson Foundation (Brown et al., 2007); and the National Institute on Disability and Rehabilitation research (Caldwell et al., 2007).

Authors of the ‘IBSEN’ study (Glendinning et al., 2008) acknowledge two potential sources of bias. Despite the randomized design, the population from which the sample was drawn, was potentially biased. For instance, 26% of the intervention group (those with an individual budget) had previously been in receipt of a ‘Direct Payment’ (similar intervention). However, only 4% nationally were using a Direct Payment. Therefore, people with previous experience of a ‘Direct Payment’ were over represented in the study intervention, when compared to the national average. The authors felt that 26% of the intervention group may, therefore, have provided more positive responses due to previous experience with direct payments. Moreover, this over representation may have resulted in smaller differences in terms of costs and outcomes than may have been observed in a more representative sample, since comparisons were not being made with traditional services, but rather another form of individualized funding. As a result, the authors factored previous experience of a direct payment into their analysis and did not find any effect on the results for either of the aforementioned concerns (Glendinning et al., 2008, pp. 44–45 & 80).

### 6.4 Synthesis of quantitative results

Each outcome will be discussed in detail in relation to primary, secondary, adverse and other outcomes (see Sections 6.4.2–6.4.9). Table 4 summarizes the outcomes of interest for all seven included studies, providing an overview of key significant differences and the direction of these effects (i.e., favouring intervention or control). Individualized funding was seen to statistically favour the intervention group with regard to quality of life (2 studies) client satisfaction (5 studies), adverse outcomes (2 studies) and sense of security (1 study). Cost‐effectiveness results (2 studies) were more favourable for the control group, while one measure of unmet need (out of three) also favoured the control group (1 study).

#### 6.4.1 Primary outcomes of interest

This section provides a narrative synthesis of the primary outcomes of interest, as per protocol, including Quality of Life and Client Satisfaction. Intervention group (I) and control group (C) data are presented for each of the outcomes of interest. Data are presented in line with the original studies unless further statistical tests were required to measure significant differences. Such tests are reported where applicable. Data are presented for eligible participants only (adults with lifelong disability). All analysis that was conducted in RevMan and WinPepi are presented in the Data and Analysis section (Section 9.1)

#### 6.4.2 Quality of life

Four studies reported ‘quality of life’ and/or ‘psychological well‐being’. A meta‐analysis could not be conducted due to incompatibility of study design, insufficient data and randomization differences. Where necessary, data were extrapolated to test significant differences between the intervention and control groups and mean differences were calculated using RevMan tTest calculator. A description of quality of life measures for each study can be seen in Appendix 9 (Table A9.1). (Brown et al., 2007)

Data were available for 1,822 (93%) of the eligible sample (working‐age adults). Means were calculated using a logit model (i.e., a logistical regression model where the dependent variable is categorical). Satisfaction levels, based on the reported findings (i.e., those very satisfied with way spending life), were significantly higher amongst participants from the intervention group when compared to the control group across the three study sites: Site 1 (I: 43.4 / C: 22.9, MD = 20.5, (*p* < .001)); Site 2 (I: 63.5 / C: 50.2, MD = 13.3, (*p* < .01)); and Site 3 (I: 37.5 / C: 21.0, MD = 16.5, (*p* < .001)). Combined data for the three sites were not reported, nor were standard deviations. Furthermore, it should be noted that not all recipients received an allowance, but an ‘intent‐to‐treat’ approach was utilized regardless. (Conroy, Brown, et al., 2002)

Before and after mean scores were presented for the ‘Quality of Life changes’, but no standard deviations were reported. Significant differences were reported, overall, for both the intervention and the control group indicating that quality of life had improved for everyone participating in the study. There were three separate intervention groups, one of which was recruited from the same geographic location as the control group. When all three intervention groups (combined) were compared to the control group, the change is more marked for the intervention group, with the quality of life score increasing by 12.1 points (moving from 69.2 before to 81.3 after). A similar but smaller change in the control group (mean difference (MD) = 8.4) was also observed (69.6–78.0). The figures for the single geographically similar intervention group also show a comparable pattern moving from a score of 66.7 to 78.0 (MD = 11.1). Significant differences between intervention and control groups are not reported and could not be calculated due to insufficient data. (Glendinning et al., 2008)

Quality of life responses were provided for 504 (99%) of the intervention group and 439 (98%) of the control group at six‐month follow‐up. Data were presented by disability type. Only one group, mental health service users (I: *n* = 65 / C: *n* = 64), reported a significant difference – in favour of the intervention group ‐ (I: 3.78 vs. C: 4.31, MD = −0.53, *p* < .05). *Note* : Higher GHQ scores indicated poorer outcomes.

Since data were only presented for one subgroup, means could not be calculated for the entire sample as no standard deviations were presented. Therefore, the proportion of those responding positively (at follow‐up) on the 7 point scale (227 (I), 215 (C)) were compared to those who were ambivalent or negative for both controls and interventions, with no significant differences detected using Upton's Chi square (*p* = .28). When proxies were excluded, the sample was reduced to 308 intervention respondents and 302 controls. Once again, there was no significant difference between the two groups (*p* = .77) (Table 6).

**Table 6:** Results from WinPepi – Glendinning et al. (2008)

Study	Outcome	Study arm	Sample size	Proportion (positive/yes)	Upton chi square	*p*‐value
Quality of life		Intervention	504	0.45	1.16	.28
		Control	439	0.49		
Quality of life (excluding proxies)		Intervention	308	0.41	0.08	.77
		Control	302	0.42		
Client satisfaction		Intervention	478	0.78	7.54	< .01
		Control	431	0.70		
Client satisfaction (excluding proxies)		Intervention	268	0.78	4.22	<.05
		Control	288	0.70		
Psychological ill‐health		Intervention	448	0.36^a^	0.84	.36
		Control	380	0.33^a^		
Psychological ill‐health (excluding proxies)		Intervention	344	0.37^a^	0.00	.98
		Control	300	0.37^a^		
Self‐perceived health		Intervention	507	0.35	2.20	.14
		Control	446	0.40		
Self‐perceived health (excluding proxies)		Intervention	311	0.33	0.03	.87
		Control	317	0.34		

^a^ Higher scores indicate worse ill‐health

#### 6.4.3 Quality of life – psychological well‐being

Relevant data on psychological well‐being are presented for two studies below. Although the GHQ was used in both, a meta‐analysis could not be conducted as Glendinning et al. (2008) randomly assigned participants to either the intervention or control group, while Woolham and Benton (2013) randomly selected their sample from within the relevant populations (i.e., those in receipt of individualized funding and those receiving traditional supports). (Glendinning et al., 2008)

The total number of respondents on the GHQ‐12 included 448 (88%) intervention group and 380 (85%) control group participants. A higher score on the GHQ‐12 indicates worse overall well‐being. There were no significant differences observed when comparing intervention and control groups (I: M = 13.83, *SD* = 6.74 / C: 13.80, *SD* = 6.85, MD = 0.03, (*p* = .95), 95% CI [−0.899, 0.959]) (Figure 8). (Woolham & Benton, 2013)

**Figure 4:**
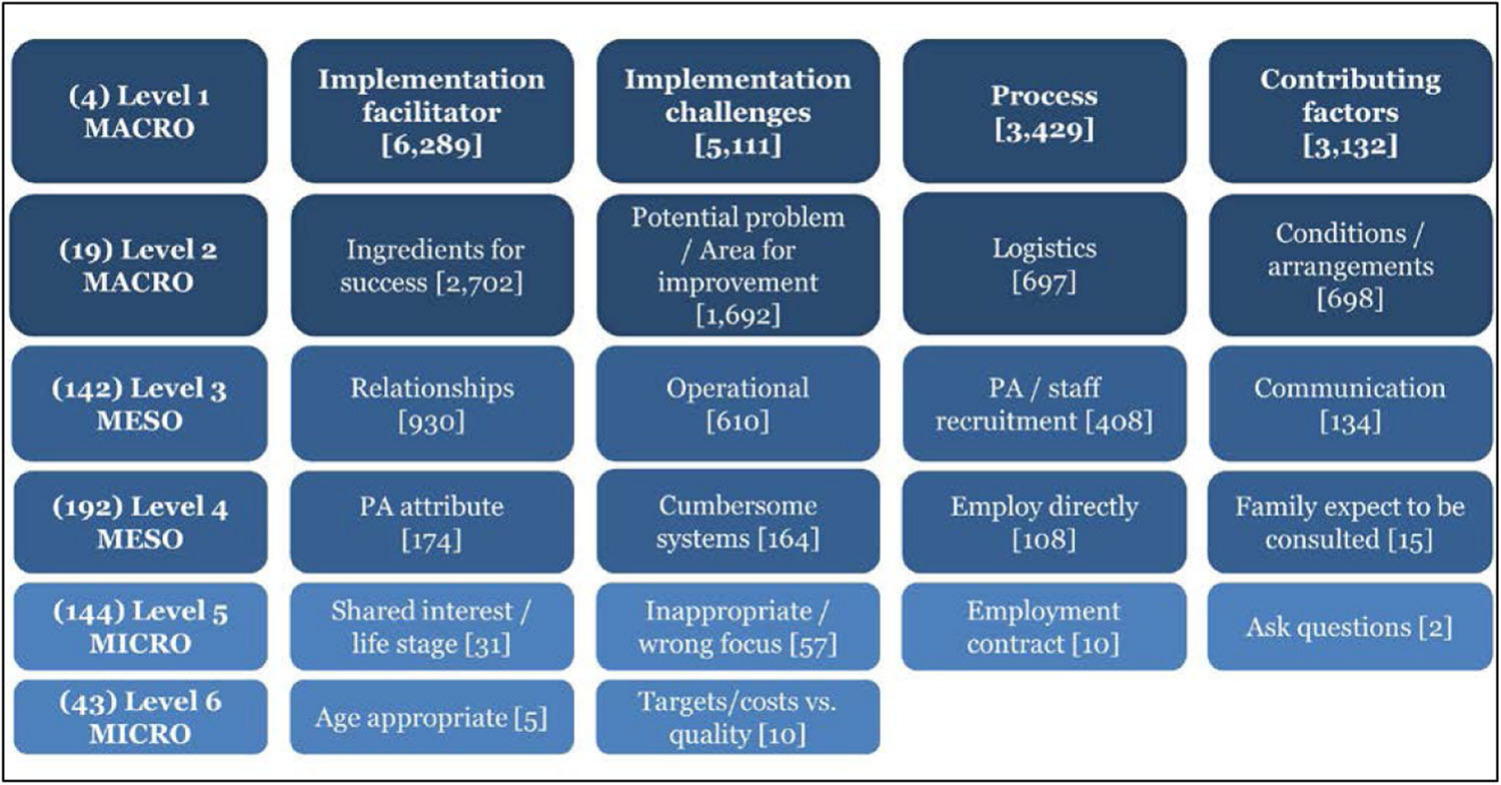
Example of coding levels 1 to 6 (Macro, Meso, Micro) [Color figure can be viewed at wileyonlinelibrary.com]

**Figure 5:**
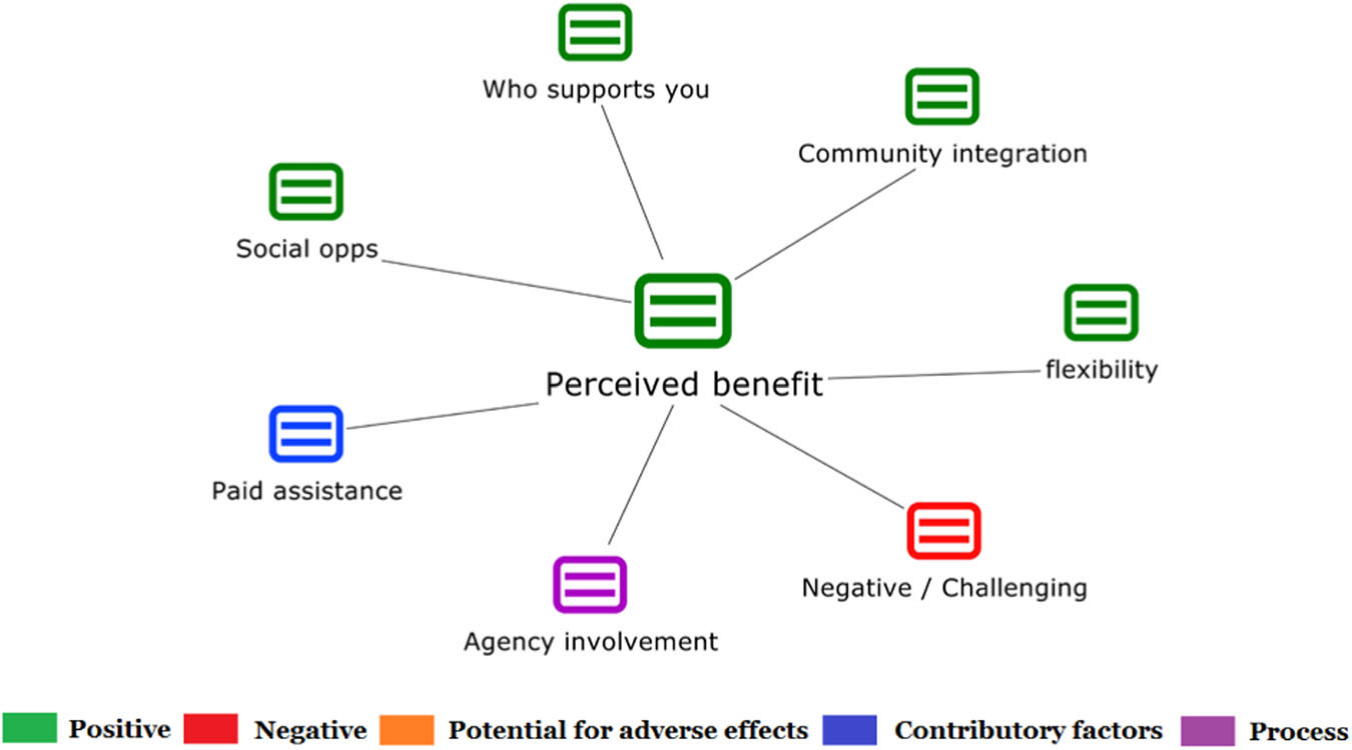
Codes co‐occurring with ‘perceived benefits’ 60 times of more [Color figure can be viewed at wileyonlinelibrary.com]

**Figure 6:**
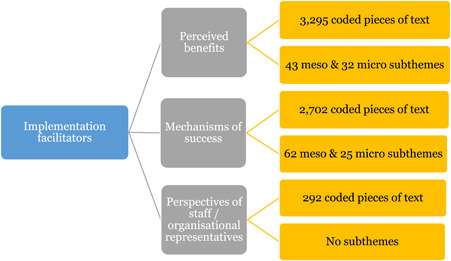
Coding structure of ‘Implementation Facilitators’ [Color figure can be viewed at wileyonlinelibrary.com]

**Figure 7:**
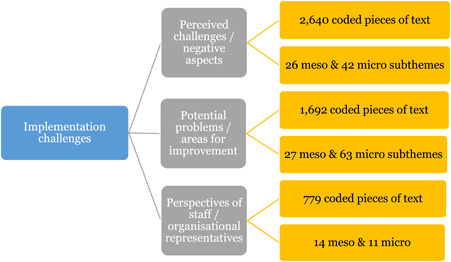
Coding structure of “Implementation challenges” [Color figure can be viewed at wileyonlinelibrary.com]

**Figure 8:**
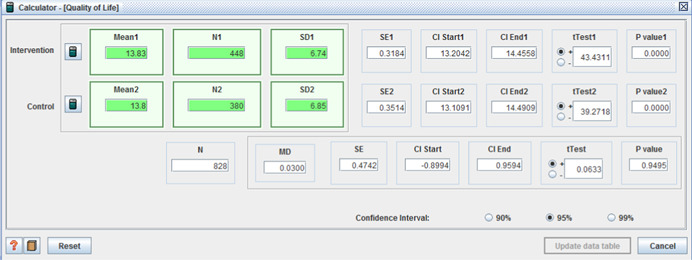
Quality of Life –Psychological Wellbeing – Glendinning et al. (2008) [Color figure can be viewed at wileyonlinelibrary.com]

**Figure 9:**
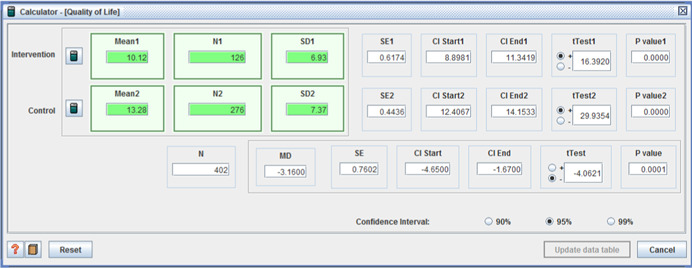
Quality of Life –Psychological Wellbeing – Woolham & Benton (2013) [Color figure can be viewed at wileyonlinelibrary.com]

Relevant data were presented separately for the older versus younger service users, but older participants were excluded from this review since there was no way to confirm a ‘life‐long disability’. This led to a reduction in the total sample to 126 (70%) in the intervention and 276 (71%) in the control groups. GHQ scores, for eligible adults, indicated that the intervention group had significantly better psychological well‐being when compared to the control group (I: M = 10.12, *SD* = 6.93 / C: M = 13.28, *SD* = 7.37, MD = −3.16, (*p* < 0.001), 95% CI [−4.65, −1.67]) (Figure 9).

#### 6.4.4 Client satisfaction

Five studies reported ‘Client satisfaction’. Three of these were nonrandomized, but all used different measures of client satisfaction. Data are presented as reported (where means and standard deviations were available), or extrapolated to test for significant differences between categorical data using Upton's *χ*² square (as recommended by Campbell (2007). A description of the client satisfaction measures for each study can be seen in Appendix 9 (Table A9.2). (Beatty et al., 1998)

**Figure 10:**
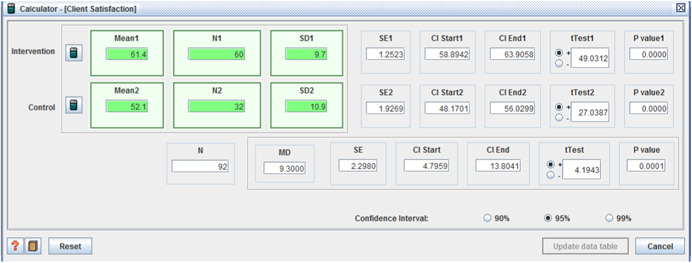
Client Satisfaction – Beatty et al. (1998) [Color figure can be viewed at wileyonlinelibrary.com]

The full eligible sample of 60 intervention respondents and 32 individuals from the control group took part in the study. An overall satisfaction score was calculated based on 16 responses to the ‘Personal Assistance Satisfaction Index’ (ranging from 16 to 80). Responses were then collapsed into two categories representing: (a) those who were ‘very satisfied’ or ‘extremely satisfied’, and (b) those who were ‘not at all satisfied’, ‘slightly satisfied’, or ‘somewhat satisfied’. The higher the score, the higher the overall levels of satisfaction. Intervention and control groups were compared within the positive category, with the intervention group reporting significantly higher scores (I: 61.4, *SD* = 9.7 / C: 52.1, *SD* = 10.9, MD = 9.3, (*p* < .001), 95% CI [4.80, 13.80]) (Figure 10). (Benjamin et al., 2000)

**Figure 11:**
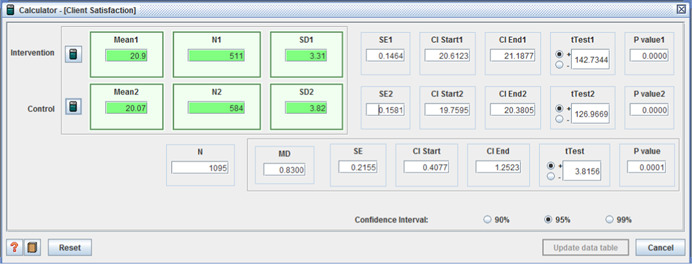
Client satisfaction (Technical Quality) – Benjamin et al. (2000) [Color figure can be viewed at wileyonlinelibrary.com]

**Figure 12:**
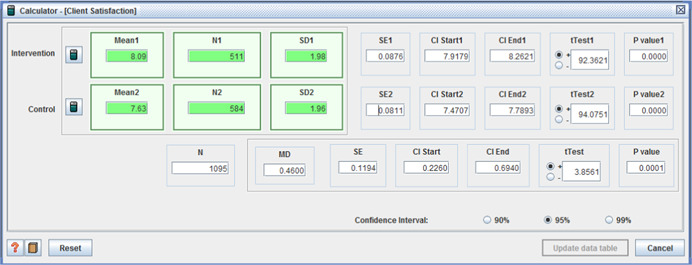
Client satisfaction (Service Impact) – Benjamin et al. (2000) [Color figure can be viewed at wileyonlinelibrary.com]

**Figure 13:**
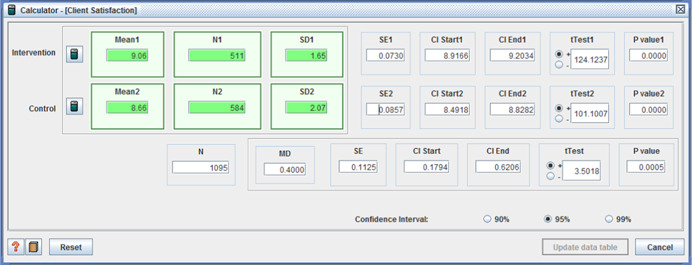
Client satisfaction (General Satisfaction) – Benjamin et al. (2000) [Color figure can be viewed at wileyonlinelibrary.com]

**Figure 14:**
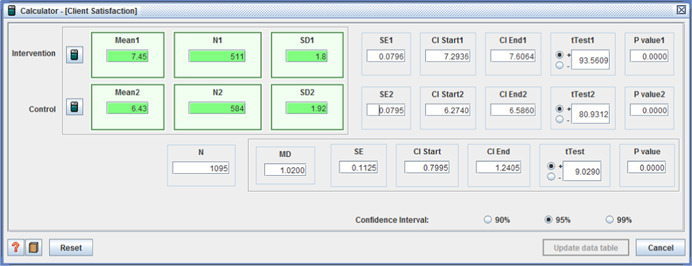
Client satisfaction (Interpersonal Manner) – Benjamin (2000) [Color figure can be viewed at wileyonlinelibrary.com]

A total of 511 intervention and 584 control group participants were involved in this study. Five items of client satisfaction were measured and reported separately. The higher the reported score, the greater the levels of satisfaction experienced. The intervention group reported significantly higher satisfaction scores on four of the five items (Figures 11, 12, 13, 14) including:

- ‘technical quality’ (I: 20.90, *SD* = 3.31 / C: 20.07, *SD* = 3.82, MD = 0.83, (*p* < .001), 95% CI [0.41, 1.25];
- ‘service impact’ (I: 8.09, *SD* = 1.98 / C: 7.63, *SD* = 1.96, MD = 0.46, (*p* < .001), 95% CI [0.23, 0.69]);
- ‘general satisfaction’ (I: 9.06, *SD* = 1.65 / C: 8.66, *SD* = 2.07, MD = 0.40, (*p* < .001), 95% CI [0.18, 0.62]); and
- ‘interpersonal manner’ (I: 7.45, *SD* = 1.80 / C: 6.43, *SD* = 1.92, MD = 1.02, (*p* < .001), 95% CI [0.80, 1.24]).

**Figure 15:**
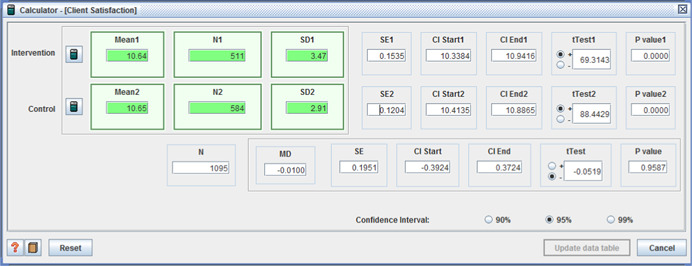
Client satisfaction (Provider shortcomings) ‐ Benjamin et al. (2000) [Color figure can be viewed at wileyonlinelibrary.com]

There was no significant difference between intervention and controls for ‘provider shortcomings’ (I: 10.64, *SD* = 3.47 / C: 10.65, *SD* = 2.91, MD = −0.01, (*p* = .96), 95% CI [−0.39, 0.37]) (Figure 15). (Brown et al., 2007)

In order to compare mean differences, client satisfaction data were collapsed into two categories ‐ the way the caregiver helped around house/community and overall care arrangement. Means were predicted using logit models. Data were available for 1,822 (93%) of the eligible sample across three sites. With regard to the first category above, significantly more intervention group members reported being very satisfied across all sites: Site 1 (I: 90.4 / C: 64.0, MD = 26.4, (*p* < .001)); Site 2 (I: 85.4 / C: 70.9, MD = 14.5, (*p* < .01)); and Site 3 (I: 84.4 / C: 66.0, MD = 18.4, (*p* < .001)). In relation to overall care arrangements, higher mean scores were also seen across the intervention groups: Site 1 (I: 71.0 / C: 41.9, MD = 29.2, (*p* < .001)); Site 2 (I: 68.2 / C: 48.0, MD = 20.2, (p < .01)); and Site 3 (I: 51.9 / C: 35.0, MD = 16.9, (*p* < .001)). There were insufficient data to combine data from the three sites. Furthermore, it should be noted that not all recipients received an allowance, but an ‘intent‐to‐treat’ approach was utilized regardless. (Caldwell et al., 2007)

**Figure 16:**
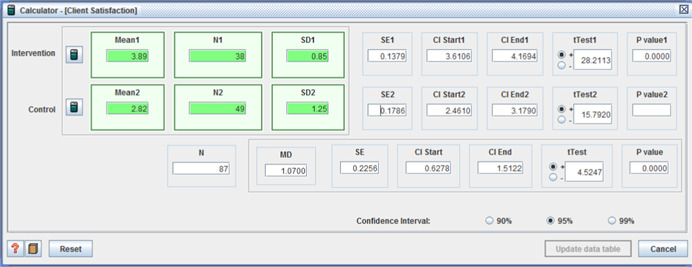
Client Satisfaction – Caldwell et al. (2007) [Color figure can be viewed at wileyonlinelibrary.com]

Time 3 data are presented for both the intervention group (*n* = 38) and control group (*n* = 49). Means and standard deviations were reported. At time 3, the intervention group was significantly more satisfied with the service than the control group (I: M = 3.89, *SD* = 0.85 / C: M = 2.82, *SD* = 1.25, MD = 1.07, (*p* < .001), 95% CI [.63, 1.51]) (Figure 16). (Glendinning et al., 2008)

Categorical data were presented for levels of client satisfaction ranging from ‘extremely satisfied’ to ‘extremely dissatisfied’. A total of 478 (94%) intervention group and 431 (96%) controls reported satisfaction data. The proportion of those responding positively on the 7 point scale (378 (I), 306 (C)) was significantly greater in the intervention group than in the control group when compared to those who were ambivalent or negative in both groups (*p* < .01 using Uptons Chi square). When proxies were excluded, the sample was reduced to 268 intervention respondents and 288 controls. Once again, significantly more of those in the intervention group were satisfied when compared to their control group counterparts (*p* < .05) (Table 6).

#### 6.4.5 Secondary outcomes of interest

This section provides a narrative synthesis of the secondary outcomes of interest (as per protocol), including Physical Functioning and Costs Data. Intervention group (I) and control group (C) data are presented for each of the outcomes alongside results from statistical tests of difference. Upton chi square was used to compare proportions from two independent samples whilst RevMan was used to conduct *t*‐tests. Data are presented for eligible participants only (working‐age adults).

#### 6.4.6 Physical functioning

Four studies collected data related to physical functioning, but only one reported such data in terms of measuring differences between intervention and control groups. The remaining studies used the data as coefficients for further analysis. A description of physical functioning measures for each study can be seen in Appendix 9 (Table A9.3). (Woolham & Benton, 2013)

**Figure 17:**
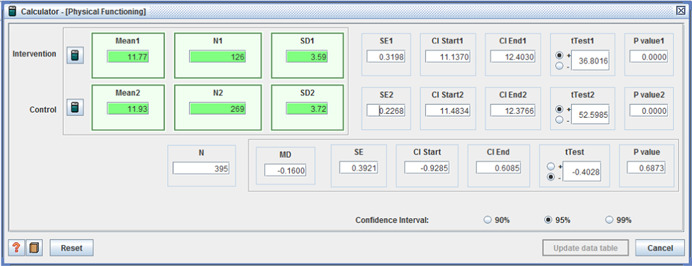
Physical Functioning – Woolham & Benton (2013) [Color figure can be viewed at wileyonlinelibrary.com]

Activities of Daily Living (ADL) data were presented separately for the older versus younger service users; the former were excluded from this review since there was no way to confirm a life‐long disability. The resulting sample comprised 126 (70%) in the intervention group and 269 (71%) in the control group. There was no significant difference between the two groups in terms of physical functioning (I: M = 11.77, *SD* = 3.59, C: M = 11.93, *SD* = 3.72, MD = −0.16, (*p* = .69), 95% CI [−0.93, 0.61]) (Figure 17).

#### 6.4.7 Costs data

While most studies present costs data in some form, only three did so for both the intervention and control group. Two of these studies report cost‐effectiveness analysis, one of which involved a randomized trial (Glendinning et al., 2008). Furthermore, the authors of the more recent study caution against any direct comparisons with the former due to methodological differences. For this reason, all studies are reported separately. Data are reported as seen in the original papers, with the exception of Woolham, where non‐eligible adult respondents are excluded for part of the narrative results. A description of costs data measures for each study can be seen in Appendix 9 (Table A9.4). (Brown et al., 2007)

Within the ‘Cash and Counselling’ study, the effect on Medicaid and Medicare expenditures was compared between intervention and control groups. The overall sample of working‐age respondents comprised 2,109 participants (92% of the baseline sample) across three study sites. The average monthly cost for eligible intervention group members was $1,183 compared to $1,040 for control group individuals. However, the costs varied considerably across the three sites, ranging from a monthly average of $513 in Arkansas to $1,884 in Florida (intervention) and from $422 to $1,593 for controls (respectively). The average monthly cost was significantly higher for the intervention group across all three sites (*p* < .01 for Arkansas and Florida, *p* < .05 for New Jersey).

Intervention‐control group differences were used to measure the effect of Medicaid costs overall. This was also divided into ‘Personal Care/Home & Community Based Services (HCBS)’ and ‘Other Medicaid costs’. With regard to the overall Medicaid costs, there were no significant differences observed for mean differences in two study sites (Arkansas I: M = 14,125 / C: M = 12,862, MD = 1,263, (*p* = .14), New Jersey: I: M = 26,863 / C: M = 26,049, MD = 814, (*p* = .59)), whilst a significant increase among the intervention group was observed in the Florida site (I: M = 27,433 / C: M = 24,106, MD = 3,327, (*p* < .001). When examining Personal Care/HCBS alone, there was a significant increase for the intervention group across all three sites (Arkansas I: M = 5,435 / C: M = 2,430, MD = 3,005, (*p* < .001), Florida I: M = 22,017 / C: M = 18,321, MD = 3,696, (*p* < .001), New Jersey I: M = 11,166, C: M = 9,220, MD = 1,946, (*p* < .001)) (Brown et al., 2007, Table V.1; Dale & Brown, 2005). Combined data for the three sites were not reported, nor were standard deviations. (Glendinning et al., 2008)

Within the IBSEN study, cost‐effectiveness was analyzed by using the mean difference in outcomes of interest (e.g., the GHQ‐12), and dividing it by the mean difference in costs. This allowed ‘incremental cost‐effectiveness ratios’ (ICERs) to be examined for each outcome of interest. Prior to doing this however, costs were compared descriptively across three domains including: (a) social care costs; (b) health care costs; and (c) costs of care and support planning and management.

Data for social care costs were available for 268 (53%) of the intervention group and 250 (56%) of the controls. An average weekly cost of £279 and £296 was reported for each group respectively with no significant between‐group differences. The mean weekly health care costs for the intervention group were significantly higher than the control group (£83 vs. £59; *p* < .05). It should be noted however, that the potentially non‐eligible ‘older population’ had the highest mean cost (£107 per week) compared to people with a physical disability (£76), learning disability (£23) or mental health problem (£76). With respect to care management, the intervention group had significantly higher costs (£217 vs £128 mean cost, *p* < .001) which was most probably due to the significantly higher mean number of visits (I: 1.66, C: 0.98, *p* < .001).

In terms of cost‐effectiveness, Incremental Cost‐Effectiveness Ratios (ICERs) were presented with bootstrapped estimates of standard error (se). ICERs were examined using ASCOT and GHQ scores, and while trends indicated a positive direction for the intervention group, these were not statistically significant. Notably, a sub‐group analysis (using scatterplots) showed that the potential for cost‐effectiveness is strongest with people with mental health problems as reflected in responses on both the ASCOT and GHQ‐12. (Woolham & Benton, 2013)

Data were presented for the entire intervention group (*n* = 177) and 72% of the control group (*n* = 271). The total number per group fell to 124 (72%) and 191 (51%) in the intervention and control groups respectively after non‐eligible older people were removed. The mean weekly package costs for the (eligible) intervention and control groups were £355 and £268 respectively. Standard deviations are not presented and therefore statistical testing was limited, although it is clear that packages are more costly for the intervention group.

Similar to Glendinning et al. (2008), bootstrapping was used to draw comparisons based on outcomes of interest, in this case the ADL and GHQ measures. Although exact figures are not presented, scatterplots reveal some intervention versus control group differences. It should be noted that overall cost‐benefit analysis represents the whole sample, including older adults. When comparing ADL scores, there is little difference between the two groups (both relatively independent), but based on this outcome, the package costs are higher for the intervention group. The scatterplots for GHQ scores show that the control group was experiencing ‘some degree of ill‐being’. While the intervention group were experiencing better well‐being, the costs were again higher on average. Woolham and Benton's comparison of working‐age and older intervention individuals, showed that the former cohort had better outcomes (well‐being and independence levels), but the costs were also higher for the working‐age adults. The authors suggest that findings should be treated with caution since the one of the measures used to inform the cost‐benefit analysis (ADL) did not report statistical differences between intervention and control groups (see 6.4.6).

#### 6.4.8 Adverse outcomes

Adverse outcomes are reported in some form, in five of the seven included studies, although there was considerable variation in the outcomes measured. The only commonality was seen in the two nonrandomized studies (Benjamin et al., 2000; Caldwell et al., 2007), which both measured unmet needs, albeit using different tools. Data are narratively presented as in the case of original studies, with further analysis reported as necessary. A description of adverse outcomes measures for each study can be seen in Appendix 9 (Table A9.5). (Benjamin et al., 2000)

**Figure 18:**
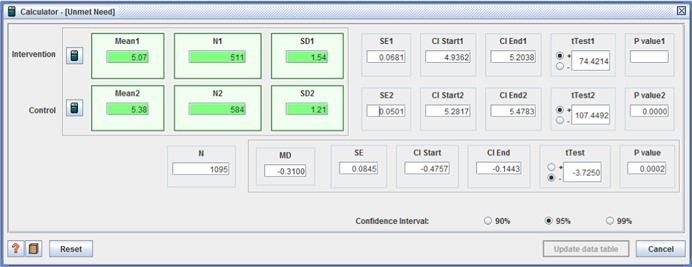
Unmet need – ADL – Benjamin et al. (2000) [Color figure can be viewed at wileyonlinelibrary.com]

**Figure 19:**
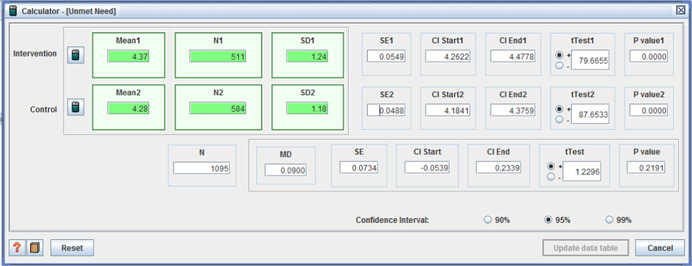
Unmet need – IADL – Benjamin et al. (2000) [Color figure can be viewed at wileyonlinelibrary.com]

**Figure 20:**
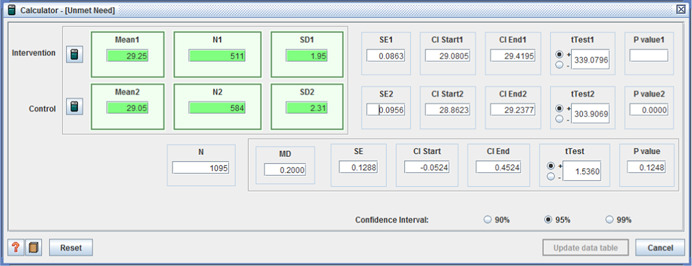
Unmet need – Physical or psychological risk – Benjamin et al. (2000) [Color figure can be viewed at wileyonlinelibrary.com]

There are two adverse outcomes reported within this study. The first, ‘unmet need’, is broken down into two further domains i.e., ADL and Incremental ADL. The second main adverse outcome reported is physical and psychological risk. Both outcomes are presented for the intervention (*n* = 511) and control group (*n* = 584). With regard to ADL, the control group reported significantly fewer needs (I: M = 5.07, *SD* = 1.54, C: M = 5.38, *SD* = 1.21, MD = −0.31, (*p* < .001), 95% CI [−0.48, −0.14]). There were no significant differences detected on IADL (I: M = 4.37, *SD* = 1.24, C: M = 4.28, *SD* = 1.18, MD = 0.09, (*p* = .22), 95% CI [−0.05, 0.23]). Similarly, there were no significant differences detected for physical or psychological risk: (I: M = 29.25, *SD* = 1.95 / C: 29.05, *SD* = 2.31, MD = 0.20, (*p* = .13), 95% CI [−0.05, 0.45]). (Figures 18, 19, 20) (Brown et al., 2007)

Data for eligible participants (working‐age adults) from the Cash and Counselling study are presented below. Data were available for 1,822 (93%) of the eligible sample on the first two adverse outcomes below. A further four care‐related health problems/events were reported for 1,938 (99%) of the working‐age sample.

1. Based on the reported findings, significantly fewer intervention group members had unmet needs with regard to helping with daily living activities across the three study sites: Site 1 (I: 25.8 / C: 41.0, MD = −15.2, (*p* < .01)); Site 2 (I: 26.7 / C: 33.8, MD = −7.1, (*p* < .05)); and Site 3 (I: 46.1 / C: 54.5, MD = −8.4, (*p* < .05)).
2. The second adverse outcome measured, related to rudeness or disrespect on the part of the caregiver. Fewer people in the intervention group reported such adverse outcomes across the three sites, although these differences were only statistically significant in two of the three sites: Site 1 (I: 10.5 / C: 29.5, MD = −18.9, (*p* < .01)); and Site 3 (I: 18.7 / C: 30.1, MD = −11.4, (*p* < .01)).
3. There was no significant difference in those reporting having had a fall in two of the three sites. However in the third site, significantly fewer individuals from the intervention group had experienced a fall: Site 3 (I: 18.7 / C: 28.0, MD = −9.3, (*p* < .01)).
4. Once again, only one of the three sites witnessed a significant difference between intervention and control members who reported contractures developing/worsening, with significantly more of the control group reporting such developments: Site 2 (I: 9.0 / C: 14.0, MD = −5.0, (*p* < .05).
5. For those reporting bedsores developing/worsening, only one site reported significant differences, with controls reporting such developments more often than the intervention group: Site 1 (I: 5.9 / C: 12.6, MD = −6.7, (*p* < .05)).
6. Finally, significantly more control group members reported having had a urinary tract infection in one of the three sites: Site 2 (I: 7.7 / C: 11.7, MD = −4.0, (*p* < .05)).

Combined data for the three sites were not reported, nor were standard deviations. Furthermore, it should be noted that not all recipients received an allowance, but an ‘intent‐to‐treat’ approach was utilized regardless. (Caldwell et al., 2007)

**Figure 21:**
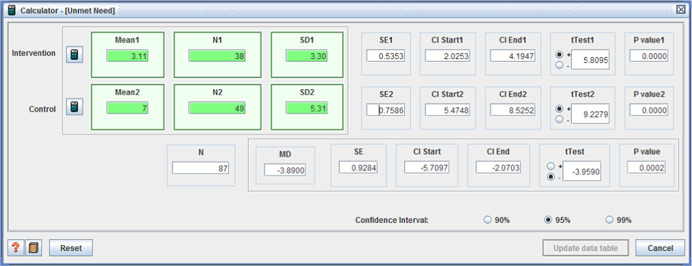
Unmet need – Caldwell et al. (2007) [Color figure can be viewed at wileyonlinelibrary.com]

Unmet needs were compared for intervention group at time 3 (*n* = 38) and the control group (*n* = 49). Significantly fewer people from the intervention group had unmet needs at time 3 compared to the control group (I: M = 3.11, *SD* = 3.30 / C: M = 7, *SD* = 5.31, MD = −3.89, (*p* < .001), 95% CI [−5.71, −2.07]) (Figure 21). (Conroy, Brown, et al., 2002)

Challenging behaviour was compared between people in the intervention and control groups, providing before and after data. Since this is a scale, containing various maladaptive behaviours, means appear to be presented but no standard deviations are reported. No significant differences were reported. As with other outcomes of interest reported in this study, there were three intervention sites and only one control site. The control site was geographically similar to one of the intervention sites. However, the overall findings changed following a comparison of the mean difference for all intervention sites versus the geographically similar site; the score in the combined intervention groups increased from 86.3 at baseline to 88.2 at follow‐up (MD = 1.9), while the control group scores also increased from 84.2 to 89.6 (MD = 5.4), both changes indicating an improvement in challenging behaviour. (Glendinning et al., 2008)

Within the IBSEN study, the GHQ‐12 was used to indicate a risk of ‘psychological ill‐health’. The bimodal (0–1) GHQ scoring method was used to indicate the likely presence of psychological distress according to a designated cut‐off score of 4 or more (Glendinning et al., 2008). A total of 448 (88%) of the intervention group and 380 (85%) controls responded to this item. For the overall sample, 36% (*n* = 161) of the Intervention group obtained a score of 4 or more whilst the same was true for 33% (*n* = 125) of the control group. The differences between intervention and control were not statistically significant using Upton Chi Square (*p* = .36). This did not change when proxy respondents were excluded (Table 6). Furthermore, there were no significant differences by user group.

#### 6.4.9 Other health and social care outcomes of interest

Upon review of the evidence, it became apparent that there were other health and social care outcomes reported that were not categorized exactly as anticipated within the review protocol, but which were still considered very relevant. These were evident in three of the seven studies (see below). The RevMan *t*Test for testing significant differences between outcome means were not reported. A description of other outcomes measures for each study can be seen in Appendix 9 (Table A9.6). (Benjamin et al., 2000)

**Figure 22:**
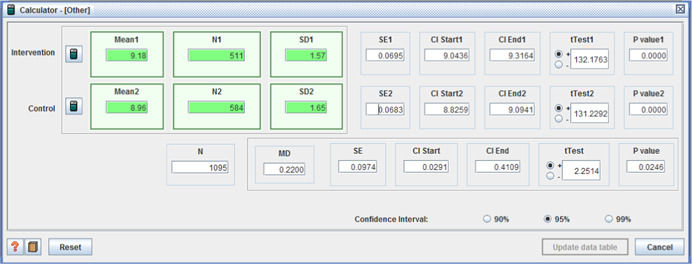
Other – Sense of security – Benjamin et al. (2000) [Color figure can be viewed at wileyonlinelibrary.com]

Sense of security was an outcome reported (for both the intervention (*n* = 511) and control groups (*n* = 584)) under ‘safety’ along with physical and psychological risk (previously reported under adverse outcomes). Significantly more people in the intervention group felt safe with the provider and felt they got along with the provider when compared to the control group (I: M = 9.18, *SD* = 1.57, C: 8.96, *SD* = 1.65, MD = 0.22, (*p* < .05), 95% CI [0.03, 0.41]) (Figure 22). (Caldwell et al., 2007)

**Figure 23:**
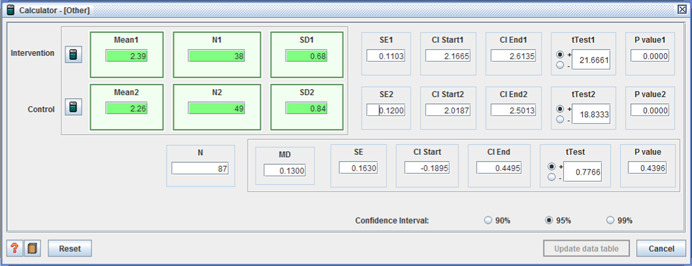
Other – Community Participation – I vs C – Caldwell et al. (2007) [Color figure can be viewed at wileyonlinelibrary.com]

**Figure 24:**
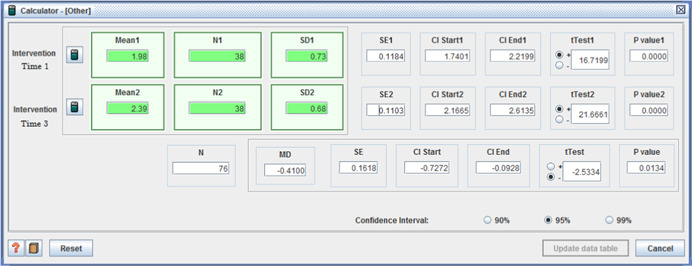
Other – Community Participation – T1 to T3 – Caldwell et al. (2007) [Color figure can be viewed at wileyonlinelibrary.com]

Community participation was measured at time three for both the intervention (*n* = 38) and control group (*n* = 49). There was no significant difference reported between the two groups in this respect (I: M = 2.39, *SD* = 0.68 / C: M = 2.26, *SD* = 0.84, MD = 0.13, (*p* = .439), 95% CI [−0.19, 0.45]) (Figure 23). Interestingly, over the three study periods, community participation increased significantly for the intervention group, but similar data could not be presented for the control group since data was were collected at time 3 for this group (I‐T1: M = 1.98, *SD* = 0.73 / I‐T3: M = 2.39, *SD* = 0.68, MD = −0.41, (*p* < .05), 95% CI [−0.73, −0.09]) (Figure 24). (Glendinning et al., 2008)

Two additional outcomes of interest were reported in the IBSEN study, including changes in self‐perceived health and in the ASCOT scores.

With regard to self‐perceived health, 507 (99%) intervention group members responded along with 446 (99%) controls. There was no significant intervention‐control group difference (*p* = .138) when Upton's Chi square was used to compare proportions of those who responded positively (I: n = 177, C: *n* = 178) with those who responded with neutral or negative responses. This finding was similar when proxy responses were excluded (I: *n* = 103, C: n = 108, *p* = .87) (Table 6). Subgroup analysis conducted by the authors did not demonstrate any significant differences within or between groups.

**Figure 25:**
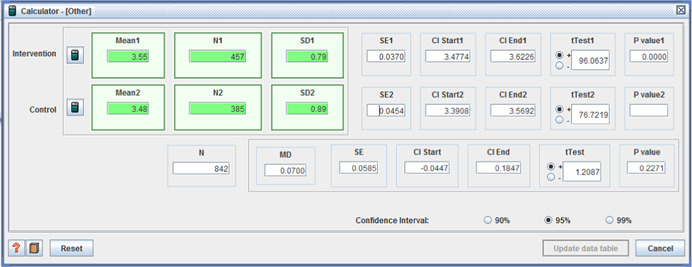
Other – ASCOT – Glendinning et al. (2008) [Color figure can be viewed at wileyonlinelibrary.com]

When examining the ASCOT scores, 90% of intervention group members (*n* = 457) and 86% of controls (*n* = 385) responded. A comparison of mean scores showed no significant between‐group difference (I: M = 3.55, *SD* = 0.79 / C: M = 3.48, *SD* = 0.89, MD = 0.07, (*p* = .227), 95% CI [−0.045, 0.185]) (Figure 25) nor did a subgroup analysis conducted by the authors.

### 6.5 Description of included qualitative studies

As outlined earlier, 69 unique studies (representing 96 titles) were included in the review. Twenty‐eight of these studies were published at least once, while the remaining 41 were sourced from grey‐literature, most of which were published online as a government, research organization or NGO report. The vast majority of studies were conducted in the UK (*n* = 41, 59%) or the US (*n* = 14, 20%), followed by Australia (n = 7), Canada (n = 3), Ireland (n = 2), Belgium (*n* = 1) and Germany (*n* = 1) (Table 5). All studies were written in English with the exception of the Belgian study which was in Dutch. Studies varied from individual case studies, in‐depth interviews and focus groups to surveys with open‐ended questions and qualitative secondary analysis (Appendix 3).

Sample sizes ranged from 1 individual case study to 3,103 respondents who provided open‐ended survey responses. The mean sample size was approximately 134 (median = 44). As per protocol, the studies reported implementation experiences from the perspective of individuals with a disability, or their representative respondent. Implementation successes and challenges were also reported from the perspective of funding/provider organizations.

#### 6.5.1 Participant characteristics

Approximately 9,224 eligible people were represented in the included studies. Of these approximately three‐quarters (73%, 6,689) were people with a disability or a family member/advocate; the remaining 27% (2,535) were paid/unpaid support or organizational staff. Exact figures are not available due to inconsistent or insufficient reporting of sample sizes. However, when sample size outliers were excluded, the total sample was over 3,700 (66% individuals with a disability/representatives). Ages ranged from 3 to 85+ years, although children and older people without a life‐long disability were excluded from the analysis, where possible.

The mean age was 38 years (for the 11 studies in which this was reported) and more than half (56%) of the sample was female according to the 43 (62%) studies in which the gender of participants was indicated. Eight per cent of the sample was from an ethnic minority (28 studies provided such details, *n* = 6,713). A mix of impairments was represented in the sample including physical, cognitive/intellectual, mental health, developmental, and/or multiple/secondary disabilities. Breakdown by intervention and control group (where available) can be seen in Appendix 4 (where available).

#### 6.5.2 Intervention characteristics

At least 17 different names were used to describe the intervention of interest including: ‘direct payment’, ‘in‐direct payment’, ‘self‐directed’, ‘self‐determined’, ‘self‐managed’, ‘consumer‐directed’, ‘microboard’, ‘user‐controlled’, ‘person‐centred supports’, ‘individualized supports’, ‘individual budget’, ‘private hire’, ‘individualized funding’, ‘participant direction’, ‘personal budget’, ‘individualized packages’ and ‘individualized recovery budget’. Indeed, a combination of models was used within some studies, whilst others included supplementary use of intermediary brokerage or other formal and informal supports. A full list of names and accompanying descriptions is provided in Appendix 4.

The vast majority of participants utilized a direct payment (30%) or a combination of models (25%) (Table 5). Irrespective of the type of intervention/model, the person with a disability (or their family/representative) had some degree of control over the budget, which could be used for achieving a range of personal, health and social care outcomes, although different restrictions applied across studies. Studies were excluded if a budget was restricted to one purpose only, such as supporting people in the workplace, since choice and control were limited from the outset; such models did not clearly fit the intervention as described in the study protocol. All of the interventions were financed by State funds. Nineteen studies indicated a minimum and maximum value of budgets, ranging from $139 to $12,500 per month in the United States, £92 to £7,800 in the UK, $203 to $5,708 in Australia, $167 to $7,500 in Canada and €100 to €13,000 in other European countries. These values are only indicative as they are applicable to a number of countries and time periods and do not, therefore, take into account changing currency values or other economic considerations.

### 6.6 Risk of bias in included qualitative studies

As per protocol, quality and risk of bias within qualitative studies are based on CASP and overall CerQual scores (Appendix 7). Furthermore, the discussion below was guided by, and structured according to, the relevant CerQual headings (i.e., methodological limitations, relevance, adequacy of data and coherence). This is intended to provide transparency in terms of assessing the robustness of individual study findings. However, CerQual scores, as indicated in Appendix 7, should be interpreted with caution, since CerQual is intended to assess reviews/syntheses of qualitative findings (retrospectively) rather than individual studies per se (Lewin et al., 2015). Thus, the CerQual analysis below was conducted prospectively, providing insight into how much confidence should be placed in individual studies when analyzing and interpreting the data.

Most studies (70%) had an overall CerQual score of ‘high’ or ‘moderate’, whilst only six studies (9%) were rated as ‘very low’ (Figure 3). To this end, a sensitivity analysis was conducted by removing studies with a very low CerQual score and comparing results to the analysis conducted with all studies included (Alakeson, 2007; Blumberg, Ferguson, & Ferguson, 2000; Jordan, 2004; Secker et al., 2011; Waters & Chris, 2014; Williams & Tyson, 2010).

#### 6.6.1 Methodological limitations

The methodological limitations of individual qualitative studies were determined – as recommended by Lewin et al (2015) – by using the appropriate assessment which, in this case, was the CASP toolkit. As shown in Figure 3, a substantial proportion of studies had methodological limitations, with 22 rated as ‘low’ (*n* = 10, 14%) or ‘very low’ (*n* = 12, 17%). Despite that fact that the lowest CerQual score was obtained in relation to methodological rigor, more than two‐thirds of studies (68%) were rated as ‘moderate’ to ‘high’. Very often these low scores related to insufficient detail to assess quality or the use of a primarily quantitative study design. Full details are provided in Appendix 7.

#### 6.6.2 Relevance

Relevance was judged according to the extent to which individual studies related to the overall review question in terms of context – including population, phenomenon, and setting. As discussed earlier, whilst the descriptions and implementation of the interventions varied considerably across studies, their core elements were fundamentally in line with the intervention as defined in the protocol. Consequently, ‘relevance’ had the highest CerQual rating with 87% scoring ‘high’ to ‘moderate’ and only nine studies rated as ‘low’.

#### 6.6.3 Adequacy of data

Adequacy was assessed based on the degree of richness and quantity of data presented in each individual study (Lewin et al., 2015). Most studies fared very well in this respect with 71% achieving a ‘high’ to ‘moderate’ CerQual score. Twenty studies were rated as ‘low’ (*n* = 15) or ‘very low’ (*n* = 5). As outlined in Appendix 7, the quantity of data was assessed by examining the quartile represented by the sample size and the amount of relevant data coded in the initial line‐by‐line coding exercise. The mean sample size was 44 and the mean number of codes per study was 376. The richness of data was assessed by the depth of detail, the amount of raw data provided, and the uniqueness of the data was in terms of context (e.g., population, geography and type of disability).

#### 6.6.4 Coherence

Coherence was a little more difficult to assess as outlined by Lewin et al. (2015) since the overall review findings were not clear when the CerQual assessment was being conducted. Having said that, the first round of coding had been completed and a deeper understanding of the combined data was emerging, along with preliminary patterns within the data. In order to make an assessment of coherence, the data were assessed in terms of the extent to which the findings were grounded in the data, how the authors had triangulated the findings in terms of study design (mixed qualitative methods), multiple‐respondent groups and how the findings related to international evidence. Overall, 77% of studies were rated as ‘high’ to ‘moderate’, with the remaining studies obtaining ‘low’ scores.

### 6.7 Synthesis of qualitative results

#### 6.7.1 Analysis

The analysis of qualitative data was informed by, and conducted within a realist evaluation framework which considers ‘Contexts, Mechanisms and Outcomes’ (Pawson & Tilley, 1997). As such, critical realists not only concentrate on outcomes of interest, but also the context and mechanisms under which certain outcomes are achieved. According to Jagosh (2017), context can be interpreted as anything in the backdrop, that may not formally be part of, but can impact upon, the intervention such as cultural norms and values, history, existing public policy or economic conditions. Mechanisms may be defined by underlying entities, processes or structures (Astbury & Leeuw, 2010). For social interventions, mechanisms can be a cognitive process, which stimulate or demotivates stakeholders – including those delivering the intervention (Jagosh, 2017). Context and mechanisms can, therefore, affect the outcomes or effectiveness of an intervention.

Pawson and Tilley (2004) acknowledge the most ineluctable limitation of a realist evaluation is the constant supply of new explanations for the efficiency or effectiveness of an intervention, based on programme modifications and fresh circumstances. Pawson and Tilley suggest a pragmatic approach to such limitations, which lends itself well to a meta‐synthesis of international evidence. While all eventualities cannot be anticipated, knowledge is considerably improved by systematically capturing and synthesizing shared experiences, successes and challenges.

During stage one of the analysis (reading and coding the studies), five general themes emerged all of which were colour‐coded and which included: positive (green); negative (red); potential for adverse effects (orange); contributory factors (blue); and process (purple) (see MaxMaps – 9.2). At the end of stage one, there were 18,279 individually coded pieces of text, representing 696 possible individual themes, of varying weight ‐ ranging from 1 piece of coded text (represented by 114 codes) and up to 894 pieces of coded text (pertaining to 1 code: negative/challenging). At this stage in the analysis, the first set of codes was discussed in detail with the second reviewer, who had screened full texts. Any unexpected themes were examined to ensure conceptual agreement between reviewers.

During stage two, the themes were refined by exploring relationships between the codes. The first step was to re‐examine all codes that represented just one piece of text and merging themes together, where appropriate. This reduced the total number of codes to 599. At this point, the relationships between themes were explored, leading to their subsequent refinement and the identification of four superordinate themes, under which all remaining subordinate themes were categorized.

Once studies had been conceptually folded together, a total of 544 final themes were identified including all subthemes (see Appendix 10). However, these were categorized into six levels of detail, based on Bronfenbrenner's terminology (1995), ranging from macro [Level 1] to micro [Level 6] (see Figure 4) – and consistent with the approach adopted by Fleming, McGilloway, and Barry (2016d) and Laragy & Ottmann (2011). With regard to overarching themes, most fell within the ‘implementation facilitators’ category, representing 6,289 coded pieces of text, followed by ‘implementation challenges’ (*n* = 5,111), and finally the mechanisms affecting the implementation and effectiveness of the intervention, namely the ‘process’ of implementation (*n* = 3,429) and ‘contributory factors’ (*n* = 3,132). The last two categories were ‘cross‐cutting’ themes, often overlapping with ‘implementation facilitators’ and ‘challenges’. Indeed, categorization was sometimes not straightforward or blurred due to the complex and individualized nature of the social intervention in question. This is addressed in more detail below.

As shown in the example below, MAXMaps were used to examine relationships between codes and, in particular, ‘co‐occurring codes’. Co‐occurring codes relate to a piece of text that had two or more codes assigned to it. Generally, co‐occurring codes which appeared 10 per cent of the time were examined, but when this produced too much (or too little) data, the percentage was adjusted accordingly until meaningful results emerged. For example, 662 coded pieces of text were identified as pertaining to the theme of ‘perceived benefits’ and therefore, 10 per cent of this figure (or 60) were used to filter the co‐occurring codes (i.e., codes that co‐occurred 60 times or more (across all 69 studies) in relation to ‘perceived benefits’ (see Figure 5). Each theme is discussed below in more detail.

The remainder of this results section will summarize the qualitative findings in a narrative manner, using illustrative quotations to support and amplify key points. A more detailed analysis based on the use of MAXMaps and the identification of key concepts, theories and co‐occurring themes, is provided later in Sections 9.2.1–9.2.3. As mentioned previously, a sensitivity analysis was carried out to determine if the MAXMaps of co‐occurring themes were affected by the removal of studies with a very low CerQual score from the analysis. The results from this sensitivity analysis generally led to little or no change to the analysis. The detail of each sensitivity analysis is presented in Sections 9.2.1–9.2.3.

Data will be presented in two main sections, which examine respectively the successes or implementation facilitators (6.7.2) and challenges to implementation (6.7.3). Two cross‐cutting themes ‐ ‘processes’ and ‘contributory factors’ ‐ will be discussed in parallel and intermittently dispersed throughout the results sections, as appropriate. Some of the key messages from these themes will be expanded below, informed by the use of more MAXMaps and contextualized with the use of selected illustrative quotes transcribed directly from included studies and based both on participants’ actual responses as well as comments from the authors.

#### 6.7.2 Overarching (Macro) theme 1: Implementation facilitators

The first overarching (Macro ‐ level 1) theme – ‘implementation facilitators’ ‐ contained three macro (level 2) categories or subthemes relating to: (a) ‘perceived benefits’ for people with a disability or their representative (Appendix 10 – rows 376–451), (b) ‘mechanisms of success’ (Appendix 10 – rows 288–375), and (c) the perspectives of staff or organizational representatives (Appendix 10 – rows 101–125) (see Figure 6). Each is described in more detail below.

##### Perceived benefit

**Figure 26:**
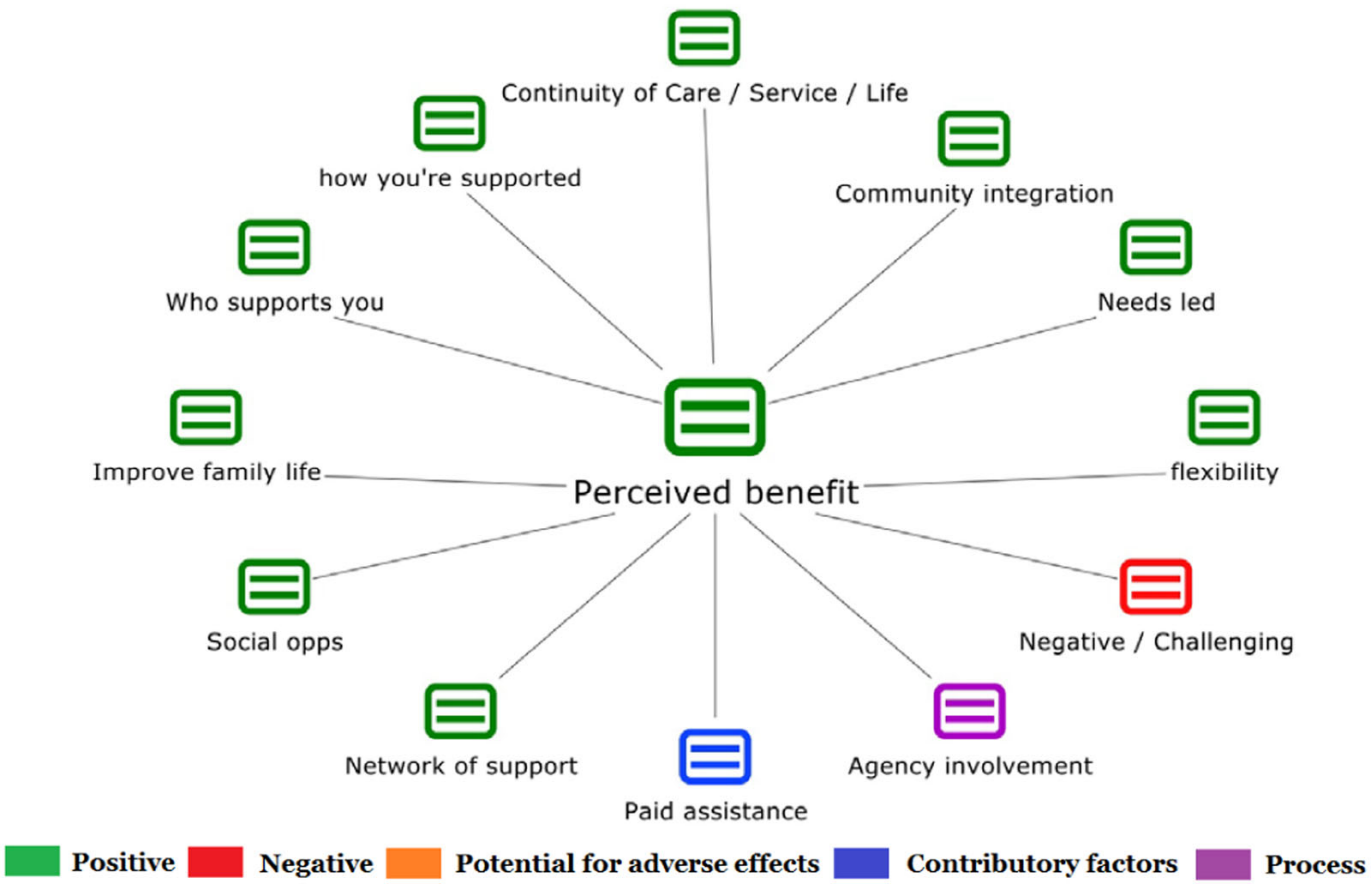
Codes co‐occurring with “perceived benefits” 50 times or more [Color figure can be viewed at wileyonlinelibrary.com]

Perceived benefits was, by far, the most commonly occurring theme across the whole qualitative analysis, accounting for 18% of all codes, including 79 subordinate themes (rows 379–454, Appendix 10). The most frequently cited co‐occurring themes are displayed in the MAXMap shown in Figure 26. These included: flexibility, a needs led approach, continuity of care/life, community integration, improved family life and social opportunities. It should be noted that perceived benefits did not only refer to positive outcomes, but also highlighted contextual factors and mechanisms that facilitated successful implementation, for example: network of support, paid assistance and agency involvement.

###### Flexibility

**Figure 27:**
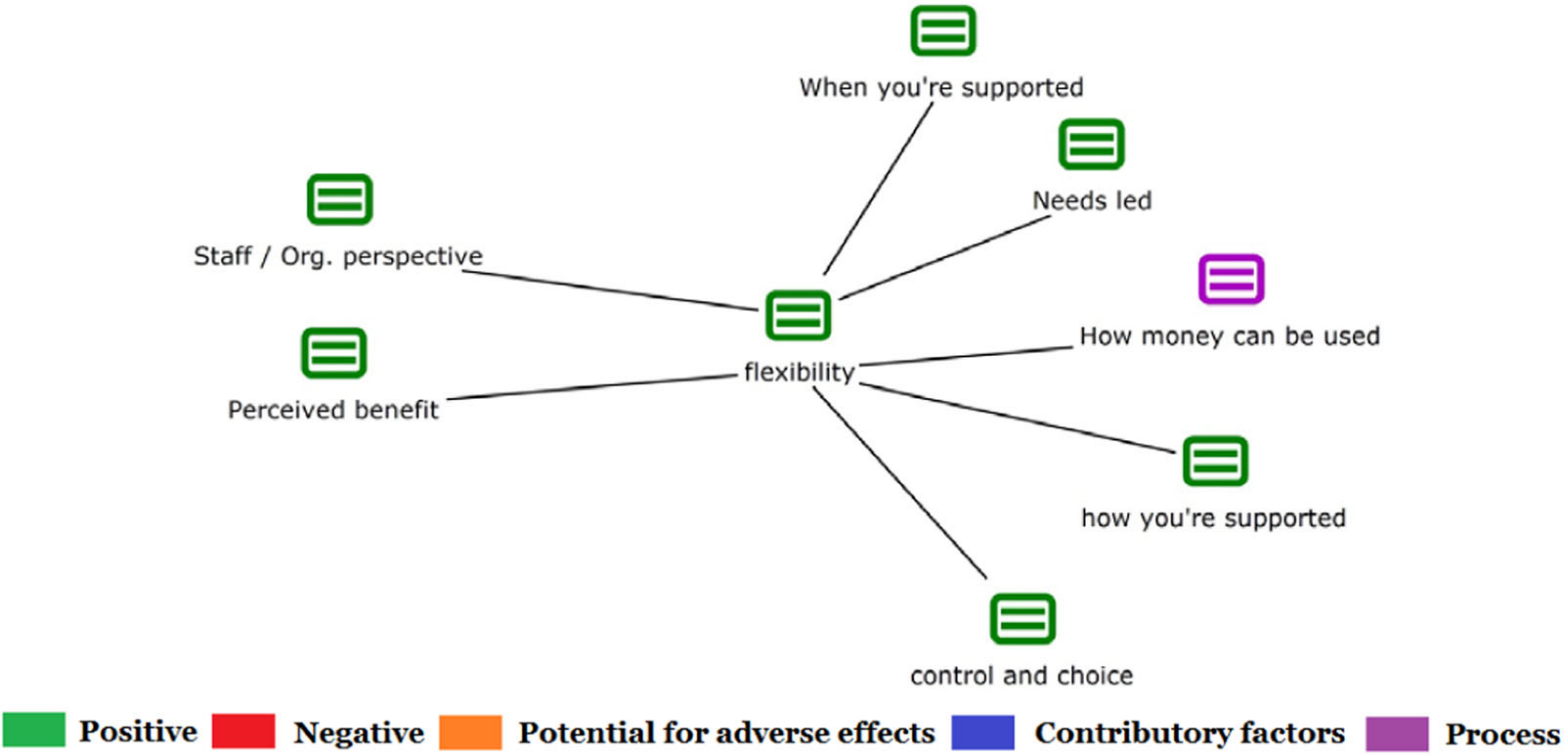
Codes co‐occurring with “flexibility” 12 times or more [Color figure can be viewed at wileyonlinelibrary.com]

Flexibility was generally associated with increased choice and control, but specific aspects frequently mentioned were: the extent to which the intervention was seen as ‘needs led’; the flexibility of the intervention in terms of type and timing of support; and flexibility in how the funding could be used (Figure 27). The quotes below reflect some of these commonly reported views: (Box 1)

Box 1 **Selection of illustrative quotations pertaining to flexibility**

***Needs led***

*“Respondents universally expressed the belief that participant direction enabled them to tailor the individuals’ supports and services to their specific needs.”* (Gross, Wallace, Blue‐Banning, Summers, & Turnbull, 2013)

**Type and timing of support**

“*With an individual budget, this consumer in Michigan has been able to hire an assistant to work with her on social skill development at times that meet the consumer's need and not vice versa.”* (Alakeson, 2007)

***How funding can be used***

*“Consumers were able to get therapies and equipment such as communication devices and lifts that were not accessible before or took years to get.” (supporter)* (Vinton, 2010)

***Freedom to choose who supports you***

*“I wanted to choose a male the same approximate age as my son to hang out with and do appropriate activities.”*

*“I wanted to choose the person who was coming into my house and our lives.”* (Butler, 2006)

In relation to the latter quotation, people usually valued particular attributes in their personal assistants, which influenced their decision in terms of who supported them (see rows 363–369, Appendix 10).

###### Freedom

‘Freedom’, was the most cited perceived benefit overall, representing 23% (773) coded pieces of text. Some of these freedoms have been discussed above, i.e., freedom to choose ‘who supports you’, as well as, ‘how’, ‘when’ and ‘where’ the support is provided. However, freedom also extended to personal freedoms such as ‘perceived autonomy’, ‘self‐determination’, ‘self‐direction’, ‘self‐reliance’, ‘sense of empowerment’, ‘space and freedom’ and ‘freedom to make mistakes’ (see rows 394–408, Appendix 10, for full list of ‘freedom’ themes).

*“I get to choose who, where and what. I wasn't comfortable when we had the lady coming in, putting me to bed at 6 and getting me up at 9, I’m 25, I don't want a complete stranger coming in to my house and washing my hair for me. Now, I can choose somebody that I trust and that I'm comfortable around.” (PSI service user)* (Sheikh, Vanson, Comber, & Watts, 2012)

*“*…*freedom to make our own choices, and to fail. Let us fail if need be. By failing, we can learn from our failures. If we do fail, do not blame it solely on our disability. We are only human after all”. (Participant)* (O'Brien, 2015)

###### Improved self‐image

Improved self‐image, self‐belief and self‐esteem were frequently cited benefits for people with a disability, representing 12% (402) pieces of coded text. As can be seen from Appendix 10 (rows 412–435), these improvements were multi‐faceted. Participants reported feeling more confident, having hope and a more positive outlook in life, in turn, feeling less stress and anxiety. They also reported feeling more resilient with self‐managing behaviour which had the knock‐on effect of improving perceived self‐worth. People also reported enhanced emotional experiences, feeling more safe and ‘cared for’:

*“It's hard to describe, the feeling you get inside when you feel so positive you know, the feeling that you're moving in the right direction*… *(Tim)”* (Coyle, 2009)

*“Everything in my life is just better, have a direction for my future*…*feel more confident, happy and really excited about my future” (C8)* (Buchanan, Peterson, & Falkmer, 2014)

###### ‘More bang for your buck’

This theme was considerable in size (representing 12% (384) pieces of coded text) and incorporated two conceptually different subthemes (rows 439–445). The first was a perceived value for money, in the conventional sense, with people reporting being able to shop around for the best value, or indeed make savings by removing the middle man:

*I get more so that's wonderful*… *I never could have afforded to go to pool therapy on my own…. You get so much more bang for your buck. You get more for the money as far as product goods, and hours of service.* (San Antonio & Niles, 2005)

**Figure 28:**
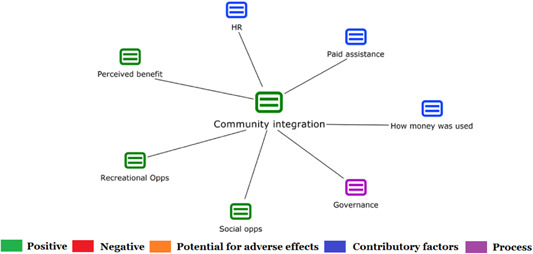
Codes co‐occurring with “community integration” 10 times or more [Color figure can be viewed at wileyonlinelibrary.com]

The second, perhaps more important theme in relation to value for money, was the perception that people could avail of better opportunities in terms of social and recreational opportunities, getting outdoors and being able to contribute to society and the community through civic participation. Unsurprisingly, many of these ‘new opportunities’ were closely associated with community integration (Figure 28). The importance of this community integration cannot be understated, and is threaded throughout the results (Box 2).

Box 2 **Selection of illustrative quotations pertaining to community integration**

***Community integration***

*“We run into some of his friends around town. He has become a part of his own community. I have lived here for 30 years, but people didn't know my son. Now they do.”* (Conroy, Brown, et al., 2002 *)*

***Recreational Opportunities***

*“I got a mountain bike. I enjoy having a bike and use it to go out with friends to places like Reddish Vale. I think it's a good social thing and I think it's fun and I like being out in the fresh air* (Eost‐Telling, 2010)

***Social Opportunities***

*‘I’m able to go out with my friends as and when I can and it means that I feel more positive about things than I did when I had more limited opportunities to do things’. Case study 21* (Homer & Gilder, 2008)

***Having paid assistance***

*“direct payments have ‘permitted’ disabled people to employ personal assistants, a facility that, in turn, has enabled them to participate in many activities outside the home, such as shopping trips, attending education and training courses, and leisure activities: pursuits which many non‐disabled people take for granted, but which are often denied to people who have their personal support needs met through less flexible arrangements.* (Carmichael & Brown, 2002)

###### Blurring of themes – Food for thought

Before moving onto the second major subtheme here, it is worth noting some of the contradictions within the data. For example, one of the key themes for ‘perceived benefits’ illustrated in the MAXMap (Figure 26) was ‘negative/challenging’. It may seem odd that the ‘negative /challenging’ theme would co‐occur with ‘perceived benefits’ but this demonstrates a blurring of concepts which can be explained by the individualized nature of the intervention; thus, for one person, directly employing support workers might be perceived as empowering, whilst for another, it may be stressful. This is illustrated by the following quotes:

*Perceived positively*

*“I cannot begin to describe the difference employing my own care has made to me – Being able to choose has given me freedom in myself.”* (Oliver & Zarb, 1992)

*Perceived negatively*

*“There are times when I just put my head in my hands and wonder why on earth I am putting myself through all the hassle of employing people when I could theoretically receive an equivalent service—it is a lot of extra work and a lot of extra stress and strain.* (Carmichael & Brown, 2002)

###### Agency involvement

**Figure 29:**
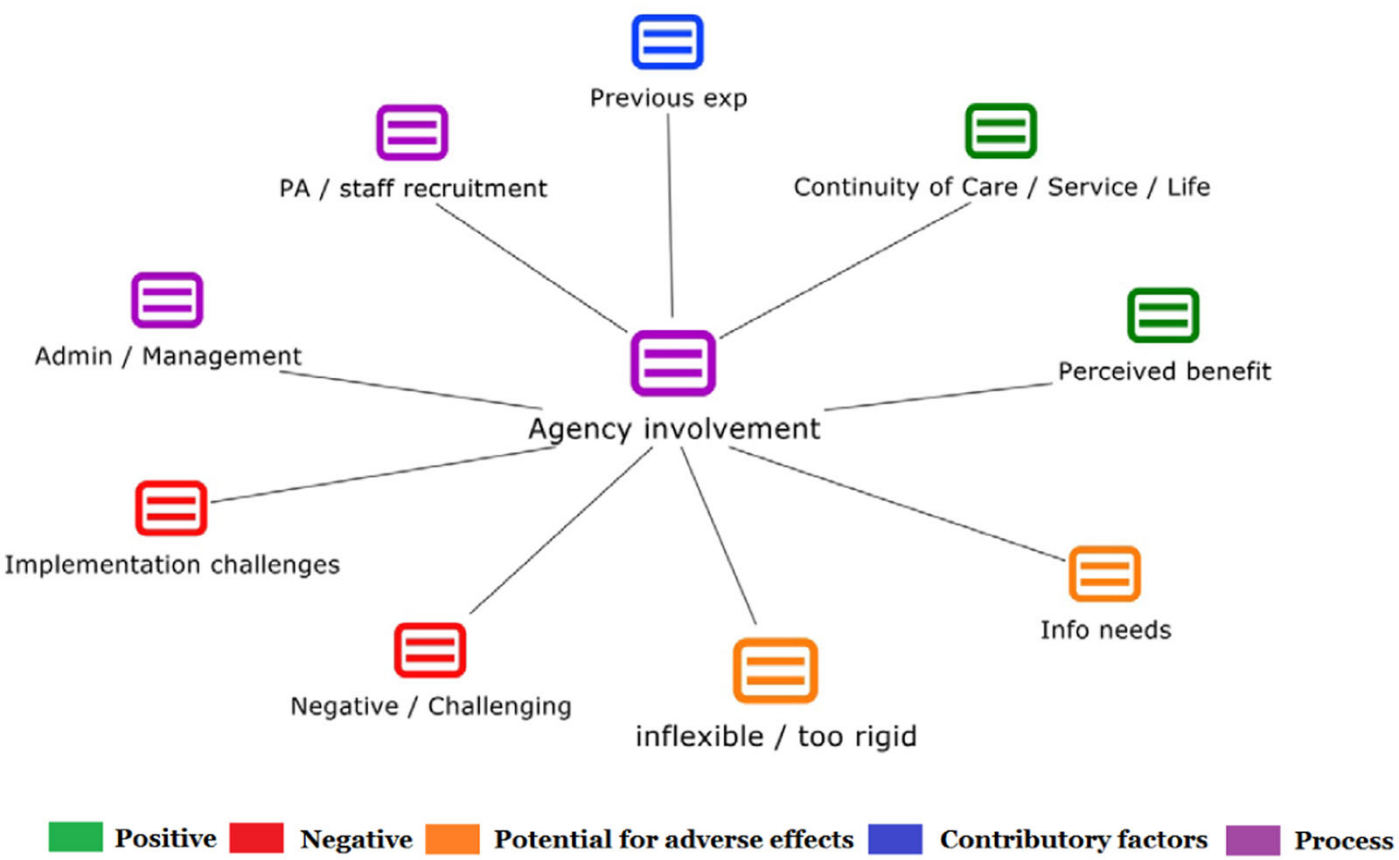
Codes co‐occurring with ‘Agency involvement’ 15 times of more [Color figure can be viewed at wileyonlinelibrary.com]

Another example of a, conceptually ‘blurred’, co‐occurring theme is ‘Agency involvement’. This theme is, in fact, categorized as a ‘cross‐cutting theme’, as mentioned previously. Cross‐cutting themes are, generally, associated with both positive and negative responses, as is demonstrated in the MAXMap associated with ‘agency involvement’ (Figure 29). In terms of perceived benefits, there was a strong association with the positively perceived ‘continuity of care/service’.

*“Once they received the direct payment they continued to use the same agency they were already using to purchase care privately; Angela had a good relationship with the agency, and the agency could ensure the carer provided was familiar to Catherine.”* (Kinnaird & Fearnley, 2010)

**Figure 30:**
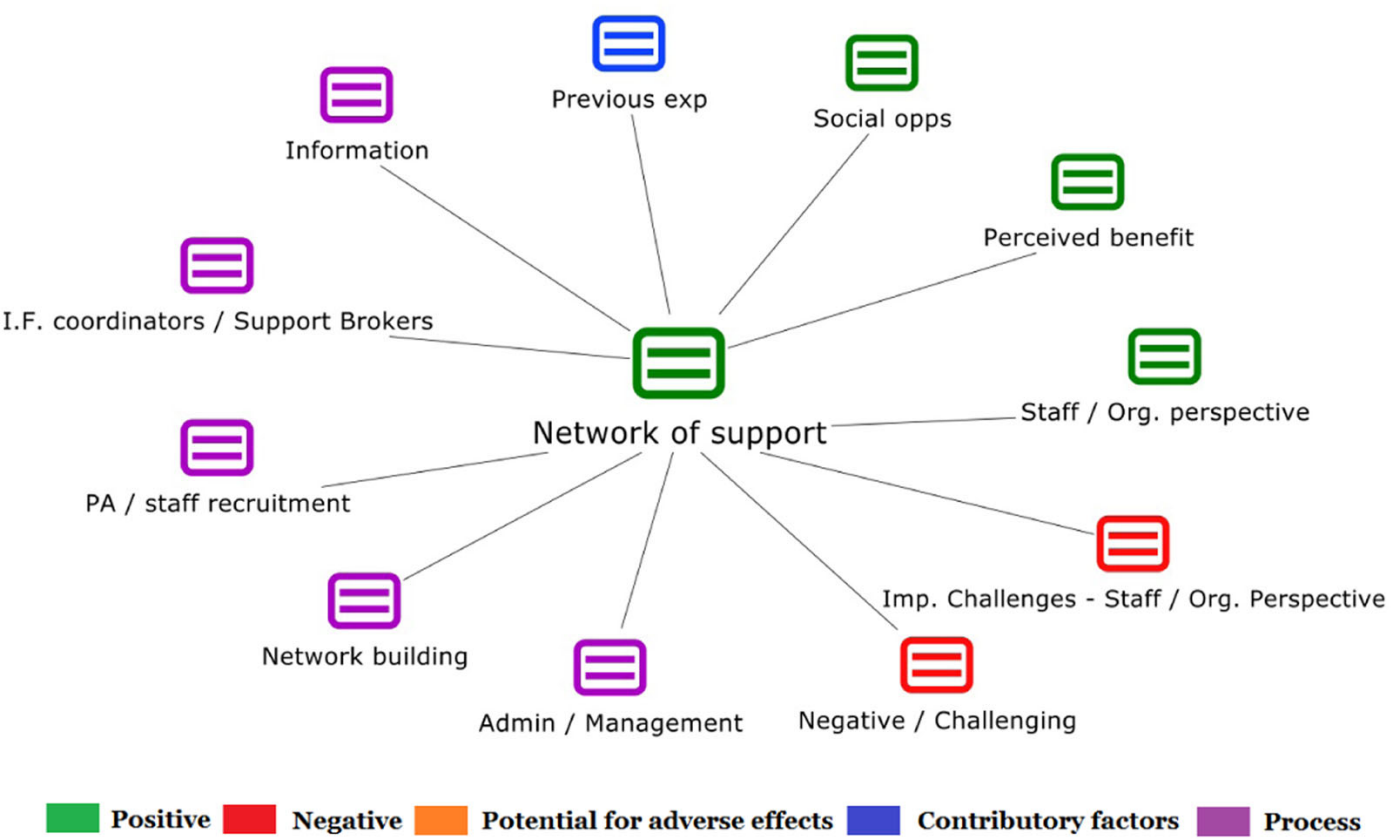
Codes co‐occurring 12 times of more with ‘network of support’ [Color figure can be viewed at wileyonlinelibrary.com]

Receiving help from agencies was often reported to relieve stress for people with a disability or their representative(s), stress that was often associated with staff recruitment or general management of an individualized fund (Figure 29).

##### Mechanisms of success

Mechanisms of success was the second major subtheme within ‘implementation facilitators’ and involved 2,702 coded pieces of text and 87 subthemes (rows 291–378, Appendix 10). The main subthemes will be discussed in this section and again supported with the use of selected illustrative quotations. These included: relationships, network of support, trust, financial recognition for voluntary work, appropriate pay, shift in power and thinking creatively.

###### Relationships

‘Relationships’ was the most common theme, with ‘network of support’ the most frequently occurring sub‐theme (rows 347–374, Appendix 10).

####### Network of support

A MAXMap analysis highlighted the integral role that the ‘network of support’ for the person with a disability plays in the complex processes associated with receiving and managing an individualised fund. This network of support typically comprised unpaid supports, such as family, friends and colleagues, but the analysis (Figure 30) clearly indicates that paid coordinators or support brokers were also strongly associated with the person's network of support. The types of support offered, included sourcing information, recruiting staff, helping to broaden the person's network and finally providing assistance with administrative and managements tasks. It should be noted that the network of support was also sometimes perceived negatively by people with a disability and staff/organizational representatives, aspects which are discussed later.

*“Find a family or a good friend you can count on for back‐up because you never know when your daily caregiver isn't going to show up. You'd have some sort of emergency back‐up that you know will be there.”* (Young & Sikma, 2003)

####### Collaborative relationships

Collaborative relationships were also often cited as important. This was frequently linked to ‘shared learning’ and ‘shared understanding’. Such collaborations ranged from individual/family dynamics to shared learning among support organizations and government agencies. People with a disability often spoke about PAs and their network of support having a ‘better understanding’ as a result of individualized funding, while others hired their family because they felt that they had a better understanding of their needs.

*Collaborative relationships between individuals and providers*

*Key factors for successful partnerships included having positive, collaborative relationships between support workers, person with disability and family members and regular communication between family and service providers.* ( A. Jones et al., 2015)

*Collaboration between agencies / departments*

*One fiscal manager that we interviewed felt that a real benefit of the project was that is forced and fiscal and program people to work together and gain an understanding of how all their jobs impact peoples’ lives.* (Conroy, Brown, et al., 2002)

A closely related cross‐cutting theme was ‘interpersonal relationships’ (rows 69–77, Appendix 10). Among these were consumer attributes, with certain characteristics enabling a more successful and collaborative relationships – including being proactive and open to new ideas. 

*His strength, humour, and flexibility have helped him to attract and maintain a group of supports who share his interests, appreciate his individuality, and view him as their friend.* (Malette, 1996)

Other important aspects that affected relationships were 1) ‘financial recognition for voluntary work’ (amongst others – see Appendix 10, rows 305–308), and 2) ‘trust’; the latter emerged throughout the results.

*Financial recognition for voluntary work*

**Figure 31:**
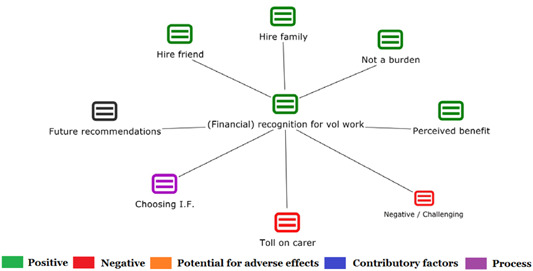
Codes co‐occurring 3 times of more with ‘Financial recognition for voluntary work’ [Color figure can be viewed at wileyonlinelibrary.com]

In the context of relationships, a MAXMap (Figure 31) revealed that ‘financial recognition for voluntary work’ was one of the reasons why people choose to take up individualized funding. It was related to the ability to hire family or friends, and sometimes meant that people with a disability no longer viewed themselves as a burden, since they were able to financially reward work that had previously been provided voluntarily:

**Figure 32:**
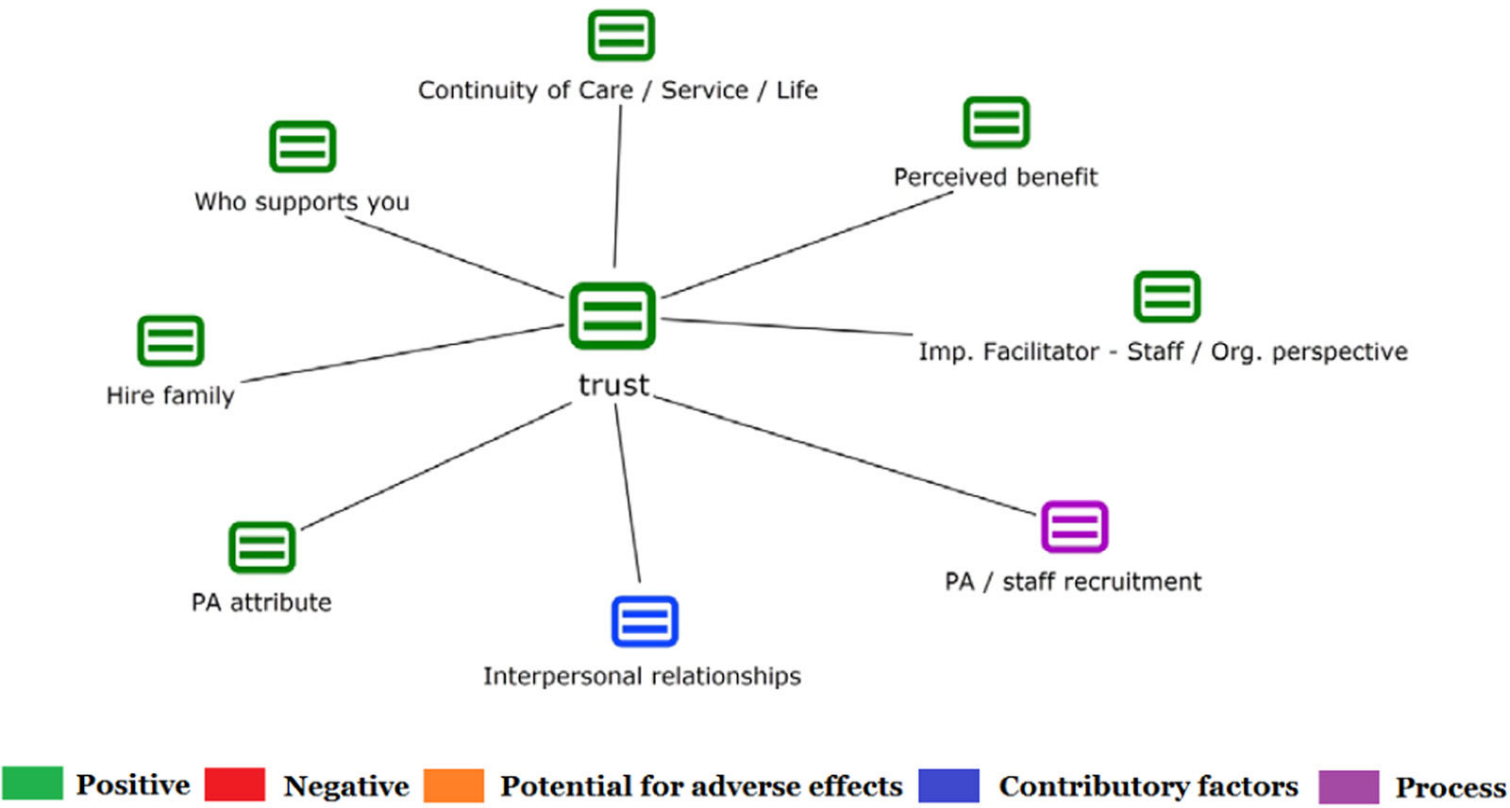
Codes co‐occurring 6 times of more with ‘trust’ [Color figure can be viewed at wileyonlinelibrary.com]

*Control over life*

*“It makes me happier that someone is now getting paid to do the jobs, like showering me. I think it is a job someone should get paid to do. It has given me more control over my life.* (Adams & Godwin, 2008)

*Valued role*

*"You get something and it's nice to get something for the care you provide. So it is socially valued. "(carer)* (Breda et al., 2004)

*Less of a burden*

*“Well I had to rely on my friends to come and help us. And I didn't like it. I couldn't pay them anything, so I just had to rely on people fitting us in really. There is a big difference now because I feel like they're not doing it for nothing. I don't feel as guilty because they're getting something.” Personal budget holder* (Lambert, Lister, & Keith, 2011)

*Trust*

Trust was discussed in relation to all relationship types, paid and unpaid, and often directly impacted continuity of care/service/life. When non‐family members were hired, people often spoke of hiring a person ‘known to the individual/family’ (sometimes a friend), again reinforcing the importance of trust.

*“Many people have very personal needs, such as assistance with bathing, and this program allows them to choose people with whom they are comfortable. As one person put it, “I can choose people I trust.”* (Walker et al., 1996)

Other important (albeit less frequently cited) ‘relationship’ subthemes can be seen in Appendix 10 (rows 347–378).

###### Other important ‘meso’ and ‘micro’ subthemes

There were many other meso and micro themes relating to ‘mechanisms of success’ (see rows 292–346). A small number will now be highlighted before moving onto the final subtheme under ‘implementation facilitators’. One such mechanism of success was the changing dynamics when employing supports directly. The ‘shift in power’ from ‘agencies’ to the person with a disability/representative was a common theme, empowering users to ensure high quality supports are in place.

*“I didn't actually know I could be the boss of him instead of him being the boss of me.” (Recipient)* (Witcher, Stalker, Roadburg, & Jones, 2000)

*“If they don't do it for you, and it is a reasonable need, then you have the authority to fire them and get somebody else…[the most important benefit is] to get back in control of your life again.”* (Eckert, San Antonio, & Siegel, 2002)

Furthermore, participants identified a number of mechanisms as integral to success including being a good employer, treating staff well and offering an appropriate rate of pay.

*“I get to select my PAs pay rate; I like to pay my PAs as much as possible on Sundays and Bank Holidays. This way, they do not mind working on these days”.*(O'Brien, 2015)

*Thinking creatively / long‐term vision with short term gaols*

‘Thinking innovatively / creatively’, ‘transparency’, ‘inclusivity’, and ‘positive‐risk taking’ were all viewed positively. Having a ‘long‐term aspirational vision / plan’, facilitated by ‘achievable short term goals’ was often cited, and was linked with a perceived ‘sense of purpose’.

*“For another person, one of his family members spoke of him identifying a long term goal of moving out of his family home but that he needed some help in identifying the smaller goals needed in order to realise this goal.” “Cooking healthier meals and buying appropriate ingredients were some of her current goals.”* (A. Jones et al., 2015)

##### Implementation facilitators from staff/organisational perspectives

**Figure 33:**
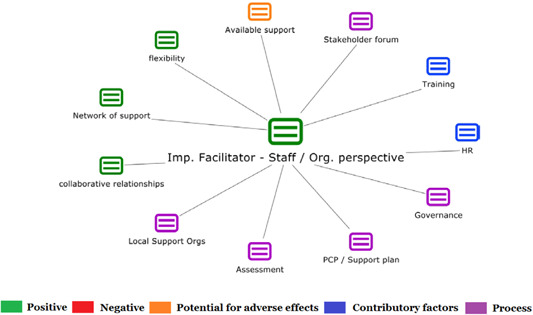
Codes co‐occurring 15 times of more with ‘Implementation facilitator – Staff / Organisational Perspective’ [Color figure can be viewed at wileyonlinelibrary.com]

This macro subtheme of ‘facilitators of success’ represented a minority of respondents (27%), and subsequently accounts for the smallest grouping of themes, totalling 292 with no meso or micro subthemes. However, MAXMaps were used to demonstrate the most common co‐occurring themes (Figure 33). There was some cross‐over with the perceived benefits (from the perspective of budget users), particularly around flexibility, network of support and collaborative relationships. Many of the remaining key facilitators (from perspective of staff/organization representatives) related to the process of implementation, such as the use of ‘local support organizations’, the ‘assessment’ process, ‘governance’ and having a ‘stakeholder forum’.

###### Local support organisations

**Figure 34:**
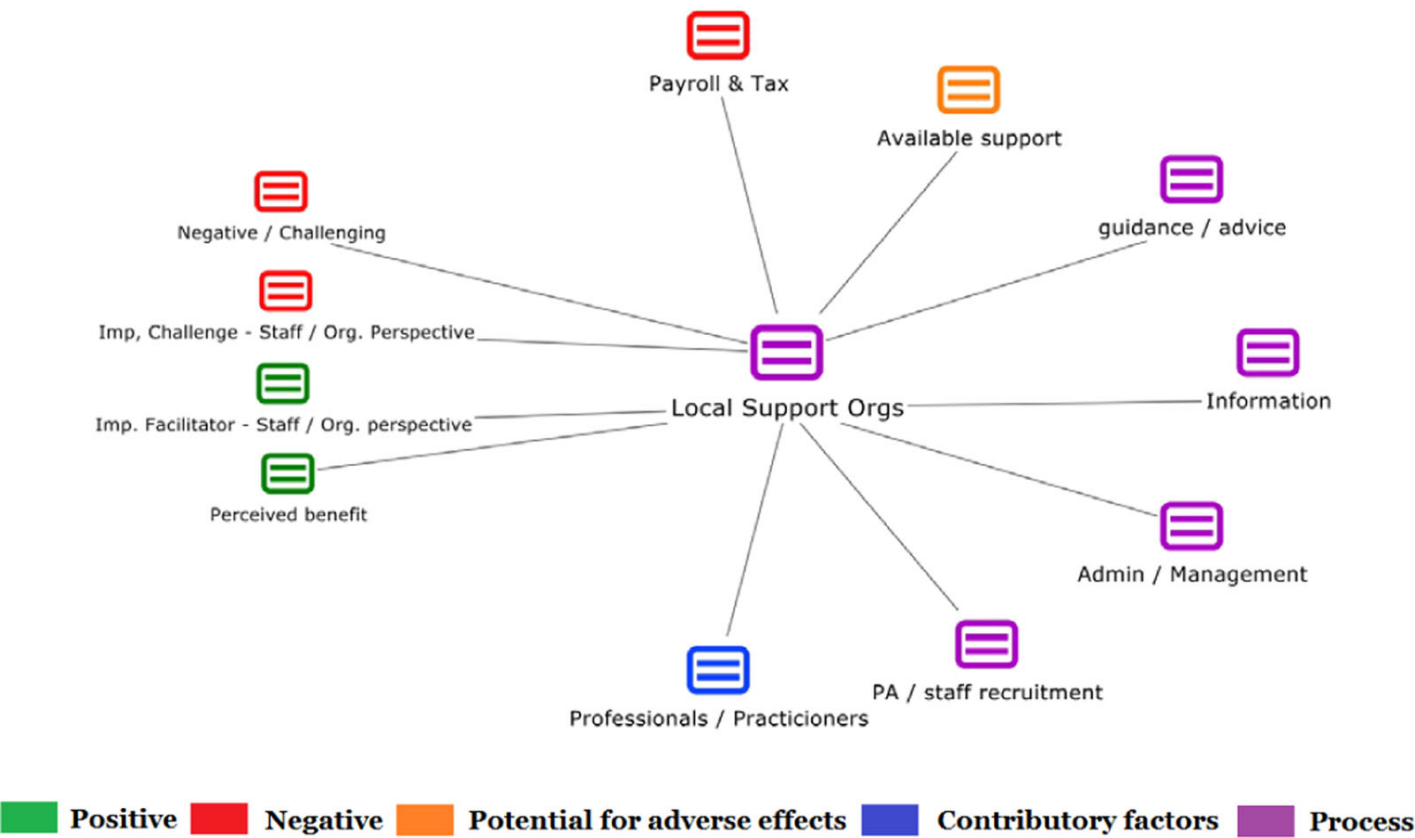
Codes co‐occurring 10 times of more with “Local support organisations’ [Color figure can be viewed at wileyonlinelibrary.com]

In relation to local support organizations, further MAXMaps (Figure 34) revealed that the strongest associations were with other cross‐cutting themes, namely the ‘provision of information’, ‘guidance and advice’, ‘support with staff recruitment’ and support with ‘administrative tasks’ such as ‘payroll and tax’.

*“In looking at why direct payments have expanded more quickly in some parts of the country than others, the link between strong user‐led support and political commitment from local authorities/trusts was highlighted.”* (Priestley et al., 2010)

*“There has to be and there are good partnerships that are in place. There has been increasing recognition of the important role user led organisations can play.” (Commissioner)* (Bola, Coldham, & Robinson, 2014)

It should be noted that there were major concerns raised about the limited capacity (of small local organizations) as numbers increased, with no alternatives in place to offer the much needed support outlined above:

*Seven respondents said that the limited capacity of local support services had been a barrier to increasing uptake of direct payments.* (Jordan, 2004)

###### Assessment

**Figure 35:**
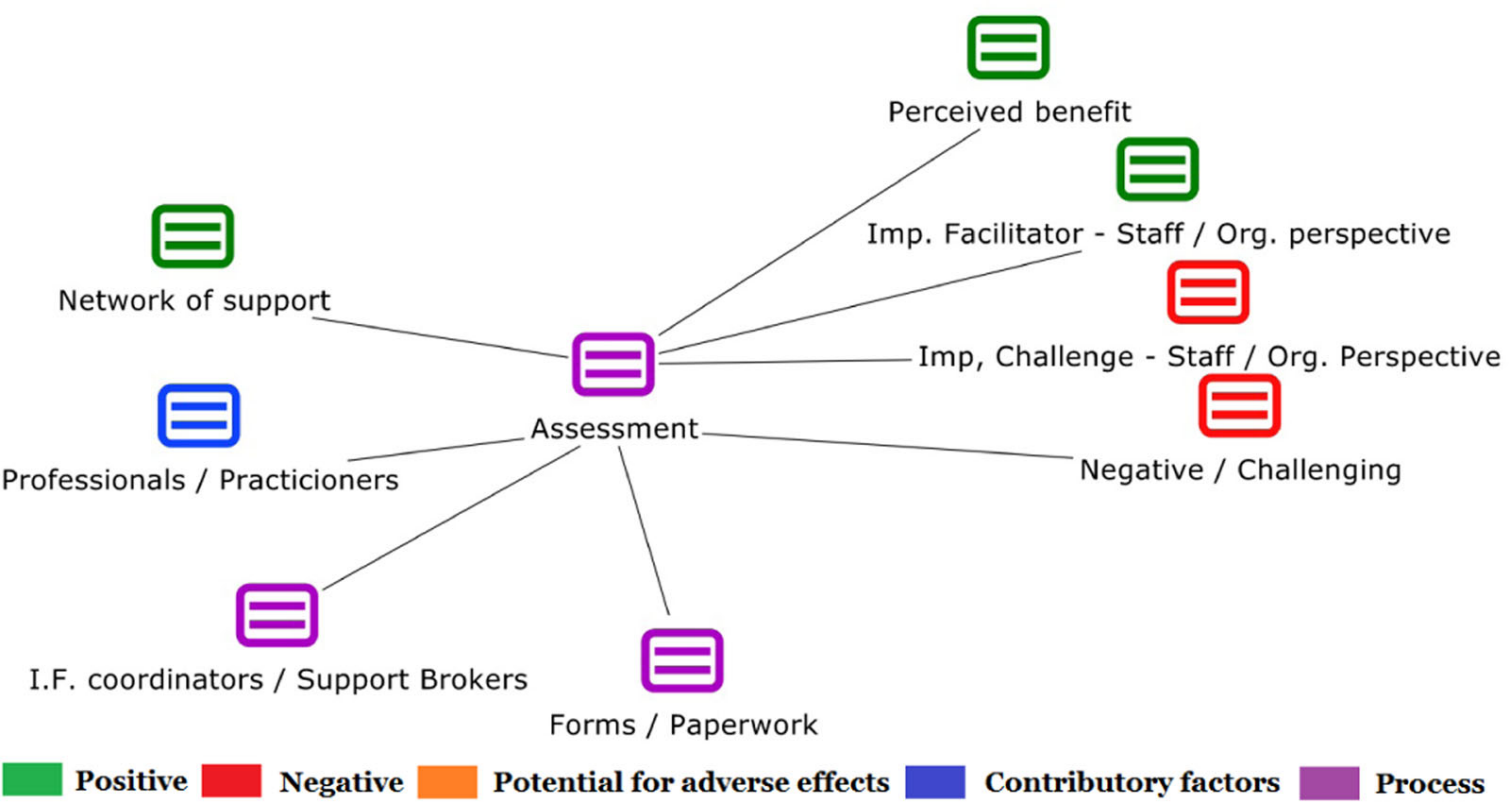
Codes co‐occurring 10 times of more with ‘assessment’ [Color figure can be viewed at wileyonlinelibrary.com]

Assessment (of need) was another process theme that was associated with implementation facilitators from the perspective of staff. Network of support was strongly associated with assessment (Figure 35). Although family members highly valued and sometimes had to fight to be present during assessment, staff were more concerned about assessing whether the person with a disability had a strong network of support, and therefore a suitable candidate for individualized funding. It should be noted, that this assessment of available support, in itself, sometimes caused discomfort for some carers.

*Assessing network of support*

*“In terms of a duty of care, I think our staff are quite clear that everyone can get a direct payment as long as there's a circle of support to help them with it, and I think we're doing that. (Team Leader)”* (Riddell et al., 2006)

*Carer discomfort with assessment of available support*

*“*…*during service user assessments practitioners are required to ask carers whether they are ‘willing and able’ to continue providing support and about any help they may need to do so.* … *some carers reported feeling uncomfortable being asked about their ‘willingness’ to continue providing care in front of the service user.”* (Glendinning, Mitchell, & Brooks, 2015)

In terms of the approach towards assessment, a ‘holistic or comprehensive approach’ was valued, as was being ‘outcome focussed’ – specifically focussing on personal, health, social care, mental health, quality of life and emotional well‐being.

###### Training and human resources

**Figure 36:**
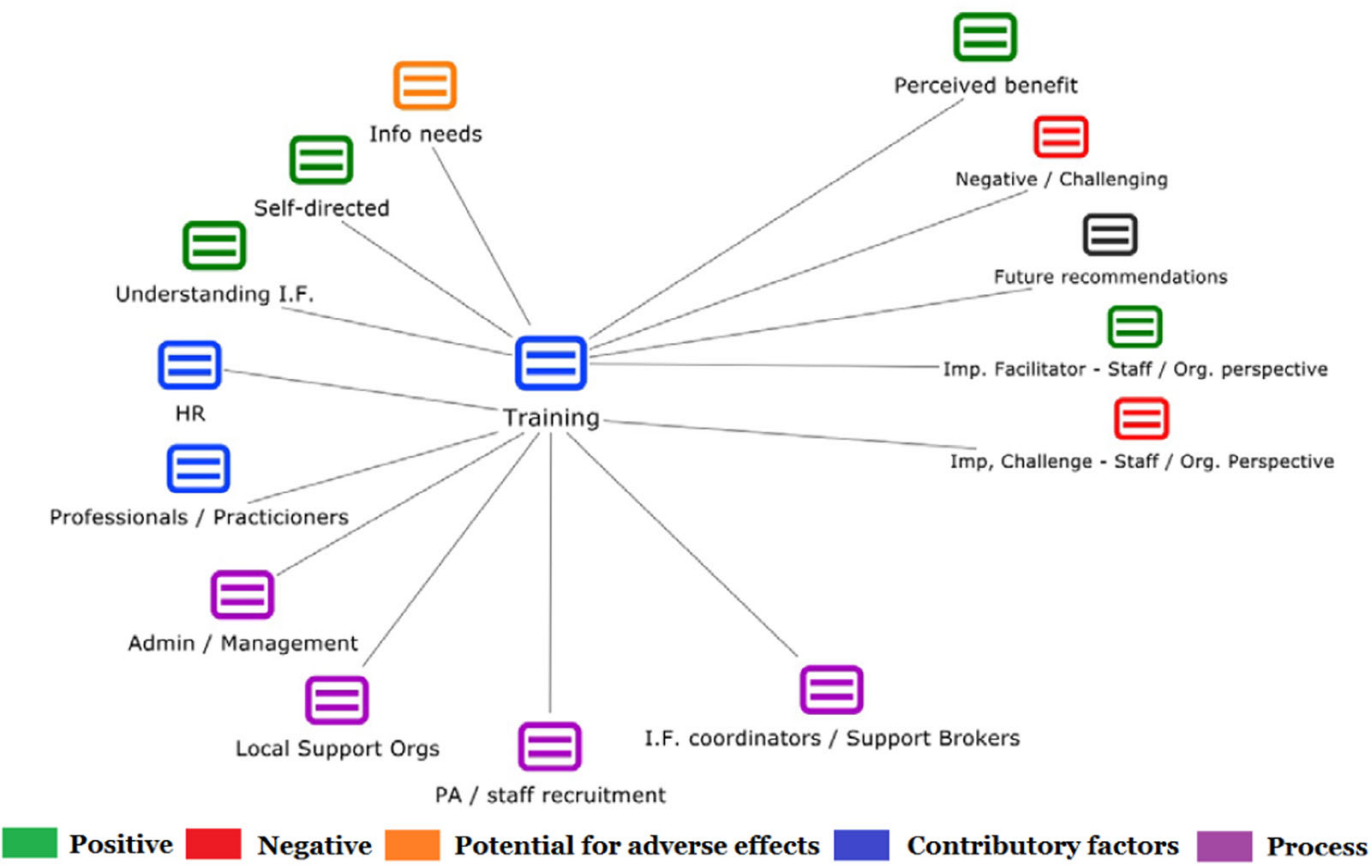
Codes co‐occurring 8 times of more with ‘training’ [Color figure can be viewed at wileyonlinelibrary.com]

Finally, analysis revealed that ‘training’ and ‘human resources’ were cross‐cutting contributory factors which facilitated (or in some cases challenged) implementation. In terms of facilitators of successful implementation, MAXMaps (Figure 36) revealed a strong association with the availability of well‐trained and informed professionals/practitioners including individualized funding coordinators/support brokers. Having a clear understanding of individualized funding was a perceived benefit whilst training was often suggested as a means of improving knowledge and understanding (for staff). Furthermore, provision of training (to people with a disability/representatives), particularly around staff recruitment and management / administrative skills, was often cited as a facilitator to successful implementation.

*“The supporting organisation saw its role as giving advice on purchasing services, providing advocacy and a payroll service, and offering support with recruitment and the employer role. Providing, or accessing, training for recipients was another of its tasks.”* (Witcher et al., 2000)

**Figure 37:**
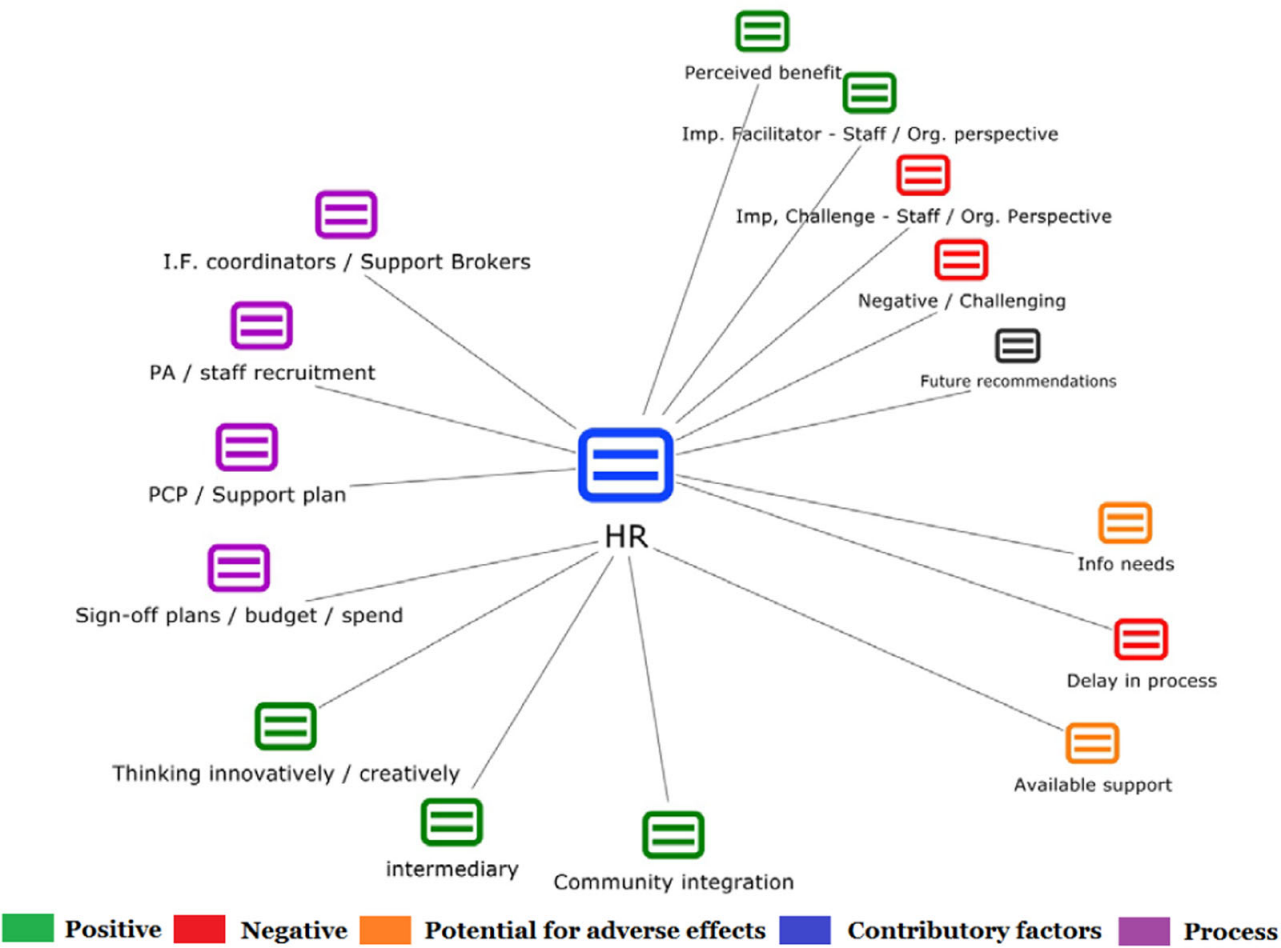
Codes co‐occurring 10 times of more with ‘Human resources’ [Color figure can be viewed at wileyonlinelibrary.com]

‘Human Resources’ ‐ itself a macro (level 2) process theme ‐ had 18 subordinate themes (rows 50–67 – Appendix 10), most of which related to different types and quality of human resources available to people with a disability. However, MAXMaps (Figure 37) revealed other key aspects associated with HR, such as ‘thinking innovatively/creatively’, ‘community integration’ (both previously discussed – 4.7.2.1 & 4.7.2.2) and the use of ‘intermediary services’.

*“This created a sense of trust and assurance for HSE staff who were otherwise cautious about releasing funds to individuals. Governance issues were of less concern due to the presence of an ‘intermediary body’”* (Fleming, McGilloway, et al., 2016d)

#### 6.7.3 Overarching (Macro) theme 2: Implementation challenges

Overall, there were fewer coded pieces of text directly linked to challenges when compared to those linked to facilitators of success (5,111 vs. 6,289). Three macro ‘level 2’ themes were identified here including: (a) ‘perceived challenges / negative aspects’ for people with a disability of their representative, (b) ‘potential problems/areas for improvement’; and (c) the perspectives of staff or organizational representatives (Appendix 10 ‐ rows 103–287;Figure 7).

##### Perceived challenges/Negative aspects

**Figure 38:**
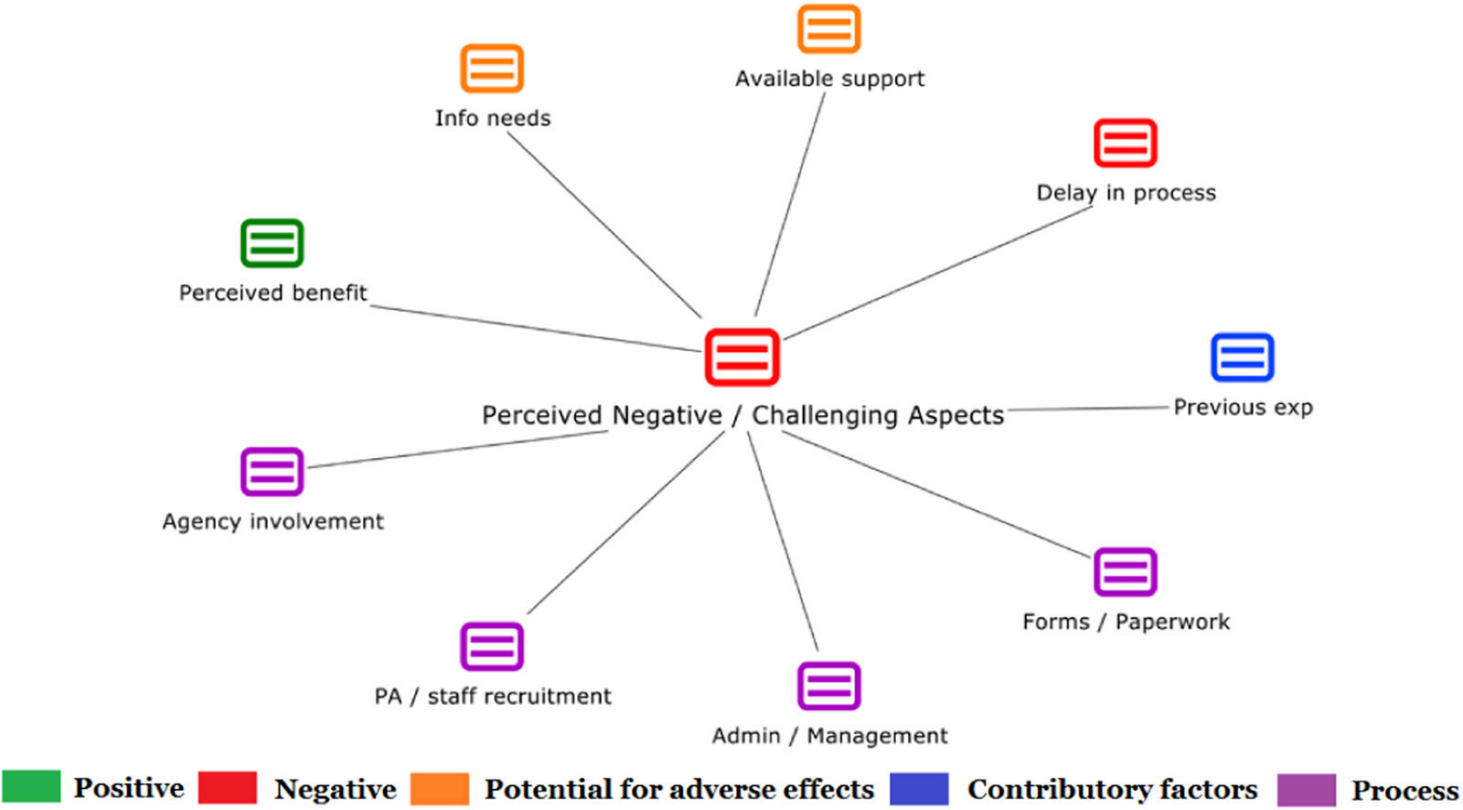
Codes co‐occurring with ‘Perceived negative / challenging aspects’ 60 times of more [Color figure can be viewed at wileyonlinelibrary.com]

There were 2,640 coded pieces of text associated with this theme, including 68 subordinate themes, categorized under ‘individual factors’ (rows 180–196), ‘external factors’ (rows 139–179) and ‘cross‐cutting challenges’ (rows 129–138). ‘Perceived challenges/negative aspects’ was also an independent theme, associated with 820 pieces of coded text. A MAXMap revealed that the majority of co‐occurring themes related to implementation ‘processes’ (Figure 38) ‐ including ‘staff recruitment’, ‘administration/management’ (particularly around forms and paperwork) and ‘information needs’ (Figure 38).

*Frequently the management of the budget – particularly the complex paperwork – was associated with additional burden* (Hatton & Waters, 2013)

###### Agency involvement

Agency involvement was another of these processes – reflecting difficult past experiences, very often cited as the reason for choosing individualized funding in the first place.

*Some people had been keen to apply for a direct payment as soon as they heard about it, seeing this as a way to stop using services which were restrictive and denied them choice and control: "as soon as I heard about it I wanted to do it: to take charge of my own care was wonderful".* (Witcher et al., 2000)

The other main concerns related to agency involvement (Figure 29) included: a perception that individualized funding was ‘too rigid or inflexible’; a need for more information; and the lack of ‘available support’ within agencies.

*Inflexible*

*“The Council can be inflexible in how they decide what to claw back ‐ Gillian needs to spend much more on support during university terms so our spending is quite erratic. On one occasion they tried to take back funds that we needed later in the year – not very helpful!’ (Carer)”* (Homer & Gilder, 2008)

*Information needs*

*“One [representative] raised a formal complaint against a practitioner after being misinformed about direct payments for people with dementia lacking capacity and the practitioner was removed from her case.”* (*Laybourne, 2014*)

*Available support*

*“The available labor pool was a barrier for some, particularly in rural areas.”* (Young & Sikma, 2003)

###### Delay in processLaybourne, 2014

One final challenge, commonly associated with perceived challenges (Figure 38), was the ‘delay in process’. The types of processes that were reported to cause delays related to ‘governance’ and specifically the ‘sign‐off’ of budgets or agreement on the proposed use of funds.

*“Participants in both groups experienced long delays at the stage of validation of their personal budget. One LA participant summed up the frustration of this process: ‘They agree it, it goes back to the social worker ‐ I don't know ‐ goes back to the finance board for them to agree. Well if one board agrees it at the council, why does it have to go all the way round the houses*…*why can't they just bang, do it.’ LA group user”* (N. Campbell et al., 2011)

Delays were also linked to the ‘review’ process, either in terms of receiving a review in a timely manner or awaiting feedback after the review had taken place. These delays were a source of ‘stress’ for individuals and their representatives. There were other challenges related to ‘delays in payroll’ (and associated tax issues) which were occasionally linked to payment of staff, but more often relating to gaining ‘access to funds’ in the first place.

*Delay receiving review appointment*

*“On the other hand, there are a number of service users who continue to experience stress and anxiety associated with delays after the set‐up phase, for example in trying to schedule reviews.”* (Sheikh et al., 2012)

*Delay in processing payments*

*“Almost every month, I make a phone call in the middle of the month and call [the local funding agency] and say: ‘Dear Mrs* …, *what is going on? Where is our money? Please remember, we need it in time. [*…*] we have certain dates, when the health insurance will debit our account’. (Margret, mother of budget user, Group 3)”* (Junne & Huber, 2014)

Lastly, ‘human resource’ issues, particularly around ‘available support’, also caused delays in the process, with little information available to people to proactively address these issues (i.e., how and where to access support workers).

*“A few pointed out that sometimes they were left without a carer, if one person left and they had to take the time to recruit another. They suggest perhaps a list of approved carers in the local area would help.”* (McGuigan et al., 2016)

The above text describes the co‐occurring themes associated with perceived challenges, as in independent theme, but as can be seen from Appendix 10 there were also many subordinate themes categorized under individual, external and cross‐cutting mechanisms. Some of these will now be discussed.

###### Individual

Challenges at an individual level related to ‘fears of losing funding’ and attendant services in the future as well as personal issues such as ‘self‐neglect’ or ‘managing ill‐health’.

*“l could worry myself sick over whether funding changes might devastate my plans. I try not to think about it because I feel that my life is in their hands.”* (Zarb & Nadash, 1994)

‘Negative emotions’ also presented challenges, such as ‘lack of motivation’ or ‘feeling isolated and lonely’; these were often linked to the transition from institutional settings to more independent living arrangements.

*Most seemed happy to be living independently, but some mentioned that they were still learning to be on their own and did not have any friends to come over to visit.* (Smith, Taub, Heaviland, Bradley, & Cheek, 2001)

Indeed, a minority of participants noted that they did not think that individualized funding was appropriate for everyone.

*“It is a great idea but it can't be a cure all for everyone*…*it will be too distressing to go through the process” (Service user)* (Rogers, Ockwell, Whittingham, & Wilson, 2009)

###### External

External factors were cited much more frequently than the above ‘individual factors’ (1,225 pieces of coded text vs. 180) and were divided into 40 subordinate themes (rows 139–179, Appendix 10). The most common of these related to the interaction with ‘third parties’. This included experiencing a ‘negative or hostile attitude’ which was most commonly associated with agency involvement or with professional/practitioners. One example was a sense of being ‘discouraged’ from availing of individualized funding in the first place, or an ‘unresponsiveness’ of staff toward users.

*“there is no information available from his social worker. In fact his social worker got really quite angry and upset with me, which was… interesting!* … *It was as if they didn't want it. Nothing positive was said about direct payments. (SP4)* (Laybourne, 2014)

Others reported that third parties were ‘serving their own interests’, rather than the interests of the person with a disability.

*When one participant expressed an interest in self‐directing his arrangement with his service provider, he was asked, “What would happen if everyone wants to go elsewhere? Where does that leave us [as an organization]?”(P3).* (Rees, 2013)

Finding the right balance of power was also challenging, due to perceived ‘paternalistic’, ‘authoritarian’ or ‘patronising’ behaviour towards people with a disability or their representatives. This among other things (e.g., ‘a weak network of support’ or ‘increased bureaucracy’ related to administration and management) was perceived to have taken a ‘toll on carers’ which, in turn, had impacted negatively on the overall experience of individualized funding.

*“I do think it's a terrific amount of work that's on top of your caring time and sometimes I feel we'd be as well just doing the caring. (Parent of adult with complex needs, Local Authority 1)”* (Riddell et al., 2006)

*Brilliant idea as long as a family member can undertake all of the paperwork involved. Has improved my sons quality of life 100% but has given me 100% more work.* (Wilson & Pickin, 2010)

‘Increased bureaucracy’ ‐ often linked to ‘logistics’ such as the ‘need for additional bank accounts’ (rows 462–490, Appendix 10) ‐ also meant there was no time for other pressing matters, such as ‘finding competent staff’. This was compounded by ‘staff turnover/retention’; these were commonly associated with other external factors such as ‘rurality’ or ‘low pay, the latter feeding directly into the second most common ‘external factor’ i.e., ‘financial issues’.

*Additional bank accounts*

*“I keep 3 separate bank accounts, one each being for DLA, ILF and DP. I have been told that I have to have these separate accounts, but this involves me in a significant amount of additional hassle, having to work out the proportion of each PAs time that needs to come out of each funding stream/account”. Case study 3* (Homer & Gilder, 2008)

*Low pay*

*In some cases, families were worried that they would lose their support workers if they could not provide them with enough paid hours, or enough pay.* (Leahy, Ong, de Meyrick, & Thaler, 2010)

Another major financial issue commonly cited was ‘disappointment in terms of the level of funding received’, which was exacerbated by a ‘lack of clarity’ about ‘how the allocated money could be used’. This ‘lack of clarity’ was exacerbated by mixed messages or experiencing inconsistent, inflexible or rigid approaches.

*“There is not a list available that tells me what I can and what I can't spend my direct payments on. Also it varies from council to council what they think you can spend your direct payment on.”* (Wilson & Pickin, 2010)

###### Cross‐cutting challenges

Lastly, cross‐cutting challenges related to both people with a disability and staff/organizational representatives. These challenges related to: ‘increased workload’; systems and processes that were ‘too complex’; processes that were ‘not inclusive’ or perceived to be ‘intrusive’; ‘inequitable distribution of funds’; and a ‘lack of trust’ and ‘risk aversion’ which, in turn, often led to difficulties ‘relinquishing control’. Ultimately, these factors, along with the many other challenges previously discussed, led to a high degree of ‘stress’ for many involved (Box 3).

Box 3 **Selection of illustrative quotations pertaining to stress**

***Increased workload***

*“The main focus was on the increased workload, a perception of high levels of pressure and stress and competing demands such as eCPA, audits, Safeguarding and new computer systems.”* (Rogers et al. 2009)

***Complexity of systems***

*“The sheer complexity of arrangements was difficult for both workers and recipients to grasp: ‘It really needs someone in the DSS or I don't know where, to sit down and get an overview of all the systems*…*because they're a mess. As someone who's working at the coal‐face, they're a mess.’"* (Witcher et al. 2000)

***Inequitable distribution of funds***

*“Really, who do we cherry pick or* … *for SDS. It's not equitable because we don't have the time to do it or offer it to all our clients. I don't” (Practitioner)* (Eost‐Telling, 2010)

***Relinquishing control***

*“Releasing control is the issue. We're such paternalistic agencies with well defined infrastructures. For years, we've had individual budget money in small sums ($3–5,000) available through our Family Support program. Now that more money is involved, there is more tension.”* (Olmstead, 1999)

##### Potential problems/Areas for improvement

This second macro sub‐theme relates to perceived problems or areas for improvement as distinct from challenges in the sense that, whilst problematic, most participants were able to adjust to, or overcome, the difficulty in order to proceed with the intervention. However, it was felt by many respondents that, if left unaddressed, these potential problems would become untenable over time. There were five categories amounting to 89 subordinate themes (rows 198–287 – Appendix 10).

The most commonly discussed concerns related to ‘operational challenges’ (rows 264–287). Among these, ‘information needs’ was by far the most cited problem with ‘inaccurate information’, ‘mixed messages’ and ‘inaccessible information’ confounding the issue further.

*Inaccurate information*

*‘Well in fact the social worker gave us the wrong information, so I was never fully aware how all the bits fitted together’. ULO participant* ( N. Campbell et al., 2011)

*Mixed messages*

*Receiving inconsistent or contradictory information served to confuse individuals more and generate extra stress and anxiety.* (Shaw, 2008)

*Inaccessible information*

*Others were overwhelmed with the sheer volume of information received on entering the scheme.* (McGuigan et al., 2016)

**Figure 39:**
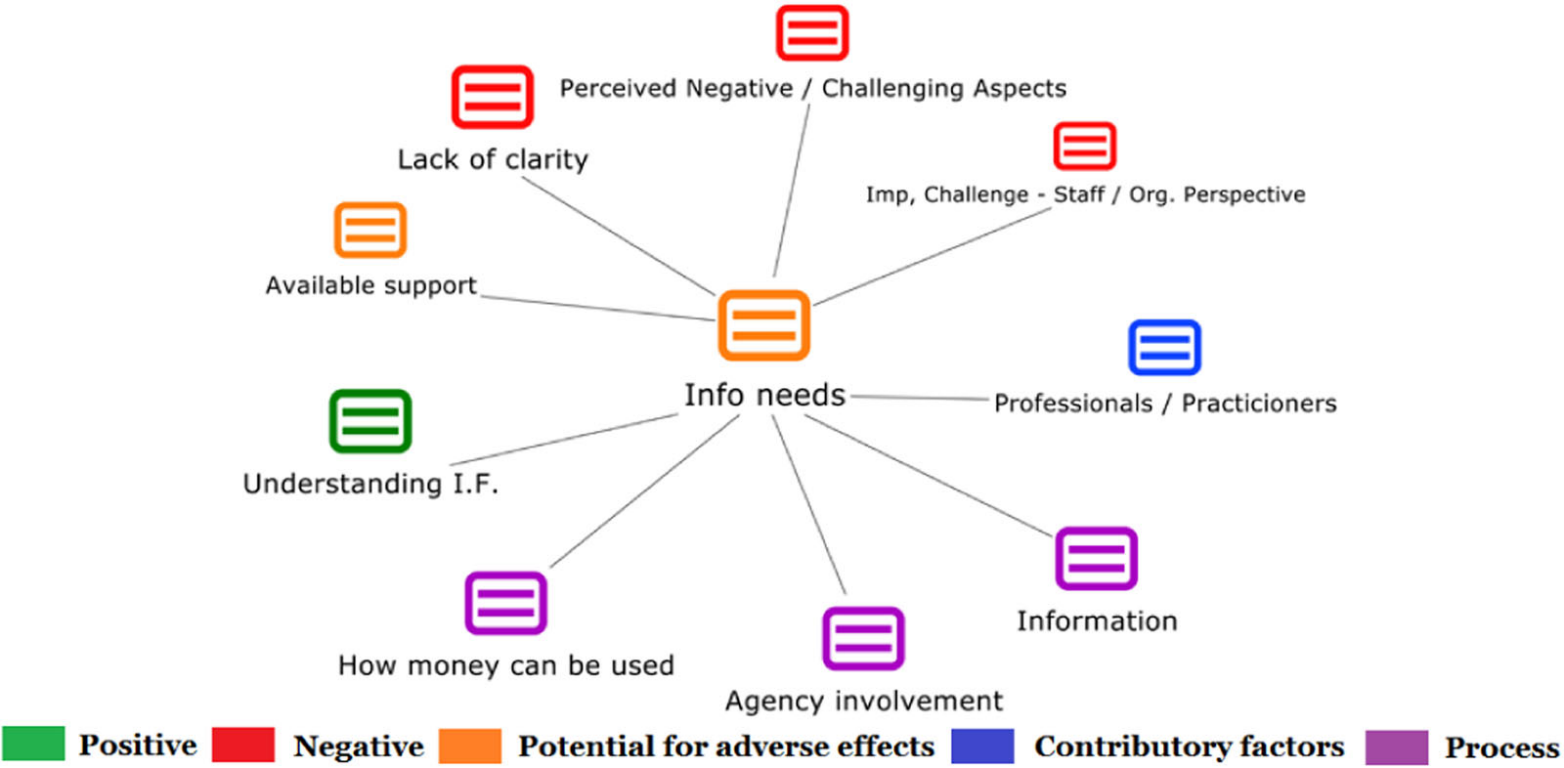
Codes co‐occurring 15 times of more with ‘Information needs’ [Color figure can be viewed at wileyonlinelibrary.com]

A MAXMap confirms these confounders, with a strong link to the theme ‘lack of clarity’ (Figure 39). Co‐occurring themes indicated that people required information from professionals/practitioners and agencies about basic aspects of implementation, namely: a deeper understanding of individualized funding, what kind of supports were available, where that support could be accessed, and what the money could be used for (amongst other things).

*One manager explained that ‘[Support Planners] are giving clients missing information about what their entitlements are’. This manager felt that the Support Planners were not sufficiently informed about the SLF, which was why they did not always provide clear information.* (A. Jones et al., 2015)

###### Cumbersome systems

The next potential problem area (operationally) was a ‘cumbersome system’.

*The difficulty comes – not with what is trying to be achieved, but rather the systems and culture within services.* (Bola et al., 2014)

*But then she warned: ‘this envisaged flexibility has been hampered by the use of systems such as performance indicators and target‐setting in the work environment; which limits the time of interactions with service users, a crucial social work function’.*(Williams & Tyson, 2010)

Micro subthemes reveal a perception that systems had an ‘inappropriate focus’ (particularly during needs assessment).

*“Some people with mental health problems raised concerns that the forms used for the questionnaire were not geared towards their needs so that they had to go through a lot of questions that were not relevant to them.”* (Newbronner et al., 2011)

Others held negative views around the utilization of the ‘medical model’ and targets/costs being the focus for staff rather than quality of services.

*Medical model*

*This potentially results in a tendency to medicalize, compartmentalize, and intrude upon daily living freedoms that would not be tolerated by those without disabilities.*(Young & Sikma, 2003)

Targets versus quality

*But subsequent contract negotiations raised concerns about moving away from a person centred approach, posing difficult questions of targets versus quality* (N. Campbell et al., 2011)

In fact, some people felt that implementing individualized funding equitably was not really a priority for staff, but rather ‘firefighting’ the more challenging or acute cases. Others perceived the system to be ‘inflexible and too rigid’, often duplicating work. ‘Inconsistent approaches’ (level 4 meso theme) were also highlighted as operational challenges, which led to frustrations, further confounding the information needs, as previously discussed.

###### Human Resources

‘Human resources’ (HR) was the second most discussed potential problem or area for improvement (rows 224–246, Appendix 10). The biggest issue relating to HR was the lack of ‘available support’, which is a theme that has come up numerous times previously.

*‘My carers seem to come and go all the time; I only receive direct payments to pay for a few hours a week. So it is not enough for someone to leave an [other] employment for and a few hours don't always appeal.’* (Shaw, 2008)

At a micro level, people reported an ‘under or over‐estimation of need’, also reflecting the ‘need for additional help’, having to ‘rely too much on informal supports’ or becoming ‘over‐reliant on one person’. Others felt they now had ‘less contact with formal services’, which posed a concern for them.

*Under‐estimation of needs*

*“My only concern relates to the fact that I am not getting enough money to cover each month. I really need someone to come in every day, rather than no‐one being here on Tuesday and Friday as happens at the moment’* (Adams & Godwin, 2008)

*Rely on informal supports*

*A family from a non‐English speaking background reported particular difficulty, as translation services were not provided, meaning that they had to rely on a family member living overseas to translate and assist in filling out the SLF application via Skype.* (A. Jones et al., 2015)

The next HR issue was the lack of training with many reporting little or no training. People with disabilities reported ‘needing skills and knowledge’ (e.g., in areas such as ‘vetting of support workers’, ‘placing adverts’, ‘rostering’ or ‘disciplinary role as employer’) but, in fact, those who supported them (paid and unpaid) also required training (e.g., facilitating a ‘journey of discovery’ as part of the person‐centred‐planning process).

*Lack of training*

*This person's family member felt that support workers were not adequately trained in how to support her daughter's mental health needs.* (A. Jones et al., 2015)

*Needing skills and knowledge*

*Managing personal assistants was not, however, always straightforward. Again, there did not seem to be much proactive practical support or training available from local authorities or third party organisations on how to manage staff, beyond completing the necessary paper work.* … *aspects which participants found challenging included addressing poor performance, asking someone to leave, and employment law.* (Lambert et al., 2011)

Another HR challenge concerned working relationships and the need to develop ‘respectful boundaries’, in order to avoid ‘conflict’.

*For other users, although happy with the much better relationship they enjoyed with staff since using direct payments, there was a feeling that boundaries between work and friendship needed to be clear. Some people had experienced problems where staff had not respected this.* (Stainton & Boyce, 2004)

Sometimes this reflected the need for paid or unpaid supporters to adjust their approach to supporting individuals with a disability (e.g., moving from paternalistic to empowering dynamic), but other times it required ‘behaviour changes’ for the individuals with a disability themselves. Such changes reflected the need to move away from ‘learned passivity’ where people (often formerly institutionalized) needed to become more independent and self‐reliant, sometimes linked to the need to let go of previous arrangements; for others it was learning to accept help on offer.

*Move from paternalistic to empowering dynamic*

*She has also suggested to me to back off. She is good! She felt he could deal with less help from me. We worked on it and she was right. He now lives without assistance from both of us. (Parent talking about support worker)* (Butler, 2006)

*Learned passivity*

*Similarly, some participants, particularly those with longer experiences of service use, did not find it easy to adjust to the opportunity to think and take responsibility for themselves: ‘I wasn't really participating… because it's sort of the [practitioner's] job to do things like that…. I didn't really want to get my hands dirty with it’ (A03, budget ongoing).* (Hamilton et al., 2015)

###### Disabling practices

Another potential problem or area for improvement related to ‘disabling practices’ (rows 197–210, Appendix 10). This category reflected, amongst other things, the sense that professionals/practitioners or agencies were acting as ‘gatekeepers to funds’ (particularly at assessment) and ‘over‐riding’ the wishes of the end users. At a micro level people sometimes felt that their ‘hands were tied’, being pressurized around decision‐making, with ‘no alternative options’ provided and therefore choice and control was limited. In a small number of cases people felt even ‘more restricted’ than before the intervention. People felt that disabling practices also extended to the wider public, with a lack of understanding of individualized funding, with the need for ‘disability awareness’ generally within society, ultimately facilitating community integration, itself heralded as a perceived success of the intervention.

*“More concerning was when this seemed to reflect a more pervasive (although not necessarily explicit) enactment of power differentials in which it was the professionals rather than the service user that set the agenda: ‘It's probably me, but I get the feeling that they think that I’m lower than them and I… shouldn't question things, I should just go along with it’ (B03, budget ongoing).”* (Hamilton et al., 2015)

The final two potential problems or areas for improvement related to ‘financial issues’ and ‘negative emotions/perceptions’.

###### Financial issues

Anxiety and stress was experienced when participants spoke about future problems that could emerge due to, for example, ‘budget cuts’ whereby funders may try to ‘claw back funds’ or discontinue individualized funding. Other financial issues raised concerned ‘charges for people with a disability’ to cover, for example, administration costs. ‘Hidden costs’ were also flagged amongst the ‘unsustainable’ aspects of implementation, as were disappointment with level of funding and financial issues more generally. Finally, ‘keeping funding sources separate’ was another concern for people with a disability; further complicating spending restrictions/criteria (with different needs being addressed by different funding streams), causing undue confusion and stress (Box 4).

Box 4 **Selection of illustrative quotations pertaining to financial issues**

***Hidden costs***

*“For the two people who were faced with advertising, the start up payment was woefully inadequate: an initial newspaper advert was placed and $25 did not cover the cost*… *Respondents also detailed a range of other start up costs involved, which they had to pay for themselves: insurance, payments for a personal assistant to go on a lifting and handling course, overalls and plastic aprons for personal assistants. The start up payment clearly needs to be substantially increased.”* (Leece, 2000)

***Multiple funding streams***

*“The third area of concern related to a situation which arises when there are multiple funding streams and systems are not properly integrated, leading to an increased administrative burden” (*Rummery, Bell, Bowes, Dawson, & Roberts, 2012 *)*

***Unnecessarily bureaucratic and burdensome process***

*“I keep 3 separate bank accounts, one each being for DLA, ILF and DP. I have been told that I have to have these separate accounts, but this involves me in a significant amount of additional hassle, having to work out the proportion of each PAs time that needs to come out of each funding stream/account”. Case study 3* (Homer & Gilder, 2008)

###### Negative emotions

‘Negative emotions or perceptions’ are presented in terms of subthemes (rows 247–263, Appendix 10) including ‘increased responsibilities’ associated with individualized funding, which were often apprehensively undertaken, with people sometimes feeling ‘daunted’ by the new role and responsibilities. Others felt a sense of ‘guilt’ or that they were ‘asking for too much’ or perceived themselves as a ‘burden’. This feeds into the ‘vulnerability’ experienced by people with a disability, highlighted by concerns as to ‘what would happen to them when their parents pass away’. This was exacerbated by a perceived dependency on an imperfect system that, sometimes, was not challenged for fear of ‘rocking the boat’, potentially jeopardizing the supports in place.

Finally people were often ‘suspicious’ of the system due to negative previous experiences or because of the perceived restrictive/disabling processes in place. For example, people felt that they were ‘penalized for working’, or that individualized funding was ‘set up to fail’, with agencies occasionally accused of ‘paying lip service’ to the concept of individualised funding. Left unchecked, such negative perceptions could adversely affect the delicate relationship balance, previously discussed, and therefore the need for information, communication and transparency is further reinforced.

##### Implementation challenges from perspective of staff / organizational representatives

**Figure 40:**
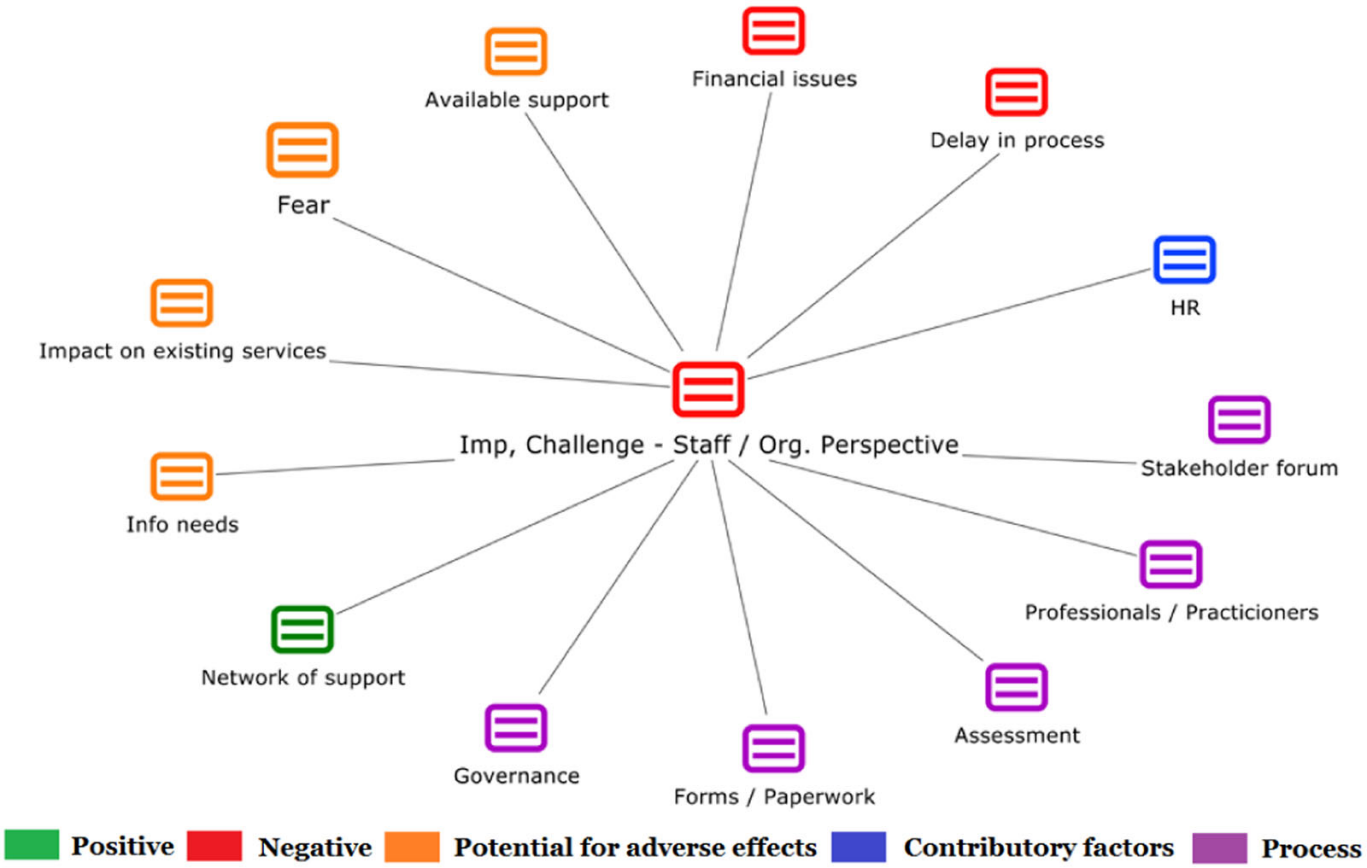
Codes co‐occurring 18 times of more with ‘Implementation challenges from perspective of staff / organisational representatives’ [Color figure can be viewed at wileyonlinelibrary.com]

This third and final macro theme, within implementation challenges, represents 779 coded pieces of text and 24 subordinate themes (rows 102–125, Appendix 10). As with facilitators of implementation, many cross‐cutting ‘processes’ and ‘contributing factors’ fed into implementation challenges, from the perspective of staff or organizational representatives. A MAXMap was produced to demonstrate the main areas of concern for this cohort of stakeholders (Figure 40). Many of issues highlighted, repeat the concerns of end users (previously presented), such as ‘available support’, ‘information needs’, ‘financial issues’, problems associated with ‘delays in process’, ‘governance’, ‘administrative tasks’, and ‘HR’ issues. However a unique concern relates to fear, one of the three subordinate themes discussed below.

###### Fear

**Figure 41:**
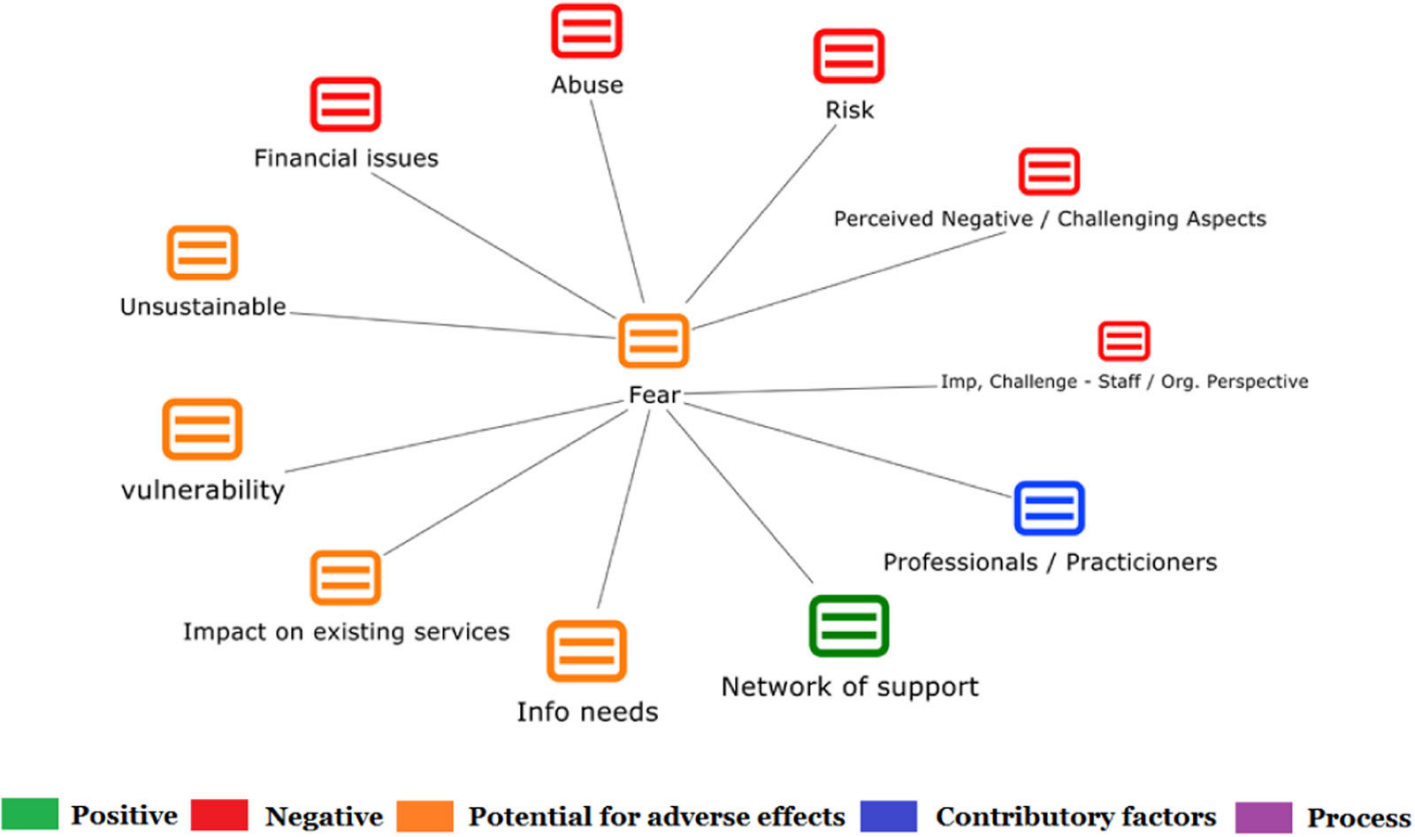
Codes co‐occurring 5 times of more with ‘Fear’ [Color figure can be viewed at wileyonlinelibrary.com]

In terms of subthemes, ‘fear’ was the most common theme associated with staff/organizational representatives (rows 105–118, Appendix 10). A MAXMap revealed that many fears were linked to perceived ‘risks’ for people with a disability (Figure 41). This included fears of ‘abuse’ (by directly employed staff or even their own network of support), with ‘vulnerabilities’ potentially being exploited by various parties. The data also revealed that risk was closely linked to ‘safeguarding’ individuals with a disability (as perceived by staff), which was sometimes linked to ‘risk aversion’ when assessing, planning and delivering activities (particularly in relation to community integration).

“‘Concerns from social workers regarding their accountability’; ‘Social work practice is still rather paternalistic in some quarters staff have concerns re: risk and control’; Perceived vulnerability of some groups/individuals’” (Jordan, 2004)

There were also fears associated with the perception that individualized funding was ‘unsustainable’, the ‘impact on existing services’ and ‘financial issues’. An associated concern, for organization representatives, related to people with disabilities ‘poaching agency staff’ for direct employment, thereby reducing the workforce within agencies.

*Impact on existing services*

‘Impact on existing services’, related to fears that individual purchasing power would lead to the ‘privatization of care’ which, in turn, would lead to loss in jobs and a potential decline in quality of supports. Furthermore, it was perceived that ‘economies of scale’ would be jeopardized ‐ with the knock‐on effect that larger service providers would dominate the market, in turn, reducing choice (Box 5).

Box 5 **Selection of illustrative quotations pertaining to impact on existing services**

***Poaching agency staff***

*‘The agency we were using couldn't guarantee the continuity of carer that I wanted for my wife so, when I got to know a good one, I asked her to leave the agency and come and work for my wife as a PA. Now she gets better pay and conditions, even paid holidays, so we are all happy’. Case study 20* (Homer & Gilder, 2008)

***Privatisation of care***

*“The use of private, not‐for‐profit and voluntary bodies to provide services was a form of privatisation and would inevitably lead to job losses for existing council workers.”* (Riddell et al., 2006)

***Quality of supports***

*This issue of private contractors not having a background in Personal Budgets, not understanding the development history or meaning of 'choice and control', and therefore missing the key point of personalisation when delivering on contracts is something that concerns service users and carers.* (Bola et al. 2014)

***Job losses***

*“Another care manager, who was also a day centre manager, experienced some conflict of interest in that, if all users went on to direct payments, the day centre would close.”* (Witcher et al., 2000)

***Domination of market by larger providers***

*“smaller providers voiced concerns that if provider organizations were not able to develop systems that could cope with varying and flexible demands from SDS users, they might not be able to continue operating if a large amount of business came that way. This would mean that fewer, larger providers could dominate the market, potentially reducing choice, increasing costs and increasing prices for SDS users.”* (Rummery et al., 2012)

###### Financial concerns

At a micro level, financial concerns often related to fears of ‘fraud’ or ‘misuse’ (of money) by people with a disability or their representative. However, as a number of people with a disability pointed out, it would be ‘self‐destructive to misuse’ the money, potentially leaving people in vulnerable or unviable situations.

It should also be noted that there was very little evidence of reported misuse of funds across the body of research. Another unsubstantiated fear was that people with a disability would ‘flood the system’ looking for individualized funding. In fact, uptake was generally lower than expected, often requiring additional and substantial efforts to boost uptake.

*“P1 pointed out that ‘it is in my interest to make sure that my money is being used well… If I spend foolishly I can't get out of bed in the morning … it's not in people's interest to let money go missing’.”* (O'Brien, 2015)

*Accommodating diverse levels of need*

Another challenge for staff, that frequently emerged, related to difficulties accommodating diverse levels of need. This was particularly challenging when transitioning, from delivering information and supports, between people with ‘high support needs’ and those with ‘ongoing support requirements’ and conversely to those who required ‘little support’. This challenge was heightened by trying to deliver individualized supports to people from ‘different backgrounds’, with ‘different life experiences’ and attendant expectations.

*“People were in vastly different situations: some were lifelong service users, others new to social care, while many had changing health conditions.”* (N. Campbell et al., 2011)

*“There are a range of experiences for people whilst an inpatient – some feel it necessary, others hate it so clearly need a range of crisis options to respond to the range of experiences.”* (Bola et al., 2014)

###### Staff scepticism

The final challenge related to staff scepticism about individualized funding. Some of these skepticisms relate to fears and other factors (previously discussed) but staff members were also concerned about process issues such as ‘governance, ‘calculation of allocation’, ‘assessment’ (particularly ‘self‐assessment’), ‘inter‐personal relationships’ and other ‘HR’ issues. Such skepticism would likely impact on the delivery of services, with some participants reporting a lack of knowledge, engagement and commitment from some staff members.

*“I just thought it was another fashionable thing that's coming in and then it'll all be finished by… when something else replaces it, to be honest”* (Eost‐Telling, 2010)

## 7 DISCUSSION

### 7.1 Summary of main results

The present study involved a mixed methods review which identified 4 quantitative, 66 qualitative and 3 mixed‐methods studies that met the inclusion criteria. Data pertain to a 24‐year period from 1992 to 2016 and represent the outcomes and/or views and experiences of over 14,000 participants/respondents, including people with disabilities and their carers/family members as well as practitioners/staff. The quantitative studies included 3 randomised, 3 randomly‐selected study samples and 1 non‐randomised study, representing 19 titles in total. The qualitative studies represented 96 titles in total and a range of designs including in‐depth interviews, mixed qualitative methods, case studies, open‐ended survey questions and other methods, such as secondary qualitative analysis.

The complexity of the intervention and inherent methodological limitations may be reflected in the low number of quantitative versus qualitative studies, which represented just 9% of the included studies. Notably, many other descriptive quantitative studies were found, but they were not investigating effectiveness and/or did not meet the inclusion criteria. Furthermore, the very high level of heterogeneity did not allow for a meta‐analysis of these quantitative data – confounded by the fundamental incompatibility of study design. In addition, the risk of bias was either unclear or high in the majority of studies (Figure 2), although the quality of the quantitative research was judged to be fair to good for most studies.

#### 7.1.1 Quantitative findings

In all, 35 measures were used to test the various health and social care outcomes of interest, as outlined in the protocol. Some studies reported multiple measures for the same outcome of interest (e.g., 5 different measures of client satisfaction (Benjamin et al., 2000)). Brown et al. (2007) and Glendinning et al. (2008) reported most of the outcomes of interest – four and five respectively. The remaining five studies only reported between one and three outcomes of interest. Of the 35 measures reported, there was no difference detected between the intervention and control group for 13 (37%), with a further 6 (18%) reporting no difference in (at least) one of the three study sites (Brown et al., 2007;Table 4).

For those that did report statistical differences across the relevant health and social care outcomes reported, most were in favour of the intervention group, with the (partial) exception of cost‐effectiveness and adverse effects.

In terms of primary outcomes of interest, the most consistently positive outcome for the intervention group was ‘client satisfaction; five of the seven studies reported this primary outcome, with all five showing intervention group participants to be significantly more satisfied than their control group counterparts. The four studies that reported on the second primary outcome – Quality of Life) – were evenly divided between ‘no difference detected’ and a significantly positive result for the intervention when compared to the control groups.

In terms of secondary outcomes, one study reported ‘physical functioning’ with no difference detected between groups. Five studies reported adverse effects across a range of outcomes, with no difference detected in two studies and significantly positive results in favour of the intervention group in one study. The remaining two studies (reporting several adverse outcomes and several study sites) were evenly divided between no difference detected and significantly positive results in favour of the intervention group.

Cost‐effectiveness data (a secondary outcome of interest) were available for only two studies, with no difference detected in one and statistically significant differences in favour of the control group in the other, but only on one of two measures. It should be noted that the study by (Brown et al., 2007), had three study sites and two measurements. One cost‐effectiveness measures favoured the control group across all three sites, while the second measure reflected no difference between intervention and control groups in two of the three sites, with the last site favouring the control group (Table 4).

The penultimate outcome of interest, adverse effects, was presented for five studies. One study favoured the intervention group, while there was no difference between intervention and control groups for two of the five studies. Benjamin et al. (2000) presented two measures of adverse effects, with one showing no difference between groups, while the second favoured the control group – representing one of the 11 measures (used across 5 studies). Brown et al. (2007) presented 6 separate measures with one measure favouring the intervention group across all three study sites. Differences between study sites were seen across all of the remaining five measures, ranging from no difference to favouring the intervention group. None of the adverse effect measures favoured the control group in this study.

Finally, data were available for four ‘other’ relevant health and social care outcomes with no difference detected in three of the four. The remaining outcome – ‘Safety / Sense of security’ – was significantly different in favour of the intervention group.

##### Qualitative findings

The qualitative meta‐synthesis presented the experiences of individuals participating in an individualized funding intervention, as well as documenting implementation successes and challenges from the perspective of multiple stakeholders. The views of over 9,000 people were captured in the 69 studies, 73% from the perspective of individuals with a disability or their representative. As with the quantitative findings, the intervention was positively received overall despite, amongst other things, considerable issues accessing funding, implementation challenges and process delays. Most people reported, even those who were somewhat aggrieved, that they preferred the intervention over traditional service provision.

The improved levels of satisfaction, consistently reported in the quantitative data, are most likely linked to the many perceived benefits which were identified inform the qualitative findings and, in particular, improvements in self‐image and self‐belief. Participants reported feeling more empowered, self‐determined, and confident with an enhanced sense of purpose and freedom. They also reported a sense of control over their lives – self‐directing their supports with an active involvement in decision‐making, identification and procurement of supports and activities.

These perceived improvements, in people's sense of self‐belief and self‐worth, are most likely reflected in the positive changes demonstrated in the quality of life outcomes. Where no differences were detected, it is reasonable to suggest that the many challenges experienced and discussed in the qualitative synthesis, particularly at early implementation stage, may have adversely impacted on perceived quality of life. Participants often reported feeling more burdened with the complexity and level of bureaucracy involved in the new process than in their formerly more passive role in traditional services. This was most prevalent in the early stages of implementation with perceptions generally improving over time and once people had settled into their new way of life. This suggests, from a research perspective, that 6 months is not an appropriate follow‐up time point for assessment in the sense that there may not be sufficient time for the intervention to be put in place and to bed down appropriately. It is interesting to note that Brown et al. (2007), who conducted a large‐scale, high quality, relatively ‘low risk’ study, collected data 9 months after baseline, and found highly significant differences in favour of the intervention group.

Regardless of duration between baseline and follow‐up, the implementation challenges associated with the overly complex systems – seemingly framed around existing assessment, review, governance, and financial arrangements – continued to present problems over the 25 year period covered by this review. Therefore, perhaps it was these systemic issues that negatively impacted participants’ quality of life over longer periods of time. Importantly, the qualitative findings emphasize a need to simplify processes, predicated on respectful, inclusive and trust‐based working relationships, rather than the perceived authoritarian dynamic, whereby informal unpaid carers reportedly feel there is an assumption that they will provide unconditional ‘free’ support, for fear of losing (the highly prized) individualized funding and attendant supports or because no alternative exists. Participants often perceived staff as focusing too narrowly on finances and costs, rather than on the quality of supports provided. Participants also felt that the review process was inequitable and one‐sided, whereby very high standards of reporting and transparency was expected from end users, but unresponsive, delayed and poor quality support was perceived to reflect the funding bodies and providers. While these examples may not be true for the majority of cases, such perceptions fed into the tension and conflicts that sometimes seriously challenged the success of the intervention. The lack of clarity and lack of information as well as inconsistent approaches were all compounding factors and indeed, these were most commonly reported challenge/complaint across all studies. Thus, the provision of timely, accessible and transparent information is a priority.

Unfortunately, there was extensive evidence of disabling practices and attitudes among some funding bodies and support agencies. Staff members were often fearful of misuse of funds or other fraudulent activities by individuals with a disability or their network of support. Staff often perceived people with a disability to be vulnerable to these kinds of situations and they tended, therefore, to be very risk averse in order to safeguard their clients. Interestingly, only one quantitative study reported on client safety and a significant difference was found in favour of the intervention group. This finding strengthens the reported qualitative experience that staff fears were generally alleviated with regard to safeguarding and risk when the intervention was implemented successfully, with strong networks of paid and/or unpaid support in place (Coyle, 2009; Dimitriadis, Laurie, Lane, & Lyall, 2007; Olmstead, 1999; Phillips, Mahoney, & Foster, 2006; Witcher et al., 2000). In fact, the intervention group generally experienced significantly fewer adverse outcomes when compared to their control group counterparts, including unmet needs, with the exception of one study (representing 1 out of 11 adverse measures collected across 5 studies). This is not to say that risk and safety concerns were absent from the qualitative data, with some instances of conflict and abuse reported, although this was far from the predominant experience.

Finally in terms of value for money, many studies descriptively reported the costs of delivering individualized packages of support, but only two looked at the more important question of cost‐effectiveness, with a third conducting a cost‐benefit analysis. Based on the available data, the evidence of cost‐effectiveness was inconclusive. Glendinning et al. (2008) found no difference, while in the ‘Cash and Counseling’ study, one measure of cost‐effectiveness was seen to favour the control group while the other measure was inconsistent between study sites (with two of the three sites showing no difference;Brown et al., 2007). Woolham & Benton (2013) found costs to be considerably higher for the intervention group, but the attendant cost‐benefit analysis also showed the control group to be experiencing ‘some degree of ill‐being’ when compared to the intervention group (Woolham & Benton, 2013).

As outlined earlier in this review, early studies have shown individualized funding to result in cost savings (Conroy, Fullerton, et al., 2002; Zarb & Nadash, 1994) or cost neutrality (Stainton et al., 2009). This cost neutrality is consistent with more recent findings from Canada and New Zealand, where costs were found to be generally lower or on par with traditional methods (Field, 2015; Stainton, Asgarova, & Feduck, 2013) and cost neutral – as far as the level of care and support package is concerned (K. Jones et al., 2012). While Woolham & Benton (2013), in this review, tentatively suggest better well‐being for the intervention group, Stainton et al. (2009) suggest that certain modes of delivery (such as microboards) may in fact offer equal or better value for money when other considerations such as building social capital, ongoing network support and ability to support persons with complex support needs, are taken into consideration.

In line with this thinking, the qualitative data also support the concept that individualized funding offers value for money, both financially and in terms of opportunity. Participants reported the ability to ‘shop around’ in order to find the best value for money. Perhaps more importantly, however, the qualitative data also revealed that people placed equal, if not more, importance on the value to purchase services from within mainstream, community based settings, in turn, increasing community integration and attendant experiences and opportunities.

Furthermore, the qualitative findings showed that staff and organizations were often surprised by the modest requests for funding from people with a disability, perhaps because such individuals reportedly, did not wish to be a burden on the system or to potentially use funding that would be more beneficial to somebody else. This burden and guilt, sometimes reported from recipients of individualized funding, could potentially be avoided if a universal, robust and equitable resource allocation system was in place, whereby every individual is assessed on the same basis, rather than subjective and informal assessment processes often described in the findings reported here.

It is also important, when considering the issue of cost‐effectiveness, to take into account the possible longer term benefits or cost savings of individualized funding such as ‘Quality Adjusted Life Years’ or ‘Disability Adjusted Life Years’. While these longitudinal data are not currently available, the benefits reported from our qualitative findings, in terms of for example, perceived health improvements, greater self‐reliance and more independent living arrangements, would tentatively suggest that quality of life, mental health, wellbeing and other health and social care outcomes improve for service users as a result of individualized funding. If this is indeed the case, resource use within the formal healthcare system may be substantially reduced. An urgent need for more economic evaluations is indicated.

### 7.2 Overall completeness and applicability of evidence

The very broad search strategy adopted for this review (as described in Appendix 2) outlines the totality and breadth of the evidence presented. The large proportion of grey literature (*n* = 42 studies, 55%), in particular, highlights the amount of government – funded and organisation‐commissioned research that has been conducted during the 25 year‐period. The exclusion of these data would have compromised the completeness and applicability of the review and especially given the strong implementation focus adopted throughout, with organization‐commissioned research often prioritizing implementation. Having said that, the considerable list of excluded studies (Appendix 5) which, albeit did not meet our eligibility criteria, highlights the very strong interest in, and increasing awareness of the importance of, individualized funding across the world.

Only sevenstudies, with eligible quantitative data, were identified to address the first aim of the review – to assess the effectiveness of the intervention across a range of primary and secondary outcomes. As indicated earlier, the fundamental incongruity of the studies and other analytical limitations precluded the possibility of undertaking any kind of sub‐group analysis. However, this was balanced by the very rich and abundant qualitative data (69 studies) which represents a very large group of >9,000 intervention participants and provide important and useful insights into the particular contexts and mechanisms under which individualized funding is more (or less) successful and the factors that impact implementation. Importantly, these findings are based on the experiences of a very wide range of stakeholders including individuals with a disability, their representatives/advocates and support workers, funders and organizational staff/representatives.

### 7.3 Limitations and potential biases in the review process

The published protocol was closely followed. However, given the unexpected scale of the review and the complex nature of study designs, a number of changes were required as outlined in 5.3.5. For example, a ‘results refinement’ process had to be developed to deal with the unmanageable number of search results and to filter the studies in a robust, transparent and replicable manner. The changes to protocol, that may introduce bias, include the fact that, due to the huge number of studies involved, only one reviewer conducted the detailed quality assessment, although double screening of full texts did involve a degree of quality screening, as outlined previously. Qualitative coding was also conducted by only one reviewer, although emerging key themes were discussed with a second reviewer, with unexpected themes explored and discussed in detail.

Another change, that may introduce bias, related to the tightening of eligibility in terms of population. Older adults (>65) without evidence of a life‐long disability were excluded (e.g., age‐related frailty vs. life‐long disability). This was implemented to ensure that the population of interest, those with a disability, was appropriately represented in the evidence presented. However, there is a possibility, that by removing older people, there may have been older people, with a life‐long disability who were inadvertently excluded, due to insufficient data to assess their disability status. However, every effort was made to include older adults who did report a life‐long disability or another eligible disability, such as dementia.

As with both previous reviews (Carter Anand et al., 2012; Webber et al., 2014), the evidence presented in this review is limited methodologically with the subsequent impact on quality and risk of bias clearly reported. However, having reviewed the extensive body of literature, we would argue that such limitations are inherent in complex social interventions (as discussed previously), and, as such, these limitations provide useful implementation insights, and a depth of understanding that directly impact on future policy development and future research in this area. Furthermore, it should be reiterated that the evidence in the current study was subjected to a more thorough screening process than in the two previous reviews, with more robust inclusion criteria utilized around methodological design and rigour.

### 7.4 Agreements and disagreements with other studies or reviews

As outlined in the protocol, the authors were aware of only two previous systematic reviews prior to commencing this study (Carter Anand et al., 2012; Webber et al., 2014). In one sense, the eligibility criteria within the current study were broader and more inclusive; for example, Webber et al. limited their review to mental health users only. The need for a results refinement process (Appendix 2) further highlights the broad scope of the current review. In another sense, however, this review was more restrictive in terms of the quality of evidence. To this end, quantitative studies were excluded if they were not designed to robustly evaluate effectiveness or did not have a control group, while previous reviews included studies without control groups (for example). Therefore, the studies included in this review are very different, in some respects from those captured in the above reviews.

At the same time, however, the findings from this review were consistent in many respects with the two reviews previously identified. For example, Carter Anand et al. (2012) concluded that: participants were positive about the experience of individualized funding; collaborative relationships between government, providers, users and carers are integral to the success of individualized funding; resource allocation models are essential and require government involvement and leadership, and that objective needs‐based assessments should be used to determine individual budgets. Periods of transition also need to be carefully planned with supports established to empower a change in practice among existing services providers. Advisor, management and support‐broker services should be widely available for those who require them. A person‐centred approach should be at the centre of the design and delivery of individualized funding and people with a disability should be empowered and supported in the decision‐making processes with appropriate safeguards in place to manage risk and promote safety (Carter Anand et al., 2012).

While safeguarding is always important when working with vulnerable groups, the evidence from this review would caution against over‐emphasizing this area. Staff and the wider network of support for the person with a disability, can inadvertently have a disabling effect, potentially inhibiting the community integration and fulfilment of personal potential. An over emphasis on safeguarding also carries the risk of people ‘falling back on the system’, when inherent implementation challenges present themselves, rather than focusing on the facilitators of successful implementation, such as building a strong and supportive network of support, and training advocates to help individuals with a disability navigate the new, independent, self‐reliant path. As reiterated throughout the review, every situation is different and some people will have higher support needs than others, but the starting point should be one of trust, enablement and empowerment, fully exploring the most self‐determined path and subsequently ensuring necessary supports are in place either temporarily or permanently.

There were also a number of similarities with the review by Webber et al. (2014) and especially where similar studies appeared in both reviews. For instance, perceived benefits were reported in relation to choice and control, flexibility, improved satisfaction, quality of life, greater independence, empowerment, confidence among other personal, health and social care outcomes. Conversely, one study in the Webber et al. review found individualized funding to be cost‐effective (Forder et al., 2012) but that study did not meet the eligibility criteria for this review because only 26% of the study population had a disability/mental health problem.

A considerable number of additional literature reviews were excluded when screening titles and abstracts (Harkes et al., 2014), or when screening full text (which led to the exclusion of five reviews). Harkes et al.'s systematic review focussed on published evidence and intellectual disabilities only and as such, the review was more limited in scope. However, the recommendations were consistent with the findings reported here, including the need for more accessible information, the need for staff training, more local support organizations and the streamlining of funding streams. The authors also highlighted the problematic reluctance amongst practitioners to promote individualized funding. None of the remaining studies identified in the screening process were systematic reviews although, importantly, the references contained therein, informed the hand‐searching for this study.

## 8 AUTHORS’ CONCLUSIONS

### 8.1 Implications for practice and policy

Previous reviews have concluded that there is little evidence to suggest that governments in the past (e.g., in the UK) had clear strategies underpinning the implementation of individualized funding (Harkes et al., 2014). However, recent years have seen a considerable and growing interest in individualized funding as a means to improve the lived experience of people with a disability and their wider network of support (paid and unpaid). As highlighted throughout this review, the availability of robust casual evidence is limited and therefore conclusions must be interpreted with caution. However, this review provides a comprehensive synthesis of available evidence to help inform the decision making of governments, funders and policy makers, whilst also providing researchers in the field with useful information and recommendations for future research.

#### 8.1.1 Practitioners – shift the focus!

This review presented evidence that some of those delivering health and social services, for people with a disability, may be skeptical about individualized funding due to personal concerns around their occupational role (e.g., job loss) and for those they serve (e.g., safeguarding, risk aversion). Furthermore, organizations responsible for delivering services sometimes perceive individualized funding as a top‐down, Government led cost‐cutting measure. All three of these notions, amongst many other misconceptions presented in this review, (e.g., misuse of funds, recipients flooding the system), are not grounded in evidence. In fact, the limited cost‐effectiveness data are inconclusive. The findings of this review suggest that decision makers and those on the front line of implementing individualized funding, might need to shift their focus from one of resistance and skepticism, to one of openness and enthusiasm.

Many services sell themselves as ‘person‐centred’, in line with international best practice. If that is the case, the overwhelmingly positive response in terms of client satisfaction, both quantitatively and qualitatively, should inform practitioner responses and positively influence their attitudes toward individualized funding. In terms of quantitative outcomes, with the exception of one adverse measure, all the evidence points to no difference or improvements – based on the use of individualized funding. While outcomes data are limited to just seven studies, the concerns associated with safeguarding and risk aversion are, by and large, unfounded. This is, of course, a reflection of the hard work, in terms planning and delivery, from both paid and unpaid supports. Practitioners should therefore trust in their ability to engage with the end user and their network of support to safely deliver services, through this new mode of funding and, in turn, provide better quality and highly valued services.

Finally, in terms of job losses, there is a need to shift the focus to one of potential opportunity. This review highlights that one of the most substantial implementation challenges was the lack of available support. This is not consistent with the notion that those working within the health and social care services may lose their jobs as a result of individualized funding. Whilst it is possible that the job descriptions, in terms of day‐to‐day tasks, may change, ultimately this may lead to better job satisfaction, since inter‐personal and working relationships were seen to improve as a result of individualized funding. Whilst the evidence from this review is overwhelmingly positive, more research is needed to assess the impact of individualized funding on workplace relations. The reported challenges generally arose from attempts to embed the new mode of service delivery into traditional systems, thereby leading to unnecessary bureaucracy, stress, anxiety and burden for those delivering and receiving services. As such, this review also suggests that an overhaul is required in terms of governance, and the associated assessment, monitoring and review processes that were traditionally used, but which may no longer be fit for purpose within the individualized funding model of service delivery.

#### 8.1.2 Training

One area requiring further investment is education and training across the board. Practitioners need to acquire or improve upon their skills in order to fully realize the potential of individualized funding. Firstly, more education is required outlining the background and philosophy of individualized funding. This review highlighted that those with a better understanding of individualized funding were highly valued by end users. It instilled confidence in those receiving services, but those practitioners also acted as a valuable source of information and guidance. Unfortunately, however, many practitioners did not fully understand individualized funding, or the implementation plan (if any existed). This in turn, led to inconsistent approaches, mixed messages and misinformation – aspects which caused distress and frustration for those in receipt of services. If those implementing individualized funding are well informed, then a ‘trickle‐down’ effect should ensure consistent messages to end users and their representatives.

While such education may be delivered for end‐users, training is also required for the informal support network, in order to move from a paternalistic to empowering relationship. This move is challenging, as highlighted in this review, often causing tension and conflict, but with the right ‘behaviour change’ training, family and friends may learn to adjust their learned behaviour, to one that is more enabling, trusting and equitable. Finally, this behaviour change is also required for individuals with a disability, who sometimes require guidance in moving from a passive role to one of self‐reliance and self‐direction. The findings of this review indicated that simply moving to individualized funding encouraged such behaviour change, but in other circumstances, a prolonged history or institutionalization warranted more directive action.

Lastly, as highlighted throughout this review, the network of support is integral to success. As part of this, paid supporters need to have the communication and facilitation skills to guide, for example, the journey of discovery, whereby a person (perhaps for the first time) explores what they want to achieve in the short and longer term, and the steps that are required to achieve those goals. Developing a plan, detailed enough to allow progression, but flexible enough to respond to changing (physical and health) needs or personal preferences, is also something that requires training and experience.

#### 8.1.3 Financing individualized funding

The changing economic and social landscapes, in recent times, amongst a number of countries throughout the world with many years’ experience of implementing individualized funding (e.g., Scotland and England) – has meant that the delivery of such supports has had to be amended and adjusted. These changes reflect how the 2008–2013 recession adversely affected health and social care spending, with European countries such as Greece, Ireland, Spain and Portugal (arguably some of the hardest hit European countries of the recent global financial crisis) having seen substantial cuts in these areas (Pearson & Ridley, 2016). With many European countries still feeling the effects of the recent financial crisis, the Irish government, for example, is expectant that plans to implement individualized funding can be framed within a cost‐neutral paradigm (Department of Health, 2016).

However, policy makers, in countries planning initial implementation of individualized funding, need to be cognizant of the inevitable set‐up and transitionary period, whereby the whole sector shifts their thinking and practical approach to delivering services. As outlined above, this requires, amongst other things, significant investment in training. Furthermore, there will be costs associated with changing the traditional governance, monitoring, and review systems, an essential step to ensure successful implementation. Indeed, on a more practical level, there will be a period of time when a person may be availing of traditional services, while trialling new supports, often within the mainstream, community setting – perhaps requiring dual‐funding. It is inevitable that additional set‐up costs will be required. If cost neutrality however, continues to be a driving force, then policy needs to be in place to release funds from ‘block funding’, thereby providing the flexibility to part fund traditional services while also part funding new and emerging sources of support.

Indeed, those countries which are striving to improve the delivery of individualised funding are not limited to economic casualties of the recession; others with little austerity – having avoided the 2008–2013 recession – are also striving to improve the delivery of individualized funding, such as efforts under the new National Disability Insurance Scheme (NDIS) in Australia (Reddihough, Meehan, Stott, Delacy, & Australian Cerebral Palsy, R., Australian Cerebral Palsy Register, G., & the Australian Cerebral Palsy Register, G., 2016). Policy makers can look to such countries, that are utilizing a social insurance scheme, for guidance into the future, but this review would suggest that vast amounts of money are being spent on services with which many people are dissatisfied and simply do not want to use. Arguably therefore, the first step could involve an overhaul of current systems, including the allocation of funding. As such, service providers should be included in this process, encouraged to develop business plans that outline the necessary steps to transition from traditional service delivery to one that embodies the philosophy and ethos of individualized funding.

#### 8.1.4 Final thoughts

Regardless of the intention (or evidence base for effectiveness), it seems that individualized funding is consistently being adopted and supported globally as shown by the overwhelmingly positive response amongst individuals with a disability and their representatives, highlighted in this review. It is also seen as a mechanism that helps achieve the goals outlined in the United Nations Convention on the Rights of People with Disabilities. This review provides an important and comprehensive resource and robust evidence base for policy makers and funders wishing to make informed decisions around the implementation of individualized funding. It presents the most robust effectiveness data currently available, whilst also specifically highlighting the all‐important implementation successes and challenges. The latter can directly impact planning and cost‐effectiveness. Indeed, such cost factors are important in highlighting successful aspects worthy of investment whilst also demonstrating potential (and costly) pitfalls that can be avoided with prudent planning and careful consideration.

### 8.2 Implications for research

This review clearly highlights and synthesizes the extensive and rich qualitative evidence from studies conducted in many countries – across changing social, political, economic, social care, and healthcare landscapes – and over a considerable period of time. It also points to the inherent difficulties associated with collecting quantitative data on complex social interventions of this nature, with a subsequent lack of robust effectiveness data. As a result, the authors suggest the need for more methodologically rigorous evaluation studies ideally forming an integral element of any implementation plan for countries considering the piloting or national roll‐out of individualized funding. The authors also suggest the use of more appropriate methods for real world evaluations of complex interventions within complex systems, such as realist evaluation (Pawson & Tilley, 1997).

The time frame for evaluating complex social interventions should be carefully considered. Six months was the minimum follow‐up period for studies included in this review, with some (excluded) studies collecting data before the six‐month period had lapsed. The qualitative meta‐synthesis underscored the significant challenges experienced during early implementation, and a perception that a true sense of benefits, challenges, processes, procedures and inter‐personal relationships only emerged after sufficient time had passed. Therefore, future researchers should consider (resources permitting) conducting studies which incorporate longer follow‐ups (minimum 9 months), and ideally at multiple time‐points over a longer period of time. Due to ethical considerations, and the individualized, needs‐led nature of the intervention in question, methodological limitations, such as potential loss/attrition at follow‐up, are unavoidable. However, as Glendenning et al. and Brown et al. have effectively demonstrated, the use of large randomized samples goes some way toward addressing this issue.

This review highlights that the evidence on cost effectiveness is inconclusive (as is arguably the case for many social care interventions) and any perceptions that individualized funding is more expensive (or cost efficient) are not grounded in evidence. Indeed, this review also highlights the fact that robust financial data are often not available at national or local level. Researchers need to work closely with policy makers and practitioners to outline the type, level and depth of data required to conduct an in‐depth cost‐effectiveness analysis. In fact, considerable thought needs to be given to all evaluative data required, considering ways to avoid duplication of effort. Such collaborative relationships need to be developed in the early planning stages, well before initial implementation has commenced.

Mixed methods designs are also recommended for future research in the field of individualized funding (and social care interventions more generally). The (limited) quantitative data presented in this review, if considered on a stand‐alone basis – would potentially cast doubt on the continued promotion and implementation of individualized funding, notwithstanding the considerable methodological limitations of the studies in question. By contrast, the qualitative findings provide a useful insight into when, how and for whom the intervention works and the many challenges/pitfalls. For example those with an intellectual disability or mental health problem, often need more input from brokerage/facilitation or intermediary supports, particularly at initial set‐up stage.

However, there is an urgent need for more effectiveness studies and perhaps more standardized approaches to data collection to ensure better comparability across studies and countries. The development of the ASCOT scale (PSSRU, 2014) is a good example of such standardization and not least given the relative lack of reliable and validated measures with which to assess outcomes (as indicated by the disparity between measures used in studies included in this review). At the same time however, it is important that researchers feel able to respond appropriately to country‐specific contextual factors and issue of national interest without an over‐emphasis on global comparisons. In direct response to these contextual factors, the majority of studies, within this review, adopted a methodologically tailored approach. This inevitably meant that a meta‐analysis was not possible, but valuable data was still available to inform future policy and practice. As such, robust data, even if very localized and context‐specific, are better than poor quality data or no data at all.

Finally, the authors of this review would encourage the adoption of mixed‐methods approaches in further systematic reviews when assessing the effectiveness of complex ‘real‐world’ interventions in the field of health and social care. Our experience indicates that mixed‐methods reviews are certainly more complex and time consuming than more traditional approaches. However, the rewards are considerable, not only in terms of providing a more thorough synthesis of available evidence which takes into account the experiences and views of potentially many more participants, but also offering a wealth of detail and useful insights to improve our knowledge and understanding around important health and social care issues across the world.

## 9 DATA AND ANALYSES

### 9.1 Quantitative analysis

See Table 6 and Figures 8, 9, 10, 11, 12, 13, 14, 15, 16, 17, 18, 19, 20, 21, 22, 23, 24, 25.

### 9.2 Qualitative analysis

#### 9.2.1 Overarching (Macro) theme 1: Implementation facilitators

##### Perceived benefit (n = 3,295)

The subtheme ‘perceived benefit’ was a ‘level 2’ macro code (with 75 subordinate themes) but it was also an independent code representing 662 pieces of coded text. Initially, a code co‐occurrence MAXMap was produced for codes that co‐occurred 60 times or more (Figure 5), but this was reduced to 50 to produce more detail. As shown in Figure 26, the perceived benefits, from the perspective of the individual with a disability or their representative (green codes), were ‘flexibility’, ‘community integration’, the freedom to choose ‘who supports you’, and ‘social opportunities’. Sensitivity analysis resulted in no change to the co‐occurring codes.

###### Flexibility

Flexibility was a meso (level 3) code with no specific sub‐codes, representing 177 pieces of coded text (row 316, Appendix 10). Figure 27 demonstrates co‐occurring themes. Sensitivity analysis resulted in ‘choice and control’ co‐occurring marginally less often with ‘perceived benefit’, when studies rated with a ‘very low’ CerQual score were excluded.

###### Freedom

‘Freedom’, a level 3 (meso) theme, represented 773 (23%) coded pieces of text (rows 393–408, Appendix 10). Personal freedom (level 4) was a key sub‐theme pertaining to ‘perceived autonomy’, ‘self‐determination’, ‘self‐direction’, ‘self‐reliance’, ‘sense of empowerment’, ‘space and freedom’ and ‘freedom to make mistakes’ (level 5 themes;rows 402–407). Another key sub‐theme was ‘Freedom to choose / individualization (level 4), pertaining to ‘who supports you’, as well as, ‘how’, ‘when’ and ‘where’ the support is provided (level 5 themes) (rows 394–400).

###### Community integration

Community integration was a meso (level 3) subtheme, with no particular subthemes, representing 151 pieces of coded text (row 383, Appendix 10). Figure 28 demonstrates co‐occurring themes. Sensitivity analysis resulted in no change.

###### Agency involvement

‘Agency involvement’ was a meso (level 3) subtheme, with six subthemes, representing 807 pieces of coded text (rows 543–549, Appendix 10). Figure 29 demonstrates co‐occurring themes. Sensitivity analysis resulted in no change with regard to implementation facilitators.

##### Mechanisms of success (n = 2,702)

The second subtheme here – ‘mechanisms of success’ ‐ represented 87 subthemes (62 meso and 25 micro). Most of the themes within this category came under the meso (level 3) theme – ‘relationships’ (*n* = 930, 34%).

###### Relationships

####### Network of support

‘Network of support’ was a meso (level 4) subtheme. It was the most frequent subordinate code, representing 306 pieces of coded text (row 362, Appendix 10). Figure 30 demonstrates co‐occurring themes. Sensitivity analysis resulted in no change.

####### Financial recognition for voluntary work

‘Financial recognition for voluntary work’ was a meso (level 4) subtheme, with no particular subthemes, representing 41 pieces of coded text. Figure 32 demonstrates co‐occurring themes.

####### Trust

‘Trust’ was a meso (level 4) subtheme, with no particular subthemes, representing 82 pieces of coded text. Figure 30 demonstrates co‐occurring themes. Sensitivity analysis resulted in no change.

Finally, other important (albeit less frequently cited) ‘relationship’ subthemes were: ‘active listening skills’ or the person with a disability feeling ‘heard’; ‘moral support’; ‘dignity and respect”’; ‘use of humour’; ‘shifting the focus from negative to positive’; ‘managing expectations’; and ‘strong leadership’.

##### Implementation facilitators from staff/organisational perspectives (*n* = 292)

‘*Implementation facilitators from staff/organisational perspectives*’ was a macro (level 2) theme, with no particular subthemes, representing 292 pieces of coded text. Figure 33 demonstrates co‐occurring themes. Sensitivity analysis resulted in no change.

###### Local support organizations

Local support organisations was a meso (level 3) theme, representing 161 pieces of coded text (rows 538–539, Appendix 10). Figure 34 demonstrates co‐occurring themes. Sensitivity analysis resulted in no change.

‘Assessment’ was a ‘level 3’ meso code, itself with 4 subordinate codes, representing 171 pieces of coded text (rows 505–509, Appendix 10). Figure 35 demonstrates co‐occurring themes. Sensitivity analysis resulted in no change.

‘Training’ was a ‘level 3’ meso code, representing 137 pieces of coded text (rows 47–48, Appendix 10). Figure 36 demonstrates co‐occurring themes. Sensitivity analysis revealed that HR was less associated with training, when low quality studies were excluded.

Human Resources’ (HR) was (level 2) process theme, with 18 subordinate themes, representing 691 coded pieces of text (rows 50–67 – Appendix 10). Figure 37 demonstrates co‐occurring themes. Sensitivity analysis resulted in no change.

#### 9.2.2 Overarching (Macro) theme 2: implementation challenges

##### Perceived challenges / negative aspects (*n* = 2,640)

‘Perceived negative or challenging aspects’, from the perspective of individuals with a disability, was an independent macro (level 2) theme representing 820 pieces of coded text and 68 subordinate themes (rows 129–196, Appendix 10). Figure 38 demonstrates co‐occurring themes. Sensitivity analysis resulted in no change.

##### Potential problems/Areas for improvement (*n* =  1,692)

###### Information needs

Information needs was a meso (level 4) theme representing 371 pieces of coded text and 9 subordinate themes (rows 280–287, Appendix 10). Figure 39 demonstrates co‐occurring themes. A sensitivity analysis revealed that ‘information needs’ were associated (marginally) fewer times with ‘agency involvement’, when studies rated with a ‘very low’ CerQual score were excluded.

##### Implementation challenges from perspective of staff/organizational representatives

Implementation challenges from perspective of staff/organizational representatives was a macro (level 2) theme representing 779 pieces of coded text and 24 subordinate themes (rows 103–127, Appendix 10). Figure 40 demonstrates co‐occurring themes. A sensitivity analysis revealed that staff/organizational representatives were marginally less concerned about ‘network of support’, ‘forms/paperwork’ and ‘impact on existing services’ when studies rated with a ‘very low’ CerQual score were excluded.

###### Fear

Fear was a meso (level 3) theme representing 262 pieces of coded text and 15 subordinate themes (rows 105–119, Appendix 10). Figure 41 demonstrates co‐occurring themes. Sensitivity analysis resulted in no change.

#### 9.2.3 Overarching (Macro) themes 3 & 4: ‘Process’ & ‘Contributing factors’

##### Severity/type of disability

**Figure 42:**
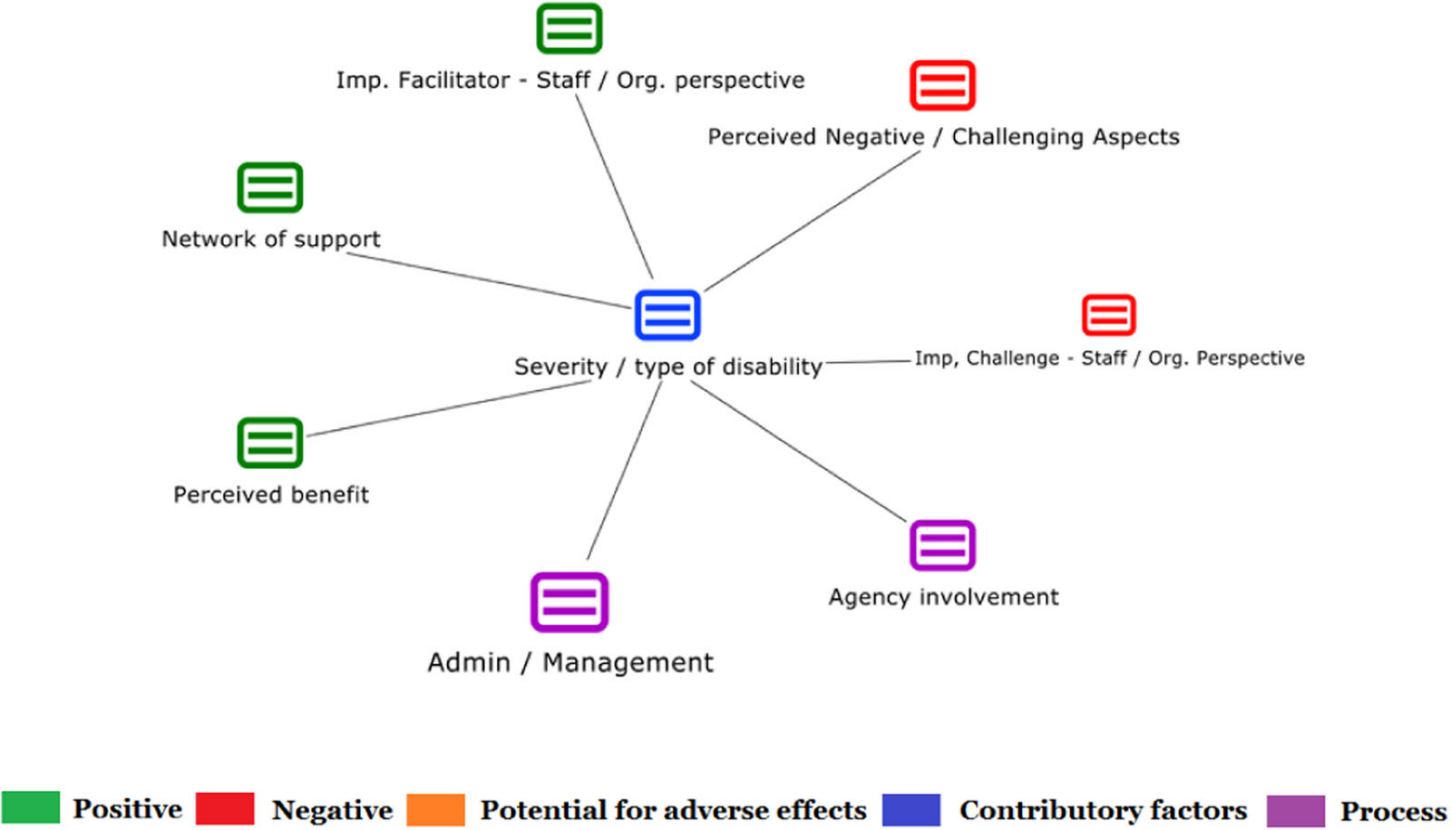
Codes co‐occurring 6 times of more with ‘Severity / type of disability’ [Color figure can be viewed at wileyonlinelibrary.com]

*Severity / type of disability* was a meso (level 3) theme representing 84 pieces of coded text (row 100, Appendix 10). Figure 42 demonstrates co‐occurring themes. Sensitivity analysis resulted in no change.

##### Informal setting

**Figure 43:**
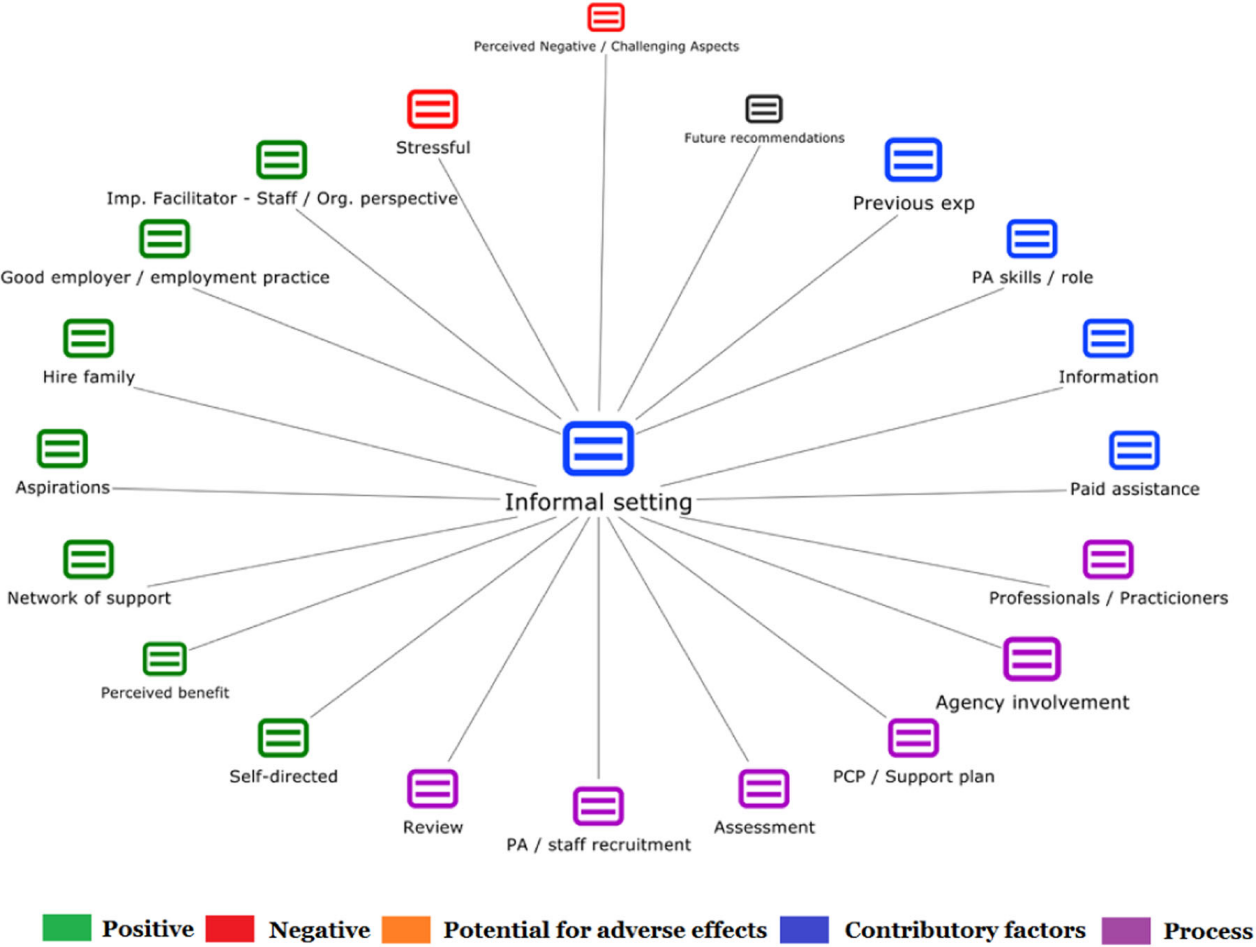
Codes co‐occurring 2 times of more with ‘Informal Setting’ [Color figure can be viewed at wileyonlinelibrary.com]

Informal setting was a meso (level 3) theme representing 33 pieces of coded text (row 80, Appendix 10). Figure 43 demonstrates co‐occurring themes. Sensitivity analysis resulted in no change.

## FUNDING INFORMATION

External funding: The lead author is a fully funded PhD scholar on the “Structured Population and Health‐services Research Education” (SPHeRE) program (SPHeRE, 2015) which is supported by Genio (PHD/2007/16): a non‐profit funding organization whose mission is to develop, test, and scale, cost‐effective ways of supporting people who are disadvantaged to live full lives in their communities; and the Health Research Board: the lead agency in Ireland supporting and funding health research (Genio, 2016; HRB, 2015). Genio funds a wide range of disability, mental health and dementia initiatives in Ireland and also commissions a wide range of research projects in these fields.

## CONFLICT OF INTERESTS

This SR was conducted as part of Padraic Fleming''s (lead author) Ph.D. (Principal Supervisor SMcG). A potential conflict of interest may exist, in that both the lead author (PF) and the individualized funding initiatives that are the subject of his research, are funded by the same agency (i.e., Genio). However, it is important to note that PF is completing his Ph.D as part of a prestigious structured doctoral program in the field of population health/health services research called SPHeRE funded by the Health Research Board in Ireland (www.sphereprogramme.ie). All SPHeRE scholars receive intensive instruction in various methodologies during the course of their first year whilst they are also encouraged to pursue high standards, rigor and objectivity in everything that they do. Furthermore, they are supervised, not only by top health services researchers in the country, but are also supported and guided by an academic panel of senior health services/population health researchers throughout the course of their studies.

Thus, the lead author strived to be as objective and independent as possible and any conflict of interest was disclosed in the reporting of the study. All necessary steps were also taken to avoid any bias that arose in this respect, such as MH independently reviewing the quality of PF and SMcG''s paper that was included in the review. FK was previously the Director of Research and Evidence in Genio. MF, TS, SOD and MH have no conflict of interest.

## AUTHOR CONTRIBUTIONS

**Systematic Review Methods**: PF has participated in Cochrane Collaboration Systematic Review training delivered by the Health Research Board in Dublin and PRISM training workshops (quantitative and qualitative) in Maynooth University Centre for Mental Health and Community Research (www.cmhcr.eu/prism‐training/). PF has extensive research skills required for completing a systematic review including: literature searching, data synthesis and analysis and preparing papers for publication.

SMcG is a co‐author (along with MF) on two published Cochrane/Campbell reviews including: (a) parenting programmes for child conduct problems; and (b) home‐care “reablement” services for improving and maintaining functional independence in older adults. She is also currently involved as a co‐author of four ongoing Cochrane/Campbell reviews (three with MF) on: mindfulness interventions in schools; interventions to improve mathematical outcomes for children with dyscalculia; palliative care interventions; and diabetes care.

MH has previously participated in Cochrane Collaboration Systematic Review training delivered by the Health Research Board in National University of Ireland, Galway. MH has lead or co‐authored a number of published systematic reviews including recently; (a) A systematic review of the costs and effects of self‐management interventions for chronic musculoskeletal pain and (b) A systematic review of measurement tools for adherence to non‐pharmacological self‐management treatment for chronic musculoskeletal conditions; both of which are published in leading international journals in the field, Physical Therapy and Archives of Physical Medicine and Rehabilitation respectively. MH has extensive experience in systematic review methodologies including article screening, data extraction, data synthesis and manuscript preparation.

MF is the lead author or co‐author on five of the Cochrane/Campbell reviews listed above. MF was recently elected as the co‐chair of the Campbell Social Welfare Group. She is also Associate Lecturer with the UK Cochrane Centre and delivers Cochrane training workshops in Ireland. In addition, MF, along with SMcG, are co‐founders and directors of PRISM (Promoting research Innovation in Systematic Reviews and Meta‐analysis), a research and training/teaching hub set up in Maynooth University to develop capacity and expertise in systematic review methodology for professionals and researchers in Ireland (see www.cmhcr.eu/prism‐training/ for further details.

**Statistical Analysis**: Both MF and SMcG have previously been authors on completed systematic reviews using meta‐analytic techniques, whilst other reviews are in progress (as outlined above). They also deliver training workshops on systematic reviewing which include the use of statistical methods in meta‐analyses. All seven researchers are trained in statistical analysis and have attended formal workshops on meta‐analytic techniques.

**Information Retrieval**: All seven researchers are knowledgeable in information retrieval and the lead researcher consulted with a social sciences librarian at Maynooth University. During the protocol stage, we liaised with the information retrieval specialist at Campbell.

### Plans for updating the review

The authors will examine the review every three years for update.

## Supporting information

Supporting information

Supporting information

Supporting information

Supporting information

Supporting information

Supporting information

Supporting information

Supporting information

Supporting information

Supporting information
