# Supplementary material for: Individualized funding interventions to improve health and social care outcomes for people with a disability: A mixed‐methods systematic review
Source: Campbell Syst Rev. 2019 Jul 19;15(1-2):e1008. doi: 10.4073/csr.2019.3 (PMC8356501; doi:10.4073/csr.2019.3)
Supplement: Supplementary file 1 — Supporting information [file CL2-15-e1008-s001.docx]

# Appendix 1: Search Strings for various electronic databases / search engines

| Database | EMBASE (13,216 returns) – Emtree headings and title, abstract | | | | | | | | |  |
| --- | --- | --- | --- | --- | --- | --- | --- | --- | --- | --- |
| Syntax | 'intellectual impairment'/exp OR 'disability'/exp OR handicap OR ((people OR person* OR individ*) NEAR/3 (disabil* OR disable*)):ab,ti OR insanity OR (mental NEAR/1 (instability OR infantilism OR deficiency OR disease OR abnormality OR change OR confusion OR defect* OR disorder* OR disturbance OR illness OR insufficiency)):ab,ti OR (psych* NEAR/1 (disease OR disorder* OR illness OR symptom OR disturbance)):ab,ti AND ('financial management'/exp OR ((budget OR finance* OR fund* OR resource OR money OR income OR purchas* OR broker* OR salary OR capital OR investment OR profit) NEAR/3 (individual* OR person*)):ab,ti) OR 'cash for care':ab,ti OR 'consumer directed care':ab,ti OR 'direct payment':ab,ti OR 'indicative allocation':ab,ti OR 'individual budget':ab,ti OR 'individual service fund':ab,ti OR 'managed account':ab,ti OR 'managed budget':ab,ti OR 'notional budget':ab,ti OR 'personal budget':ab,ti OR 'personal health budget':ab,ti OR personalisation:ab,ti OR 'personalised care':ab,ti OR personalization:ab,ti OR 'person centred':ab,ti OR 'pooled budget':ab,ti OR 'recovery budget':ab,ti OR 'resource allocation system':ab,ti OR 'self-directed assessment':ab,ti OR 'self-directed care':ab,ti OR 'self-directed support':ab,ti OR 'support plan':ab,ti OR 'virtual budget':ab,ti OR 'disability living allowance' OR **'self-determin*':ab,ti** AND [1985-2015]/py **AND [humans]/lim** | | | | | | | | | |
| Database | PsychInfo (12,560 returns) – database heading and title, abstract | | | | | | | |  | |
| Syntax | ( TI ( (((((((((((((((DE "Disability Evaluation") OR (DE "Disability Management")) AND (DE "Syndromes" OR DE "Disabled (Attitudes Toward)" OR DE "Intellectual Development Disorder (Attitudes Toward)" OR DE "Dementia" OR DE "AIDS Dementia Complex" OR DE "Dementia with Lewy Bodies" OR DE "Presenile Dementia" OR DE "Semantic Dementia" OR DE "Senile Dementia" OR DE "Vascular Dementia" OR DE "Mental Illness (Attitudes Toward)" OR DE "Physical Disabilities (Attitudes Toward)" OR DE "Sensory Disabilities (Attitudes Toward)")) OR (DE "Disabilities" OR DE "Developmental Disabilities" OR DE "Learning Disabilities" OR DE "Multiple Disabilities" OR DE "Reading Disabilities")) OR (DE "Disorders" OR DE "Adventitious Disorders" OR DE "Atypical Disorders" OR DE "Behavior Disorders" OR DE "Communication Disorders" OR DE "Congenital Disorders" OR DE "Feeding Disorders" OR DE "Intellectual Development Disorder" OR DE "Learning Disorders" OR DE "Mental Disorders" OR DE "Physical Disorders")) OR (DE "Special Needs")) OR (DE "Disabled (Attitudes Toward)" OR DE "Intellectual Development Disorder (Attitudes Toward)" OR DE "Mental Illness (Attitudes Toward)" OR DE "Physical Disabilities (Attitudes Toward)" OR DE "Sensory Disabilities (Attitudes Toward)")) OR (DE "Brain Disorders" OR DE "Acute Alcoholic Intoxication" OR DE "Anencephaly" OR DE "Aphasia" OR DE "Athetosis" OR DE "Balint's Syndrome" OR DE "Brain Damage" OR DE "Brain Neoplasms" OR DE "Cerebral Palsy" OR DE "Cerebrovascular Accidents" OR DE "Chronic Alcoholic Intoxication" OR DE "Diaschisis" OR DE "Dysexecutive Syndrome" OR DE "Encephalitis" OR DE "Encephalopathies" OR DE "Epilepsy" OR DE "Epileptic Seizures" OR DE "General Paresis" OR DE "Hydrocephalus" OR DE "Intracranial Abscesses" OR DE "Kluver Bucy Syndrome" OR DE "Leukoencephalopathy" OR DE "Microcephaly" OR DE "Organic Brain Syndromes" OR DE "Parkinson's Disease" OR DE "Tay Sachs Disease")) OR (DE "Mental Health" OR DE "Community Mental Health")) OR (DE "Mental Disorders" OR DE "Adjustment Disorders" OR DE "Affective Disorders" OR DE "Alexithymia" OR DE "Anxiety Disorders" OR DE "Autism" OR DE "Chronic Mental Illness" OR DE "Dementia" OR DE "Dissociative Disorders" OR DE "Eating Disorders" OR DE "Elective Mutism" OR DE "Factitious Disorders" OR DE "Gender Identity Disorder" OR DE "Hysteria" OR DE "Impulse Control Disorders" OR DE "Koro" OR DE "Mental Disorders due to General Medical Conditions" OR DE "Neurosis" OR DE "Paraphilias" OR DE "Personality Disorders" OR DE "Pervasive Developmental Disorders" OR DE "Pseudodementia" OR DE "Psychosis" OR DE "Schizoaffective Disorder")) OR (DE "Infantilism")) AND (DE "Costs and Cost Analysis" OR DE "Budgets" OR DE "Health Care Costs")) OR (DE "Finance")) OR (DE "Funding")) OR (DE "Money" OR DE "Resource Allocation" OR DE "Venture Capital")) OR (DE "Health Care Costs") ) OR AB ( (((((((((((((((DE "Disability Evaluation") OR (DE "Disability Management")) AND (DE "Syndromes" OR DE "Disabled (Attitudes Toward)" OR DE "Intellectual Development Disorder (Attitudes Toward)" OR DE "Dementia" OR DE "AIDS Dementia Complex" OR DE "Dementia with Lewy Bodies" OR DE "Presenile Dementia" OR DE "Semantic Dementia" OR DE "Senile Dementia" OR DE "Vascular Dementia" OR DE "Mental Illness (Attitudes Toward)" OR DE "Physical Disabilities (Attitudes Toward)" OR DE "Sensory Disabilities (Attitudes Toward)")) OR (DE "Disabilities" OR DE "Developmental Disabilities" OR DE "Learning Disabilities" OR DE "Multiple Disabilities" OR DE "Reading Disabilities")) OR (DE "Disorders" OR DE "Adventitious Disorders" OR DE "Atypical Disorders" OR DE "Behavior Disorders" OR DE "Communication Disorders" OR DE "Congenital Disorders" OR DE "Feeding Disorders" OR DE "Intellectual Development Disorder" OR DE "Learning Disorders" OR DE "Mental Disorders" OR DE "Physical Disorders")) OR (DE "Special Needs")) OR (DE "Disabled (Attitudes Toward)" OR DE "Intellectual Development Disorder (Attitudes Toward)" OR DE "Mental Illness (Attitudes Toward)" OR DE "Physical Disabilities (Attitudes Toward)" OR DE "Sensory Disabilities (Attitudes Toward)")) OR (DE "Brain Disorders" OR DE "Acute Alcoholic Intoxication" OR DE "Anencephaly" OR DE "Aphasia" OR DE "Athetosis" OR DE "Balint's Syndrome" OR DE "Brain Damage" OR DE "Brain Neoplasms" OR DE "Cerebral Palsy" OR DE "Cerebrovascular Accidents" OR DE "Chronic Alcoholic Intoxication" OR DE "Diaschisis" OR DE "Dysexecutive Syndrome" OR DE "Encephalitis" OR DE "Encephalopathies" OR DE "Epilepsy" OR DE "Epileptic Seizures" OR DE "General Paresis" OR DE "Hydrocephalus" OR DE "Intracranial Abscesses" OR DE "Kluver Bucy Syndrome" OR DE "Leukoencephalopathy" OR DE "Microcephaly" OR DE "Organic Brain Syndromes" OR DE "Parkinson's Disease" OR DE "Tay Sachs Disease")) OR (DE "Mental Health" OR DE "Community Mental Health")) OR (DE "Mental Disorders" OR DE "Adjustment Disorders" OR DE "Affective Disorders" OR DE "Alexithymia" OR DE "Anxiety Disorders" OR DE "Autism" OR DE "Chronic Mental Illness" OR DE "Dementia" OR DE "Dissociative Disorders" OR DE "Eating Disorders" OR DE "Elective Mutism" OR DE "Factitious Disorders" OR DE "Gender Identity Disorder" OR DE "Hysteria" OR DE "Impulse Control Disorders" OR DE "Koro" OR DE "Mental Disorders due to General Medical Conditions" OR DE "Neurosis" OR DE "Paraphilias" OR DE "Personality Disorders" OR DE "Pervasive Developmental Disorders" OR DE "Pseudodementia" OR DE "Psychosis" OR DE "Schizoaffective Disorder")) OR (DE "Infantilism")) AND (DE "Costs and Cost Analysis" OR DE "Budgets" OR DE "Health Care Costs")) OR (DE "Finance")) OR (DE "Funding")) OR (DE "Money" OR DE "Resource Allocation" OR DE "Venture Capital")) OR (DE "Health Care Costs") ) ) AND ( TI ( person* OR individ* OR fund* OR financ* OR cash OR pay* OR broker* OR self-direct* OR “Cash for care" OR "consumer directed care" OR "direct payment" OR "indicative allocation" OR "individual budget" OR "individual service fund" OR "managed account" OR "managed budget" OR "notional budget" OR "personal budget" OR "personal health budget" OR personalisation OR "personalised care" OR personalization OR "person centred" OR "pooled budget" OR "recovery budget" OR "resource allocation system" OR "self-directed assessment" OR "self-directed care" OR "self-directed support" OR "support plan" OR "virtual budget" OR “disability living allowance” OR **"self-determin*"**) OR AB ( person* OR individ* OR fund* OR financ* OR cash OR pay* OR broker* OR self-direct* OR “Cash for care" OR "consumer directed care" OR "direct payment" OR "indicative allocation" OR "individual budget" OR "individual service fund" OR "managed account" OR "managed budget" OR "notional budget" OR "personal budget" OR "personal health budget" OR personalisation OR "personalised care" OR personalization OR "person centred" OR "pooled budget" OR "recovery budget" OR "resource allocation system" OR "self-directed assessment" OR "self-directed care" OR "self-directed support" OR "support plan" OR "virtual budget" OR “disability living allowance” **OR "self-determin*")** ) | | | | | | | | | |
| Database | ASSIA (8,622) – subject heading, title, abstract | | | | | | | |  | |
| Syntax | **(((SU.EXACT("Benefits" OR "Compensation" OR "Minimum Wage" OR "Pensions" OR "Restitution (Corrections)" OR "Salaries" OR "Wages") OR SU.EXACT("Costs" OR "Health Care Costs" OR "Housing Costs" OR "Rents") OR SU.EXACT("Capital") OR SU.EXACT("Foreign Investment" OR "Human Capital" OR "Investment") OR SU.EXACT("Fund Raising") OR SU.EXACT("Income" OR "Profits") OR SU.EXACT("Resource Allocation") OR SU.EXACT("Child Support" OR "Contributions (Donations)" OR "Financial Support" OR "Food Stamps" OR "Grants" OR "Subsidies") OR SU.EXACT("Money") OR SU.EXACT("Finance" OR "Public Finance")) AND (SU.EXACT("Blind" OR "Congenitally Handicapped" OR "Deaf" OR "Handicapped" OR "Mentally Retarded" OR "Physically Handicapped") OR SU.EXACT("Senility") OR SU.EXACT("Alzheimer's Disease") OR SU.EXACT("Community Mental Health" OR "Mental Health") OR SU.EXACT("Acquired Immune Deficiency Syndrome" OR "Alcoholism" OR "Alzheimer's Disease" OR "Anorexia Nervosa" OR "Arthritis" OR "Attention Deficit Disorder" OR "Blood Diseases" OR "Breast Cancer" OR "Bulimia" OR "Cancer" OR "Cerebral Palsy" OR "Depersonalization" OR "Diabetes" OR "Diseases" OR "Disorders" OR "Eating Disorders" OR "Epilepsy" OR "Heart Diseases" OR "Influenza" OR "Language Disorders" OR "Leprosy" OR "Leukemia" OR "Mental Illness" OR "Obesity" OR "Paranoia" OR "Personality Disorders" OR "Physical Abnormalities" OR "Plague" OR "Poliomyelitis" OR "Psychosis" OR "Schizophrenia" OR "Sociopathic Personality" OR "Tuberculosis" OR "Venereal Diseases") OR SU.EXACT("Affective Illness" OR "Depression (Psychology)") OR SU.EXACT("Autism") OR SU.EXACT("Developmental Disabilities") OR SU.EXACT("Learning Disabilities") OR SU.EXACT("Disability Recipients"))) OR (ab(broker* OR self-direct* OR Cash for care OR consumer directed care OR direct payment OR indicative allocation OR individual budget OR individual service fund OR managed account OR managed budget OR notional budget OR personal budget OR personal health budget OR individual?ed fund OR individuali?ed OR personali?ation OR personali?ed care OR person centred OR pooled budget OR recovery budget OR resource allocation system OR self-directed assessment OR self-directed care OR self-directed support OR support plan OR virtual budget OR disability living allowance OR self-determin*) OR ti(broker* OR self-direct* OR Cash for care OR consumer directed care OR direct payment OR indicative allocation OR individual budget OR individual service fund OR managed account OR managed budget OR notional budget OR personal budget OR personal health budget OR individual?ed fund OR individuali?ed OR personali?ation OR personali?ed care OR person centred OR pooled budget OR recovery budget OR resource allocation system OR self-directed assessment OR self-directed care OR self-directed support OR support plan OR virtual budget OR disability living allowance OR self-determin*))) AND pd(19850101-20161231)** | | | | | | | | | |
| Database | Medline First Search (8,800) – mesh heading, title, abstract | | | | | | |  | | |
| Syntax | yr: 1985-2016 and ((mh: Disability and mh: Evaluation) OR mh: Dyslexia OR (mh: Dyslexia, and mh: Acquired) OR (mh: Intellectual and mh: Disability) OR (((mh: International and mh: Classification and mh: of and mh: Functioning, and mh: Disability) and mh: Health) OR (mh: Vision and mh: Disorders) OR (mh: ATR-X and mh: syndrome) OR (((mh: Spastic and mh: Paraplegia and mh: 18, and mh: Autosomal and mh: Recessive) OR (mh: Developmental and mh: Disabilities) OR mh: Epilepsy) OR (mh: Muscular and mh: Diseases) OR (mh: Down and mh: Syndrome) OR (mh: Disabled and mh: Persons) OR ((mh: Health and mh: Services and mh: for and mh: Persons with Disabilities) OR ((mh: Mentally and mh: Disabled and mh: Persons) OR mh: Persons with Hearing and mh: Impairments) OR (mh: Deaf-Blind and mh: Disorders) OR (mh: Mental and mh: Disorders) OR ((mh: Mental and mh: Disorders and mh: Diagnosed and mh: in and mh: Childhood) OR (mh: Mental and mh: Health) OR ((mh: Mental and mh: Retardation, and mh: X-Linked) OR ((mh: Mentally and mh: Ill and mh: Persons) OR ((mh: Delirium, and mh: Dementia, and mh: Amnestic, and mh: Cognitive and mh: Disorders) OR ((mh: Affective and mh: Disorders, and mh: Psychotic) OR ((mh: Abducens and mh: Nerve and mh: Diseases) OR ((mh: Antisocial and mh: Personality and mh: Disorder) OR (mh: Anxiety and mh: Disorders) OR (mh: Anxiety, and mh: Separation) OR mh: Apraxias) OR (mh: Articulation and mh: Disorders) OR (mh: Asperger and mh: Syndrome) OR (mh: Attention and mh: Deficit) and ((mh: Disruptive and mh: Behavior and mh: Disorders) OR ((mh: Attention and mh: Deficit and mh: Disorder with Hyperactivity) OR ((mh: Auditory and mh: Diseases, and mh: Central) OR (mh: Autistic and mh: Disorder) OR (mh: Bipolar and mh: Disorder) OR ((mh: Child and mh: Behavior and mh: Disorders) OR (mh: Communication and mh: Disorders) OR (mh: Deaf-Blind and mh: Disorders) OR (mh: Depressive and mh: Disorder) OR (mh: Learning and mh: Disorders) OR ((mh: Motor and mh: Skills and mh: Disorders) OR (mh: Movement and mh: Disorders) OR (mh: Psychomotor and mh: Disorders) OR (mh: Psychophysiologic and mh: Disorders) OR (mh: Psychotic and mh: Disorders) OR mh: Schizophrenia) or mh: Deafness) or mh: Blindness)))))))))))) or (ti: autis* or ti: disabil* or ti: handicap* or ti: disable* or ti: insan* OR ti: mental* or ti: disorder* or ti: dementia or ti: retard*) or (ab: autis* or ab: disabil* or ab: handicap* or ab: disable* or ab: insan* OR ab: mental* or ab: disorder* or ab: retard*) and (mh: Financial and mh: Management) or (ab: Cash w1 care OR ab: consumer w directed w care OR ab: direct w payment OR ab: indicative w allocation OR ab: individual w budget OR ab: individual w service and ab: fund OR ab: managed w account OR ab: managed w budget OR ab: notional w budget OR ab: personal w budget OR ab: personal w health w budget OR ab: personali?ation OR ab: personali?ed w care OR ab: person w centred OR ab: pooled w budget OR ab: recovery w budget OR ab: resource w allocation w system OR ab: self-directed w assessment OR ab: self-directed w care OR ab: self-directed w support OR ab: support w plan OR ab: virtual w budget OR ab: disability w living w allowance) or (ti: Cash w1 care OR ti: consumer w directed w care OR ti: direct w payment OR ti: indicative w allocation OR ti: individual w budget OR ti: individual w service and ti: fund OR ti: managed w account OR ti: managed w budget OR ti: notional w budget OR ti: personal w budget OR ti: personal w health w budget OR ti: personali?ation OR ti: personali?ed w care OR ti: person w centred OR ti: pooled w budget OR ti: recovery w budget OR ti: resource w allocation w system OR ti: self-directed w assessment OR ti: self-directed w care OR ti: self-directed w support OR ti: support w plan OR ti: virtual w budget OR ti: disability w living w allowance) or (ti: fund* n3 individual* OR ti: budget* n3 individual* OR ti: financ* n3 individual* OR ti: resourc* n3 individual* OR ti: money n3 individual* OR ti: income n3 individual* OR ti: purchas* n3 individual* OR ti: salary n3 individual* OR ti: capital n3 individual* OR ti: invest* n3 individual* OR ti: profit n3 individual*) OR (ti: fund* n3 person* OR ti: budget* n3 person* OR ti: financ* n3 person* OR ti: resourc* n3 person* OR ti: money n3 person* OR ti: income n3 person* OR ti: purchas* n3 person* OR ti: salary n3 person* OR ti: capital n3 person* OR ti: invest* n3 person* OR ti: profit n3 person* **OR ti: self-determin*)** or (ab: fund* w individual* OR ab: budget* w individual* OR ab: financ* w individual* OR ab: resourc* w individual* OR ab: money w individual* OR ab: income w individual* OR ab: purchas* w individual* OR ab: salary w individual* OR ab: capital w individual* OR ab: invest* w individual* OR ab: profit w individual*) OR (ab: fund* w person* OR ab: budget* w person* OR ab: financ* w person* OR ab: resourc* w person* OR ab: money w person* OR ab: income w person* OR ab: purchas* w person* OR ab: salary w person* OR ab: capital w person* OR ab: invest* w person* OR ab: profit w person* **OR ab:** **self-determin*))** | | | | | | | | | |
| Database | SCOPUS (10,994) – title, abstract, keyword | | | | | | | |  | |
| Syntax | TITLE-ABS-KEY ( disabil* OR disabl* OR mental OR disorder OR autis* OR deaf OR blind OR dementia) AND TITLE-ABS-KEY ( budget* OR finance* OR fund* OR broker* OR resource* OR money OR income OR purchas* OR salary OR capital OR investment OR cash OR profit OR "Cash for care" OR "consumer directed care" OR "direct payment" OR "indicative allocation" OR broker* OR "individual budget" OR "individual service fund" OR "managed account" OR "managed budget" OR "notional budget" OR "personal budget" OR "personal health budget" OR personali?ation OR "personali?ed care" OR "person-cent*" OR "pooled budget" OR "recovery budget" OR "resource allocation system" OR "self-directed assessment" OR "self-directed care" OR "self-directed support" OR "support plan" OR "virtual budget" OR "disability living allowance" OR broker* ) AND TITLE-ABS ( individ* OR person* OR self-direct* OR self-deter* OR disabil* OR disabl* OR mental OR disorder OR autis* OR dementia OR deaf OR blind W/4 budget OR finance* OR fund* OR resource OR money OR income OR purchas* OR broker* OR salary OR capital OR investment OR profit ) AND **( EXCLUDE(SUBJAREA,"ENGI" ) OR EXCLUDE(SUBJAREA,"COMP" ) OR EXCLUDE(SUBJAREA,"PHAR" ) OR EXCLUDE(SUBJAREA,"AGRI" ) OR EXCLUDE(SUBJAREA,"IMMU" ) OR EXCLUDE(SUBJAREA,"PHYS" ) OR EXCLUDE(SUBJAREA,"ENER" ) OR EXCLUDE(SUBJAREA,"DENT" ) OR EXCLUDE(SUBJAREA,"EART" ) OR EXCLUDE(SUBJAREA,"CENG" ) OR EXCLUDE(SUBJAREA,"VETE" ) ) AND ( EXCLUDE(SUBJAREA,"BIOC" ) OR EXCLUDE(SUBJAREA,"CHEM" ) ) AND ( EXCLUDE(PUBYEAR,1984) OR EXCLUDE(PUBYEAR,1983) OR EXCLUDE(PUBYEAR,1982) OR EXCLUDE(PUBYEAR,1981) OR EXCLUDE(PUBYEAR,1980) OR EXCLUDE(PUBYEAR,1979) OR EXCLUDE(PUBYEAR,1978) OR EXCLUDE(PUBYEAR,1977) ) AND ( EXCLUDE(PUBYEAR,1976) OR EXCLUDE(PUBYEAR,1975) OR EXCLUDE(PUBYEAR,1974) OR EXCLUDE(PUBYEAR,1973) OR EXCLUDE(PUBYEAR,1972) OR EXCLUDE(PUBYEAR,1971) OR EXCLUDE(PUBYEAR,1970) OR EXCLUDE(PUBYEAR,1969) ) AND ( EXCLUDE(PUBYEAR,1968) OR EXCLUDE(PUBYEAR,1967) OR EXCLUDE(PUBYEAR,1966) OR EXCLUDE(PUBYEAR,1965) OR EXCLUDE(PUBYEAR,1964) OR EXCLUDE(PUBYEAR,1963) OR EXCLUDE(PUBYEAR,1962) OR EXCLUDE(PUBYEAR,1958) OR EXCLUDE(PUBYEAR,1957) OR EXCLUDE(PUBYEAR,1956) OR EXCLUDE(PUBYEAR,1955) OR EXCLUDE(PUBYEAR,1954) OR EXCLUDE(PUBYEAR,1953) OR EXCLUDE(PUBYEAR,1952) OR EXCLUDE(PUBYEAR,1951) OR EXCLUDE(PUBYEAR,1950) OR EXCLUDE(PUBYEAR,1949) OR EXCLUDE(PUBYEAR,1947) OR EXCLUDE(PUBYEAR,1946) OR EXCLUDE(PUBYEAR,1943) OR EXCLUDE(PUBYEAR,1942) OR EXCLUDE(PUBYEAR,1941) OR EXCLUDE(PUBYEAR,1940) OR EXCLUDE(PUBYEAR,1939) OR EXCLUDE(PUBYEAR,1938) OR EXCLUDE(PUBYEAR,1934) OR EXCLUDE(PUBYEAR,1933) OR EXCLUDE(PUBYEAR,1932) OR EXCLUDE(PUBYEAR,1926) OR EXCLUDE(PUBYEAR,1925) OR EXCLUDE(PUBYEAR,1924) OR EXCLUDE(PUBYEAR,1923) OR EXCLUDE(PUBYEAR,1922) OR EXCLUDE(PUBYEAR,1915) OR EXCLUDE(PUBYEAR,1914) OR EXCLUDE(PUBYEAR,1912) OR EXCLUDE(PUBYEAR,1909) OR EXCLUDE(PUBYEAR,1906) )** | | | | | | | | | |
| Database | Sociological Abstracts (9,839) -subject heading, title, abstract | | | | | | | | | |
| Syntax | **(((SU.EXACT("Benefits" OR "Compensation" OR "Minimum Wage" OR "Pensions" OR "Restitution (Corrections)" OR "Salaries" OR "Wages") OR SU.EXACT("Costs" OR "Health Care Costs" OR "Housing Costs" OR "Rents") OR SU.EXACT("Capital") OR SU.EXACT("Foreign Investment" OR "Human Capital" OR "Investment") OR SU.EXACT("Fund Raising") OR SU.EXACT("Income" OR "Profits") OR SU.EXACT("Resource Allocation") OR SU.EXACT("Child Support" OR "Contributions (Donations)" OR "Financial Support" OR "Food Stamps" OR "Grants" OR "Subsidies") OR SU.EXACT("Money") OR SU.EXACT("Finance" OR "Public Finance")) AND (SU.EXACT("Blind" OR "Congenitally Handicapped" OR "Deaf" OR "Handicapped" OR "Mentally Retarded" OR "Physically Handicapped") OR SU.EXACT("Senility") OR SU.EXACT("Alzheimer's Disease") OR SU.EXACT("Community Mental Health" OR "Mental Health") OR SU.EXACT("Acquired Immune Deficiency Syndrome" OR "Alcoholism" OR "Alzheimer's Disease" OR "Anorexia Nervosa" OR "Arthritis" OR "Attention Deficit Disorder" OR "Blood Diseases" OR "Breast Cancer" OR "Bulimia" OR "Cancer" OR "Cerebral Palsy" OR "Depersonalization" OR "Diabetes" OR "Diseases" OR "Disorders" OR "Eating Disorders" OR "Epilepsy" OR "Heart Diseases" OR "Influenza" OR "Language Disorders" OR "Leprosy" OR "Leukemia" OR "Mental Illness" OR "Obesity" OR "Paranoia" OR "Personality Disorders" OR "Physical Abnormalities" OR "Plague" OR "Poliomyelitis" OR "Psychosis" OR "Schizophrenia" OR "Sociopathic Personality" OR "Tuberculosis" OR "Venereal Diseases") OR SU.EXACT("Affective Illness" OR "Depression (Psychology)") OR SU.EXACT("Autism") OR SU.EXACT("Developmental Disabilities") OR SU.EXACT("Learning Disabilities") OR SU.EXACT("Disability Recipients"))) OR (ab(broker* OR self-direct* OR Cash for care OR consumer directed care OR direct payment OR indicative allocation OR individual budget OR individual service fund OR managed account OR managed budget OR notional budget OR personal budget OR personal health budget OR individual?ed fund OR individuali?ed OR personali?ation OR personali?ed care OR person centred OR pooled budget OR recovery budget OR resource allocation system OR self-directed assessment OR self-directed care OR self-directed support OR support plan OR virtual budget OR disability living allowance OR self-determin*) OR ti(broker* OR self-direct* OR Cash for care OR consumer directed care OR direct payment OR indicative allocation OR individual budget OR individual service fund OR managed account OR managed budget OR notional budget OR personal budget OR personal health budget OR individual?ed fund OR individuali?ed OR personali?ation OR personali?ed care OR person centred OR pooled budget OR recovery budget OR resource allocation system OR self-directed assessment OR self-directed care OR self-directed support OR support plan OR virtual budget OR disability living allowance OR self-determin*))) AND pd(19850101-20161231)** | | | | | | | | | |
| Database | Worldwide Political Science Abstracts (6,450) – subject headings, title, abstract | | | | | | | | | |
| Syntax | **(((SU.EXACT("Benefits" OR "Compensation" OR "Minimum Wage" OR "Pensions" OR "Restitution (Corrections)" OR "Salaries" OR "Wages") OR SU.EXACT("Costs" OR "Health Care Costs" OR "Housing Costs" OR "Rents") OR SU.EXACT("Capital") OR SU.EXACT("Foreign Investment" OR "Human Capital" OR "Investment") OR SU.EXACT("Fund Raising") OR SU.EXACT("Income" OR "Profits") OR SU.EXACT("Resource Allocation") OR SU.EXACT("Child Support" OR "Contributions (Donations)" OR "Financial Support" OR "Food Stamps" OR "Grants" OR "Subsidies") OR SU.EXACT("Money") OR SU.EXACT("Finance" OR "Public Finance")) AND (SU.EXACT("Blind" OR "Congenitally Handicapped" OR "Deaf" OR "Handicapped" OR "Mentally Retarded" OR "Physically Handicapped") OR SU.EXACT("Senility") OR SU.EXACT("Alzheimer's Disease") OR SU.EXACT("Community Mental Health" OR "Mental Health") OR SU.EXACT("Acquired Immune Deficiency Syndrome" OR "Alcoholism" OR "Alzheimer's Disease" OR "Anorexia Nervosa" OR "Arthritis" OR "Attention Deficit Disorder" OR "Blood Diseases" OR "Breast Cancer" OR "Bulimia" OR "Cancer" OR "Cerebral Palsy" OR "Depersonalization" OR "Diabetes" OR "Diseases" OR "Disorders" OR "Eating Disorders" OR "Epilepsy" OR "Heart Diseases" OR "Influenza" OR "Language Disorders" OR "Leprosy" OR "Leukemia" OR "Mental Illness" OR "Obesity" OR "Paranoia" OR "Personality Disorders" OR "Physical Abnormalities" OR "Plague" OR "Poliomyelitis" OR "Psychosis" OR "Schizophrenia" OR "Sociopathic Personality" OR "Tuberculosis" OR "Venereal Diseases") OR SU.EXACT("Affective Illness" OR "Depression (Psychology)") OR SU.EXACT("Autism") OR SU.EXACT("Developmental Disabilities") OR SU.EXACT("Learning Disabilities") OR SU.EXACT("Disability Recipients"))) OR (ab(broker* OR self-direct* OR Cash for care OR consumer directed care OR direct payment OR indicative allocation OR individual budget OR individual service fund OR managed account OR managed budget OR notional budget OR personal budget OR personal health budget OR individual?ed fund OR individuali?ed OR personali?ation OR personali?ed care OR person centred OR pooled budget OR recovery budget OR resource allocation system OR self-directed assessment OR self-directed care OR self-directed support OR support plan OR virtual budget OR disability living allowance OR self-determin*) OR ti(broker* OR self-direct* OR Cash for care OR consumer directed care OR direct payment OR indicative allocation OR individual budget OR individual service fund OR managed account OR managed budget OR notional budget OR personal budget OR personal health budget OR individual?ed fund OR individuali?ed OR personali?ation OR personali?ed care OR person centred OR pooled budget OR recovery budget OR resource allocation system OR self-directed assessment OR self-directed care OR self-directed support OR support plan OR virtual budget OR disability living allowance OR self-determin*))) AND pd(19850101-20161231)** | | | | | | | | | |
| Database | CINAHL (12,903) – title, abstract, keyword | | | | | | | | | |
| Syntax | ( (AB ((MH "Attitude to Disability") OR (MH "Neurobehavioral Manifestations+") OR (MH "Behavioral and Mental Disorders+") OR (MH "Behavior and Behavior Mechanisms+") OR (MH "Disability Evaluation") OR "disabilities" OR (MH "Employee, Disabled+") OR (MH "Community Mental Health Nursing") OR "mental" OR (MH "Mental Health") OR (MH "Health Services for Persons with Disabilities") OR (MH "Mental Health Services+") OR ("Dementia+"))) AND (TX ("personal budget" OR (MH "Health Services Purchasing+") OR (MH "Financial Management+") OR (MH "Financial Support+") OR (MH "Resource Allocation+"))) AND (TI (person* OR individ* OR fund* OR financ* OR cash OR pay* OR self-direct*)) OR (AB(“Cash for care" OR "consumer directed care" OR "direct payment" OR "indicative allocation" OR "individual budget" OR "individual service fund" OR "managed account" OR "managed budget" OR "notional budget" OR "personal budget" OR "personal health budget" OR personalisation OR "personalised care" OR personalization OR "person centred" OR "pooled budget" OR "recovery budget" OR "resource allocation system" OR "self-directed assessment" OR "self-directed care" OR "self-directed support" OR "support plan" OR "virtual budget" OR “disability living allowance” OR "Broker*" **OR** "**self-determin***")) ) OR ( (TI ((MH "Attitude to Disability") OR (MH "Neurobehavioral Manifestations+") OR (MH "Behavioral and Mental Disorders+") OR (MH "Behavior and Behavior Mechanisms+") OR (MH "Disability Evaluation") OR "disabilities" OR (MH "Employee, Disabled+") OR (MH "Community Mental Health Nursing") OR "mental" OR (MH "Mental Health") OR (MH "Health Services for Persons with Disabilities") OR (MH "Mental Health Services+" OR "Dementia+"))) AND (TX ("personal budget" OR (MH "Health Services Purchasing+") OR (MH "Financial Management+") OR (MH "Financial Support+") OR (MH "Resource Allocation+"))) AND (TI (person* OR individ* OR fund* OR financ* OR cash OR pay* OR self-direct*)) OR (TI(“Cash for care" OR "consumer directed care" OR "direct payment" OR "indicative allocation" OR "individual budget" OR "individual service fund" OR "managed account" OR "managed budget" OR "notional budget" OR "personal budget" OR "personal health budget" OR personalisation OR "personalised care" OR personalization OR "person centred" OR "pooled budget" OR "recovery budget" OR "resource allocation system" OR "self-directed assessment" OR "self-directed care" OR "self-directed support" OR "support plan" OR "virtual budget" OR “disability living allowance” OR "Broker*" **OR** "**self-determin***")) ) | | | | | | | | | |
| Database | EconLit with Full text (2,111) - title, abstract, keyword | | | | | | | | | |
| Syntax | ( (AB ((MH "Attitude to Disability") OR (MH "Neurobehavioral Manifestations+") OR (MH "Behavioral and Mental Disorders+") OR (MH "Behavior and Behavior Mechanisms+") OR (MH "Disability Evaluation") OR "disabilities" OR (MH "Employee, Disabled+") OR (MH "Community Mental Health Nursing") OR "mental" OR (MH "Mental Health") OR (MH "Health Services for Persons with Disabilities") OR (MH "Mental Health Services+") OR ("Dementia+"))) AND (TX ("personal budget" OR (MH "Health Services Purchasing+") OR (MH "Financial Management+") OR (MH "Financial Support+") OR (MH "Resource Allocation+"))) AND (AB (person* OR individ* OR fund* OR financ* OR cash OR pay* OR self-direct*)) OR (AB(“Cash for care" OR "consumer directed care" OR "direct payment" OR "indicative allocation" OR "individual budget" OR "individual service fund" OR "managed account" OR "managed budget" OR "notional budget" OR "personal budget" OR "personal health budget" OR personalisation OR "personalised care" OR personalization OR "person centred" OR "pooled budget" OR "recovery budget" OR "resource allocation system" OR "self-directed assessment" OR "self-directed care" OR "self-directed support" OR "support plan" OR "virtual budget" OR “disability living allowance” OR "Broker*" **OR "self-determin*"))** ) OR ( (TI ((MH "Attitude to Disability") OR (MH "Neurobehavioral Manifestations+") OR (MH "Behavioral and Mental Disorders+") OR (MH "Behavior and Behavior Mechanisms+") OR (MH "Disability Evaluation") OR "disabilities" OR (MH "Employee, Disabled+") OR (MH "Community Mental Health Nursing") OR "mental" OR (MH "Mental Health") OR (MH "Health Services for Persons with Disabilities") OR (MH "Mental Health Services+" OR "Dementia+"))) AND (TX ("personal budget" OR (MH "Health Services Purchasing+") OR (MH "Financial Management+") OR (MH "Financial Support+") OR (MH "Resource Allocation+"))) AND (TI (person* OR individ* OR fund* OR financ* OR cash OR pay* OR self-direct*)) OR (TI(“Cash for care" OR "consumer directed care" OR "direct payment" OR "indicative allocation" OR "individual budget" OR "individual service fund" OR "managed account" OR "managed budget" OR "notional budget" OR "personal budget" OR "personal health budget" OR personalisation OR "personalised care" OR personalization OR "person centred" OR "pooled budget" OR "recovery budget" OR "resource allocation system" OR "self-directed assessment" OR "self-directed care" OR "self-directed support" OR "support plan" OR "virtual budget" OR “disability living allowance” OR "Broker*" **OR "self-determin*"))** ) | | | | | | | | | |
| Search Engine | Google Scholar – 5,960 (of which 432 imported into Endnote) | | | | | | | | | |
| Syntax | disability disabled mental disorder budget fund cash allocation personalized | | | | | | | | | |
| Database | OpenGrey.eu – 412 (of which 6 were imported into Endnote) | | | | | | | | | |
| Syntax | Personal Budget – 0/17  Individualised funding – 0/17  Individualized funding – 0/1  individual budget – 0/74  cash and counselling - 0  “consumer directed care” – 0  "direct-payment" OR “direct payment” 1/6 (2 others not available by link or through google)  "personal health budget" – 0  “Person centred” - 0/71  Broker 0/87  Money AND disability 1/2  Money AND disorder 0/1  Money AND mental 0/9  Money AND dementia 0/2  Cash for care - 4/19  Personalisation AND disability – 0/4  AND disorder – 0/1 AND mental - 0/1 AND dementia – 0/1  personalised care AND disability – 0/2  personalised care AND disorder / mental / dementia – 0/1  person centered – 0/13  "self-directed" AND DDMD – 0/5 | | | | individualized AND disability – 0/2  individualized AND disorder – 0/1  individualized AND mental – 0/2  individualized AND dementia – 0/2  individualised AND disability – 0/5  individualised AND disorder – 0/6  individualised AND mental – 0/4  individualised AND dementia – 0/3  personalised AND disability – 0/4  personalised AND disorder – 0/7  personalised AND mental – 0/8  personalised AND dementia – 0  personalized AND disability – 0  personalized AND disorder – 0/1  personalized AND mental – 0/1  personalized AND dementia – 0/1  budget AND disability – 0  budget AND mental – 0/1  budget AND disorder – 0/1  budget AND dementia – 0  "support plan" - 0/7  Self-determined – 0/22 | | | | | |
| Database | GreyLit – 873 (of which 31 were imported in Endnote) | | | | | | | | | |
| Syntax | Handicap – 0/2  Retard – 0/7  Blind – 0/14  Deaf – 0/3  Impairment – 0/35  Autism – 0/9  Autistic – 0/3  Personalisation – 0 / 6  Personalization AND disability – 4 / 265  "personalised care" – 0 / 1  "personalized care" – 2 / 27  "self-directed" – 0/3  broker – 0/5  Cash for care - 2/16  Pooled budget 0/3  self-directed support – 0  indicative allocation – 0/5  recovery budget – 0/18  disability living allowance – 0/1  virtual budget – 0/1  notional budget – 0  individualized funding AND disability – 1/33  individualized funding AND disorder - 0/8  individualized funding AND mental – 0/8  individualized funding AND dementia – 0  cash for care – 0/16 | | Personal budget AND disability – 1/14  Personal budget AND mental – 0/3  Personal budget AND disorder – 0/1  Personal budget AND dementia – 0/0  individual budget AND disability – 0  individual budget AND mental – 0  individual budget AND disorder – 0  individual budget AND dementia – 0  budget AND disability – 0/39  budget AND mental – 0/20  budget AND disorder – 0/15  budget AND dementia - 0  "cash and counselling" – 4/10  “consumer directed care” – 10/38  "direct-payment" OR “direct payment” – 3/22  "personal health budget" – 0/45  support plan AND disability – 1/31  support plan AND disorder – 0/23  support plan AND mental – 0/23  support plan AND dementia – 0/3  “Self determination” - 0/11  resource allocation system – 0/22  Direct payment 0/22 (many repeated)  individual service fund AND disability - 2/23  individual service fund AND disorder - 0/4  individual service fund AND mental - 0/4  individual service fund AND dementia – 0  managed budget AND disability - 0/5  managed budget AND disorder – 0  managed budget AND mental – 1/6  managed budget AND dementia - 0 | | | | | | | |
| Database | Proquest dissertation and Thesis – 7,975 | | | | | |  | | | |
| Syntax | ab(disabil* OR disabl* OR insan* OR handicap* OR dementia OR mental health OR mental* OR infantil* OR disorder OR autis* OR deaf OR blind) AND ab(budget* OR finance* OR fund* OR resource* OR money OR income OR purchas* OR salary OR capital OR investment OR cash OR profit) AND ab(individ* OR person* OR self-direct* OR self-determin*)  OR  ti(disabil* OR disabl* OR insan* OR handicap* OR dementia OR mental health OR mental* OR infantil* OR disorder OR autis* OR deaf OR blind) AND ti(budget* OR finance* OR fund* OR resource* OR money OR income OR purchas* OR salary OR capital OR investment OR cash OR profit) AND ti(individ* OR person* OR self-direct* OR self-determin*) | | | | | | | | | |
| **Database** | VHL Regional Portal - Latin America database - 549 (of which 1 was imported into Endnote) <http://search.bvsalud.org/portal/>  (Excluded Medline from search results – All other databses searched) | | | | | | | | | |
| **Syntax** | **Individualized funding – 0/4**  **Direct Payment – 0/21**  **Cash for care – 0/6**  **Cash and counseling – 0/0**  **Personal budget – 0/71**  **consumer directed care – 0/3**  **person centred – 0/21**  **money AND disability – 0/5**  **Money AND mental 1/82**  **Money AND dementia 0/6**  **disability AND payment 0/8**  **mental AND payment 0/19**  **dementia AND payment -0/1**  **personalised care – 0/25** | | | **"self-directed" – 0/65**  **broker – 0/13**  **Pooled budget 0/0**  **indicative allocation – 0/0**  **recovery budget – 0/7**  **disability living allowance – 0/0**  **virtual budget – 0/6**  **notional budget – 0/0**  **personalisation – 0/6**  **Self-determined – 0/58**  **Support plan AND disability – 0/6**  **Support plan AND mental – 0/104**  **Support plan AND dementia – 0/12** | | | | | | |
| **Database** | **NORART - (Norwegian and Nordic index to periodical articles)**  **601 (zero imported into Endnote)** | | | | | | | | | |
| **Syntax** | **Disability – 0/126**  **Dementia - 0/51**  **Mental – 0/424** | | | | | | | | | |
| **Database** | **Australian Policy Online – 985 (of which 16 were imported into Endnote)** | | | | | | | | | |
| **Syntax** | **Searched “Individualised funding” – 6/21**  **Direct Payment – 2/125**  **Cash for care – 0/36**  **Cash and counselling – 0/3**  **Personal budget – 1/110**  **consumer directed care – 0/54**  **money AND disability – 0/47**  **Money AND mental 0/33**  **disability AND payment 0/78**  **personalised care – 1/13**  **"self-directed" – 2/32**  **broker – 0/55** | | | | | **Pooled budget 0/11**  **indicative allocation – 0/49**  **recovery budget – 0/20**  **disability living allowance – 3/38**  **virtual budget – 0/20**  **notional budget – 0/16**  **personalisation – 0/60 (Some already captured)**  **Self-determined – 0/73 (some already captured)**  **Support plan – 1/91 (many already captured)** | | | | |
| Search engine | Google – 1,000 (of which 25 were added to Endnote) | | | | | | | | | |
|  | Google will be searched to identify any relevant conference proceedings in addition to relevant NGOs that may have relevant research unpublished elsewhere.  The following terms will be searched:  disability disabled mental disorder budget fund cash allocation personalized individualised  200 results were searched, since the latter 100 did not produce any relevant results. 21 relevant results were added to Endnote.  The following specific searches were then conducted searching the first 100 results for each:  **Direct payment disability mental dementia** – 3 (many information leaflets etc but not research, however publications were checked for many organisations)  **Personal budget disability mental dementia** – 0 (Most already reviewed in previous searches)  **Individualised funding disability mental dementia** – 1 (Most already reviewed in previous searches)  **Cash for care disability mental dementia** – 0 (Results were more about paying carers)  **Cash and counselling disability mental dementia** – 1  **Consumer directed care disability mental dementia** – 2  **Brokerage disability mental dementia** – 0  **Individual service fund disability mental dementia** – 0 | | | | | | | | | |
| **Post screen** | 103 additional titles included in full-text screen | | | | | | | | | |
|  | Forward citation searching (40) and hand-searching based on conference papers and other sources that guided the search (63) | | | | | | | | | |
| **Key Journals** | Seven journals were searched using key terms at the end of the screening process (February 2017) - 259 (of which 2 additional titles were screened) | | | | | | | | | |
| **Syntax** | Cash and counseling - 2/5  Cash for care – 0/5  Personal Budget – 0/36  Individual Budget – 0/22  Indicative allocation – 0/8  Notional budget – 0/8  Pooled budget – 0/8  Recovery budget - 0/6  Virtual budget – 1/2 | Personal Health Budget – 0/16  Direct-Payment OR direct payment – 0/73  Individual service fund – 0/0  micro board OR microboard OR micro-board – 0/10  Disability living allowance – 0/8  Supplemental Security Income – 1/1  individualised fund OR individualized fund - 0/22  consumer-directed care OR consumer directed care – 0/32 | | | | | | | | |
