# Supplementary material for: Individualized funding interventions to improve health and social care outcomes for people with a disability: A mixed‐methods systematic review
Source: Campbell Syst Rev. 2019 Jul 19;15(1-2):e1008. doi: 10.4073/csr.2019.3 (PMC8356501; doi:10.4073/csr.2019.3)
Supplement: Supplementary file 2 — Supporting information [file CL2-15-e1008-s002.docx]

# Appendix 2: Paper outlining results refinement process

**Identifying and tackling challenges in undertaking mixed-methods systematic reviews: an exemplar from the field of disability**

Padraic Fleming, Sinead McGilloway

**Introduction**

There are many well documented challenges in undertaking robust systematic reviews (Francis, Baker, & Soares, 2012; Mahood, Eerd, & Irvin, 2014; Runnels, Tudiver, Doull, & Boscoe, 2014) and not least the fact that researchers strive to answer often narrow questions by locating / identifying studies that use precise tools and rigorous methods to measure effect. This is to be expected and endorsed in terms of improving the quality and robustness of such reviews. However, this approach can often lead to ‘empty reviews’, with relatively few studies that meet the very stringent inclusion/eligibility criteria and/or specific search strategy terms; arguably, these may be considered to be of only questionable utility for service providers and policy makers. Very often, the recommendations from an ‘empty review’, is the call for more, and improved research. Such conclusions have drawn criticism, and lead commentators to question the value of systematic reviews (Lang, Edwards, & Fleiszer, 2007). These kinds of challenges may be exacerbated when conducting reviews that involve the use of mixed methods and which require a deeper understanding, perhaps, of contextual factors. For example, the importance of context was a recurring theme at the recent ‘What Works Global Summit 2016’, held in London last November. In terms of context, for example, the ‘Contextualized Health Research Synthesis Program’ (CHRSP), in Canada, argue that the key question to ask when providing evidence-based support for decision makers, policy makers and clinicians is not ‘What works?’ but ‘What will work here? (WWGS, 2016, p. 47).

The current short article arose from an oral presentation at the ‘What Works’ conference which was very well received (in line with the emerging conference theme) and which, as a result, it was felt should be shared with a larger audience. The principal aim of the ongoing review that provided the context for this presentation and the current paper, is to determine whether or not individualised funding is an effective mechanism for improving the health and social care outcomes of people with a disability. A second aim is to critically appraise and synthesise the qualitative evidence relating to stakeholder perspectives and experiences of individualised funding with a particular focus on the stage of ‘initial implementation’ (Fleming et al., 2016).

The specific objective of this current paper is to suggest and highlight strategies for dealing with challenges associated with: (1) addressing a broad research question; (2) using both quantitative and qualitative methods to measure effect; and (3) the need to highlight the importance of context during the implementation of a complex intervention with internationally diverse terminology and a broad range of outcomes. We outline how we addressed these challenges when undertaking a mixed methods systematic review - in the field of disability - which generated initial search results totalling an unmanageable number of 105,329 potentially relevant references/studies.

**Background**

Individualised funding is an umbrella term that encapsulates a growing range of descriptors for a mechanism of allocating disability-sector state funds directly towards the individual with a disabling impairment or their support network. Designating the funds in such a manner aims to place the individual at the centre of the decision making process in order to provide a self-determined life, whereby making day-to-day decisions about personal, health and social care needs empowers the individual to choose what supports they require, how this support is provided, when and by whom (Carr, 2010; Glasby & Littlechild, 2009; United Nations, 2006). This is a paradigm shift away from the traditional agency-directed, group-based provision of services. It is not new however, with (cumulatively) decades of experience in the US, Canada, the UK, Australia and the Netherlands. The emerging language used to describe this new funding model has varied widely including: ‘Cash and Counseling’ in the US; ‘Self-managed Care’ in Canada; ‘Direct Payments’ in the UK; ‘Self-Directed Funding’ in Australia; and ‘Person-centered Budget’ in the Netherlands, to name but a few (Fleming, 2016). This variation in terminology relates to the broader health and social care systems that are in place. The interventions have evolved in these country-specific contexts in different ways but ultimately all strive for personalised services resulting in improved outcomes while using existing state funds.

International policy has been promoting independent, self-directed supports for people with a disability for several decades. On foot of the first independent living movements in the US and Canada during the late 1960s / early 1970s, the UK reached a critical legislative milestone during the 1990s when the Community Care Act was established, emphasising the importance of people with a disability living in their own homes. This was closely followed by the Direct Payments Act 1996, which saw UK national implementation of Direct Payments commence in 1997. The ‘United Nations Convention on the Right of Persons with Disabilities’ (UNCRPD) subsequently endorsed individualised funding as one way to achieve self-determination (United Nations, 2006), leading to smaller countries, such as Ireland, to adapt international best practice within its national policy (Department of Health, 2012; Inclusion Ireland, 2016).

**Method**

The search strategy for the review was purposely broad, in order to identify all relevant quantitative and qualitative studies. Thus, it focused on: 1) the population of interest, itself expansive, including adults (18 and over) with any form of disability, mental health issue or dementia; and 2) the intervention which endeavoured to capture any financial intervention using state funds. Study design, comparator groups or outcomes of interest were not included at search stage. A wide range of academic databases (including general, psychological, medical, social, economic, business and policy), regional specific databases, sources of grey literature and search engines were employed. . Where thesauri existed, relevant subject terms were exploded in order to capture all narrower terms (e.g. Table 1). Known terms, identified in existing literature were also included in the search strings (Table 2). Further detail of the search strategy is available in the protocol (Fleming et al., 2016).

[INSERT TABLE 1 HERE]

[INSERT TABLE 2 HERE]

**Results**

This broad search strategy resulted in 105,329 references, 90% of which (95,245) were automatically imported into Endnote. The remaining 10% (9,562) were screened at search stage since automatic import into Endnote was not possible or would have required reference import on an individual basis, which was not feasible. The latter approach was only required for sources of grey literature, such as ‘Australia Policy Online’ (a grey literature database), where 985 titles were screened online, after the search was conducted, but only 16 were imported in Endnote (top right quadrant of Figure 1). In total, 522 grey literature references were manually imported into Endnote, giving a total of 95,767 references, which were saved in a ‘master file’. Of these references, 13,493 duplicates were removed from a ‘working file’. Only 9,265 were automatically found by Endnote when matching titles against the standard ‘author, year and title’. This was due to discrepancies in author name or order, in year or slight variations in title. After adjusting the criteria for matching ‘title only’ or ‘author only’, a further 4,228 duplicates were identified. The latter was only reached after manual verification that the titles found were in fact duplicates and not, for example, separate publications from same study. In this way, a total of 13,493 duplicates were successfully identified and removed, leaving a total of 82,274 potentially eligible studies for the next ‘refinement’ stage described below.

**[INSERT FIGURE 1 HERE]**

There were limited resources for conducting the systematic review in terms of budget and manpower (as is often the case). Consequently, practicalities dictated the need for a new systematic approach to further reduce the remaining 82,274 titles before double screening of titles and abstracts could commence. This new approach was a departure from the published protocol (Fleming et al., 2016), but provided a robust, transparent and replicable process. This refinement strategy consisted of the three following steps:

1. Text mining

This involved scanning the titles, as displayed in Endnote, to identify irrelevant terms such as ‘embryo, stem cell or biobank’. Using Endnote’s search function, these terms were used to locate titles which included these irrelevant terms. Results were manually scanned to see if there were in fact potentially relevant studies included. If not, all search results were copied to a new Endnote file (named ‘irrelevant titles’) and removed from the ‘working file’. This process was repeated across several hundred terms. All terms identified in the titles and variations or related terms were recorded including the number of titles removed based on each set of terms (Appendix 1).

1. Failsafe check

In order to check if any potentially relevant titles had inadvertently been removed, the ‘irrelevant titles’ endnote file was used to conduct a failsafe check. This involved searching the titles and abstracts for any of the ‘known terms’ (Table 1). This amounted to 149 titles being returned to the working file. In addition, as new terms emerged during the consequent steps in the review, the ‘irrelevant titles’ file was searched for these new terms. These terms can be seen in Table 2, although no further titles were located based on these searches. After steps one and two had been completed, almost two-thirds of the titles (64%, 52,770) had been successfully removed, taking approximately one month’s work (March 2016).

1. Manual title screen

A manual title screen was then carried out in line with our protocol. This involved reading each title to identify clearly irrelevant studies. Despite the previous two steps in the refinement process, many irrelevant studies remained. For example, there were many studies discussing the stock market and in particular brokers within the stock market. However, brokers and brokerage is a commonly used term within the individualised funding literature and therefore any such terms could not be automatically removed. Any ambiguous titles were not removed, since there were no grounds for doing so. In total, a further 22,346 titles, or 27% of the total titles were removed. Once again, step two was repeated as a failsafe check. Figure two below demonstrates the percentage of titles removed per database. Unsurprisingly ‘Business Source Complete’ and ‘EconLit’ saw almost all titles removed (99.2% and 97.6% respectively). Applied Social Science Indexes and Abstracts (ASSIA), on the other hand, had the least removed, although 49.7% were still deemed irrelevant. Step three took another month to complete (April 2016).

**[Insert Figure 2 here]**

Following the search refinement process, the double screening of titles and abstracts commenced with a much more manageable 7,158 references (Figure 1), or 8.7% of the original number. This process took approximately five months with one full time screener and two part-time (second) screeners (May – September 2016). A total of 6,934 titles were removed during this process with an overall inter-rater reliability score of 0.6, which reflects ‘good agreement’ (Higgins & Green, 2011). The inter-rater reliability moved from ‘fair agreement’ (0.4 – 0.59) in earlier batches of reviewed references to an ‘excellent agreement’ score of 0.8 on batches of references reviewed towards the end of the process. This was due to the complexity and variation within the interventions in question, and the need for ongoing clarification, discussion and refinement of the screening process. During the title/abstract screening process, 134 articles, 19 books and several conference proceedings were identified, which did not meet the eligibility criteria, but which may have contained additional relevant references. These documents were used to conduct ‘forward citation chasing’, during the hand-searching process, which led to almost 3,700 titles scanned and an additional 104 references added to the full-text eligibility screen. These additional texts were generally grey literature consisting of organisational and governmental reports which would not be contained within the academic databases searched and often did not contain keywords that would have made them clearly identifiable in the grey literature search. The final number of titles included in the full text review was 328.

**Discussion and conclusion**

There is much work being undertaken to enhance aspects of the systematic review process, with such papers and guidance featured in the Campbell Collaboration Methods Group, for example. However, while innovative methods continue to emerge, researchers are hesitant to negate the proven and often extensive methods undertaken in traditional reviews. Indeed for the current review, one of the two (anonymous) information retrieval specialists who reviewed the protocol, recommended the inclusion of additional databases such as Business Source Complete and EconLit. However, as shown earlier, these databases were totally unsuitable for this particular review. In fact, no references retrieved from these two databases were included in the 286 articles selected for full text eligibility screen. This is useful information because it strongly suggests that these databases should be excluded when updating the review, thereby immediately reducing the number of titles by 3,886 (4%). While this process can only be conducted post hoc, the above example strengthens the argument to present summary tables based on the relevance of databases searched. While only possible post-hoc, presenting statistics on the appropriateness of databases utilised strengthens the argument made by Alison Bethel, data retrieval specialist from University of Exeter Medical School, to generate and report a summary table for systematic review searches. Such summaries show: 1) the databases from which the included references are drawn 2) the databases from which unique references versus duplicates were identified; and 3) those databases which were searched and which contained a relevant reference that was not detected in the search strategy adopted (Bethel, 2016). Furthermore presenting such summary information provides useful information for future search strategy development and filter design by highlighting the unsuitability of some databases in certain subject areas.

The search refinement strategy presented in this paper offers a systematic, robust, transparent, cost and time efficient method of reducing a large number of search results to a more manageable number. As this review demonstrates, it is sometimes necessary to have a very broad and inclusive search strategy but this can lead to inordinately large and cumbersome files when using the traditional, reliable and validated method of systematic review searching. Indeed there are other methods of searching which may also offer a useful alternative, such as the ‘Pearl Harvesting Search Strategy’ (Keenan, Connolly, & Stevenson, 2016). Similar to ‘cluster searching’, this method is particularly useful for reviews of complex interventions that rely on an understanding of context (Booth et al., 2013). Whilst these kinds of search strategies may be more time-efficient, they require a finite list of terms and in the case of our review, this was not deemed feasible in view of the still evolving terminology around individualised funding initiatives within the disability sector.

Further research is recommended to test the sensitivity and specificity of alternative search and/or refinement methods when compared with those that are currently used in practice. The final review report is due to be published in the Campbell Library in 2018.

Bethel, A. (2016). *Search summary tables*. Paper presented at the What Works Global Summit 2016.

Booth, A., Harris, J., Croot, E., Springett, J., Campbell, F., & Wilkins, E. (2013). Towards a methodology for cluster searching to provide conceptual and contextual "richness" for systematic reviews of complex interventions: Case study (CLUSTER). *BMC Medical Research Methodology, 13*(1), 118-118. doi:10.1186/1471-2288-13-118

Carr, S. (2010). *Personalisation: a rough guide (revised edition)*. London: Social Care Institute for Excellence.

Department of Health. (2012). *Value for Money and Policy Review of Disability Services in Ireland*. Dublin, Ireland: Department of Health Retrieved from <http://health.gov.ie/blog/publications/value-for-money-and-policy-review-of-disability-services-in-ireland/>.

Fleming, P. (2016). *How personal budgets are working in Ireland: Evaluating the implementation of four individualised funding initiatives for people with a disability in Ireland*. In G. Trust (Series Ed.), [www.genio.ie](http://www.genio.ie), (pp. 24). Retrieved from [www.genio.ie/personal-budgets](http://www.genio.ie/personal-budgets)

Fleming, P., Furlong, M., McGilloway, S., Keogh, F., Hernon, M., & Stainton, T. (2016). *Protocol: Personal Budgeting Interventions to Improve Health and Social Care Outcomes for People with a Disability: A Systematic Review*. In The Campbell Library, (pp. 73). Retrieved from <http://campbellcollaboration.org/lib/project/350/>

Francis, D., Baker, P., & Soares, J. (2012). Methodological challenges in conducting a systematic review of community wide interventions to increase physical activity. *Journal of Science and Medicine in Sport, 15*, S214. doi:10.1016/j.jsams.2012.11.521

Glasby, J., & Littlechild, R. (2009). *Direct payments and personal budgets: putting personalisation into practice* (2nd Edition ed.). Bristol, UK: Policy Press.

Higgins, J. P., & Green, S. (2011). Cochrane Handbook for Systematic Reviews of Interventions. Version 5.1.0. Retrieved from <http://handbook.cochrane.org/>

Inclusion Ireland. (2016). Inclusion Ireland and Down Syndrome Ireland host successful Personal Budgets seminar. Retrieved from <http://www.inclusionireland.ie/content/news-items/1504/inclusion-ireland-and-down-syndrome-ireland-host-successful-personal-budgets>

Keenan, C., Connolly, P., & Stevenson, C. (2016). Protocol: Universal Preschool- and School-based Education Programmes for Reducing Ethnic Prejudice and Promoting Respect for Diversity among Children Aged 3-11: A Systematic Review and Meta-Analysis. Retrieved from The Campbell Library website: <http://www.campbellcollaboration.org/library/universal-preschool-and-school-based-education-programmes-for-reducing-ethnic-prejudice-and-promoting-respect-for-diversity-among-children-aged-3-11-a-systematic-review-and-meta-analysis.html>

Lang, A., Edwards, N., & Fleiszer, A. (2007). Empty systematic reviews: hidden perils and lessons learned. *Journal of Clinical Epidemiology, 60*(6), 595-597. doi:10.1016/j.jclinepi.2007.01.005

Mahood, Q., Eerd, D. V., & Irvin, E. (2014). Searching for grey literature for systematic reviews: challenges and benefits. *Research Synthesis Methods, 5*(3), 221-234. doi:10.1002/jrsm.1106

Runnels, V., Tudiver, S., Doull, M., & Boscoe, M. (2014). The challenges of including sex/gender analysis in systematic reviews: a qualitative survey. *Systematic reviews, 3*(1), 33-33. doi:10.1186/2046-4053-3-33

United Nations. (2006). Convention on the Rights of Persons with Disabilities. Retrieved from <http://www.un.org/disabilities/convention/conventionfull.shtml>

WWGS. (2016, 26 - 28 September). *Putting evidence to  work for better policy and practice.* Paper presented at the What Works Global Summit 2016, London.


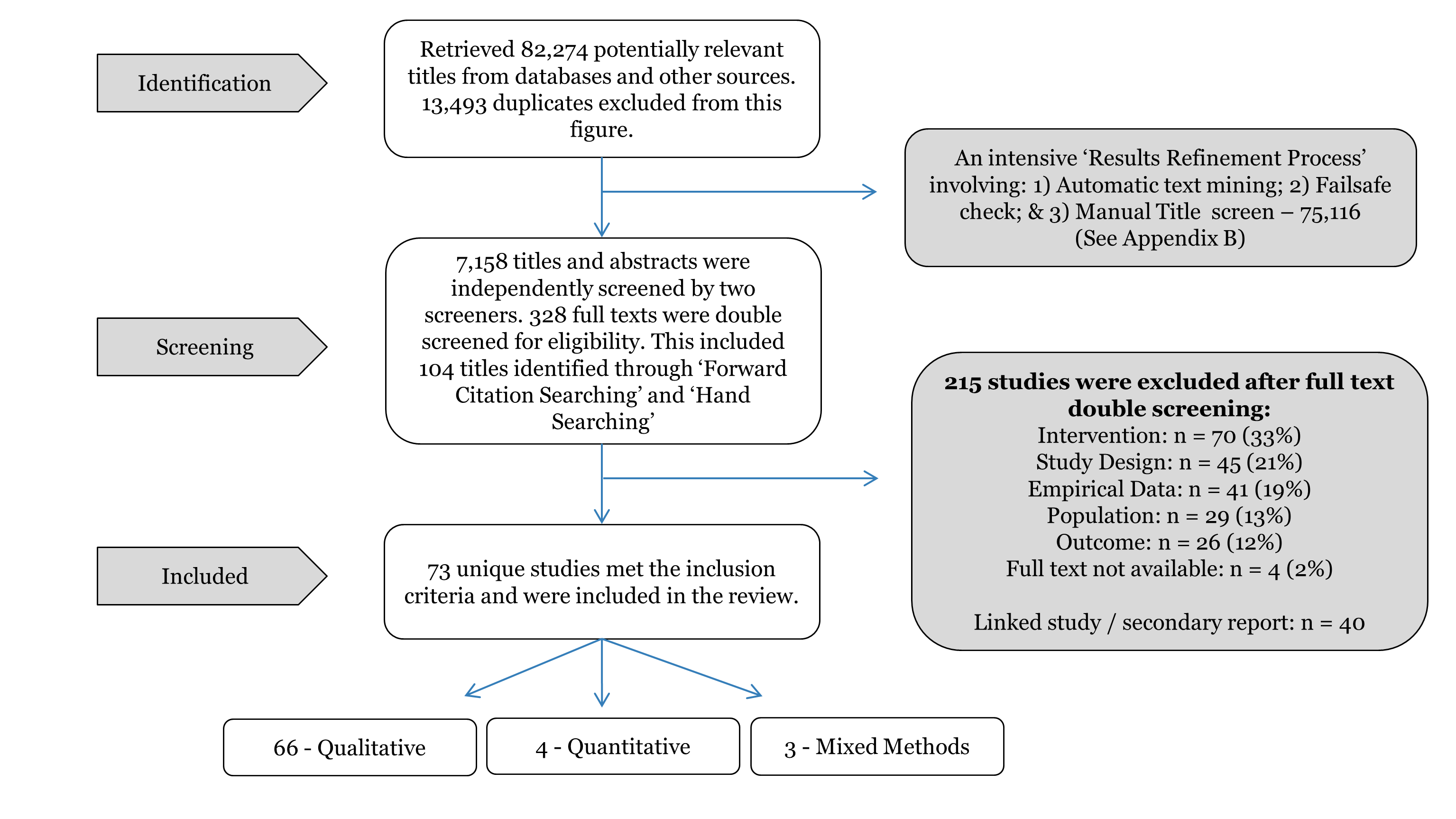


**Figure 1 – Search results refinement process**

**Figure 2 – Percentage of titles removed by type of database**

**during step three of the refinement process**

**Appendix 1**

| Term used for text minding | Number removed |
| --- | --- |
| aboriginal | 93 |
| abortion | 44 |
| acupuncture | 18 |
| addict OR nicotine OR substance | 258 |
| alcohol | 573 |
| algorith OR computat OR simulat OR artificial OR whipash OR in vitro OR in vivo | 163 |
| ambul OR skeleton OR pulmonary OR post-trauma | 172 |
| amphetamine OR methy OR immun OR circulat OR electro OR menstrual | 373 |
| anatonmy OR surgical OR surgeon OR scopic OR analgesia OR rectal OR plasty | 174 |
| animal OR cattle OR laborato OR hemo OR radiatio OR receptor OR inhibitor | 238 |
| antiretroviral | 41 |
| arabic OR middle east OR islam OR muslim OR arab- OR syria | 147 |
| armed conflict | 16 |
| army OR marine | 34 |
| arthritis | 358 |
| artist OR music OR poet OR dance OR television OR cinema OR cyber OR movie | 262 |
| aslyum OR phobic OR phobia | 61 |
| back pain OR yoga | 195 |
| banking | 66 |
| behavior OR behaviour | 968 |
| bereaved OR bereavement | 50 |
| biolog OR antibio OR bioeth OR biomeas OR biomec OR biomed | 127 |
| biomarker | 51 |
| birth OR newborn OR premature OR infan OR perinatal OR neonate NOT country of birth | 178 |
| blood OR ulcer OR vein OR phenyl OR morphol OR transfus OR serum OR neuron OR protein | 168 |
| body mass index OR BMI | 40 |
| border OR healer OR sacred OR god | 73 |
| bowel | 67 |
| brain | 382 |
| breast OR headache OR migraine OR alternative medicine OR complementary medicine OR trimester OR blood pressure | 224 |
| burden | 250 |
| bureaucrat | 33 |
| Cancer | 1114 |
| capital market OR foreign exchange OR real estate OR mortgage | 243 |
| carcinoma | 26 |
| care pathway OR drama OR geriatri | 125 |
| catholic OR priest | 44 |
| childhood OR ADHD OR attention deficit OR attention-deficit | 83 |
| children OR child OR baby OR infant OR adolescent OR teenager OR youth NOT adult | 4155 |
| commercial OR consumeri OR insured OR business | 254 |
| communist OR russia OR soviet union OR crimea | 212 |
| computer AND technology | 558 |
| congenital | 52 |
| congress OR senate | 71 |
| contracepti OR condom | 47 |
| COPD OR pulmonary OR steroid OR T cell OR rheuma OR nervous system OR asthma OR pediatric | 196 |
| corporate NOT incorporate | 182 |
| credit card OR visa OR venture capital OR compulsive buy | 84 |
| credit OR trade-off OR IPO OR coalition | 170 |
| crisis | 162 |
| cultural OR culture | 709 |
| currency OR millenium OR globali | 130 |
| CVD OR cardiac OR heart disease OR angina OR coronary | 201 |
| death OR mortal | 189 |
| dental OR dentist OR teeth OR tooth OR dontal OR oral health OR denture | 252 |
| derma OR psor OR burn injury | 38 |
| design OR access OR wheelchair OR day-care OR daycare OR resuscitat | 468 |
| diabetes | 526 |
| diabetic | 64 |
| disaster OR earthquake OR tsunami OR world trade center OR 9/11 OR bomb OR plague | 144 |
| discrimin OR prejudice OR equality OR unequal OR inequity | 288 |
| divorce OR marriage | 168 |
| Domestic Violence | 60 |
| donor OR donation OR marketing OR advert OR charity OR olympic | 199 |
| dose OR prescrib OR dopamine OR malpractice OR health record OR regulation OR casino | 208 |
| drug | 593 |
| eating disorder OR anorexia OR bulimia OR fat OR obese OR obesity OR overweight OR weight loss OR gastro | 714 |
| education | 775 |
| election OR elector OR president OR dictator OR white house OR voting OR vote OR democra OR non-govern | 658 |
| electr OR energy OR conserva OR ecosy | 147 |
| embryo OR stem cell OR biobank | 66 |
| emergency | 156 |
| end of life OR DNR OR hospice OR end-of-life | 171 |
| engineer OR gene therapy | 35 |
| entrepreneur | 152 |
| environmental OR climate change OR climate OR weather OR hurricane | 439 |
| epidemi | 134 |
| ethnicity OR race | 130 |
| facebook or twitter | 18 |
| feminism | 27 |
| feminist OR conformity OR eugenic OR girl OR boy OR pubert | 77 |
| fibromyalgia OR myocardial OR thrombo OR infarction | 130 |
| financial | 1216 |
| financial crisis OR socialist OR nationalist OR nationalism OR socialism OR patriot | 209 |
| financial service OR federal reserve | 41 |
| fluenz OR vaccine OR fetal OR smallpox OR chicken OR pneumo OR smoke OR H1N1 | 149 |
| food OR nutrient OR nutrit OR farm OR agricultur OR ranch | 475 |
| fracture OR neck OR femoral OR osteoporos OR shoulder OR low-back | 100 |
| fraud OR whistle OR insurance claim OR theft OR burglary OR scam | 49 |
| gambl OR monarch OR laser | 97 |
| gay lesbian homosexual LGBT | 91 |
| general anesthes | 3 |
| general practice OR GP | 117 |
| genetic | 341 |
| Genome | 35 |
| global | 271 |
| Global warming | 4 |
| gulf OR iran OR persian OR kurd OR iraq | 130 |
| gynaecolo OR hysterectom OR steril OR health literacy | 45 |
| haemo OR dialysis | 42 |
| heart OR gout OR atrial OR renal | 198 |
| hip fracture | 42 |
| HIV | 1087 |
| homeless | 235 |
| hospital | 834 |
| HPV | 10 |
| hyperten OR metabol OR pedagog OR radio OR DNA OR vascular | 337 |
| illegal OR crime OR criminal OR forensic | 231 |
| implant OR graft OR ankle OR knee OR skin | 105 |
| incontinence OR urine OR urinary | 62 |
| industr | 247 |
| institution | 464 |
| insulin OR pancrea OR cells OR mice OR insect OR sheep OR apes OR monkey OR paediat | 138 |
| intercourse OR sexual function OR sexual dysfunction OR sexual behavior OR sexual behaviour OR sexual politic | 50 |
| international law OR legiti OR convention | 134 |
| internet OR world wide web | 179 |
| investment | 251 |
| in-vitro fertilization OR IVF OR ferility OR infertility OR reproductive | 107 |
| jewish OR palestine | 74 |
| kidney | 59 |
| label OR hyper OR thyroid OR discount OR patholog OR esophag OR generation | 196 |
| law OR litigation | 245 |
| lesion OR glaucoma OR syphilis OR tuberculos OR malaria OR prophylax | 87 |
| leukemia OR lymph | 56 |
| lifestyle OR migration OR migrant OR oversea OR disaspora | 342 |
| linguistic OR language | 238 |
| love OR faith OR affection NOT love | 115 |
| lung disease OR smoking cessation | 88 |
| mammogram OR mammography | 31 |
| married OR marital | 121 |
| maternity OR paternity OR cannabis OR postnatal OR prenatal OR twins | 114 |
| media NOT mediate OR journalis OR digital OF mobile tech OR euthanasia OR information tech OR e-learn | 208 |
| medical | 473 |
| medication OR medicine OR prescription | 578 |
| memory OR stimuli OR cognitive impairment OR synap | 250 |
| menopaus OR hormon OR ovar | 42 |
| military OR veteran | 338 |
| mindful OR curriculum OR teens | 74 |
| MISC: athlete OR retinopath OR partum OR dyslexi OR dietary OR chiropract OR decolon OR evil OR geography | 160 |
| MISC: brain drain OR antibod OR apartheid OR bully OR amputee OR prosthe OR way-find OR tactile OR orthot | 68 |
| MISC: gait OR lottery OR lotto OR torture OR coffee OR chocolate OR freez OR cesarean | 54 |
| MISC: lonli OR loneli OR lonely OR recycl OR wine OR worldview OR backpack OR tourist OR pilgrim OR crowdfund | 71 |
| MISC: pastor OR gospel OR poverish OR manufact OR surviv | 110 |
| MISC: shooting OR ocular OR battered OR tornado OR holocaust OR stockbroke OR discount broke OR statin OR acoustic | 71 |
| MISC: treaty OR charter OR nazi OR copyright OR caff OR parol OR librar OR color OR colour | 66 |
| morph OR poly OR fragil | 80 |
| motor OR aircraft OR truck OR automobile | 116 |
| mouse OR genom OR enzyme OR sequencing | 52 |
| multimorb OR multi-morb OR frail OR fitness OR incidence | 163 |
| murder | 6 |
| mutual fund OR investor | 166 |
| native amer OR alcatraz | 30 |
| natural disaster | 11 |
| neuro OR urolog OR oncolog | 427 |
| nuclear OR weapon OR conflict OR buddh | 424 |
| nursing home OR respite OR care home | 394 |
| oil OR natural resource | 77 |
| opioid OR drink driving OR buprenor OR methadone OR crystal OR heroin OR detox OR inject | 119 |
| oxygen OR oxide OR water OR gas OR carbon OR pollut OR tropical OR irrigat OR sanitat | 198 |
| palliative | 164 |
| pathology | 158 |
| patient- OR inpatient OR in-patient OR outpatient OR out-patient | 299 |
| payoff OR widow OR shareholder OR tribal OR colonial | 152 |
| peace OR cambodia OR empire OR war- OR sudan OR diplomac OR diplomatic | 181 |
| personality | 685 |
| pharma | 343 |
| philanthrop | 34 |
| phone | 78 |
| physician | 283 |
| police OR policing OR jail OR court | 243 |
| politician | 14 |
| poor OR diet OR poverty | 234 |
| porn | 4 |
| portfolio OR risk aversion | 40 |
| pregnancy OR pregnant | 155 |
| prevalence | 203 |
| prison OR inmate OR incarner OR offender OR juvenile | 248 |
| property OR price | 180 |
| prostitution | 7 |
| protestant OR ortodox OR clergy OR christ OR hindi | 68 |
| psoriasis OR seasonal affective disorder | 23 |
| psychotic OR schizoph | 448 |
| psychotropic OR polypharmacy | 48 |
| PTSD OR posttrauma OR Post trauma OR veteran | 95 |
| radiology OR radiograph OR MRI OR diagnost OR bichemic OR lumbar OR magnetic | 221 |
| recession OR downturn OR austerity | 64 |
| refugee | 102 |
| religion OR religious OR spiritual OR church | 440 |
| residential OR institution OR hospital | 170 |
| retina OR retino OR opthal OR tomography OR pigment OR lipo OR glucose OR macular | 43 |
| risk factor OR depression OR antidepress OR secret OR photo OR hardship | 479 |
| romant OR romanc | 25 |
| same-sex | 20 |
| scholar OR soviet OR hitler OR stalin OR saddam | 67 |
| school OR kinder OR montessori OR arithmetic OR elementary | 1247 |
| screening | 125 |
| sedentary OR respirator OR physical activ | 172 |
| self-harm OR self harm OR suicid OR self-injur | 105 |
| sex offender | 13 |
| sex OR obama' OR prostit NOT essex NOT sussex | 111 |
| sex work | 19 |
| sexual abuse | 52 |
| sexual assualt | 18 |
| sexual OR arousal | 159 |
| sexual violence | 6 |
| sexually | 41 |
| sick | 326 |
| sleep OR wake OR expatriate OR insomn | 131 |
| sodium OR salt OR narcotic OR overdose | 231 |
| soft dollar | 14 |
| soldier | 22 |
| sovereign | 72 |
| sport OR football OR tennis OR golf OR swim OR athletic OR paralympic | 303 |
| stigma OR privilege OR leprosy | 135 |
| stock exchange OR lehman brother OR bank OR liquidity OR hedge fund OR schwab | 341 |
| stock market | 73 |
| stock OR CEO OR debt OR repayment OR forecast OR nasdaq OR NYSE OR S&P | 277 |
| stroke | 499 |
| student OR pupil OR classroom | 1129 |
| substance abuse | 255 |
| suicide | 254 |
| supported employment | 127 |
| surgery OR cervical OR robot OR plastic OR neonatal OR stoma | 168 |
| survivor OR intensive care | 94 |
| teach OR absentee OR lecture | 511 |
| telehealth OR ehealth OR e-health OR wearable OR web | 171 |
| temper | 46 |
| tobacco OR cigarette OR smoking OR cigar | 272 |
| toddler OR stranger OR parenting OR mother OR husband OR wife OR wives | 293 |
| tourism | 54 |
| toxic OR chemical OR placebo OR polar | 283 |
| trade OR stock OR trading | 192 |
| traffick OR cocaine OR heroine | 48 |
| transgender | 33 |
| tumor OR tumour OR antigen OR mice OR t-cell | 80 |
| united nation OR east timor OR kosovo OR indonesia OR regime OR coloniz OR north korea | 135 |
| university OR college OR math OR library | 334 |
| urban slum | 6 |
| vagin OR infect OR chlamydia OR hepatit | 97 |
| verbal OR blog OR social media OR online OR game | 213 |
| victim OR domestic OR probat | 162 |
| violen OR terror OR volatil OR 911 OR maltreat | 359 |
| wall street | 36 |
| weight OR kilo | 82 |
| welfare | 190 |
| womb OR uterus OR uterine OR pelvic OR transplant OR anesthesi OR amnesia OR insomnia | 137 |
| world health organization OR ICD OR IMF | 38 |
| world war OR genoc OR starv | 26 |
