# Supplementary material for: Individualized funding interventions to improve health and social care outcomes for people with a disability: A mixed‐methods systematic review
Source: Campbell Syst Rev. 2019 Jul 19;15(1-2):e1008. doi: 10.4073/csr.2019.3 (PMC8356501; doi:10.4073/csr.2019.3)
Supplement: Supplementary file 3 — Supporting information [file CL2-15-e1008-s003.docx]

## Appendix 3: Qualitative Study Characteristics

| **First Author (year)**  **(linked)** | **Pub Status**  **(linked)** | **Program Name** | **Intervention Description** | **Country**  **(Language)** | **Design** | **N** | **Type of disability** | **Sample Characteristics** | **Funding Source** | **Type of data presented** |
| --- | --- | --- | --- | --- | --- | --- | --- | --- | --- | --- |
| Oliver (1992) | NP  Not Linked | Personal Assistance Scheme with Personal Assistance Advisor | PwD employed and managed PAs directly. Advisors provide advocacy work, advice and information about PA schemes and about managing workers and/or employment law. Nearly a quarter required some kind of practical assistance (e.g. help with applying to Independent Living Fund). | England  English | Mixed methods including a qualitative study and uncontrolled cross-sectional survey.  Face-t0-face interviews were conducted | 48  RR: 33% (16)  PwD only | Not specified | Age:  Range: 32-70  Mean: 45  Female:  n = 9 (56%)  Ethnic/racial minority:  Unknown | Independent Living Fund and the Local Authority. | The majority of data reported was raw data using direct quotes.  RWC: 1,865 |
| Zarb (1994) | NP  Not Linked | Direct or indirect cash payment | Individual workers employed and managed directly by PwD using a direct payment (i.e. paid directly to an individual by either a local authority or the Independent Living Fund) or indirect payments (i.e. payments administered by a third party such as a local disability organisation) | England  English | Mixed methods including qualitative in-depth interviews and an uncontrolled cross-sectional survey (included open-ended questions). | 131 (Qual)  RR: 63%  (83)  PwD only | Not specified | Age:  Range: 16-64  Mean: 45  Female:  78% (of overall sample, n =65)  Ethnic/racial minority:  4% (of overall sample, n = 3) | Independent Living Fund and the Local Authority | A matrix summary of open-ended responses was reported.  The majority of in-depth data reported was raw data using direct quotes  RWC: 10,925 |
| Malette (1996) | NP  Not Linked | Microboard | A micro board is a small non profit society comprised of family and friends who assist PwD to develop individualized housing and support options. This includes direct funding to PwD to secure housing and support options, freedom to choose where and with whom to live, and autonomy in hiring and defining the nature of personal care and support | Canada  English | Qualitative case studies including: Interviews, participant observation, document analysis | 3 | Physical | Age:  Range: 25-27  Female:  66% (n=2)  Ethnic/racial minority:  Not reported | governmental funding source - Community Services Branch (CSB), Ministry of Health | Findings are presented as detailed case studies with extensive use of direct quotes.    RWC:  c.29,197 |
| Walker (1996) | NP  Not Linked | Self-directed personal service (SDPS) program | After a task-based assessment by Long Term Care staff, PwD hire assistants through the SDPS program. A local agency provides payroll support including workers benefits and compensation. | USA  English | Qualitative  Most interviews conducted face-to-face, but where preferred telephone interviews took place. | 90  24%  (22)  16 PwD & 6 PAs | Not specified | Age not specified although Medicaid criteria is mentioned (not detailed). It would appear participants are adults. | Medicaid | Data was presented in summary format with direct quotes used extensively.  Results word count: 1,848 |
| Holman (1999) | NP  Not Linked | Direct payment (DP)  Brokerage services  User-controlled independent living trust | After assessment by the Local Authority direct payment is made to the PwD to secure the relevant services.  Although rare, some people with higher support needs use a service broker to support the PwD to use direct payments.  An innovative framework (legal structure) enabling PwD with high support needs to receive a DP, with decision-making support from dedicated family/friends. | England  English | Qualitative  Informal and semi-structured. | 10  4 case studies involving:  2 DPs, 1 Brokerage user & 1 Trust  (Family members and care coordinators were also interviewed)  6 local authority representatives were also interviewed. | Physical, intellectual and complex, high-support needs. | Two adults did not specify age. One PwD in 20’s, and one 30 years old.  Female:  50% (n = 2)  Ethnic/racial minority:  Not reported | Local Authority Community Care Services | Data presented in 4 cases studies with direct quotes used extensively.  Local authority implementation feedback summarised.  RWC: c. 15,000 |
| Olmstead (1999) | NP  Not Linked | Self-determination using Individual Budget | Self-determination is about sharing power and control and negotiating relationships among consumers, families, advocates, providers and support coordinators. Individual budgets are provided to PwD and families to enable decision making, choice, control & individualised supports. | USA  English | Uncontrolled cross-sectional survey with open-ended qualitative responses | 21  RR: 52%  (12)  Organisational representatives from 11 States. | Developmental, Intellectual & Mental Health | Not reported | Models varied across states but all were using public funds. | Implementation data was summarised under themes with direct quotes used extensively.  RWC: 4,181 |
| Blumberg (2000) | P  Not Linked | Self-directed services utilising Community Service Brokerage  & Direct employment of Personal Support Agent | The Community Support broker works as an intermediary between the PwD / family and the range of available supports. Other services involve planning and management of supports. Control is placed with the PwD/family and not formal service agencies. | USA  English | Qualitative case study of one individual | 1  Self-selected. | Physical | Age: 31  Male | State / federal support dollars | Data is presented in the form of a case study, detailing the story of one persons experience of Self-directed services and Brokerage Service  RWC: 1,866 |
| Dawson (2000) | NP  Not Linked | Direct Payment (with 3 levels of available support) | Direct payments provided by local authorities to individuals in lieu of community care services with 1) self-management, 2) assisted management (PwD choses agent to assist with managing payment/supports), and 3) assisted by the Independent Living Project. | England  English | Mixed methods including qualitative and uncontrolled cross-sectional survey.  People interviewed throughout pilot (some multiple times). | 48  RR: 48%  (23)  Some PwD had supports present. | Physical | Not reported | Local authority in lieu of community care services. | Data was summarised under key themes with direct quote used extensively.  RWC: 8,465 |
| Glendinning (2000b)  **Linked:**  Glendinning (2000, 2000a) | P  Linked:  2 additional qual. titles – 1 P and 1 NP | Direct Payment | Local authority social services departments provide PwD with cash (direct payments) with which to employ their own personal assistants (PAs). | England  English | Mixed methods:  Mainly qualitative but (1 of 3 schemes) conducted a survey providing open-ended qualitative responses.  Face-to-face interviews with PwD and focus groups with PAs. | 58  98%  (57)  44 PwD  13 PAs | PwD with more complex support needs were invited to participate. | Age Range: 18-65  Details of respondents not reported. | Local authority social services department | Data was summarised with extensive use of direct quotes sourced across several titles.  RWC: 7,067 |
| Leece (2000) | P  Not Linked | Direct Payment | A payment made by a local authority to an individual whom it has assessed as needing community care services. The local authority makes the payment instead of arranging the assessed service. | England  English | Qualitative open-ended user satisfaction postal survey, completed anonymously | 19  RR: 68%  (13 / 11 eligible^Ϫ^)  4 provided detailed qual. feedback to the draft findings but not to the original survey. | Physical and Sensory (1) | Of those who provided details  Age range:  21 – 62  Female: 64% (N = 7)  Ethnic/racial minority:  9% (n = 1) | Local Authority Community Care Services | Data was summarised with extensive use of direct quotes. ^Ϫ^Data from 2 respondents who did not receive DP was excluded.  RWC: 1,583 |
| Pearson (2000) | P  Not Linked | Direct Payment | Local authorities make cash payments to service users with physical and sensory impairments and learning difficulties under the age of 65. The majority of respondents received the payment directly, rather than through an intermediary. | UK:  England and Scotland  English | Qualitative  Face-to-face interviews | 100  RR: 35%  (35) | Physical and Intellectual | Age range:  25-60  Female:  40%  (n = 14)  Ethnic/racial minority:  0% (n = 0) | Local authorities | Data summarised with occasional use of direct quotes.  RWC: 2,245 |
| Witcher (2000) | NP  Not Linked | Direct Payments and Indirect payments (via voluntary organisation) | A direct payment is money paid by the local authority directly to a PwD whom it has assessed as needing community care services. The local authority makes the payment instead of arranging services. In practice, recipients often employ their own Personal Assistants (PAs) but this is not a requirement. | Scotland  English | Qualitative.  Semi-structured interviews. Methods adapted in response to assessed support needs, as required. | 31   - 12 PwD   8 professional  4 local organisation reps.   - Data from 7 PwD excluded as not in receipt of DP | 5 Physical & Sensory  3 Physical  3 Learning  1 physical and learning | All adults  Ethnic/racial minority:  0% (n = 0) | Local Authority | Data was presented in various ways. 1) Summary of findings, 2) ‘Pen Pictures/Vignettes’, 3) Summary with extensive use of direct quotes.  RWC: 14,452 |
| Smith (2001) | NP  Not Linked | Person-centred supports | Person-centred supports whereby a budget is determined based on a needs based resource allocation system. The budget is attached to an individual not an agency. | USA  English | Qualitative  Case study  Face-to-face interviews and focus groups. | One case study involving interviews with dozens of families, self-advocates, direct support workers, providers, administrators, and other dedicated advocates | Developmental | Not specified | Medicaid home and community-based waiver | Summary data presented with very few direct quotes used.  RWC: 7,535 |
| Carmichael (2002)  Linked:  Carmichael (2001)  Evans (2002) | P  Linked to 2 additional titles - NP | Direct Payment | Direct Payments enables PwD who need assistance with a range of daily living activities to buy their own support. PwD can choose and control their own support arrangements. Authorities made contractual agreements with third party organisations who oversaw the money on behalf of PwD | England  English | Mixed methods including - User survey, A survey of social services staff, one-to-one interviews, cost comparison,participant diaries, survey of 5 independent DP support orgs, literature review | Unclear – multiple stakeholders representing work with 71 PwD using DPs | Physical (50), Learning (15), Mental Health (4), Older person (2) | Not reported | Local Authority | Data were summarised with extensive use of direct quotes throughout.  RWC: 2,113 |
| Conroy (2002) | NP  Not Linked | Self-determination pilot projects | Three pilots with varying degrees of intermediary and fiscal coordination. Pilot 1 recruited an in-house coordinator to support the PwD and family in developing a budget and plan. Pilot 2 used existing staff for fiscal support and used service coordinators (sometimes private consultant) for planning. Pilot 3 used 10 independent service brokers to provide a menu of needs-led services. Consultants provided training to PwD and brokers. | USA  English | Mixed methods  Including: face-to-face interviews, focus groups, telephone interviews & before and after controlled study (eligible quant data - see Appendix 4). | 112 (Overall)  82 (I)  30 (C)  RR: 68.8% | Mental Health (including various secondary ‘major’ disabilities) | Average age: 25 (I)  Female (I): 29%, n=24  Ethnic/racial minority (I):  46%, n=38 | State funds were used on a pilot basis (outside the standard federal waiver program – to avoid associated rigidity) | Data was presented in various ways including summaries in free text and table format. ‘Stories’ or vignettes were also presented.  RWC: c.7,607 |
| Eckert (2002)  **Qual:**  Phillips (2002, 2006)  San Antonio (2003, 2005, 2007)  Simon- Rusinowitz (2014)  **Quant:**  Foster (2003)  Dale (2004, 2004a, 2005, 2007)  Lepidus (2005)  Brown (2007)  Carlson (2007)  Shen (2008) | NP  Linked:  15 additional titles including 6 qual. (2 P & 4 NP)  and 9 quant. (3 P & 6 NP) | Cash and Counseling | Provides PwD a monthly allowance to hire workers of their own choosing, and to purchase care-related services and goods (within state guidelines). Designated representatives (family / friends) can help PwD to make care-management decisions. | USA  English | Mixed Methods including: Face-to-face and telephone interviews at 2 time points. (’06) Face-to-face interviews (‘02, ’03, ’05) Telephone interviews (’14)  Randomised controlled before/after study (eligible quant data - see Appendix 4). | 59 (2002) 57 (2003) 58 (2005) 90 (2006) 16 (2014)  Total (280 – 36 (ineligible participants) =244  40 PwD 204 Staff | Physical, sensory, intellectual, develop-mental, dementia | Of 40 eligible PwD:  Age-range:  20-85  Female:  55%  (n =22)  Ethnic/racial minority:  50%  (n = 20) | Medicaid | Data for PwD and carers was presented as individual case studies with extensive use of direct quotes.  (‘02, ’03, ’05)  Summary implementation data presented for the staff interviews with extensive use of quotes in (’14) title.  RWC: 134,260 |
| Young (2003) | P  Not Linked | Self-directed care | After self-identifying as capable to self-direct, PwD undergo an assessment of need by a case manager. Case managers allot a number of monthly hours and also provide information. PwD hire, train and supervise the providers. | USA  English | Mixed methods including qualitative case studies, document reviews & telephone satisfaction survey. | Qual:  30 cases involving 86 in-depth interviews including 28 PwD, 30 individual providers and 24 case managers | Not specified | Age-range:  29-72 (mean 49.3)  Female:  69%  (n = 21)  Ethnic/racial minority:  16%  (n = 5) | Medicaid | Data was presented in various ways including summaries in free text and table format.  Sizeable direct quotes were used occasionally  Results word count: 1,905 |
| Breda (2004) | NP  Not linked | Personal Assistance Budget  (Persoonlijke-Assistentiebudget) | Introduced to enable social integration. After an application and assessment report prepared by multi-disciplinary team, funds are made available to PwD to purchase the desired support in their own home situation. PwD have the ability to recruit care assistants themselves and act as their direct employer. | Belgium  Dutch | Mixed methods including qualitative case studies and an uncontrolled cross sectional survey. | 15 cases involving 60 interviews  (- 6 ineligible participants) 9 cases  Family members, friends, carers, PAs, org. reps. Contributed | Of 9 adult PwD:  4 Physical, 3 Mental Health, 2 Sensory or autism | Of 9 adult PwD:  Age range:  29 – retirement age  (mean = 36)  Female:  44%  (n = 4)  Ethnic/racial minority:  Not reported | Flemish Fund | Individual case studies were presented. Data summaries were also provided under key themes. Both used direct quotes extensively.  RWC:  c. 19,000 |
| Jordan (2004) | NP  Not Linked | Direct Payments | Not outlined | England  English | Cross-sectional survey with open-ended questions. | 150  RR: 50%  (n = 75) | Commissioning organisations focus on people with cerebral palsy but references made to intellectual and mental health also. | Directors of social services in local authorities | Local Authorities | Implementation data was presented in summary format with direct quotes used extensively.  RWC: 1,329 |
| Stainton (2004) | P  Not Linked | Direct Payment | PwD become the employers/contractors, in most cases using their DP to recruit and hire personal assistants (PAs) to provide the required support. As such, they take on all responsibilities related to that role, such as insurance, tax deductions,  etc. Direct payments can be used for all eligible community care services except long term residential care. | Wales  English | Qualitative 10 pilot interviews were conducted to identify themes. Followed by 23 in-depth interviews. | 50 (+10 pilot interviews)  RR: 50%  (n= 25)  (-2 ineligible = 23  (+ 10 pilots = 33) | Majority physical | Age range:  < 65  Female:  Not reported  Ethnic/racial minority:  Not reported | Local Authority | Data was summarised with extensive use of direct quotes  RWC: 4,270 |
| Emslie (2005) | NP  Not linked | Individualised planning and support | The intervention aims to enable PwD to live as citizens in the community and to tailor disability and other supports to their needs.  Funds are allocated to PwD and their families / significant others rather than specific programmes. A standard ‘Regional Equity Formula’ is used to assign an amount. Facilitation varied between dedicated support roles and capacity building as part of usual work. | Australia  English | Qualitative face-to-face interviews with 102 PwD plus 8 written submissions.  Plans were reviewed for 71 PwD.  Communication needs are met on a needs-led basis. | 110 PwD or family  (-23 ineligible = 87  257 organisational / community reps. | Full sample of PwD:  41% intellectual, 21% physical, 15% autism, 13% ABI, 8% Neuro and 1% sensory | Full sample of PwD:  Age range:  3 – 64 (Mean 28.8)  Female: 40% (n=44)  Ethnic/racial minority:  0% (n=0) | Department of Human Services | Data was summarised under key themes with extensive use of direct quotes.  Implementation data was summarised using free text and tables.  RWC:  c.27,000 |
| Rosenberg (2005) | NP  Not Linked | Managed care programme with ‘Self-Directed Supports’ option | Self-Directed Supports is a consumer-directed service provision scheme in which control  over specific services like supportive home care, respite care, and transportation rests with the  PwD and their loved ones, to a much greater extent than with conventional agency-directed  services. | USA  English | Qualitative focus groups with PwD, managers, Inter-disciplinary Teams, and org. staff. | 14 focus groups with approx. 85 in total. | Physical , intellectual and developmental | Not reported | Medicaid | Focus group data was summarised under key themes with extensive use of direct quotes.  RWC:  12, 582 |
| Butler (2006) | NP  Not Linked | Private Hire | “Private hire” describes an arrangement whereby families of PwD choose to become employers and hire staff to support their adult sons or daughter. Families receive funding from Government to directly employ at least some of their own supports as opposed to using agencies to do this for them. | Canada  English | Mixed methods- Uncontrolled cross-sectional survey (including open-ended responses) and qualitative study (discussion groups and focus groups) | Survey – 177  RR: 42% (n=75)  Discussion group / Focus groups (n=40+)  Families and support network  c.115 | Developmental | Of survey respondents:  Age range:  18 – 65+ PwD 85% <=34  Female: 44% (n=33)  Ethnic/racial minority:  Not reported | Government of  Alberta’s Persons with Developmental Disabilities (PDD) program | Focus groups / discussion group findings were summarised without use of direct quotes. Open-ended responses (50% of data) were summarised with extensive use of direct quotes.  RWC:  6,048 |
| Sanderson (2006) | P  Not Linked | Self-Directed Support using an Individual Budget | After a self-assessment to determine likely amount of funds and the development of a support plan, a PwD has a number of options to manage the budget. PwD can have their money as a direct or indirect payment, or their care manager could purchase a service on their behalf, or they could ask a provider to keep their money as an individual service fund. | England  English | Mixed methods including uncontrolled before and after survey and qualitative case studies | 31  RR: Unknown | Not specified | Age range: 18-61 | Local authority | Data were presented in various forms including summaries with extensive use of direct quotes. Three case studies were also presented.  RWC:  c. 2,176 |
| Speed (2006) | P  Not Linked | Direct Payment | The PwD becomes ‘the service purchaser’ which enables them to control the use of their care/support money with full budgetary power. More recently this has been enhanced by the creation of the individualised budgets scheme. | England  English | One qualitative self-selected case study | 1 | Physical | Age: Unknown  Male | Local Authority | Data is presented as a case study, under key themes. It is written in the first person.  RWC: c. 2,600 |
| Alakeson (2007) | NP  Not Linked | Self-Directed Care (SDC) using an individual budget | Each PwD is given control of an individual budget with which to purchase goods and services to meet his or her needs. These supports are of their own choosing rather than ones that are chosen on their behalf, and away from providers with whom they do not want to work. | USA  English | Qualitative case studies involving 3 States.  Structured interviews were conducted with PwD,  Staff and State Officials. | Undisclosed number of PwD.  46 interviews with org. staff, policy-makers, academic experts, consumer advocates and provider reps. | Mental Health | Not reported | Medicaid and State general funding in 1 of 3 States | Data is summarised under key themes with minimal use of direct quotes.  RWC:  1,134 |
| Caldwell (2007)  Linked:  Caldwell (2005) | P  Linked: 1 additional Qual. title - NP | Consumer Directed Supports with indiviualised budget | The State provides an individualised budget to PwD living at home with their families. Service facilitators, employed by the state, assist families with developing service plans and coordinate payments for approved services by the state | USA  English | Mixed methods including survey of 294, where qualitative study participants were recruited. Face-t0-face interviews were conducted | 9 PwD with proxy respondents for all cases. Two PwD participated jointly with proxy | Intellectual and developmental  disabilities | PwD:  Age range:  19-47  Female: Not reported  Ethnic/racial minority:  33% (n=3) | Illinois Home Based Support Services Program | Data was summarised with extensive use of direct quotes throughout.  RWC: 3,377 |
| Dimitriadis (2007) | NP  Not Linked | Direct Payment | After an assessment of need, an agreed amount of funding is transferred monthly to a PwD or support person to enable the person to use the funds to purchase supports in line with an agreed plan. PwD is accountable for the budget, which they self-manage. They also monitor the quality of supports and services. | Australia  English | Mixed methods. Qualitative interviews, Cross-sectional surveys, & Secondary data analysis | 25  RR: 40%  (N = 10)  (-1 ineligible = 9 in total)  4 PwD & 5 proxies | 5 physical, 1 sensory, 4 intellectual, 1 intellectual and psychiatric | Age range:  32 – 61  Female :  33% (n=3)  Ethnic/racial minority:  11% (n =1) | Department of Human Services,  Disability Services | Data was summarised mainly by free text with extensive use of direct quotes. Tables were also used to summarise certain themes.  RWC:  c. 10,500 |
| Adams (2008) | NP  Not Linked | Direct Payment Scheme | Direct Payment recipients are placed in the role of employers allowing PwD to receive funding to make their own arrangements for accessing support, rather than using the services provided by their Local Authority | England  English | Mixed methods  Uncontrolled cross-sectional survey (with open-ended qualitative questions included). Face-to-face interviews conducted | 7539  RR: 7% (n=526) | 242 (46%) physical / long term illness, 11 (2%) sensory, 42 (8%)learning, 9 (2%) mental health, 114 (22%) elderly, 108 (21%) carer in receipt of DP | Age range:  18-85+ (89%)  Female:  58% (n = 305)  Ethnic/racial minority:  12% (n =63) | Local Authority | Data were summarised under key themes with extensive use of direct quotes. (sometimes quantifying responses due to the large numbers)  RWC: c. 3,500 |
| Daly (2008) | P  Not Linked | Individual Budget (IBs) | IBs are an extension of Direct Payments bringing together various existing funding streams (Community care purchasing budgets, Community  equipment budgets, Supporting People funding, Disabled Facilities grants, Independent Living Fund, Access to Work ) in order to permit social care users to construct care packages to suit their needs and provide them with the outcomes that they wanted. | England  English | Qualitative action research. An iterative process whereby researchers interviewed PwD multiple time. Individual and group data collection took place | 44  RR: 68% (n=30) | 22 (73%) Learning disability, 7 (23%)Severe physical disability, 1 (3%) enduring mental health need | Age range:  16-50+  Female:  37% (n = 11)  Ethnic/racial minority:  Not reported | Local Authority (Various existing funding schemes) | Data were summarised under key themes with extensive use of direct quotes.  RWC: 1,945 |
| Dinora (2008) | NP  Not Linked | Consumer-directed (CD) personal assistance services | PwD can direct personal assistance, respite, and companion services. The PwD is the employer of record for their workers; meaning they hire, manage, and fire their own workers (including family members). A fiscal intermediary is responsible for payroll functions. | USA  English | Mixed Methods:  Cross-sectional survey (including open-ended qualitative questions) | 783  RR: 19% (n=145) | Physical, Developmental, Mental Health | Age range:  18-75+  Female:  47% (n = 68)  Ethnic/racial minority:  22% (n = 10) | Medicaid | Data were summarised under commonly occurring themes and then quantified. Direct quotes were used sparingly.  RWC: 556 |
| Glendinning (2008)  Linked:  **Qual:**  Glendinning (2009, 2011)  Rabiee (2009)  **Quant:**  Jones (2012)  Netten (2012) | NP  Linked:  5 additional titles including 3 qual. (2 P & 1 NP and 2 quant. (2 P) | Individual Budgets (IBs) | IBs bring together the resources from a number of different funding streams. PwD should have a greater role for self-assessment; greater opportunities for self-definition of needs and desired outcomes; and increased opportunities for PwD to determine those outcomes are achieved. PwD should know how much money they will receive, how much services cost, and they should be offered support in planning. | England  English | Mixed method involving:  Qualitative in-depth interviews with multiple stakeholders  Randomised controlled trial  (See Appendix 4)  91% completed face-to-face. 9% by telephone. | Qual:  130 (Glen. ‘08)  14 (Rabiee ’09)  Staff interviews included: 13 IB leads, 11 training & dev’t., 4 funding stream leads, 1 Occ. Therapist, 23 managers  Total 144+52 =196 | Of 144 PwD:  Physical / sensory 26% (n=37), Intellectual 29% (n = 42), Mental health 16% (n = 23)  Data from older people (n=42) with no evidence of lifelong disability excluded. | Of 144 PwD:  Average age:  18-84  Female:  Not reported  Ethnic/racial minority:  8% (n=12) | Funding was sourced from various funding streams and this varied between the 13 study sites. Types of funding streams included ‘Access to work’ & ‘Independent Living Fund’. | Data from PwD and implementation data were summarised under key themes with extensive use of direct quotes throughout  RWC: 27,364 |
| Homer (2008) |  | Self-directed Support (SDS) using a Direct Payment and other funding streams | SDS is support that is purchased directly by clients using funds from a variety of public sources, including (direct payment, free personal care, supporting people, health, disabled living allowance, independent living fund, access to work, disabled student allowance), which is sometimes brought together into a single pot. | Scotland  English | Qualitative case studies involving PwD and their informal carers where appropriate. | 24 case studies involving 18 PwD and 12 informal carers (-3 ineligible) = 27 | All 24 cases:  Physical 54% (n=13) Learning 21% (n= 5) Multiple 17% (n=4) Mental health 8% (n=2) Dementia 8% (n = 2) | All 24 cases:  Age range:  8-80  Female:  67% (n=16)  Ethnic/racial minority:  4% (n=1) | Local Authority (Various existing funding schemes) | The data is summarised using direct quotes extensively.  RWC: 15,385 |
| Lord (2008)  Linked:  Lord (2006) | P  Linked to 1 additional title - NP | Individualised funding | A funding allocation to individuals and/ or families by government (in contrast to block funding to agencies) on the basis of the person’s specific disability related needs and support requirements, enabling the person to acquire services and supports of their choice. Funding agreements determined by policy are used and typically include various funding levels as well as funding limits. | Canada  English | Qualitative case study design including document analysis (support plan, budget etc.), and face-to-face interviews with families. | 4 case studies involving 130 files & 18 families (-1 ineligible) = 17. | Of all 18 families:  Developmental 98%, Psychiatric/  mental health 40%, Physical disability 38%, and Autism 19% | Of all 18 families:  Age range: 9 -82 (mean =36)  Female: 50% (n = 9)  18 countries represented (English as primary language 93%) | Multiple government sources | Data were summarised under key themes with occasional use of direct quotes.  RWC: 2,856 |
| Shaw (2008) | NP  Not Linked | Direct Payments | Direct payments are money given to an individual, by social services, to buy the support that they have been assessed as needing. An individual can take this payment instead of using a service provided by social services. Direct payments can be used to pay for a range of assessed needs. | England  English | Mixed methods: using a series of cross-sectional surveys including qualitative open-ended questions and space for comments | 28 (Respondents of scoping survey)  RR: 71%  (n = 20 – using a DP) | Deafblind | Of 20 DP users:  Age range:  Not reported  Female:  Not reported  Ethnic/racial minority:  5% (n=1) | Local authority | Qualitative data was summarised using tables and free text with extensive use of direct quotes.  RWC: 3,457 |
| Coyle (2009)  Linked:  Coyle (2011) | P  Linked to 1 additional title - P | Individual Recovery Budget | A recovery budget for this project meant a payment that can be used by the service user to acquire or purchase an item/service/access that would enhance their life, meet agreed goals and contribute to their place within their community. | England  English | Qualitative narrative case studies involving face-to-face individual interviews (twice) and focus groups (twice). | 13  7 PwD, 1 broker & 2 focus groups with staff members  (5 people in total) | Mental Health | Age range: Not reported  Female :  15%  (n = 2)  Ethnic/racial minority:  Not reported | Local Authority | Data are summarised under key themes with extensive use of direct quotes.  RWC: 12,855 |
| Rogers (2009) | NP  Not Linked | Self-Directed Support | Self-directed support aims to make PwD more socially included by allocating money to enable active participation as citizens and partners in the process of designing support plans. Allocation is based on an assessment of that focuses on support recovery outcomes such as managing safety, activities of daily living and accessing the community. | England  English | Case studies using mixed methods including qualitative interviews, secondary data analysis and a survey. | 18  RR: 94%  (n=17)  4 PwD, 2 carers and 11 staff | Mental health (n =6)  Physical (n=2) | Of 6 cases:  Age range:  Not reported  Female :  67% (n=4)  Ethnic/racial minority:  Not reported | Local Authority using a pooled  budget for social care within mental health. | Data are summarised under key themes with extensive use of direct quotes.  RWC: 2,896 |
| Sayles Wallace (2009) | NP  Not Linked | Participant direction | Participant direction allowed recipients increased decision-making authority over Waiver services. Where PwD were not ready and able to take additional responsibility for  management of those services dedicated representatives (when chosen by the individual with a disability) were able to exercise decision making authority on the individual’s behalf. | USA | Mixed methods involving a scoping survey and pilot interviews &  Qualitative case studies using telephone interviews, with opportunity to preview questions | 89 families surveyed  Current study: Subset of 8%  n=7 PwD (from 6 families) | Intellectual/cognitive; autism; sensory; physical disability; mental health | Age range: 25-45  Female :  29%  (n = 2)  Ethnic/racial minority:  14% (n=1) | Medicaid Home and Community Based Waiver | Data were summaries under key themes with extensive use of direct quotes and real life examples.  RWC: 13,333 |
| Eost- Telling (2010) | NP  Not linked | Personal Budget | Following a self-assessment questionnaire and use of a resource allocation system, an allocation is made based in need. Personal budgets can be delivered and managed in a number of flexible ways to best suit the PwD, including direct payment, payment managed by a dedicated representative, payment to a trust acting on behalf of PwD, use of intermediary service to manage payment, managed by service provider as an individual service fund, or managed by the council on behalf of the PwD. | England  English | Mixed methods including: Qualitative study & uncontrolled cross sectional survey and secondary data analysis | 179  RR: 27% (n = 48)  11 PwD  37 (practitioners and brokers) | Mental Health | Of 179:  Age range:  18-79  Female:  Not reported  Ethnic/racial minority: Not reported | Various: Local Authority, Disabled facilities grant, Supporting people, Access to Work, Independent living fund, Integrated Community equipment services | Data were summarised under key themes but much of the data were sizeable direct quotes  RWC: c. 23,500 |
| Kinnaird (2010) | NP  Not Linked | Personalisation using direct payments | Personalisation - the umbrella term that describes an individual having choice and control over the shape of their support along with a greater emphasis on prevention and early  Intervention. Direct payments (the main approach used) - money in lieu of services organised by a social work department, allowing PwD with an assessed need the opportunity to arrange their own personalised care. | Scotland  English | Mixed methods including Qualitative interviews and a questionnaire for Local Authorities.  Face-to-face (81%) or telephone (18%) semi-structured interviews | 12 case studies involving 12 carers of PwD and 10 social work staff  N = 22 | Dementia | Of 12 cases:  Age range:  60-80+  Female:  67% (n=8)  Ethnic/racial minority: Not reported | Social work department but can also incorporate additional funding sources including health board monies and the Independent Living Fund. | Individual case studies were presented as well as summarised data with extensive use of direct quotes.  RWC: 8,073 |
| Leahy (2010) | NP  Not Linked | Self-managed model | For PwD transitioning to adulthood. The PwD and their family/ advocate can choose their supports and activities including how, when and where these are delivered. Activities are based on an individual plan. A service provider acts as an intermediary to facilitate this process, supporting direct employment of support workers, and providing information, guidance and administration support as required. | Australia  English | Mixed methods including: qualitative interviews & uncontrolled cross-sectional survey | 47 PwD  RR: 57% (N = 27)  + 9 service providers = 36 total | Various | Age range:  Not reported  Female:  Not reported  Ethnic/racial minority: 28% of total population (n = 13) | NSW Department of Disability, Ageing and Home Care (DADHC) Community Participation program | Data was summarised under key themes with extensive use of direct quotes and vignettes.  RWC: 9,477 |
| Priestley (2010)  **Linked:**  Priestley (2004)  Riddell (2006)  Williams (2006) | P  Linked: 3 additional titles. 2 – NP  1 – P (Book chapter) | Direct Payments | Direct payments offer cash payments in lieu of traditional services for those eligible to receive community care. They place the recipient in control of the human and financial resources allocated for support. | UK: England, Scotland, Wales and Northern Ireland | Mixed methods including 8 case studies, document analysis, 32 local authority questionnaires, 21 interviews with policy makers  And activists and 102 telephone interviews with practitioners. | 8 case studies  32 surveys, 21 face-to-face interviews & 102 telephone interviews | Various | Not reported | Local authority | Data were summarised under key themes with extensive use of direct quotes.  RWC: 12,666 |
| Vinton (2010) | P  Not Linked | Consumer and caregiver-directed  pilot program | This was flexible state funded programme that included (1) greater choice over  type and amount of services, (2) control of funds, (3) choice  over hiring providers (including non-Medicaid providers such as family  members), and (4) flexible purchasing guidelines. A small number of PwD were self-reliant. Representatives could provide care themselves but some preferred to directly hire a support coordinator for assistance with case management. | USA  English | Mixed methods including qualitative focus groups and uncontrolled pre-post survey (including open-ended questions) | 44 (+ 3 open-ended survey respondents) = 47  9 PwD, 27 family carers, 2 nonfamily carers, 4 service providers; and 2 support coordinators | Developmental | Of 38 family reps. Providing data:  Female: 76% (n=29)  Ethnic/racial minority: 34% (n=13) | State funds, along with monies from a developmental disabilities  council. | Data were summarised under key themes with extensive use of direct quotes.  Results word count: 1,027 |
| Williams (2010) | P  Not Linked | Self-Directed Support using Individual Budgets | ‘Self-Directed Support’ refers to the new ‘operating system’ for social care, pioneered by In Control. ‘Individual Budgets’ are any form of ‘social support’ whereby the person receiving it knows the level of monetary value and exercises choice about how that sum is used (within limitations of safety, legality and meeting needs). | England  English | Qualitative data based on a one day workshop. | 11 (social workers) | Not specified | Not reported | Local Authority | Data were summarised with a combining occasional and extensive use of direct quotes.  RWC:  1,184 |
| Wilson (2010) | NP  Not Linked | Personal Budget | A personal budget is finance allocated to an individual to be used for their care and to enhance their quality of life following a needs assessment. This personal budget can be held by the individual and paid directly into their bank account (a direct payment) to purchase agreed supports. The personal budget can also be managed by the local authority. | England  English | Mixed methods: Uncontrolled Cross-sectional survey (with open-ended questions) | 735  RR: 28% (n = 203) | Physical, Intellectual, Mental Health | Age range: 18-75+  Female: 69% (n=140)  Ethnic/racial minority: Not reported | Local Authority Social Care Fund | Almost all data was reported as direct quotes under the open-ended question. Summary data with use of quotes also reported.  RWC: 7,939 |
| Campbell (2011) | NP  Not Linked | Support Planning & Brokerage using a Personal Budget (PB) or Direct Payment (DP). | The study examined support planning & brokerage by a user-led org. compared to local authority. PwD availed of a PB - an allocation of money from the local authority, to meet a PwD’s assessed needs for a given period of time. A PB can be taken as a DP or leave the LA with the responsibility for commissioning the relevant services.  With a direct payment an individual elects to receive their personal budget as a cash allocation (into a bank or similar account). They can self-manage the budget or can delegate management to a third party. | England  English | Mixed methods involving qualitative interviews at two time points and secondary data analysis. | 80  RR Time 1: 100%  RR: Time 2: 88% | Physical/sensory, intellectual, mental health, older people | Age range: 18-76+  Female: 56% (n=45)  Ethnic/racial minority: 10% (n=8) | Local Authority | Data was summarised without use of direct quotes.  RWC: 12,393 |
| Hatton (2011) | NP  Not Linked | Personal Budget | Personal budget can be taken by an individual as a direct (cash) payment; as an account held and managed by the council in line with the individual’s wishes; or as an account placed with a third party (provider) and called off by the individual; or as a mixture of these approaches. | England  English | An uncontrolled cross-sectional survey with open space for qual. comments.  Available online, in easy-to-read format and as standard paper survey | 1,114 PwD  950 carers = 2,064  RR: 41%  N=851 (left qual. comments)  417 PwD  434 carers | Aged 16-64: Physical (25%), Mental Health (8%), Learning (17%) | Of PwD:  Age range: 16-65+  Female: 61% (n=c. 254 )  Ethnic/racial minority: 11%  (n= c. 46) | Local Authority | The data were summarised using free text with extensive use of direct quotes. Key themes were also summarised using tables.  RWC:  c. 2,044 |
| Lambert (2011) | NP  Not Linked | Personal Budget (PB) | A PB is money allocated to an individual by their local authority to cover their social care needs; aiming to give more choice and control over how their social care needs are met. Of sample 38% opted for a direct payment, 45% decided to leave it to their council or another 3^rd^ party to commission and arrange services on their behalf (but still retaining choice over how their care needs are met and by whom), while 17% choose a combination of the two | England  English | Qualitative in-depth interviews.  Staff interviews took place first to build implementation context. | 48 PwD/carer  6 staff  = 54 | Physical, Learning and Mental Health, Dementia | Of PwD: Age range: up to 71+  Female: 52% (n=25)  Ethnic/racial minority: Not reported | Local Authority | Data were summarised under key themes with extensive use of direct quotes and case studies throughout  RWC:  12,981 |
| Newbronner (2011) | NP  Not Linked | Personal Budget  (with various models of delivery described) | Depending on the Local Authority, delivery of PBs were described differently including: Self-directed supports (including PB), Direct Payments, Indirect Payment via a representative, and Commissioned services | England  English | Qualitative case studies including: face-to-face interviews, telephone interviews, focus groups, small implementation discussion groups | 13 sites  RR: 38% (n=5 case studies) involving 30 (PwD/carers) + 40 implementation leads = 70  Note: 39 ineligible people excluded | Mental Health and Dementia  (only data where disability specified were extracted) | Of PwD:  Age range: 18 – 65+  Female: 70% (n=21)  Ethnic/racial minority: 23% (n=7) | Local Authority | Data were summarised with the extensive use of ‘personal stories’ and case study ‘positive practice examples’ throughout.  RWC:  8,218 |
| Ridley (2011) | NP  Not Linked | Self-Direct Supports (SDS) | The range of SDS options included both Direct Payments (DPs) and Individual Service Funds (ISFs), as well as individually tailored local authority services. DPs were clearly the most common SDS option across all 3 test sites. These were either managed directly by the PwD or managed by a 3rd party, usually family members. The second most common SDS option was ISFs arranged with external providers. | Scotland  English | Case study approach involving mixed methods including in-depth interviews and secondary data analysis of 132 PwD. | 30 qualitative case studies from 3 sites involving 30 PwD/carer and care manager (c. 90) plus 12 stakeholder interview  = 102 | Learning 33% (n=10), Physical 23% (n=7), Older – over 65 3% (n=1), Dementia 3% (n=1), Multiple disabilities 27% (n=8), Mental health 3% (n=1), Autistic 7% (n=2) | Of 30 PwD: Age range: Unclear  Female: 40% (n=12)  Ethnic/racial minority: 0% (n=0) | Local Authority | Data were summarised using free text with extensive use of direct quotes and extensive use of vignettes throughout.  RWC: 5,373 |
| Secker (2011) | NP  Not Linked | Personal Budget | Definition not included. | England  English | Mixed methods including uncontrolled cross-sectional survey (including space for comments), follow up in-depth interviews (n=2) | 14 | Mental Health | Age range: 20-65+  Female: 64% (n=9)  Ethnic/racial minority: 21% (n=3) | Making Involvement Matter in Essex pilot project | Data were summarised under key themes with extensive use of direct quotes / comments  RWC: 2,022 |
| Rummery (2012) | NP  Not Linked | Self-Directed Support (SDS) | SDS offers a range of options giving service users increased control over more personalised services. SDS aims to *empower people and to put the principles of independent living into practice. It enables individuals to direct the care or support they need to live more independently at home and can be instead of, or in addition to, services that might be arranged by their Local Authority.* | Scotland  English | Mixed methods including uncontrolled cross-sectional survey (including space for comments), and interviews and focus groups | 210  RR: 28% n = 59  plus  Focus groups n = 8 PwD, 2+6  service providers interviews n =2 & groups interviews n = 6  Total = 75 | Physical 69% (n=40), learning 17% (n = 10), age related disability/illness 9% (n=5) and mental health 5% (n= 3) | Of survey respondents:  Age range: 16-65+  Female: 59% (n=35)  Ethnic/racial minority: Not reported | Local Authority | Data were summarised under key themes with extensive use of direct quotes / comments  RWC:  3,097 |
| Sheikh (2012) | NP  Not Linked | Personal Budgets | Not defined | England  English | Longitudinal qualitative study involving interviews with PwD and service providers, followed by focus groups with frontline practitioners. | 55 PwD in total (46 at Time 1, 26 at Time 2 & 20 (+9) at Time 3.  17 service providers  7 practitioners  (- 25 ineligible older people)  = 54 | Of 46 Time 1 PwD:  Older 54% (25), Physical / Sensory 28% (13), Learning 17% (8)  At time 3 66% of respondents had a disability, while only 33% were older people. | Not reported | Local Authority | Data were summarised under key themes with extensive use of direct quotes. It was easy to disaggregate ineligible participant.  RWC:  13, 369 |
| Gross (2013) | P  Not Linked | Participant Direction | A service delivery model in which the consumer of public benefits (in this study, Medicaid Hone and Community Based Supports (HCBS) waiver services) has some level of choice and control (e.g., employer authority, budget authority) over the supports and services received. | USA  English | Qualitative case study involving individual interviews, group interviews and document review.  1 interview conducted by telephone, the remaining were face-to-face | 4 cases studies with 22 respondents. This included 4 PwD and their network of support and case manager. | Intellectual and developmental, Autism, Sensory | Of 4 PwD:  Age range: 20-29  Female: 25% (n=1)  Ethnic/racial minority: 25% (n=1) | Medicaid HCBS Waiver | Data were summarised under key themes with extensive use of direct quotes.  RWC:  c. 3,176 |
| Hatton (2013) | NP  Not Linked | Self-Directed Support using Personal Budget | The intention of self-directed support is that individuals who need support and their families are told how much money their council will make available to them to meet their needs. The person needing support and those closest to them can then determine how best to use these resources and develop a support plan describing how the person’s needs are going to be met. | England  English | An uncontrolled cross-sectional survey with open space for qual. comments.  Available online, in easy-to-read format and as standard paper survey | 2,022 PwD  1,386 carers = 3,408  RR: 29%  N=978 (left qual. comments)  488 PwD  490 carers | Aged 16-64: Physical (53%), Learning (34.7%), long-standing illness or condition (29.7%), Sensory (11.2%), Mental Health (1.7%), | Of total sample pf PwD:  Age range: 16-65+  Female: 54%  1,089  Ethnic/racial minority: 7% (n=145) | Local Authority | The data were summarised using free text with extensive use of direct quotes. Key themes were also summarised using tables.  RWC:  c. 3,891 |
| Rees (2013) | NP  Not Linked | Self-directed or Self-managed | **Self-directed:** Funding is provided to an organization that is accountable to funders. The PwD/family/significant other people direct the required types of services & supports, and report directly to the organisation.  **Self-managed:** The PwD/family/significant other people manage the all aspects of the funding, and provide financial acquittals and reports directly to the funding body / government, or do so in consultation with an agency. | Australia  English | Qualitative study involving face-to-face interviews or telephone interviews. | 48  19 PwD  21 Family members  -10 (6 pwd/4 family) not yet commenced intervention = 38 | 16 Learning, 16 Physical, 12 Acquired disability, 4 Psychiatric, Sensory/Speech | Not Reported | Department of Families, Housing, Community Services and Indigenous Affairs | Data were summarised under key themes with extensive use of direct quotes.  RWC:  c. 11,760 |
| Bola (2014) | NP  Not Linked | Personal Budget (PB) | PBs are based on assessed social care needs, but usually only available to people with substantial or critical needs. A PB should allow the service user the ability to plan and purchase their support with the allocated funds. The budget can be used in a variety of ways to meet the person’s eligible needs for social care. | England  English | Qualitative study involving face-to-face interviews and focus groups with various stakeholders | 49  15 PwD / carer / 28 service providers, 3 commissioners & 3 experts | Mental Health | Not reported | Local Authority | Data were summarised under key themes with extensive use of direct quotes, separated by stakeholder group.  RWC:  11,295 |
| Junne (2014) | P  Not Linked | 3 types of Direct Payment:  1 – direct payment through subsidised workplace2 – Personal Budget (PB) for PA 3 – ‘employment model’ | 1 – budget users worked in a protected and subsidised workplace and since 2011 have been able to receive a direct payment to pay for the subsidised job themselves, with a small portion free to use  2 – PB for assistant services to aid societal integration  3 - employment model’ which enables individuals with a severe handicap to organise their own 24-hour-care | Germany  English | Qualitative study involving 23 face-to-face interviews (some in pairs) and 4 telephone interviews. | 37  (14 budgets users, 11 care assistants, 9 employees of care providers, 3 administrators/LA) | Various: ranging from serious physical to mental health | Not reported | Local Authority (Länder) within the federal state of Germany. | Data were summarised under key themes with extensive use of direct quotes.  RWC:4,625 |
| Buchanan (2014)  **Linked:**  Peterson (2014) | P  Linked to 1 additional title - P | Individualised funds and shared management, person-centred and self-directed (SPS) services. | SPS services provide consumers with access to shared management and person-centred relationships with staff, such as a broker, advisor, guide, mentor, or support worker. Shared management requires both consumers and staff to be responsible for managing individualised funds and recovery resources, and staff to support consumers’ freedom, responsibility, and accountability in progressing their recovery journey | Australia  English | Qualitative document analysis of 473 documents based on 16 PwD’s individualised funding and hours of support. | 16 | Mental Health | Mean Age:  46  Female: 56%  (n = 9)  Ethnic/racial minority: 7% (n=1) | A non-denominational community benefit service  provider of mental health services | Data were summarised under key themes with extensive use of direct quotes. Tables were also used.  RWC:  9,251 |
| Waters (2014) | NP  Not Linked | Self-Directed Support using Personal Budget | The intention of self-directed support is that individuals who need support and their families are told how much money their council will make available to them to meet their needs. The person needing support and those closest to them can then determine how best to use these resources and develop a support plan describing how the person’s needs are going to be met. | England  English | An uncontrolled cross-sectional survey with open space for qual. comments.  Available online, in easy-to-read format and as standard paper survey | 2,679 PwD  1,386 carers = 4,065  RR: 76%  N=3,103 (left qual. comments)  c. 2,036 PwD  c. 1,067 carers | Of total sample: Physical (53%), Learning (34.7%), long-standing illness or condition (29.7%), Sensory (11.2%), Mental Health (1.7%), | Of total sample of PwD:  Age range: 16-65+  Female: 57%  c. 1,532  Ethnic/racial minority: 7% (n=177) | Local Authority | The data were summarised using free text with extensive use of direct quotes. Key themes were also summarised using tables.  RWC:  c. 1,373 |
| Coles (2015) | P  Not Linked | Direct Payment (DP) managed by a third party ‘suitable person’ | The parents in the research had taken on the role of a ‘suitable person’ (‘SP’), which was a term introduced in England and Wales due to the extension of the DP scheme under the Mental Capacity Act. Parents make decisions about the day-to-day management of care, in their role as ‘SP’. However, local authorities effectively maintained the right to counter the PwD’s initial decision to delegate to a suitable person. | England  English | Qualitative study involving interviews, observation, document & correspondence review | 8 PwD represented by 12 parents | Learning disability and complex support needs | Age range: 25-40  Female: 25%  (n =2)  Ethnic/racial minority: Not reported | Local Authority | Data were summarised under key themes with extensive use of direct quotes.  RWC: 2,282 |
| Glendinning (2015) | P  Not Linked | Personal Budget managed by third party | PBs can be allocated as cash direct payments (DPs), held and managed by the PwD or a third party such as a carer or support organisation, or held by the local authority and used to purchase council-commissioned services on the user’s behalf. PB levels are reduced to take account of help given by family carers (so long as they are willing and able to continue providing this). | England  English | Qualitative study using in-depth interviews | 14 dyads | 10 learning disability, 4 older person (2 Stroke, 3 dementia, 1 bi polar) | Age range: 20s-80s  Female: 43% (n = 6)  Ethnic/racial minority: Not reported | Local Authority | Data were summarised under key themes with extensive use of direct quotes.  RWC: 2,785 |
| Hamilton (2015b)  **Linked:**  Qual:  Larsen (2015)  Clewett (2015)  Hamilton (2015)  Hamilton (2015a)  Tew (2015) | P  Linked to 5 additional titles - P | Personal Budget | Personal budgets aim to provide ‘maximum choice, control and power’ over how any funding social care funds allocated to PwD,  through the mechanism of a personal budget.  The funding may be passed over to them or a proxy (as a ‘Direct  Payment’), or they may be invited to influence the spending of a budget  managed on their behalf by a local authority or other organisation. | England  English | Qualitative in-depth interviews. | 53 service users  &  18 family carers  &  28 Mental Health Practitioners) = 99 | Mental Health | Age range: 21-71 (Mean =44)  Female: 60%  (n =32)  Ethnic/racial minority: 13% (n=6) | Local Authority | Data were summarised under key themes with extensive use of direct quotes.  RWC:  13,708 |
| Jepson (2015)  **Linked:**  Laybourne (2014) | P  Linked to one 1 additional title - P | Indirect Payment | Direct payment managed by a third party. If a PwD is considered to lack capacity to consent to make a decision about a DP, a ‘suitable person’ can be appointed to receive and manage the payments on their behalf. This allows the PwD to have a type of relational autonomy, where another person in their life can make the significant decisions about a DP for them | England  English | Qualitative in-depth interviews. | 18 PwD  &  67 social workers  N=85 | Dementia | Not reported | Local Authority | Data were summarised under key themes with extensive use of direct quotes.  RWC:  c. 6,238 |
| Jones (2015) | NP  Not Linked | Individual packages: Supported Living Fund (SLF) and Individual Accommodation Support Packages (IASP) | Both SLF and IASP were recurrently funded, intended to complement informal supports, enabling PwD to create living arrangements to suit their life and preferences, and provided access to paid supports and services. People who used the were not tied to any one service provider or living arrangement, rather the funding was portable throughout NSW, remained with the person and was administered by a service provider of the person’s choice.  IASP was a recurrently funded package | Australia  English | Mixed methods including: Uncontrolled Cross-sectional survey (with open-ended responses) and Qualitative in-depth interviews with PwD, family, managers. Focus groups with support workers | 130  RR: 69%  90 (qual. interviews- 30 with PwD) | Intellectual 76.7%, Other 23.3% including specific learning/Attention Deficit Disorder, Autism, Physical, Acquired brain injury, Neurological, Sensory and speech, Psychiatric | Of 30 PwD:  Age:  80% < 45  Female: 45% (n =14)  Ethnic/racial minority: 3% (n=1) | Department of Family and Community Services, Ageing, Disability and Home Care (ADHC) | Data from all sources were summarised with use of direct quotes throughout.  RWC:  21,815 |
| Laragy (2015) | P  Not Linked | Various Individualised Funding:  1 - Shared Management Model and  2 - Community Living Fund | Shared Management Model (SMM) allowed people to directly employ support workers as long as a service provider held the funds and managed the wages and taxes for an agreed fee. Community Living Fund (CLF) provided support to enable the person to live with their family or live independently in the community rather than in residential care. | Australia  English | Qualitative study based on in-depth interviews | 11 respondents including: 2 PwD, 3 gov’t administrators, 4 CEOs of disability service, 1 support worker & 1 advocate | Cognitive and Physical | Not reported | National Disability Insurance Scheme (NDIS) | Data were summarised under key themes with extensive use of direct quotes.  RWC: 1,888 |
| O’Brien (2015) | NP  Not Linked | Direct Payment | Direct Payment is a payment that individuals receive to purchase their own support. PwD must set up an individual company in order to receive the direct payment. They are supported by a disability led organisation with initial-set up, business registration, tax returns etc. An informal support network provides longer-term support. | Ireland  English | Case studies involving mixed methods: Skype (online video-call) interviews, 1 open-ended questionnaire and 1 face-to-face interview. | 8 (- 1 ineligible) = 7 | Physical / Sensory 88% (n=7)  Intellectual (n=1) | Age Range:  12 - 64  Female: 14% (n =1)  Ethnic/racial minority: 0% (n=0) | Health Service Executive | Data were summarised under key themes with extensive use of direct quotes  RWC: 6,403 |
| Fleming (2016a)  Linked  Fleming (2016) | P  Linked to 1 additional title - NP | Individualised funding including 3 brokerage models and 1 Direct Payment (DP) | A DP involves funds being given directly to the PwD, who then self-manages this money to meet their individual needs and life circumstances. This may include the employment of a personal assistant to help with everyday tasks and/ or the purchase of services from private, voluntary or community service provider organisations. A brokerage model provides for a similar amount of freedom for the PwD around choice and control of services utilised, but the broker takes responsibility for administrative tasks, and also offers support, guidance and information to enable the person to successfully plan, arrange and manage their support services or care plans. | Ireland  English | 4 Qualitative case studies involving document analysis, secondary qual. data analysis, in-depth interviews and a participatory workshop. | 44 individuals  (20 PwD, 12 staff and 12 advocates) | Intellectual and developmental, physical, sensory, mental health | Of PwD:  Age Range:  Adults  Female: 45% (n =9)  Ethnic/racial minority: 0% (n=0) | All 4 pilots funded by voluntary organisation with 2 accessing ongoing funding from the Health Service Executive | Data were summarised under key themes with extensive use of direct quotes. Tables were also used to summarise data.  RWC: 5,740 |
| McGuigan (2016) | P  Not Linked | Direct Payment (DP) | A DP is a means tested cash payment made to individuals who have been assessed as needing services, in lieu of social service provision. DP allow PwD to avail of care, which they can tailor to their needs, and to source that care themselves rather than depend upon existing statutory or traditional providers. | Northern Ireland  English | Mixed methods including Qualitative interviews and Cross-sectional survey | 317  Proposed sample 10%  N = 30  (2 DP users and 28 informal carers implementing PB on behalf of family member) | Learning 40% (n=12), physical 27% (n=8), mental health 7%(n=2), 5 older (n=5) (65+) and 3 <18 | Of PwD:  Age Range:  <18-65+  Female: 37% (n = 11)  Ethnic/racial minority: Not reported | Local Authority | Data were summarised using free text with extensive use of direct quotes.  RWC: 1,851 |

C – Control / I – Intervention

P – Published in peer reviewed journal / NP – Not published in peer reviewed journal

Linked – Linked to other identified titles / Not Linked – Not linked to other identified titles

PA – Personal Assistant

PwD - Person(s) with a lifelong Disability /Dementia

RR – Response Rate

RWC: Results Word Count

*Data for the minors and the older cohort [65+ (2 sites) / 3-17 years and 60+ (1 site)] were excluded. Older cohort was excluded as there was no way to determine who had a life-long disability and who was receiving age-related home support.

** Uncontrolled pre-post longitudinal study not included. Control only used at time 3.

Adams, L., & Godwin, L. (2008). *Employment Aspects and Workforce Implications of Direct Payments*. Retrieved from Leeds:

Alakeson, V. (2007). *The contribution of self-direction to improving the quality of mental health services*. Retrieved from Washington DC, USA:

Blumberg, E. R., Ferguson, P. M., & Ferguson, D. L. (2000). Slidin' into home: supporting self-determination through personal support agents and service brokerage strategies. *Journal of Vocational Rehabilitation, 15*(2/3), 111-119 119p.

Bola, M., Coldham, T., & Robinson, Z. (2014). A study of personalisation and the factors affecting the uptake of personal budgets by mental health service users in the UK-A research study commissioned by MIND.

Breda, J., Van Landeghem, C., Claessens, D., Vandervelden, M., Geerts, J., & Schoemaekers, D. (2004). *Drie jaar later: evaluatie van het PAB-gebruik Eindrapport* Retrieved from Antwerp, Belgium:

Brown, R., Carlson, B. L., Dale, S., Foster, L., Phillips, B., & Schore, J. (2007). *Cash and Counseling: Improving the Lives of Medicaid Beneficiaries Who Need Personal Care or Home- and Community-Based Services - Final Report*. Retrieved from Princeton, NJ, USA:

Buchanan, A., Peterson, S., & Falkmer, T. (2014). A qualitative exploration of the recovery experiences of consumers who had undertaken shared management, person-centred and self-directed services. *International Journal of Mental Health Systems, 8*(1).

Butler, L. (2006). *Family voices: An exploration of the benefits families imagine when choosing supports for their adult son or daughter with developmental disabilities.* (MR29048 M.A.), Royal Roads University (Canada), Ann Arbor. Retrieved from <http://search.proquest.com/docview/304914274?accountid=12309>

<http://fh6xn3yd3x.search.serialssolutions.com/?ctx_ver=Z39.88-2004&ctx_enc=info:ofi/enc:UTF-8&rfr_id=info:sid/ProQuest+Dissertations+%26+Theses+A%26I&rft_val_fmt=info:ofi/fmt:kev:mtx:dissertation&rft.genre=dissertations+%26+theses&rft.jtitle=&rft.atitle=&rft.au=Butler%2C+Louise&rft.aulast=Butler&rft.aufirst=Louise&rft.date=2006-01-01&rft.volume=&rft.issue=&rft.spage=&rft.isbn=9780494290484&rft.btitle=&rft.title=Family+voices%3A+An+exploration+of+the+benefits+families+imagine+when+choosing+supports+for+their+adult+son+or+daughter+with+developmental+disabilities&rft.issn=&rft_id=info:doi/>

<http://fh6xn3yd3x.search.serialssolutions.com/?genre=article&sid=ProQ:&atitle=Family+voices%3A+An+exploration+of+the+benefits+families+imagine+when+choosing+supports+for+their+adult+son+or+daughter+with+developmental+disabilities&title=Family+voices%3A+An+exploration+of+the+benefits+families+imagine+when+choosing+supports+for+their+adult+son+or+daughter+with+developmental+disabilities&issn=&date=2006-01-01&volume=&issue=&spage=&author=Butler%2C+Louise> ProQuest Dissertations & Theses A&I database.

Caldwell, J. (2007). Experiences of families with relatives with intellectual and developmental disabilities in a consumer-directed support program. *Disability and Society, 22*(6), 549-562. doi:10.1080/09687590701560139

Caldwell, J. A. (2005). *Consumer-directed family support: Experiences of families with adults with developmental disabilities.* (3199940 Ph.D.), University of Illinois at Chicago, Health Sciences Center, Ann Arbor. Retrieved from <http://search.proquest.com/docview/305377492?accountid=12309>

<http://fh6xn3yd3x.search.serialssolutions.com/?ctx_ver=Z39.88-2004&ctx_enc=info:ofi/enc:UTF-8&rfr_id=info:sid/ProQuest+Dissertations+%26+Theses+A%26I&rft_val_fmt=info:ofi/fmt:kev:mtx:dissertation&rft.genre=dissertations+%26+theses&rft.jtitle=&rft.atitle=&rft.au=Caldwell%2C+Joseph+A&rft.aulast=Caldwell&rft.aufirst=Joseph&rft.date=2005-01-01&rft.volume=&rft.issue=&rft.spage=&rft.isbn=9780542465079&rft.btitle=&rft.title=Consumer-directed+family+support%3A+Experiences+of+families+with+adults+with+developmental+disabilities&rft.issn=&rft_id=info:doi/>

<http://fh6xn3yd3x.search.serialssolutions.com/?genre=article&sid=ProQ:&atitle=Consumer-directed+family+support%3A+Experiences+of+families+with+adults+with+developmental+disabilities&title=Consumer-directed+family+support%3A+Experiences+of+families+with+adults+with+developmental+disabilities&issn=&date=2005-01-01&volume=&issue=&spage=&author=Caldwell%2C+Joseph+A> ProQuest Dissertations & Theses A&I database.

Campbell, N., Cockerell, R., Porter, S., Strong, S., Ward, L., & Williams, V. (2011). *Independent Living Strategy Support planning and brokerage: Final report from the support planning and brokerage demonstration project;* . Retrieved from Bristol, UK:

Carlson, B. L., Foster, L., Dale, S. B., & Brown, R. (2007). Effects of cash and counseling on personal care and well-being. *Health Services Research, 42*(1 II), 467-487.

Carmichael, A., & Brown, L. (2002). The future challenge for Direct Payments. *Disability & Society, 17*(7), 797-808. doi:10.1080/0968759022000039082

Carmichael, A., Evans, C., & Brown, L. (2001). A Best Value Review of Direct Payments in Wiltshire - Executive Summary.

Clewett, N., Hamilton, S., Manthorpe, J., Pinfold, V., Szymczynska, P., Tew, J., & Larsen, J. (2015). How can the benefits of personal budgets for people with mental illness be sustained after the payments stop? *Research, Policy and Practice, 31*(2), 105-126.

Coles, B. (2015). A 'Suitable Person': an 'insider' perspective. *British Journal of Learning Disabilities, 43*(2), 135-141 137p. doi:10.1111/bld.12125

Conroy, J. W., Brown, M., Fullerton, A., Beamer, S., Garrow, J., & Boisot, T. (2002). *Independent Evaluation of California’s Self-Determination Pilot Projects;* . Retrieved from Narberth, PA, USA:

Coyle, D. (2009). Recovery Budgets in a Mental Health Service. *Evaluating recovery budgets for people accessing an early intervention service and the imapct of working with self-‐directed services on the team members within a North West of England NHS Trust. Liverpool: Merseycare NHS Trust*.

Coyle, D. (2011). Impact of person-centred thinking and personal budgets in mental health services: reporting a UK pilot. *Journal of Psychiatric & Mental Health Nursing, 18*(9), 796-803 798p. doi:10.1111/j.1365-2850.2011.01728.x

Dale, S., & Brown, R. (2005). *The effect of cash and counseling on Medicaid and Medicare costs : findings for adults in three states*. Retrieved from <https://aspe.hhs.gov/sites/default/files/pdf/74166/3stcost.pdf>

Dale, S., Brown, R., & Phillips, B. (2004). *Does Arkansas’ Cash & Counseling Affect Service Use and Public Costs?* . Retrieved from Princeton, NJ: <https://aspe.hhs.gov/sites/default/files/pdf/73316/ARsupc.pdf>

Dale, S., Brown, R., & Phillips, B. (2004a). *Does Arkansas’ Cash and Counseling Affect Service Use and Public Costs? Final Report* (8349-102). Retrieved from Princeton, NJ, USA:

Dale, S., & Brown, R. S. (2007). How Does Cash and Counseling Affect Costs? *Health Services Research, 42*(1p2), 488-509. doi:<http://dx.doi.org/10.1111/j.1475-6773.2006.00680.x>

Daly, G., Roebuck, A., Dean, J., Goff, F., Bollard, M., & Taylor, C. (2008). Gaining independence: an evaluation of service users' accounts of the individual budgets pilot. *Journal of Integrated Care, 16*(3), 17-25 19p.

Dawson, C. (2000). *Independent successes: Implementing direct payments*. Retrieved from York, UK:

Dimitriadis, L., Laurie, D., Lane, J., & Lyall, M. (2007). *Evaluation of the Direct Payments Project - Final Report*. Retrieved from Melbourne, Australia:

Dinora, P. (2008). Self-Determination Realized? Consumer direction: A case study of Virginia.

Eckert, J. K., San Antonio, P. M., & Siegel, K. B. (2002). *The Cash and Counseling Qualitative Study: Stories from the Independent Choices Program in Arkansas*. Retrieved from Baltimore, USA:

Emslie, A., Ifkovich, C., Lowe, L., & Lawson, H. (2005). *Evaluation of Support & Choice Implementation*. Retrieved from Victoria, Australia:

Eost-Telling, C. (2010). Final Report of the Evaluation of the Self Directed Support Pilot. *Chester: University of Chester*.

Evans, C., & Brown, L. (2002). *A user-controlled Best Value Review of direct payments*. Retrieved from York, UK:

Fleming, P. (2016). How Personal Budgets Are Working in Ireland: Evaluating the Implementation

of Four Individualised Funding Initiatives for People With a Disability in Ireland. Retrieved from [www.genio.ie/personal-budgets](http://www.genio.ie/personal-budgets)

Fleming, P., McGilloway, S., & Barry, S. (2016a). The successes and challenges of implementing individualised funding and supports for disabled people: an Irish perspective. *Disability & Society, 31*(10), 1369-1384. doi:10.1080/09687599.2016.1261692

Foster, L., Brown, R., Phillips, B., Schore, J., & Lepidus Carlson, B. (2003). *Does Consumer Direction Affect the Quality of Medicaid Personal Assistance in Arkansas? Final Report*. Retrieved from <http://www.mathematica-mpr.com/~/media/publications/PDFs/condirect.pdf>

Glendinning, C., Arksey, H., Jones, K., Moran, N., Netten, A., & Rabiee, P. (2009). *The Individual Budgets Pilot Projects: Impact and Outcomes for Carers*. Retrieved from York, UK:

Glendinning, C., D., C., J., F., S., J., K., J., M., K., . . . Wilberforce, M. (2008). *Evaluation of the Individual Budgets Pilot Programme: Final Report* (9781871713640). Retrieved from <http://php.york.ac.uk/inst/spru/pubs/1119/>

Glendinning, C., Halliwell, S., Jacobs, S., Rummery, K., & Tyrer, J. (2000a). Bridging the gap: using direct payments to purchase integrated care. *Health & Social Care in the Community, 8*(3), 192-200 199p.

Glendinning, C., Halliwell, S., Jacobs, S., Rummery, K., & Tyrer, J. (2000). *Buying Independence: using direct payments to purchase integrated health and social services*. Retrieved from Manchester, UK:

Glendinning, C., Halliwell, S., Jacobs, S., Rummery, K., & Tyrer, J. (2000b). New kinds of care, new kinds of relationships: how purchasing services affects relationships in giving and receiving personal assistance. *Health & Social Care in the Community, 8*(3), 201-211 211p.

Glendinning, C., Mitchell, W., & Brooks, J. (2015). Ambiguity in practice? Carers' roles in personalised social care in England. *Health & Social Care in the Community, 23*(1), 23-32 10p. doi:10.1111/hsc.12123

Glendinning, C., Moran, N., Challis, D., Fernández, J.-L., Jacobs, S., Jones, K., . . . Stevens, M. (2011). Personalisation and partnership: competing objectives in English adult social care? The individual budget pilot projects and the NHS. *Social Policy and Society, 10*(02), 151-162.

Gross, J. M. S., Wallace, L., Blue-Banning, M., Summers, J. A., & Turnbull, A. (2013). Examining the Experiences and Decisions of Parents/Guardians: Participant Directing the Supports and Services of Adults With Significant Intellectual and Developmental Disabilities. *Journal of Disability Policy Studies, 24*(2), 88-101. doi:10.1177/1044207312439102

Hamilton, S., Manthorpe, J., Szymczynska, P., Clewett, N., Larsen, J., Pinfold, V., & Tew, J. (2015). Implementing personalisation in integrated mental health teams in England. *Journal of Interprofessional Care, 29*(5), 488-493. doi:10.3109/13561820.2015.1035777

Hamilton, S., Szymczynska, P., Clewett, N., Manthorpe, J., Tew, J., Larsen, J., & Pinfold, V. (2015a). The role of family carers in the use of personal budgets by people with mental health problems. *Health and Social Care in the Community*. doi:10.1111/hsc.12286

Hamilton, S., Tew, J., Szymczynska, P., Clewett, N., Manthorpe, J., Larsen, J., & Pinfold, V. (2015b). Power, choice and control: How do personal budgets affect the experiences of people with mental health problems and their relationships with social workers and other practitioners? *British Journal of Social Work*, bcv023.

Hatton, C., & Waters, J. (2011). The national personal budget survey. *Lancaster: Lancaster University*.

Hatton, C., & Waters, J. (2013). *Second National Personal Budget Survey launched - In Control*. Retrieved from Lancaster University: <http://www.in-control.org.uk/media/154591/poetnationalreport.pdf>

Holman, A., & Bewley, C. (1999). Funding Freedom 2000: People with learning difficulties using Direct Payments (pp. 108). London, UK: Values in Action.

Homer, T., & Gilder, P. (2008). *A Review of Self Directed Support in Scotland*. Retrieved from Edinburgh, Scotland:

Jepson, M., Laybourne, A., Williams, V., Cyhlarova, E., Williamson, T., & Robotham, D. (2015). Indirect payments: when the Mental Capacity Act interacts with the personalisation agenda. *Health & Social Care in the Community*, n/a-n/a. doi:10.1111/hsc.12236

Jones, A., Purcal, C., Meltzer, A., Lutz, D., Fisher, K., Robinson, S., . . . Kayess, R. (2015). *Supported Accommodation Evaluation Framework (SAEF): individual packages*. Retrieved from <http://apo.org.au/resource/supported-accommodation-evaluation-framework-saef-individual-packages>

Jones, K., Netten, A., Fernández, J., Knapp, M., Challis, D., Glendinning, C., . . . Wilberforce, M. (2012). The impact of individual budgets on the targeting of support: Findings from a national evaluation of pilot projects in England. *Public Money and Management, 32*(6), 417-424. doi:10.1080/09540962.2012.728781

Jordan, C. (2004). *Direct payments in action: Implementation by social services departments in England*. Retrieved from London:

Junne, J., & Huber, C. (2014). The risk of users’ choice: exploring the case of direct payments in German social care. *Health, Risk & Society, 16*(7/8), 631-648 618p. doi:10.1080/13698575.2014.973836

Kinnaird, L., & Fearnley, K. (2010). *Lets get personal - personalisation and dementia* (ISBN 978 0 948897 63 4). Retrieved from Edinburgh, Scotland: <http://www.alzscot.org/assets/0000/1820/Lets-get-personal.pdf>

Lambert, C., Lister, C., & Keith, J. (2011). *Users of Social Care Personal Budgets*. Retrieved from UK:

Laragy, C., Fisher, K., Purcal, C., & Jenkinson, S. (2015). Australia's individualised disability funding packages: when do they provide greater choice and opportunity? *Asian Social Work and Policy Review, 9*(3), 282-292. doi:<http://researchbank.rmit.edu.au/view/rmit:33612>

Larsen, J., Tew, J., Hamilton, S., Manthorpe, J., Pinfold, V., Szymczynska, P., & Clewett, N. (2015). Outcomes from personal budgets in mental health: Service users experiences in three English local authorities. *Journal of Mental Health, 24*(4), 219-224.

Laybourne, A. H. (2014). Beginning to explore the experience of managing a direct payment for someone with dementia: The perspectives of suitable people and adult social care practitioners. *Dementia (14713012), 15*(1), 125-140 116p. doi:10.1177/1471301214553037

Leahy, S., Ong, N., de Meyrick, C., & Thaler, O. (2010). *Evaluation of the Expansion of the Self-Managed Model in the Community Participation Program*. Retrieved from Sydney, Australia: <http://www.adhc.nsw.gov.au/__data/assets/file/0011/240887/40_Evaluation_of_expansion_of_the_SMM_July2010.pdf>

Leece, J. (2000). It's a matter of choice: making direct payments work in Staffordshire. *Practice, 12*(4), 37-48.

Lepidus Carlson, B., Dale, S., Foster, L., Brown, R., Phillips, B., & Schore, J. (2005). *Effect of consumer direction on adults' personal care and well-being in Arkansas, New Jersey and Florida*. Retrieved from <https://aspe.hhs.gov/sites/default/files/pdf/74171/adultpcw.pdf>

Lord, J., & Hutchison, P. (2008). Individualized funding in Ontario: Report of a Provincial Study. *Journal on Developmental Disabilities, 14*(2), 44-53.

Lord, J., Kemp, K., Dingwall, C., & Hutchison, P. (2006). *Moving Toward Citizenship: A Study of Individualized Funding in Ontario*. Retrieved from Ontario:

Malette, P. H. (1996). *Lifestyle perspectives of persons with disabilities in a person-centered support paradigm.* (NN14788 Ed.D.), The University of British Columbia (Canada), Ann Arbor. Retrieved from <http://search.proquest.com/docview/304323146?accountid=12309>

<http://fh6xn3yd3x.search.serialssolutions.com/?ctx_ver=Z39.88-2004&ctx_enc=info:ofi/enc:UTF-8&rfr_id=info:sid/ProQuest+Dissertations+%26+Theses+A%26I&rft_val_fmt=info:ofi/fmt:kev:mtx:dissertation&rft.genre=dissertations+%26+theses&rft.jtitle=&rft.atitle=&rft.au=Malette%2C+Paul+Hector&rft.aulast=Malette&rft.aufirst=Paul&rft.date=1996-01-01&rft.volume=&rft.issue=&rft.spage=&rft.isbn=9780612147881&rft.btitle=&rft.title=Lifestyle+perspectives+of+persons+with+disabilities+in+a+person-centered+support+paradigm&rft.issn=&rft_id=info:doi/>

<http://fh6xn3yd3x.search.serialssolutions.com/?genre=article&sid=ProQ:&atitle=Lifestyle+perspectives+of+persons+with+disabilities+in+a+person-centered+support+paradigm&title=Lifestyle+perspectives+of+persons+with+disabilities+in+a+person-centered+support+paradigm&issn=&date=1996-01-01&volume=&issue=&spage=&author=Malette%2C+Paul+Hector> ProQuest Dissertations & Theses A&I database.

McGuigan, K., McDermott, L., Magowan, C., McCorkell, G., Witherow, A., & Coates, V. (2016). The impact of Direct Payments on service users requiring care and support at home. *Practice: Social Work in Action, 28*(1), 37-54. doi:10.1080/09503153.2015.1039973

Netten, A., Jones, K., Knapp, M., Fernandez, J. L., Challis, D., Glendinning, C., . . . Wilberforce, M. (2012). Personalisation through Individual Budgets: Does It Work and for Whom? *British Journal of Social Work, 42*(8), 1556-1573 1518p.

Newbronner, L., Chamberlain, R., Bosanquet, K., Bartlett, C., Sass, B., & Glendinning, C. (2011). *Keeping personal budgets personal: learning from the experiences of older people, people with mental health problems and their carers* (1904812538). Retrieved from Great Britain: <http://www.scie.org.uk>

O'Brien, A. (2015). *Direct Payment: the lived experiences of eight individuals in Ireland*. Retrieved from Cork, Ireland: <http://www.ucc.ie/en/scishop/completed/>

Oliver, M., & Zarb, G. (1992). *Greenwich Personal Assistance Schemes - An Evaluation*. Retrieved from Greenwich, London:

Olmstead, J. (1999). *Implementing Self-Determination: Perspectives from Eleven States*. Retrieved from Washington, D.C., USA:

Pearson, C. (2000). *Money talks? direct payments and competing policy discourses. (BL: DXN044935).* (U142897 Ph.D.), University of Glasgow (United Kingdom), Ann Arbor. Retrieved from <http://search.proquest.com/docview/301576955?accountid=12309>

<http://fh6xn3yd3x.search.serialssolutions.com/?ctx_ver=Z39.88-2004&ctx_enc=info:ofi/enc:UTF-8&rfr_id=info:sid/ProQuest+Dissertations+%26+Theses+A%26I&rft_val_fmt=info:ofi/fmt:kev:mtx:dissertation&rft.genre=dissertations+%26+theses&rft.jtitle=&rft.atitle=&rft.au=Pearson%2C+C&rft.aulast=Pearson&rft.aufirst=C&rft.date=2000-01-01&rft.volume=&rft.issue=&rft.spage=&rft.isbn=&rft.btitle=&rft.title=Money+talks%3F+direct+payments+and+competing+policy+discourses.+%28BL%3A+DXN044935%29&rft.issn=&rft_id=info:doi/>

<http://fh6xn3yd3x.search.serialssolutions.com/?genre=article&sid=ProQ:&atitle=Money+talks%3F+direct+payments+and+competing+policy+discourses.+%28BL%3A+DXN044935%29&title=Money+talks%3F+direct+payments+and+competing+policy+discourses.+%28BL%3A+DXN044935%29&issn=&date=2000-01-01&volume=&issue=&spage=&author=Pearson%2C+C> ProQuest Dissertations & Theses A&I database.

Peterson, S., Buchanan, A., & Falkmer, T. (2014). The impact of services that offer individualised funds, shared management, person-centred relationships, and self-direction on the lived experiences of consumers with mental illness. *International Journal of Mental Health Systems, 8*(1).

Phillips, B. S., Barbara. (2002). Moving to IndependentChoices: The Implementation of the Cash and Counseling Demonstration in Arkansas. *Princeton, NJ: Mathematica Policy Research, Inc*.

Priestley, M., Riddell, S., Jolly, D., Pearson, C., Williams, V., Barnes, C., & Mercer, G. (2010). Cultures of welfare at the front line: implementing direct payments for disabled people in the UK. *Policy & Politics, 38*(2), 307-324. doi:<http://dx.doi.org/10.1332/030557309X477956>

Priestley, M. P., Charlotte; Riddell, Sheila; Jolly, Debbie. (2004). *Disabled People and Direct Payments in the UK: preliminary analysis of key informant interviews;* . Retrieved from Leeds, UK: <http://pf7d7vi404s1dxh27mla5569.wpengine.netdna-cdn.com/files/2011/10/Key-Informant-Summary.pdf>

Rabiee, P., Moran, N., & Glendinning, C. (2009). Individual budgets: lessons from early users' experiences. *British Journal of Social Work, 39*(5), 918-935 918p.

Rees, K. (2013). It’s not just about the support: Exploring the ways in which family members and people with disabilities evaluate their self-directed/self-managed arrangements. *Queensland: Gitana Consulting and Training Services*.

Riddell, S., Ahlgren, L., Pearson, C., Williams, V., Watson, N., & MacFarlane, H. (2006). *The Implementation of Direct Payments for People Who Use Care Services*. Retrieved from Glasgow, Scotland:

Ridley, J., Spandler, H., Rosengard, A., Little, S., Cornes, M., Manthorpe, J., . . . Gray, B. (2011). *Evaluation of Self-Directed Support Test Sites in Scotland*. Retrieved from Edinburgh, Scotland:

Rogers, L., Ockwell, C., Whittingham, J., & Wilson, J. (2009). *SELF DIRECTED SUPPORT FOR MENTAL HEALTH SERVICE USERS IN WEST SUSSEX*. Retrieved from West Sussex, UK:

Rosenberg, L., Williams, E. M., & Sievert, A. L. (2005). *Consumer directed support: lessons learned from Wisconsin’s Family Care Program*. Retrieved from Wisconsin, USA:

Rummery, K., Bell, D., Bowes, A., Dawson, A., & Roberts, E. (2012). *Counting The Cost of Choice and Control: Evidence For The Costs of Self-Directed Support In Scotland*. Retrieved from Edinburgh, Scotland:

San Antonio, P. M., Eckert, K. J., Niles, K. J., & Siegel, K. B. (2003). *THE CASH AND COUNSELING QUALITATIVE STUDY: STORIES FROM THE PERSONAL PREFERENCE PROGRAM IN NEW JERSEY*. Retrieved from Baltimore, USA:

San Antonio, P. M., & J., N. K. (2005). *THE CASH AND COUNSELING QUALITATIVE STUDY: STORIES FROM THE CONSUMER-DIRECTED CARE PROGRAM IN FLORIDA*

Retrieved from Baltimore, USA

San Antonio, P. M., Simon-Rusinowitz, L., Loughlin, D., Eckert, J. K., & Mahoney, K. J. (2007). Case histories of six consumers and their families in Cash and Counseling. *Health Services Research, 42*(1P2), 533-549 517p.

Sanderson, H., Duffy, S., Poll, C., & Hatton, C. (2006). In control: the story so far. *Journal of Integrated Care, 14*(4), 3-13 11p.

Sayles Wallace, L. R. (2009). *Capturing the pioneer spirit: Family perspectives on the impact of the participant direction of the Medicaid HCBS Waiver on family members with significant intellectual disability.* (AAI3481366). Retrieved from <http://search.proquest.com/docview/1322716785?accountid=12309>

<http://fh6xn3yd3x.search.serialssolutions.com/?ctx_ver=Z39.88-2004&ctx_enc=info:ofi/enc:UTF-8&rfr_id=info:sid/Sociological+Abstracts&rft_val_fmt=info:ofi/fmt:kev:mtx:dissertation&rft.genre=dissertations+%26+theses&rft.jtitle=&rft.atitle=&rft.au=Wallace%2C+Luchara+R.+Sayles.&rft.aulast=Wallace&rft.aufirst=Luchara+R.&rft.date=2012-01-01&rft.volume=&rft.issue=&rft.spage=&rft.isbn=9781124998527&rft.btitle=&rft.title=Capturing+the+pioneer+spirit%3A+Family+perspectives+on+the+impact+of+the+participant+direction+of+the+Medicaid+HCBS+Waiver+on+family+members+with+significant+intellectual+disability&rft.issn=&rft_id=info:doi/>

<http://fh6xn3yd3x.search.serialssolutions.com/?genre=article&sid=ProQ:&atitle=Capturing+the+pioneer+spirit%3A+Family+perspectives+on+the+impact+of+the+participant+direction+of+the+Medicaid+HCBS+Waiver+on+family+members+with+significant+intellectual+disability&title=Capturing+the+pioneer+spirit%3A+Family+perspectives+on+the+impact+of+the+participant+direction+of+the+Medicaid+HCBS+Waiver+on+family+members+with+significant+intellectual+disability&issn=&date=2012-01-01&volume=&issue=&spage=&author=Wallace%2C+Luchara+R.+Sayles>. Sociological Abstracts database.

Secker, J., & Munn-Giddings, C. S., Tim. (2011). *Evaluation of the Essex mental health personal budgets pilot: interim report*. Retrieved from <http://arro.anglia.ac.uk/253502/>

Shaw, S. (2008). *Deafblind people and families’ experiences of direct payments*. Retrieved from London:

Sheikh, S., Vanson, T., Comber, N., & Watts, R. (2012). Longitudinal study of Personal Budgets for Adult Social Care in Essex Final report: London: Office for Public Management.

Shen, C., Smyer, M., Mahoney, K. J., Simon-Rusinowitz, L., Shinogle, J., Norstrand, J., . . . del Vecchio, P. (2008). Consumer-directed care for beneficiaries with mental illness: lessons from New Jersey's Cash and Counseling program. *Psychiatric Services, 59*(11), 1299-1306 1298p.

Simon-Rusinowitz, L., Schwartz, A. J., Loughlin, D., Sciegaj, M., Mahoney, K. J., & Donkoh, Y. (2014). Where Are They Now? Cash and Counseling Successes and Challenges Over Time. *Care Management Journals, 15*(3), 104-110 107p. doi:10.1891/1521-0987.15.3.104

Smith, G., Taub, S., Heaviland, M., Bradley, V., & Cheek, M. (2001). *Making Person Centered Supports a Reality: The Equality State’s Experience*. Retrieved from Cambridge, MA:

Speed, I. (2006). Direct payments: what needs to change? *Journal of Integrated Care, 14*(6), 19-22 14p.

Stainton, T., & Boyce, S. (2004). 'I have got my life back': Users' experience of direct payments. *Disability & Society, 19*(5), 443-454. doi:10.1080/0968759042000235299

Tew, J., Larsen, J., Hamilton, S., Manthorpe, J., Clewett, N., Pinfold, V., & Szymczynska, P. (2015). 'And the stuff that I'm able to achieve now is really amazing': The potential of personal budgets as a mechanism for supporting recovery in mental health. *British Journal of Social Work, 45*, i79-i97. doi:10.1093/bjsw/bcv097

Vinton, L. (2010). Caregivers' perceptions of a consumer-directed care program for adults with developmental disabilities. *Journal of Family Social Work, 13*(3), 208-226 219p. doi:10.1080/10522151003756052

Walker, P., Taylor, S., Searl, J., Shoultz, B., Hulgin, K., Harris, P., & Handley, M. (1996). *Evaluation of the Self-Directed Personal Services Program Operated through Enable.* Retrieved from New York:

Waters, J., & Chris, H. (2014). *Third National Personal Budget Survey Experiences of personal budget holders and carers across adult social care and health*. Retrieved from <http://www.in-control.org.uk/media/168205/third%20national%20personal%20budget%20survey%20oct2014.pdf>

Williams, B., & Tyson, A. (2010). Self-direction, place and community---re-discovering the emotional depths: a conversation with social workers in a London borough. *Journal of Social Work Practice, 24*(3), 319-333 315p. doi:10.1080/02650533.2010.500129

Williams, V. (2006). The views and experiences of direct payments users. In C. Pearson (Ed.), *Direct payments and personalisation of care* (Vol. 2). Edinburgh: Dunedin Academic.

Wilson, B., & Pickin, H. (2010). *Findings from the Personal Budgets Survey*. Retrieved from Cheshire UK:

Witcher, S., Stalker, K., Roadburg, M., & Jones, C. (2000). *Direct Payments: The Impact on Choice and Control for Disabled People* (1 84268 576 7). Retrieved from <http://www.gov.scot/Publications/2000/10/2f2d68f2-4138-417a-9755-17e1148928bc>

Young, H. M., & Sikma, S. K. (2003). Self-directed care: an evaluation. *Policy, Politics & Nursing Practice, 4*(3), 185-195 111p.

Zarb, G., & Nadash, P. (1994). *Cashing in on Independence - Comparing the costs and benefits of cash & services*. Retrieved from Derbyshire, UK:
