# Supplementary material for: Individualized funding interventions to improve health and social care outcomes for people with a disability: A mixed‐methods systematic review
Source: Campbell Syst Rev. 2019 Jul 19;15(1-2):e1008. doi: 10.4073/csr.2019.3 (PMC8356501; doi:10.4073/csr.2019.3)
Supplement: Supplementary file 4 — Supporting information [file CL2-15-e1008-s004.docx]

# Appendix 4: Quantitative Study Characteristics

| **First Author (year)** | **Pub Status**  **(linked)** | **Program Name** | **Intervention Description** | **Country** | **Design** | **N** | **Type of disability** | **Sample Characteristics** | **Funding Source** | **Control**  **Condition** |
| --- | --- | --- | --- | --- | --- | --- | --- | --- | --- | --- |
| Beatty (1998) | P  Not Linked | Consumer directed personal assistant services (PAS) | The personal assistant is accountable to the consumer, not to a supervising nurse or agency with the intended purpose of providing the PwD control and independence while achieving full integration within the community. | USA | Quasi-experimental non-randomised controlled longitudinal survey over a 3 year period. The control group were on a waiting list to receive PAS.  Surveys were conducted by mail and telephone.  1 outcome of interest reported | 92  60 (1)  32 (C)  RR: 100% (secondary use of existing data) | Physical | Average age: 42 (I)  44 (C)  Female: 47% (I)  42% (C)  Ethnic/racial minority:  20% (I)  15% (C) | Virginia's PAS program (I)  Medicaid Waivers and usually (fully or in part) self-funded (C) | In receipt of personal assistance services that were not consumer directed. |
| Benjamin (2000)  Linked:  Doty (1999) | P  Linked:  1 additional quant. title  NP (non-technical report) | Consumer directed In-Home Supportive services | In-Home Supportive Services (IHSS) program provides personal care, household, paramedical,  protective supervision, and medical transportation services. Consumer-directed model permits recipients to hire anyone as their provider, including family members. | USA | Controlled cross-sectional survey of random sample.  Computer assisted telephone interviews.  3 outcomes of interest reported | 1,408  RR: 77.8%  n=1,095  511 (I)  584 (C) | Various | Over 65:  54% (I)  50% (C)  Female:  70% (I)  77% (C)  Ethnic/racial minority:  62% (I)  30% (C) | Primarily by Medicaid | In receipt of in-home support services from a professional agency (not consumer directed) |
| Conroy (2002) | NP  Not Linked | Self-determination pilot projects | Three pilots with varying degrees of intermediary and fiscal coordination. Pilot 1 recruited an in-house coordinator to support the PwD and family in developing a budget and plan. Pilot 2 used existing staff for fiscal support and used service coordinators (sometimes private consultant) for planning. Pilot 3 used 10 independent service brokers to provide a menu of needs-led services. Consultants provided training to PwD and brokers. | USA | Mixed methods  Randomised, controlled before and after study.  1 site had a comparison group (eligible quant data).  Face-to-face interviews focus groups, telephone interviews were used to collect data.  3 outcomes of interest reported | 112  RR: 68.8%  N=77  63 (I)  14 (C)  RR: 68.8% | Mental Health (including various secondary ‘major’ disabilities) | Average age: 25 (I)  28 (C)  Female: 29% (I)  36% (C)  Ethnic/racial minority:  46% (I)  71% (C) | State funds were used on a pilot basis (outside the standard federal waiver program – to avoid associated rigidity) | Involving people who wanted to participate in pilot number 3 (ELARC) but were on the waiting list, receiving standard services. |
| Brown (2007)  Linked:  **Quant:**  Foster (2003)  Dale (2004, 2004, 2005, 2007)  Lepidus (2005)  Carlson (2007)  Shen (2008)  **Qual:**  Phillips (2002, 2006)  Eckert (2002)  Simon- Rusinowitz (2014)  San Antonio (2003, 2005, 2007) | NP  Linked:  15 additional titles including 8 quant. (2 P & 6 NP) and 7 qual. (2 P & 5 NP) | Cash and Counseling - Consumer-directed care | A form of consumer-directed care that provides PwD a monthly allowance to hire workers of their own choosing (including family), and to purchase care-related services and goods (within state guidelines). Designated representatives (family / friends) can help PwD to make care-management decisions. Counseling (advice & guidance) and fiscal intermediary services were offered to support  PWD / representatives. | USA | Mixed methods.  Randomised, controlled before and after study using site visits, program data and surveys. Data collected 9 months after enrolment in intervention. Control group received standard care. Intent-to-treat approach taken to analyses.  Data was collected by telephone interview.  4 outcomes of interest reported | 2287  (18- 60/64 only*)  RR: 86.0%  N=1,966  1,007 (I)  959 (C) | Physical (and in some cases secondary cognitive / develop-mental disability) | Aged 18-39: (I) 48%  (C) 48%  Female: 58% (I)  59% (C)  Ethnic/racial minority:  35% (I)  35% (C) | Medicaid | Most beneficiaries receive traditional agency-based personal  care services (PCS) or agency provided home- and community based services (HCBS) |
| Caldwell (2007)  Linked:  Heller (1999) | P  Linked:  1 additional quant. title  P (journal article) | Consumer-directed program | Individuals with disabilities and their families develop a plan with the assistance of a service facilitator and decide what services and supports are purchased. The state serves as the fiscal agent. Family members could not be hired during study timeframe. | USA | Controlled longitudinal study of a random sample. Data was collected at 3 time periods over a 9 year period (1991 – 2000).  Surveys were conducted by mail.  3 outcomes of interest reported | 369 (Caldwell, ’02)  RR: 24%  n=87  38 (I) – 3 excluded before analysis  49 (C)  RR: 24% | Intellectual disability (66%), Physical (28%), Autism (18%) | Average age: 37 (I)  28 (C)  Female: 50% (I)  49% (C)  Ethnic/racial minority:  18% (I)  33% (C) | Initially Illinois Home Based Support Services  Program | People on waiting list. Data for control group collected after 4 years (Heller, ’99) & after 9 years (Caldwell ‘07) |
| Glendinning (2008)  Linked:  **Quant:**  Jones (2012)  Netten (2012)  **Qual:**  Glendinning (2009, 2011)  Rabiee (2009) | NP  Linked:  5 additional titles including 2 quant. (2 P) and 3 qual. (2 P & 1 NP | Individual Budgets (IBs) | IBs bring together the resources from a number of different funding streams. PwD should have a greater role for self-assessment; greater opportunities for self-definition of needs and desired outcomes; and increased opportunities for PwD to determine those outcomes are achieved. PwD should know how much money they will receive, how much services cost, and they should be offered support in planning. Family members that did not live with the PwD could be hired. | England | Mixed method involving:  Randomised controlled trial involving 13 pilots. The control group received standard services. Baseline outcomes data captured before intervention commenced and again after 6 months.  Qualitative in-depth interviews with PwD and other stakeholders  91% of interviews with PwD completed face-to-face. 9% by telephone.  6 outcomes of interest reported | 1,336  RR: 72%  n-959  510 (I)  449 (C)  - 42 cases excluded before analysis | Physical (34%), Intellectual (25%), Mental health (14%) | Average age:  57 (18-75+)  Female:  56%  Ethnic/racial minority:  8%  I / C breakdown not available | Funding was sourced from various funding streams and this varied between the 13 study sites. Types of funding streams included ‘Access to work’ & ‘Independent Living Fund’. | The control group would continue with services as usual for a period of 6 months, after which time they could avail of an individual budget. |
| Woolham (2013) | P  Not Linked | Personal Budget (PB) | Based on a self-assessment, an ‘indicative budget’ is given to PwD at an early stage in the process to create a support plan (with support from others if needed). PBs can be used to buy a wide range of services once they are safe and legal. Plans must be approved by the local authority. | England | Controlled cross-sectional survey of random sample.  Self-completed postal questionnaire. Telephone assistance available where necessary.  3 outcomes of interest reported | 1049  RR: 53%  n=558  180 (I)  378 (C)  Older people were excluded  126 (I)  276 (C) | Physical (32%), Intellectual (34%), Mental health (5%) | Average age: 52 (I)  55 (C)  Female: 66% (I)  64% (C)  Ethnic/racial minority:  7% (I)  6% (C) | Social Services Funding | A random sample of ‘traditional’ service users. |

C – Control / I – Intervention

P – Published in peer reviewed journal / NP – Not published in peer reviewed journal

Linked – Linked to other identified titles / Not Linked – Not linked to other identified titles

PwD - Person(s) with a lifelong Disability /Dementia

RR – Response Rate

*Data for the minors and the older cohort [65+ (2 sites) / 3-17 years and 60+ (1 site)] were excluded. Older cohort was excluded as there was no way to determine who had a life-long disability and who was receiving age-related home support.

** Uncontrolled pre-post longitudinal study not included. Control only used at time 3.

Beatty, P. W., Richmond, G. W., Tepper, S., & DeJong, G. (1998). Personal assistance for people with physical disabilities: consumer-direction and satisfaction with services. *Archives of Physical Medicine & Rehabilitation, 79*(6), 674-677 674p.

Benjamin, A. E., Matthias, R., & Franke, T. M. (2000). Comparing consumer-directed and agency models for providing supportive services at home. *Health Services Research, 35*(1 II), 351-366.

Brown, R., Carlson, B. L., Dale, S., Foster, L., Phillips, B., & Schore, J. (2007). *Cash and Counseling: Improving the Lives of Medicaid Beneficiaries Who Need Personal Care or Home- and Community-Based Services - Final Report*. Retrieved from Princeton, NJ, USA:

Caldwell, J., Heller, T., & Taylor, S. J. (2007). Longitudinal outcomes of a consumer-directed program supporting adults with developmental disabilities and their families. *Intellectual and developmental disabilities, 45*(3), 161-173.

Carlson, B. L., Foster, L., Dale, S. B., & Brown, R. (2007). Effects of cash and counseling on personal care and well-being. *Health Services Research, 42*(1 II), 467-487.

Conroy, J. W., Brown, M., Fullerton, A., Beamer, S., Garrow, J., & Boisot, T. (2002). *Independent Evaluation of California’s Self-Determination Pilot Projects;* . Retrieved from Narberth, PA, USA:

Dale, S., & Brown, R. (2005). *The effect of cash and counseling on Medicaid and Medicare costs : findings for adults in three states*. Retrieved from <https://aspe.hhs.gov/sites/default/files/pdf/74166/3stcost.pdf>

Dale, S., Brown, R., & Phillips, B. (2004). *Does Arkansas’ Cash & Counseling Affect Service Use and Public Costs?* . Retrieved from Princeton, NJ: <https://aspe.hhs.gov/sites/default/files/pdf/73316/ARsupc.pdf>

Dale, S., Brown, R., & Phillips, B. (2004). *Does Arkansas’ Cash and Counseling Affect Service Use and Public Costs? Final Report* (8349-102). Retrieved from Princeton, NJ, USA:

Dale, S., & Brown, R. S. (2007). How Does Cash and Counseling Affect Costs? *Health Services Research, 42*(1p2), 488-509. doi:<http://dx.doi.org/10.1111/j.1475-6773.2006.00680.x>

Doty, P., Benjamin, A. E., Matthias, R. E., & Franke, T. M. (1999). *In-Home Supportive Services for the Elderly and Disabled: A Comparison of Client-Directed and Professional Management Models of Service Delivery*. Retrieved from Washington DC:

Eckert, J. K., San Antonio, P. M., & Siegel, K. B. (2002). *The Cash and Counseling Qualitative Study: Stories from the Independent Choices Program in Arkansas*. Retrieved from Baltimore, USA:

Foster, L., Brown, R., Phillips, B., Schore, J., & Lepidus Carlson, B. (2003). *Does Consumer Direction Affect the Quality of Medicaid Personal Assistance in Arkansas? Final Report*. Retrieved from <http://www.mathematica-mpr.com/~/media/publications/PDFs/condirect.pdf>

Glendinning, C., Arksey, H., Jones, K., Moran, N., Netten, A., & Rabiee, P. (2009). *The Individual Budgets Pilot Projects: Impact and Outcomes for Carers*. Retrieved from York, UK:

Glendinning, C., D., C., J., F., S., J., K., J., M., K., . . . Wilberforce, M. (2008). *Evaluation of the Individual Budgets Pilot Programme: Final Report* (9781871713640). Retrieved from <http://php.york.ac.uk/inst/spru/pubs/1119/>

Glendinning, C., Moran, N., Challis, D., Fernández, J.-L., Jacobs, S., Jones, K., . . . Stevens, M. (2011). Personalisation and partnership: competing objectives in English adult social care? The individual budget pilot projects and the NHS. *Social Policy and Society, 10*(02), 151-162.

Heller, T., Miller, A. B., & Hsieh, K. (1999). Impact of a Consumer-Directed Family Support Program on Adults with Developmental Disabilities and Their Family Caregivers. *Family Relations, 48*(4), 419-427. doi:10.2307/585250

Jones, K., Netten, A., Fernández, J.-L., Knapp, M., Challis, D., Glendinning, C., . . . Wilberforce, M. (2012). The impact of individual budgets on the targeting of support: Findings from a national evaluation of pilot projects in England. *Public Money and Management, 32*(6), 417-424. doi:10.1080/09540962.2012.728781

Lepidus Carlson, B., Dale, S., Foster, L., Brown, R., Phillips, B., & Schore, J. (2005). *Effect of consumer direction on adults' personal care and well-being in Arkansas, New Jersey and Florida*. Retrieved from <https://aspe.hhs.gov/sites/default/files/pdf/74171/adultpcw.pdf>

Netten, A., Jones, K., Knapp, M., Fernandez, J. L., Challis, D., Glendinning, C., . . . Wilberforce, M. (2012). Personalisation through Individual Budgets: Does It Work and for Whom? *British Journal of Social Work, 42*(8), 1556-1573 1518p.

Phillips, B., Mahoney, K. J., & Foster, L. (2006). *Implementation Lessons on Basic Features of Cash & Counseling Programs*. Retrieved from <http://www.appliedselfdirection.com/sites/default/files/ImplementationLessons.pdf>

Phillips, B. S., Barbara. (2002). Moving to IndependentChoices: The Implementation of the Cash and Counseling Demonstration in Arkansas. *Princeton, NJ: Mathematica Policy Research, Inc*.

Rabiee, P., Moran, N., & Glendinning, C. (2009). Individual budgets: lessons from early users' experiences. *British Journal of Social Work, 39*(5), 918-935 918p.

San Antonio, P. M., Eckert, K. J., Niles, K. J., & Siegel, K. B. (2003). *THE CASH AND COUNSELING QUALITATIVE STUDY: STORIES FROM THE PERSONAL PREFERENCE PROGRAM IN NEW JERSEY*. Retrieved from Baltimore, USA:

San Antonio, P. M., & J., N. K. (2005). *THE CASH AND COUNSELING QUALITATIVE STUDY: STORIES FROM THE CONSUMER-DIRECTED CARE PROGRAM IN FLORIDA*

Retrieved from Baltimore, USA

San Antonio, P. M., Simon-Rusinowitz, L., Loughlin, D., Eckert, K. J., & Mahoney, K. J. (2007). Case histories of six consumers and their families in Cash and Counseling. *Health Services Research, 42*(1P2), 533-549 517p.

Shen, C., Smyer, M. A., Mahoney, K. J., Simon-Rusinowitz, L., Shinogle, J., Norstrand, J., . . . del Vecchio, P. (2008). Consumer-directed care for beneficiaries with mental illness: lessons from New Jersey's Cash and Counseling program. *Psychiatric Services, 59*(11), 1299-1306 1298p.

Simon-Rusinowitz, L., Schwartz, A. J., Loughlin, D., Sciegaj, M., Mahoney, K. J., & Donkoh, Y. (2014). Where Are They Now? Cash and Counseling Successes and Challenges Over Time. *Care Management Journals, 15*(3), 104-110 107p. doi:10.1891/1521-0987.15.3.104

Woolham, J., & Benton, C. (2013). The costs and benefits of personal budgets for older people: Evidence from a single local authority. *British Journal of Social Work, 43*(8), 1472-1491. doi:10.1093/bjsw/bcs086
