# Supplementary material for: Individualized funding interventions to improve health and social care outcomes for people with a disability: A mixed‐methods systematic review
Source: Campbell Syst Rev. 2019 Jul 19;15(1-2):e1008. doi: 10.4073/csr.2019.3 (PMC8356501; doi:10.4073/csr.2019.3)
Supplement: Supplementary file 5 — Supporting information [file CL2-15-e1008-s010.docx]

**Appendix 5: Excluded studies**

| **Study** | **Primary reason for exclusion** |
| --- | --- |
| (Abbott & Marriott, 2013) | Intervention- This study focuses on managing one’s finances at a micro level. |
| (Ageing and Aged Care, 2012) | Study Design – Outcomes were measured < 6 months after beginning the intervention. |
| (Alakeson, 2008b) | Empirical Data – Discusses / references intervention of interest but does not collect empirical data |
| (Alakeson, 2008a) | Empirical Data – Discusses / references intervention of interest but does not collect empirical data |
| (Allen, 2008) | Empirical Data – Discusses / references intervention of interest but does not collect empirical data |
| (Anderson, Wiener, & Khatutsky, 2006) | Outcome - Outcomes measured were workforce variables not health and social care outcomes |
| (Arksey & Baxter, 2011) | Population – The study population was primarily people with serious health conditions but unclear if lifelong disability present |
| (Arntz & Thomsen, 2011) | Population – This study relates to frail older people (only) without any indication to a lifelong disability. |
| (ARTD Consultants, 2009) | Population - A large proportion of population was potentially <18. A follow up study where sample older included in review |
| (Barczyk & Lincove, 2010) | Empirical Data – Discusses / references intervention of interest but does not collect empirical data |
| (Barton, 2012) | Intervention - Focus of this study was person-centred-approaches but does not utilise PBs |
| (Baxter, 2006) | Intervention - Focus on individualised supports but the PWD / family do not directly control the funds |
| (Beatty, Adams, & O'Day, 1998) | Empirical Data - This paper discusses the findings of a study published (already captured in the review) |
| (Benjamin, Franke, Matthias, & Park, 1999) | Study Design - The control group was also in receipt of a personal budget but have chosen to have their support workers from an agency rather than a family member. Therefore, the effectiveness was based on type of support worker rather than I.F. |
| (Bond, 1996) | Empirical Data – Not found, however it was a very short paper (2 pages) and abstract reads as an opinion piece rather than fact |
| (Bradley et al., 2001) | Intervention - Explores the concept of self-determination rather than exploring self-determination as a process using individualised funds |
| (Brandon, 1989) | Empirical Data - Not found, however it was a very short periodical (2 pages) and therefore unlikely to report effectiveness data |
| (Breda, Gevers, Vandervelden, & Peeters, 2011) | Study design - This study compares two types of PB. The original and a new version, therefore the control group was not receiving standard care and effectiveness cannot be measured. |
| (Breda, Schoemaekers, Van Landeghem, Claessens, & Geerts, 2006) | Intervention - This study focuses on the dynamic between employer and carer. The outcomes of interest are not reported. |
| (Brewis, 2009) | Empirical Data – Not found, however it was a very short paper (2 pages) and abstract reads as journalistic rather than academic |
| (Bright & Drake, 1999) | Empirical Data – Reporting on data from another study, which was subsequently added to the review (after contacting author) |
| (Broadhurst, Yates, & Mullen, 2012) | Intervention - Study of transition interventions; one of the possible outcomes of this intervention was the receipt of a direct payment but was not the primary focus of the study. |
| (Brown, 1996) | Not Found – This paper was listed on academic databases but could not be located when retrieval attempted. |
| (Caldwell, 2006) | Population – the outcomes of interest are only for the family caregiver, not for the PwD |
| (Caldwell & Heller, 2003) | Study design - there was no control group for this study so effectiveness cannot be measured |
| (Callahan, 2000) | Intervention - The funding in this study was specifically for gaining employment however the PWD did not get to freely determine how to spend the money in order to meet health or social care needs. |
| (Callahan, 2001) | Empirical Data - Discusses / references intervention of interest but does not collect empirical data |
| (E. M. Campbell, Fortune, & Heinlein, 1998) | Intervention - This paper focuses on funding packages to move people from institutional settings to community settings but the intervention was not individualised funding |
| (M. Campbell, 2010) | Intervention – Pre-implementation study assessing knowledge and expectations |
| (Carers UK, 2008) | Study design – Insufficient information on how the data were collected and analysed or the sample of individuals interviewed. |
| (Carr, 2010) | Empirical Data – This was a literature review – references cross-checked |
| (Chopin & Findlay, 2010) | Population – This study does not involve PwD but key informants, however the data was descriptive and not related to implementation experiences. |
| (Christensen, 2006) | Empirical Data - Discusses / references intervention of interest but does not collect empirical data |
| (Clark, Hagglund, & Sherman, 2008) | Study Design – There was no control group in this study, therefore effectiveness cannot be measured |
| (Cloutier, Malloy, Hagner, & Cotton, 2006) | Intervention - The funding in this study was specifically for purchasing employment services however the PWD did not get to freely determine how to spend the money in order to meet health or social care needs. |
| (Cocks & Boaden, 2009) | Intervention – while individual/family directed supports may form part of the intervention being studied, it individualised funding was not a core element of this study |
| (Conroy & Yuskauskas, 1996) | Study design – There was no follow-up data for control group. Author contacted to confirm. (InControl) |
| (Cook, Russell, Grey, & Jonikas, 2008) | Method – There was no control group for the pre/post study |
| (Cook et al., 2010) | Outcome – Qualitative data presented was on the process of piloting and setting up a program. RCT study is discussed but not data presented. |
| (Cornford, 2011) | Empirical Data – This paper is presenting secondary data from another study |
| (Cortis, Meagher, Chan, Davidson, & Fattore, 2013) | Empirical Data – This paper was based on a literature review of existing studies. References cross-checked |
| (Cunningham & Nickson, 2010) | Outcomes – The focus of this paper was on working practices and how individualised funding has impacted the workplace. |
| (Dale & Brown, 2006) | Outcomes – The outcome of interest in this study was ‘facility use’ which was not an outcome of interest. Costs data captured elsewhere in the review. |
| (Davey et al., 2007) | Outcomes - This appears to be a descriptive study, does not report experience of service users or effectiveness data |
| (Davidson, Baxter, Glendinning, & Irvine, 2013) | Population – only 7% of the overall sample appear to have a disability. |
| (Davidson et al., 2012) | Population – only 7% of the overall sample appear to have a disability. |
| (Davies & Morgan, 2010) | Intervention – The focus of this study was the general experience of transitioning to adulthood. Individualised funding was not directly addressed |
| (Dean, Dunn, & Tomchek, 2015) | Outcome – The focus of this study was how Occupational Therapists can support PwD in a more self-directed way |
| (Dew et al., 2012) | Population – This study seems to be primarily focusing on children with a disability (or their parents) |
| (Doty, Kasper, & Litvak, 1996) | Intervention – The focus of this study was not individualised funding but more specifically the processes of self-direction. Moreover there was no control group. |
| (Dougherty & Eggers, 1996) | Intervention - Various models are discussed but none are utilised to form the basis of an effectiveness or experiential study |
| (Doyle, 1995) | Study design – Length of time participants in receipt of individualised funding was not specified. |
| (Duffy, 2007) | Intervention – This study does not look at the effectiveness of individualised funding. |
| (Edwards & Waters) | Study Design – There was insufficient information on the study design and therefore it was unclear where the data was derived from. |
| (Eriksson, 2012) | Empirical Data - Not found but appears to be a presentation based on study captured in the review. |
| (Eriksson, 2014b) | Outcome – This was a pre-implementation study and therefore does not focus on the outcomes of interest |
| (Eriksson, 2014a) | Intervention – This paper focuses on the views of organisational staff, assessing assumptions about what people believe is needed to make individualised funding a success. No intervention is implemented |
| (Evans & Carmichael, 2002) | Empirical Data - This was a process review exploring how people with disabilities were included in an evaluation of personal budgets |
| (Field, McGechie, & King, 2015) | Study Design – While the study was measuring relevant costs data, it was not based on a random or matched sample and was not measuring effectiveness. |
| (J. Fisher, Rayner, & Baines, 2011) | Method – While the study does refer to two case studies from a direct payment pilot project, the findings section is extremely brief and methodological detail is sparse. |
| (K. R. Fisher & Purcal, 2016) | Intervention – The intervention of interest was personalised housing supports, while funding was available for this, the intervention of interest was not individualised funding. |
| (Flynn, 2005) | Study design – The intervention had not been in place for 6 months and 13% had not received intervention yet. Furthermore evidence that children were involved |
| (Forbes et al., 2008) | Intervention – Not the intervention in question. Focus appears to be primary care needs of PwD, availability of services and the unmet needs of carers |
| (Forder et al., 2012) | Population - only 26% of the overall sample appear to have a disability/mental health problem. |
| (L. Foster, Brown, Carlson, Phillips, & Schore, 2000) | Method – This paper only presents data for the intervention group; no data for control group presented. |
| (L. Foster, Brown, Carlson, Phillips, & Schore, 2002) | Method – This paper only presents data for the intervention group; no data for control group presented. |
| (M. Foster, Harris, Jackson, Morgan, & Glendinning, 2006) | Intervention – This was a pre-implementation paper, when assessments were being conducted to assign direct payment |
| (Fox & Kim, 2004) | Method – It was questionable whether this was describing the intervention, nevertheless there was no control group |
| (Friedman, Wamsley, & Conwell, 2015) | Outcome – This study was seeking to determine the extent to which depression predicts voucher use – not health and social care outcomes associated with individualised funding |
| (Fyson, 2012) | Not Found - This paper appeared on several academic databases but was not available once retrieval was attempted. Furthermore the relevance of this paper was questionable at initial title/abstract screen stage |
| (G. Allan Roeher Institute, 1991) | Intervention - This was a pre-implementation study, whereby interviewees are highlighting the need for individualised funding and the potential benefits |
| (Gibson, Brooks, DeMatteo, & King, 2009) | Intervention - This study was focusing on an attendant care service within mainstream housing, however PwD do not have direct control over funding, hiring, firing or who supports them |
| (Gill & Cameron, 2015) | Population – This study was focussing on older people only with no specific mention of lifelong disability |
| (Gill, McCaffrey, Cameron, & Ratcliffe, 2016) | Population – This study was focussing on older people only with no specific mention of lifelong disability |
| (Glasby, 2008) | Population – 61% of the study population has no experience of individualised funding |
| (Graham, 2015) | Outcome – The outcome of interest in this study was the employment relationship (PA) as opposed to health and social care outcomes linked to individualised funding |
| (Gridley, Brooks, & Glendinning, 2014) | Intervention – While 33% of the study population had access to individualised funding this was not the focus of the study |
| (Grootegoed, Knijn, & da Roit, 2010) | Outcomes – The outcomes of interest in this study are the pros and cons of paying a relative and the experience of this new relationship dynamic |
| (Grossman, Kitchener, Mullan, & Harrington, 2007) | Intervention – While some of the study respondents may have had access to individualised funding, this was not the focus of the study |
| (Hagglund, Clark, Farmer, & Sherman, 2004) | Method – Respondents only had to have access to individualised funding for a period of 1 month. |
| (Hall, 2009) | Empirical Data – This was a review of literature. No intervention was implemented in this study |
| (Hall-Lande, Hewitt, Bogenschutz, & LaLiberte, 2012) | Population – The focus of this study are children not adults. |
| (Hammond, 2012) | Empirical data – This was a descriptive paper with anecdotal accounts of several cases |
| (Harding-Price, 2011) | Empirical Data - This paper refers to a pilot evaluation but there was no reference available, no description of sample, how data were collected etc. |
| (Hashemi et al., 2008) | Intervention – The focus of this study appears to be personal assistance services rather than individualised funding. |
| (Hasler & Stewart, 2004) | Empirical Data – This study discusses factors inhibiting implementation but was not based on an empirical data |
| (Hatton et al., 2008) | Study Design - 64% using self-directed supports for less than 6 months. Furthermore there was no control group. |
| (Hay & Waters, 2009) | Method – There was no control group for quantitative element and no methods described for the qualitative data presented. |
| (Head & Conroy, 2005) | Method – There was no control group for this pre-post study with baseline and 3 year follow-up |
| (Heller, Arnold, van Heumen, McBride, & Factor, 2012) | Method – The controlling variable in this study was the type of support worker and the difference this makes to certain outcomes |
| (Heller & Caldwell, 2005) | Method – The controlling variable in this study are out-of-home placements not individualised funding |
| (Henwood, 2008) | Empirical Data – This was a summary of a study already captured in the systematic review |
| (Henwood & Hudson, 2007) | Empirical Data – This was a summary of a study already captured in the systematic review |
| (Hitchen, 2013) | Intervention - This was a pre-implementation study. Mental health users discussing the potential effects of receiving individualised funding |
| (Hitchen, Williamson, & Watkins, 2015) | Intervention - This was a pre-implementation study. Mental health users discussing the potential effects of receiving individualised funding |
| (InControl) | Empirical Data – This paper was more of an advocacy piece than a clearly defined study. |
| (Inoue, Lombe, Putnam, & Mahoney, 2014) | Outcome - This paper was concerned with types of purchases, purchase patterns etc. not health and social care outcomes |
| (Irvine et al., 2011) | Population - Only 7% of the overall sample appear to have a disability. |
| (Isaacson, Cocks, & Netto, 2014) | Intervention – This study focuses on the transition of young people from the family home, but individualised funding was not the main concern. |
| (James, 2008) | Empirical Data – This was a discussion paper and not based on an empirical study |
| (Jensen, 2008) | Intervention – This study was a pre-implementation, scoping exercise to identify what families need from a service provider to support their family member, potential goals etc. |
| (Jones et al., 2010) | Population – This study does not focus on PwD specifically but on the general population of ‘patients’ including a variety of illnesses |
| (Jones et al., 2013) | Population - Only 7% of the overall sample appear to have a disability. |
| (Jones et al., 2011) | Method – There was no control group for the outcome of interest in this study (costs). Furthermore the eligible population was much broader than just PwD |
| (Jones & Netten, 2010) | Method – There was no control group for the outcome of interest in this study (costs). Furthermore the eligible population was much broader than just PwD |
| (Jones et al., 2010) | Population - Only 7% of the overall sample appear to have a disability. |
| (Judd, 2014) | Outcome - The focus of this study was on carers experience and how it impacted their life rather than the health and social care outcomes for the PwD |
| (Kaiser Family Foundation, 2006) | Intervention – The focus of this study was health insurance, not individualised funding |
| (Kassner, Coleman, & Milne, 2008) | Study Design - This was a descriptive quantitative study and there was no control group |
| (Keigher, 2000) | Intervention - The focus of this paper was ‘Personal assistant services’ rather than individualised funding. Furthermore data was not disaggregated by type of funding. |
| (Kelly, 2014) | Outcome – This paper was focussing on care and the meaning of care. Where individualised funding was discussed it was in relation to reconceptualising care |
| (Kendall & Cameron, 2014) | Intervention – This paper was examining the concept of choice and control in self-directed supports. Furthermore, a minority of participants had a disability |
| (Kestenbaum, 1992) | Not found. This appeared to be a spiral bound report available on amazon but upon further investigation, it was not possible to retrieve. |
| (Kim, White, & Fox, 2006) | Method – This was a cross-sectional survey. No baseline data was collect and the control group was unmatched |
| (Laragy, 2010) | Intervention – An intervention was not implemented in this study. Rather interviews regarding social participation with stakeholders in four countries. Unclear how this was measured. |
| (Laragy, David, & Moran, 2015) | Intervention – This paper described a framework for information provision for individualised funding programmes |
| (Larkin, 2015) | Outcome – The outcomes of interest in this paper were the impact on carer–service user relationship and on personal outcomes for the carer. Health and social care outcomes for the PwD not reported |
| (Larsen et al., 2013) | Intervention – An individualised funding intervention was not implemented in this study. It a examining factors that influence the introduction and take-up of personal budgets more generally |
| (Larson, 2006) | Empirical Data – This report was summarising ‘Cash and Counseling’ data already captured in this review |
| (Lavelle, 2009) | Intervention – This was a pre-implementation study examining concerns ahead of implementation. Also unclear if PwD actually participated in the study |
| (Lawson, Pearman, & Waters, 2010) | Study Design – There was no control group for the cross-sectional survey. Methods are not described for the qualitative data, just a series of quotes. |
| (Leece & Leece, 2006) | Outcome - The outcomes are not relevant (i.e. level of disability, financial savings, age, income. Furthermore, this was a descriptive quant study, with non – matched control group. |
| (J. Leece, 2010) | Intervention - The intervention in this study was the direct employment of support workers – the outcome of interest was the effect on the working relationship |
| (J. P. Leece, Sheila, 2010) | Intervention - The intervention in this study was the direct employment of support workers – the outcome of interest was the effect on the working relationship |
| (Lombe, 2016) | Outcome – The outcome of interest in this study was ‘saving behaviour’ not health and social care outcomes |
| (Lombe, Putnam, & Huang, 2008) | Outcome - The outcome of interest in this study was the ability of people receiving personal budgets to save money privately |
| (Lord & Hutchison, 2003) | Empirical Data - The study draws on existing empirical research and grey literature |
| (Low, Chilko, Gresham, Barter, & Brodaty, 2012) | Empirical Data - This was a policy, commentary paper – there are no empirical data |
| (Maglajlic, 1999) | Empirical Data – Not found, however it was a very short paper (2 pages) and abstract seems to focus on general barriers to uptake for people with mental health problems |
| (Mahoney, Desmond, Simon-Rusinowitz, & Squillace, 2002) | Intervention – This was a pre-implementation study looking at the preferences for consumer directed support – none of the participants had availed of consumer directed support. |
| (Mansell, 2010) | Intervention - This was a broad report looking at various aspects of good services for people with high support needs – individualised funding was referenced but not the focus |
| (Manthorpe & Stevens, 2008) | Intervention - This was a pre-implementation study, looking at possible outcomes and possible issues for people in rural areas. |
| (Manthorpe et al., 2010) | Intervention - The focus of this article was solely on training and rolling out training for staff. This does not directly relate to the implementation for PwD. |
| (Matthias, 2003) | Intervention – While the intervention was described as consumer-directed model this was not individualised funding as defined by the intervention, as people are responsible for all aspect of managing support staff but with no financial control |
| (McConkey, Bunting, Ferry, Garcia-Iriarte, & Stevens, 2013) | Intervention – Individualised funding was not the focus of this study with only 2% of participants in receipt of a personal budget |
| (McWilliam et al., 2004) | Intervention – Although the consumer-managed model of case management does reflect characteristics of the intervention of interest, there was no specific mention of individualised funding |
| (Meng, Friedman, Wamsley, Van Nostrand, & Eggert, 2010) | Outcome – Outcomes of interest are not reported at the follow up stage, just at baseline. Furthermore was it difficult to disaggregate subgroups |
| (Meyer, 2005) | Empirical Data – This was a policy paper not presenting results from an empirical study |
| (Mitchell, Brooks, & Glendinning, 2015) | Outcome – The main focus of this study are carers and outcomes for carers rather than the PwD |
| (Morse, 2011) | Method – Users of individualised funding and ‘self-funders’ participate in this study but it was not possible to disaggregate the results. Detail was sparse on sample, disability status etc. |
| (National Council on Disability, 2004) | Intervention – This study was primarily a review of the literature with interviews held with policy makers / researchers to discuss findings. An intervention is not implemented |
| (Needham, 2010) | Intervention – The focus of this paper was personalisation in general. An intervention has not been implemented. |
| (Neville, 2010) | Population – It is not clear whether whole population had a life-long disability or whether the older population had an age-related disability. |
| (Nolan, 2003) | Intervention – This was a pre-implementation study based on hypothetical situation of receiving individualised funding |
| (Norrie, Weinstein, Jones, Hood, & Bhanbro, 2014) | Population – The majority of the sample appear to be frail older people with no evidence of life-long disability |
| (O'Brien, Ford, & Malloy, 2005) | Intervention - The study focuses on employment supports, specifically a ‘career fund’ operated by a financial services organisation. The intervention did not meet the intervention definition |
| (O’Keeffe, 2009) | Outcome – The outcome of interest in this study appears to be organisational change and roll-out of personalisation. It was also difficult to determine what was based in data and on opinion. |
| (O’Keeffe, O’Keeffe, Wiener, & Siebenaler, 2007) | Method – This paper is descriptive in nature with no raw data to draw from. While implementation considerations were described it was too difficult to extract qualitative data for analysis. |
| (Orion Marketing Research, 2007) | Intervention – The intervention in this study was not clearly defined and there was no specific mention of individualised funding |
| (Goetz Ottmann, Laragy, & Haddon, 2009) | Population – The majority of PwD are minors. The study focused on the perspectives of family members and not about implementation |
| (Goetz Ottmann & Mohebbi, 2014) | Population - The sample was older people with complex needs but no specific indication of lifelong disability. |
| (Peak & Waters, 2008) | Study Design – There was no control group for the cross-sectional survey and there are no methods described for the qualitative data |
| (Pearson, 2004) | Intervention - This study does not seem to have an intervention in place but rather a policy analysis based on policy developers perceptions of individualised funding and its aims |
| (B. Phillips et al., 2003) | Study Design – Methodological detail was sparse. Do not know how many were interviewed, who were they representing, how was data analysed. |
| (V. L. Phillips, 1996) | Empirical Data – Not found, however based on the available information this periodical was too short (3 pages) to report effectiveness intervention. Furthermore the focus appears to be on role of case managers. |
| (Pitts, Soave, & Waters, 2009) | Study Design – Uncontrolled cross-sectional survey. Methodological detail was sparse for qualitative element particularly for how data was collected and analysed. |
| (Poll, Duffy, Hatton, Sanderson, & Routledge, 2006) | Study Design – This was a pre/post study with no control group. |
| (Presland, 2013) | Intervention – None of the PwD participating in the study were in receipt of individualised funding |
| (Priestley et al., 2007) | Intervention – An intervention was not implemented in this study. Rather it was examining potential mechanisms underlying uneven outcomes for PwD |
| (J. M. Prince, Manley, & Whiteneck, 1995) | Intervention - Only 27% of individuals in intervention group were funded by public (government funds). Also the length of time in receipt of intervention was unclear |
| (M. J. Prince, 2011) | Empirical Data - This paper was a comparative policy analysis, not empirical data was reported. |
| (Quach, 2010) | Not Found - Requested from author. Focus of the study appears to be on the role of broker rather than outcomes for PwD |
| (Ramakers et al., 2007) | Method – This was a cross-sectional survey design but there was no control group for part 5 of the study, which was the only part with relevant data. |
| (Richards, Waters, & Frisby) | Method – There was no control group for quantitative data. Methodological detail was sparse for the qualitative data. |
| (Richmond, Beatty, Tepper, & DeJong, 1997) | Outcome – Productivity was the outcome of interest in this study, and did not meet the inclusion criteria as outlined in the protocol |
| (Riddell et al., 2006) | Empirical Data – This appears to be a secondary report of data already captured, but the majority of data presented was not empirical |
| (Ridley & Jones, 2002) | Intervention – This was a pre-implementation study. The interviews / focus groups are based primarily on hypothetical situations |
| (Robert Wood Johnson Foundation, 2013) | Empirical Data – This study was based on a review of the ‘Cash & Counseling’ literature – references cross checked. |
| (Robinson, 2012) | Population – The majority of intervention participants are children or those representing children. |
| (Robinson et al., 2010) | Intervention – This paper outlines the plan for an evaluation rather than results. Furthermore, the focus was advanced case management, rather than individualised funding |
| (Romano, 2009) | Empirical Data – This study was based on documentary analysis of existing data but does not seem to collect empirical data |
| (Rowell & Connelly, 2008) | Intervention - The focus of this paper was the development of a scale to measure impact on employment pre and post spinal cord injury |
| (Rutter et al., 2004) | Intervention – The focus of this study was case management and care management, the definitions of which do not align with the inclusion criteria |
| (San Antonio et al., 2010) | Population - Respondents are older population with chronic health conditions rather than life-long disabilities |
| (Schore, Foster, & Phillips, 2007) | Empirical Data – The data presented in this paper has already been captured elsewhere in the systematic review.(Schore & Phillips, 2004) |
| (Schore & Phillips, 2004) | Method – There was no control group for the data presented. |
| (Sciegaj, Crisp, DeLuca, & Mahoney, 2013) | Intervention – This paper was descriptive in nature discussing how managed long-term services and supports programs have implemented participant direction. There was no raw data presented |
| (Scope, 2003) | Population – 52% of the population were minors, 35% <17 |
| (Shen et al., 2008) | Population – This paper focuses on the ‘Cash and Counseling’ older population only. While the current study identifies those that had mental illness and those that did not, there was no way of know if the 'non-mentally ill' had any other disabilities which may confound the findings. |
| (Lori Simon-Rusinowitz, Bochniak, Mahoney, Marks, & Hecht, 2000) | Empirical Data - This was a pre implementation study to inform design of Cash & Counseling study. This study was about experts perceptions of potential barriers to implementation. |
| (Lori Simon-Rusinowitz, Mahoney, Loughlin, & Sadler, 2005) | Outcome - The main focus of this paper are the health and satisfaction outcomes in relation to paid relative vs paid non-relative, not for the PwD |
| (Lori Simon-Rusinowitz, Mahoney, Marks, Zacharias, & Loughlin, 2005) | Intervention – This was a pre-implementation study focusing on consumer preferences |
| (Lori Simon-Rusinowitz et al., 2001) | Intervention - This was a pre-implementation study focusing on potential interest in a consumer directed programme |
| (Lori Simon-Rusinowitz et al., 2002) | Empirical Data – This study was based on secondary data from three separate studies with various publications already screened |
| (Slay, 2011) | Empirical Data – This was a literature review on personalisation, setting out a framework for analysing what benefits co-production might bring for individuals receiving personalised care and support. |
| (Social Interface, 2007) | Intervention – Individualised funding was not implemented in this study. Instead it focuses on the opinions of people who do not receive personal budgets, in effort to understand uptake variation |
| (Solovieva, Wallsh, Hendricks, & Dowler, 2010) | Intervention – The intervention was reasonable accommodations at work. There were 45 different sources of funding. Personal budgets were just one of those. |
| (Spall, McDonald, & Zetlin, 2005) | Intervention - The intervention in question was a ‘quasi-market’ model which does not fit the eligibility criteria for this review |
| (J. Spaulding-Givens, 2011) | Method – There was no control group for the quantitative data. The qualitative outcomes focus on barriers and facilitators to recovery goals rather than the self-directed care aspect |
| (J. C. Spaulding-Givens & Lacasse, 2015) | Method – There was no control group for the data presented |
| (Tim Stainton, Asgarova, & Feduck, 2013) | Method – This was not looking at cost-effectiveness but rather comparing various models of delivery during a snapshot in time. |
| (Tim; Stainton, Boyce, & Phillips, 2009) | Method - This study did not use a matched control group. As acknowledged by the authors “The main study was not designed to provide definitive cost comparisons with conventional services” |
| (Stevens et al., 2011) | Outcome - This paper focused on the ‘concept’ of choice not the actual experience of individual. Other relevant data was captured elsewhere in the review |
| (Sullivan, 2006) | Method – There was no control group for quantitative data. Qualitative findings were reportedly available in an appendix, however this was not found. |
| (Tattrie, Stuart, Hanes, Ford, & Gyarmati, 2003) | Outcome – The main outcome of interest was finding and staying in employment. Health and social care outcomes are not the focus |
| (Jane Tilly, 2007) | Method - It was difficult to extract any qualitative data from this study as no raw data was presented, and no clear results section. Results seem to be interspersed with references to other studies. |
| (J. Tilly & Wiener, 2001) | Method – The quantitative findings are based on secondary data. It very difficult to disaggregate qualitative findings from general discussion on survey findings from other studies |
| (Timonen, Convery, & Cahill, 2006) | Population - The main focus of this study was cash for care services for the elderly not people with disabilities. Although there was some mention of people with dementia/cognitive impairment it refers to a general population of older people with care needs. |
| (Tyson et al., 2010) | Method – There was no control group for quantitative data. While qualitative ‘real life’ stories are presented, methodological detail was sparse |
| (C. Ungerson, 1999) | Intervention – The focus of this paper was the role of the carer generally and their experiences without specific reference to implementation of individualised funding |
| (Clare Ungerson, 2004) | Population – The focus of this study was the carer / worker. Furthermore the majority of the population do not have a defined disability, rather it was older people with care needs |
| (Clare Ungerson, 2006) | Empirical Data – Not found however the reference was for conference proceedings, most likely related to studies screened |
| (Vadapalli, 2009) | Intervention – Conditional cash transfers are not an individualised funding intervention as defined by the current systematic review. While some of the participants of this programme may have a disability, they are not the focus of intervention |
| (Valios, 2000) | Empirical Data – Not found, however it was a very short paper (2 pages) and abstract reads as journalistic rather than academic |
| (Victorian Auditor-General's, 2011) | Study Design – Methodological detail was sparse with no real description of how potentially relevant quotes were obtained. Furthermore the case studies presented are not empirical data and are referenced to another source. |
| (Wadensten & Ahlström, 2009) | Intervention – This study focuses on agency directed personal assistant services, with no reference to individualised funding |
| (Walker, Hewitt, Bogenschutz, & Hall-Lande, 2009) | Population - The majority of states offer the services to adults and children with no description of the split and percentages of each. Could not determine which data was linked to adults only |
| (Waters & Hay, 2009) | Methods – There was no control group for quantitative data. Methodological detail was spare for qualitative data (i.e. no description of the methods, sampling, analysis) |
| (Weech, 2009) | Empirical Data – This was a descriptive paper where the individualised funding was discussed but no intervention or data collected |
| (Weinbach, 2010) | Intervention – This study appears to focus on the impact of counselling on the process rather than effectiveness of individualised funding |
| (Wiesel et al., 2015) | Intervention – The focus of this study was transitioning from parent’s house to own home. 38% of respondents were in receipt of individualised funding but this was not a focus of study |
| (Wilberforce et al., 2011) | Population – Only 44% of participants in this study had a defined disability. The remainder were older people, with no way to disaggregate data. |
| (Val Williams, Ponting, & Ford, 2009) | Outcome - The focus of this paper was the relationship between PA and PwD, not on the health and social care outcomes as result of individualised funding |
| (V. Williams & Porter, 2015) | Intervention – This was a pre-implementation study focussing on processes and practice of conducting support planning. |
| (Zhang, 2015) | Intervention – The focus of this study was personalised services but not individualised funding. |

Abbott, D., & Marriott, A. (2013). Money, finance and the personalisation agenda for people with learning disabilities in the UK: some emerging issues. *British Journal of Learning Disabilities, 41*(2), 106-113 108p. doi:10.1111/j.1468-3156.2012.00728.x

Ageing and Aged Care. (2012). *Evaluation of the consumer - directed care initiative - Final Report*. Retrieved from Australia: <https://www.dss.gov.au/ageing-and-aged-care-publications-and-articles-ageing-and-aged-care-reports/evaluation-of-the-consumer-directed-care-initiative-final-report>

Alakeson, V. (2008a). Let Patients Control the Purse Strings. *BMJ (British Medical Journal), 336*(7648), 807-809. doi:<http://dx.doi.org/10.1136/bmj.39524.400498.AD>

Alakeson, V. (2008b). Self-directed care for adults with serious mental illness: the barriers to progress. *Psychiatric Services, 59*(7), 792-794 793p.

Allen, D. (2008). Cash control. *Learning Disability Practice, 11*(10), 10-10 11p.

Anderson, W. L., Wiener, J. M., & Khatutsky, G. (2006). Workforce issues and consumer satisfaction in medicaid personal assistance services. *Health Care Financing Review, 28*(1), 87-101.

Arksey, H., & Baxter, K. (2011). Exploring the temporal aspects of direct payments. *British Journal of Social Work*, bcr039.

Arntz, M., & Thomsen, S. L. (2011). Crowding Out Informal Care? Evidence from a Field Experiment in Germany: Crowding out informal care. *Oxford Bulletin of Economics and Statistics, 73*(3), 398-427. doi:10.1111/j.1468-0084.2010.00616.x

ARTD Consultants. (2009). *Evaluation of the Self-Managed Model in the Community Participation Program*. Retrieved from Sydney, Australia: <http://www.adhc.nsw.gov.au/__data/assets/file/0010/240886/28_Evaluation_of_SMM_July2009.pdf>

Barczyk, A. N., & Lincove, J. A. (2010). Cash and counseling: a model for self-directed care programs to empower individuals with serious mental illnesses. *Social Work in Mental Health, 8*(3), 209-224 216p. doi:10.1080/15332980903405298

Barton, H. (2012). MAXIMISING INDIVIDUALS' CONTROL OVER THEIR LIVES. *Learning Disability Practice, 15*(3), 12-16 15p.

Baxter, A. (2006). *Towards a good life: One family's experiences with individualized supports.* (MR16734 M.A.Psy.), Wilfrid Laurier University (Canada), Ann Arbor. Retrieved from <http://search.proquest.com/docview/304921540?accountid=12309>

<http://fh6xn3yd3x.search.serialssolutions.com/?ctx_ver=Z39.88-2004&ctx_enc=info:ofi/enc:UTF-8&rfr_id=info:sid/ProQuest+Dissertations+%26+Theses+A%26I&rft_val_fmt=info:ofi/fmt:kev:mtx:dissertation&rft.genre=dissertations+%26+theses&rft.jtitle=&rft.atitle=&rft.au=Baxter%2C+Alison&rft.aulast=Baxter&rft.aufirst=Alison&rft.date=2006-01-01&rft.volume=&rft.issue=&rft.spage=&rft.isbn=9780494167342&rft.btitle=&rft.title=Towards+a+good+life%3A+One+family%27s+experiences+with+individualized+supports&rft.issn=&rft_id=info:doi/>

<http://fh6xn3yd3x.search.serialssolutions.com/?genre=article&sid=ProQ:&atitle=Towards+a+good+life%3A+One+family%27s+experiences+with+individualized+supports&title=Towards+a+good+life%3A+One+family%27s+experiences+with+individualized+supports&issn=&date=2006-01-01&volume=&issue=&spage=&author=Baxter%2C+Alison> ProQuest Dissertations & Theses A&I database.

Beatty, P., Adams, M., & O'Day, B. (1998). Virginia's consumer-directed personal assistance services program: a history and evaluation. *American Rehabilitation, 24*(2/3), 31-35 35p.

Benjamin, A. E., Franke, T. M., Matthias, R., & Park, E. (1999). Consumer Direction and In-Home Services: Recipient Perspectives on Family and Non-Family Service Provision. *Journal of Rehabilitation Administration, 22*(4), 233-247.

Bond, H. (1996). State of independence. *Community Care, 4*, 20-21.

Bradley, V., Agosta, J., Smith, G., Taub, S., Ashbaugh, J., Silver, J., & Heaviland, M. (2001). *The Robert Wood Johnson Foundation Self-Determination Initiative: Final Impact Assessment Report* (32677). Retrieved from Cambridge, MA, USA:

Brandon, D. (1989). Service with a smile... service brokerage... a community-based mental handicap service. *Nursing Times, 85*(45), 66-67 62p.

Breda, J., Gevers, H., Vandervelden, M., & Peeters, S. (2011). *HET EXPERIMENT PERSOONSGEBONDEN BUDGET: Eindverslag van de wetenschappelijke evaluatie*. Retrieved from Antwerp, Belgium:

Breda, J., Schoemaekers, D., Van Landeghem, C., Claessens, D., & Geerts, J. (2006). When informal care becomes a paid job: the case of Personal Assistance Budgets in Flanders. In C. K. Glendinning, Peter. A. (Ed.), *Cash and Care - Policy challenges in welfare state* (pp. 155 - 170). Great Britain: The Policy Press.

Brewis, R. (2009). Self-directed support in health care: can it work? *Community Living, 22*(4), 16-17 12p.

Bright, A., & Drake, M. (1999). *People with learning difficulties and their access to direct payments schemes*. Retrieved from <https://www.jrf.org.uk/report/people-learning-difficulties-and-their-access-direct-payments-schemes>

Broadhurst, S., Yates, K., & Mullen, B. (2012). An evaluation of the My Way transition programme. *Tizard Learning Disability Review, 17*(3), 124-134 111p. doi:10.1108/13595471211240960

Brown, R. I. (1996). Personal programmes and quality of life. *International Journal of Practical Approaches to Disability, 20*(3), 2-8.

Caldwell, J. (2006). Consumer-Driected Supports: Economic, Health, and Social Outcomes for Families. *Mental Retardation*.

Caldwell, J., & Heller, T. (2003). Management of respite and personal assistance services in a consumer‐directed family support programme. *Journal of Intellectual Disability Research, 47*(4‐5), 352-367. doi:10.1046/j.1365-2788.2003.00496.x

Callahan, M. (2000). *Final Report: UCPA's Choice Access Project*. Retrieved from Gautier, Mississippi:

Callahan, M. (2001). Personal budgets: the future of funding? *Journal of Vocational Rehabilitation, 16*(1), 15-26 12p.

Campbell, E. M., Fortune, J., & Heinlein, K. B. (1998). The effects of funding packages on the outcomes of integration and independence of adults with developmental disabilities in two rural states. *Journal of Developmental and Physical Disabilities, 10*(3), 257-281. doi:10.1023/A:1022868007755

Campbell, M. (2010). Personalisation–Stakeholder perceptions and the impact on social care commissioning in Liverpool.

Carers UK. (2008). *Choice or Chore: Carers’ experiences of direct payments* Retrieved from London, UK:

Carr, S. (2010). Enabling risk and ensuring safety: self-directed support and personal budgets. *Journal of Adult Protection, 13*(3), 122-136 115p. doi:10.1108/14668201111160723

Chopin, N. S., & Findlay, I. M. (2010). *Exploring Key Informants‟ Experiences with Self-Directed Funding*. Retrieved from

Christensen, K. (2006). A Stark Choice. *Community Care*(1630), 32-33.

Clark, M. J., Hagglund, K. J., & Sherman, A. K. (2008). A longitudinal comparison of consumer-directed and agency-directed personal assistance service programmes among persons with physical disabilities. *Disability & Rehabilitation, 30*(9), 689-695 687p.

Cloutier, H., Malloy, J., Hagner, D., & Cotton, P. (2006). Choice and control over resources: New Hampshire's Individual Career Account demonstration projects. *Journal of Rehabilitation, 72*(2), 4-11.

Cocks, E., & Boaden, R. (2009). *The Personalised Residential Supports Project*. Retrieved from Perth, Western Australia:

Conroy, J. W., & Yuskauskas, A. (1996). *Independent Evaluation of the Monadnock Self Determination Project*. Retrieved from Narberth, PA, USA

Cook, J. A., Russell, C., Grey, D. D., & Jonikas, J. A. (2008). A self-directed care model for mental health recovery. *Psychiatric Services, 59*(6), 600-602.

Cook, J. A., Shore, S. E., Burke-Miller, J. K., Jonikas, J. A., Ferrara, M., Colegrove, S., . . . Hicks, M. E. (2010). Participatory Action Research to Establish Self-Directed Care for Mental Health Recovery in Texas. *Psychiatric Rehabilitation Journal, 34*(2), 137-144 138p. doi:10.2975/34.2.2010.137.144

Cornford, H. (2011). Simplifying the process of personal budgets. *Community Care*(1860), 32-33 32p.

Cortis, N., Meagher, G., Chan, S., Davidson, B., & Fattore, T. (2013). Building an industry of choice: service quality, workforce capacity and consumer-centred funding in disability care. *Final report prepared for United Voice, Australian Services Union and Health and Community Services Union, Social Policy Research Centre, University of New South Wales, Sydney*.

Cunningham, I., & Nickson, D. (2010). Personalisation and its Implications for Work and Employment in the Voluntary Sector.

Dale, S. B., & Brown, R. (2006). Reducing Nursing Home Use Through Consumer-Directed Personal Care Services. *Medical Care, 44*(8), 760-767. doi:10.1097/01.mlr.0000218849.32512.3f

Davey, V., Fernández, J.-L., Knapp, M., Vick, N., Jolly, D., Swift, P., . . . Priestley, M. (2007). *Direct Payments: A National Survey of Direct Payments Policy and Practice*. Retrieved from London:

Davidson, J., Baxter, K., Glendinning, C., & Irvine, A. (2013). Choosing health: qualitative evidence from the experiences of personal health budget holders. *Journal of Health Services Research & Policy, 18*(s2), 50-58 59p. doi:10.1177/1355819613499747

Davidson, J., Baxter, K., Glendinning, C., Jones, K., Forder, J., Caiels, J., . . . King, D. (2012). Personal Health Budgets: Experiences and outcomes for budget holders at nine months. *York: Social Policy Research Unit, University of York*.

Davies, J., & Morgan, H. (2010). What kind of a future for young people with Down's Syndrome? The views and aspirations of young people and families. *Tizard Learning Disability Review, 15*(4), 22-30 29p. doi:10.5042/tldr.2010.0590

Dean, E. E., Dunn, W., & Tomchek, S. (2015). Role of occupational therapy in promoting self-determination through consumer-directed supports. *Occupational therapy in health care, 29*(1), 86-95. doi:<http://dx.doi.org/10.3109/07380577.2014.958887>

Dew, A., Veitch, C., Bulkeley, K., Bundy, A., Lincoln, M., Gallego, G., . . . Griffiths, S. (2012). Impact of individual funding on therapy service access for people with a disability in rural areas. *Journal of Intellectual Disability Research, 56*(7-8), 769.

Doty, P., Kasper, J., & Litvak, S. (1996). Consumer-directed models of personal care: lessons from Medicaid. *The Milbank quarterly, 74*(3), 377-409.

Dougherty, R. H., & Eggers, W. D. (1996). Delivering Services for the Mentally Ill and Developmentally Disabled.

Doyle, Y. (1995). Disability: use of an independent living fund in south east London and users' views about the system of cash versus care provision. *Journal of Epidemiology & Community Health, 49*(1), 43-47 45p. doi:10.1136/jech.49.1.43

Duffy, S. (2007). The economics of self-directed support. *Journal of Integrated Care, 15*(2), 26-37 12p.

Edwards, T., & Waters, J. *It's your life - take control: The implementation of self-directed support in Hertfordshire*. Retrieved from Hertford, UK:

Eriksson, S. (2012). Implications of personal budgeting in services for people with disabilities-Making of full citizenship? *Journal of Intellectual Disability Research, 56*(7-8), 726.

Eriksson, S. (2014a). The Need for Self-Determination and Imagination: Personal Budgeting and the Management of Disability Services in Finland. *Journal of Policy & Practice in Intellectual Disabilities, 11*(2), 137-148 112p. doi:10.1111/jppi.12079

Eriksson, S. (2014b). *Personal Budgeting in Municipal Disability Services - The First Experiment in Finland*. Retrieved from Helsinki, Finland: <http://www.kehitysvammaliitto.fi/wp-content/uploads/personal_budgeting_in_municipal_disability_services.pdf>

Evans, C., & Carmichael, A. (2002). *Users’ Best Value: A guide to user involvement good practice in Best Value Reviews;* . Retrieved from York, UK:

Field, A., McGechie, M., & King, J. (2015). *MIC Individualised Funding Analysis - Report for Manawanui InCharge*. Retrieved from Auckland, New Zealand:

Fisher, J., Rayner, M., & Baines, S. (2011). *Personalisation of Social Care and Health: A Co-Operative Solution*. Retrieved from Manchester, UK:

Fisher, K. R., & Purcal, C. (2016). Effective Personalised Housing Support for People with Disabilities - Case Study Analysis. *The Australian Journal of Social Issues, 45*(4), 527-542.

Flynn, M. (2005). *NEW TYPES OF WORKER PROJECT - Developing the role of personal assistants*. Retrieved from Leeds:

Forbes, D. A., Markle-Reid, M., Hawranik, P., Peacock, S., Kingston, D., Morgan, D., . . . Leipert, B. J., S. L. (2008). Availability and acceptability of Canadian home and community-based services: perspectives of family caregivers of persons with dementia. *Home Health Care Services Quarterly, 27*(2), 75-99 25p.

Forder, J., Jones, K., Glendinning, C., Caiels, J., Welch, E., Baxter, K., . . . King, D. (2012). Evaluation of the personal health budget pilot programme.

Foster, L., Brown, R., Carlson, B., Phillips, B., & Schore, J. (2000). *CASH AND COUNSELING: CONSUMER’S EARLY EXPERIENCES IN ARKANSAS*. Retrieved from Washinton DC:

Foster, L., Brown, R., Carlson, B., Phillips, B., & Schore, J. (2002). *Cash and Counseling: Consumers’ Early Experiences in New Jersey Part II: Uses of Cash and Satisfaction at Nine Months*. Retrieved from <http://www.mathematica-mpr.com/~/media/publications/PDFs/conearlyexp.pdf>

Foster, M., Harris, J., Jackson, K., Morgan, H., & Glendinning, C. (2006). Personalised social care for adults with disabilities: a problematic concept for frontline practice. *Health & Social Care in the Community, 14*(2), 125-135. doi:10.1111/j.1365-2524.2006.00602.x

Fox, M. H., & Kim, K. M. (2004). Evaluating a Medicaid home and community-based physical disability waiver. *Family & Community Health, 27*(1), 37-51 15p.

Friedman, B., Wamsley, B. R., & Conwell, Y. (2015). Do Disabled Elderly Medicare Beneficiaries with Major Depression Make Less Use of a Consumer-Directed Home Care Voucher Benefit? *Journal of Aging & Social Policy, 27*(1), 87-105 119p. doi:10.1080/08959420.2015.969148

Fyson, R. (2012). Independent and vulnerable? Tensions and contradictions in UK policy and practice associated with intellectual disability. *Journal of Intellectual Disability Research, 56*(7-8), 727.

G. Allan Roeher Institute. (1991). *The power to choose: An examination of service brokerage and individualized funding as implemented by the community living society.* . Ontario, Canada The G. Allan Roeher Institute, .

Gibson, B. E., Brooks, D., DeMatteo, D., & King, A. (2009). Consumer-directed personal assistance and 'care': perspectives of workers and ventilator users. *Disability & Society, 24*(3), 317-330 314p.

Gill, L., & Cameron, I. D. (2015). Innovation and Consumer Directed Care: Identifying the challenges. *Australasian Journal on Ageing, 34*(4), 265-268 264p. doi:10.1111/ajag.12222

Gill, L., McCaffrey, N., Cameron, I. D., & Ratcliffe, J. (2016). Consumer Directed Care in Australia: Early perceptions and experiences of staff, clients and carers. *Health & Social Care in the Community*, n-a-n/a. doi:10.1111/hsc.12328

Glasby, J. (2008). Individual Budgets and the interface with health: a discussion paper for the Care Services Improvement Partnership (CSIP). *Health Services Management Centre, University of Birmingham*.

Graham, K. (2015). Cash payments in context: (self-)regulation in the new social relations of assistance. *Disability & Society, 30*(4), 597-613 517p. doi:10.1080/09687599.2015.1037951

Gridley, K., Brooks, J., & Glendinning, C. (2014). Good practice in social care: the views of people with severe and complex needs and those who support them. *Health & Social Care in the Community, 22*(6), 588-597 510p. doi:10.1111/hsc.12105

Grootegoed, E., Knijn, T., & da Roit, B. (2010). Relatives as paid care-givers: how family carers experience payments for care. *Ageing & Society, 30*(3), 467-489. doi:<http://dx.doi.org/10.1017/S0144686X09990456>

Grossman, B. R., Kitchener, M., Mullan, J. T., & Harrington, C. (2007). Paid personal assistance services: an exploratory study of working-age consumers' perspectives. *Journal of Aging & Social Policy, 19*(3), 27-45 19p.

Hagglund, K. J., Clark, M. J., Farmer, J. E., & Sherman, A. K. (2004). A comparison of consumer-directed and agency-directed personal assistance services programmes. *Disability & Rehabilitation, 26*(9), 518-527 510p.

Hall-Lande, J., Hewitt, A., Bogenschutz, M., & LaLiberte, T. (2012). County Administrator Perspectives on the Implementation of Self-Directed Supports. *Journal of Disability Policy Studies, 22*(4), 247-256 210p. doi:10.1177/1044207311416595

Hall, E. (2009). Being in control: personal budgets and the new landscape of care for people with learning disabilities. *Mental Health Review Journal, 14*(2), 44-53 10p.

Hammond, J. (2012). Signpost - a model of self-directed support and a framework for brokerage. *Mental Health & Social Inclusion, 16*(1), 48-55 48p. doi:10.1108/20428301211205900

Harding-Price, D. (2011). Benefits of giving service users personalised budgets. *Mental Health Practice, 14*(5), 20-22 23p.

Hashemi, L., Henry, A. D., Ellison, M. L., Banks, S. M., Glazier, R. E., & Himmelstein, J. (2008). The relationship of personal assistance service utilization to other Medicaid payments among working-age adults with disabilities. *Home Health Care Services Quarterly, 27*(4), 280-298 219p.

Hasler, F., & Stewart, A. (2004). *Developing direct payments in the North East of England*. Retrieved from

Hatton, C., Waters, J., Duffy, S., Senker, J., Crosby, N., Poll, C., . . . Towell, D. (2008). *A report on In Controls's Second Phase: Evaluation and learning 2005-2006*. Retrieved from London:

Hay, M., & Waters, J. (2009). Steering my own course. *Cambridge, Cambridgeshire County Council*.

Head, M. J., & Conroy, J. W. (2005). Outcomes of Self-Determination in Michigan: Quality and Costs *Costs and outcomes of community services for people with intellectual disabilities.* (pp. 219-240). Baltimore, MD, US: Paul H Brookes Publishing.

Heller, T., Arnold, C. K., van Heumen, L., McBride, E. L., & Factor, A. (2012). Self-directed Support: Impact of Hiring Practices on Adults with Intellectual and Developmental Disabilities and Families. *American Journal on Intellectual & Developmental Disabilities, 117*(6), 464-477 414p. doi:10.1352/1944-7558-117.6.464

Heller, T., & Caldwell, J. (2005). Impact of a Consumer‐Directed Family Support Program on Reduced Out‐of‐Home Institutional Placement. *Journal of Policy and Practice in Intellectual Disabilities, 2*(1), 63-65. doi:10.1111/j.1741-1130.2005.00010.x

Henwood, M. (2008). Self-directed support: grounds for optimism. *Community Care*(1722), 34-35 32p.

Henwood, M., & Hudson, B. (2007). The Independent Living Funds -- what does the future hold? *Journal of Integrated Care, 15*(4), 36-42 37p.

Hitchen, S. (2013). Personal Budgets for all? An Action Research Study on Implementing Self-Directed Support in Mental Health Services.

Hitchen, S., Williamson, G. R., & Watkins, M. (2015). Personal budgets for all? Implementing self-directed support in mental health services. *Action Research, 13*(4), 372-391. doi:10.1177/1476750314568207

InControl. *Your Way. The story so far of Self Directed Support in the London Borough of Richmond upon Thames*. Retrieved from

Inoue, M., Lombe, M., Putnam, M., & Mahoney, K. J. (2014). Understanding Saving and Purchase Patterns of Consumers in a Self-Directed Care Program: The West Virginia Experience. *Journal of Policy Practice, 13*(2), 101-117 117p. doi:10.1080/15588742.2014.881272

Irvine, A., Davidson, J., Glendinning, C., Jones, K., Forder, J., Caiels, J., . . . King, D. (2011). *Personal Health Budgets: Early experiences of budget holders - Fourth Interim Report*. Retrieved from London, UK:

Isaacson, N. C., Cocks, E., & Netto, J. A. (2014). Launching: The experiences of two young adults with intellectual disability and their families in transition to individual supported living. *Journal of Intellectual & Developmental Disability, 39*(3), 270-281 212p. doi:10.3109/13668250.2014.929643

James, A. N. (2008). A critical consideration of the cash for care agenda and its implications for social services in Wales. *Journal of Adult Protection, 10*(3), 23-34 12p.

Jensen, M. (2008). *Creating individualized services for people with developmental disabilities.* (MR36590 M.A.), Royal Roads University (Canada), Ann Arbor. Retrieved from <http://search.proquest.com/docview/304812369?accountid=12309>

<http://fh6xn3yd3x.search.serialssolutions.com/?ctx_ver=Z39.88-2004&ctx_enc=info:ofi/enc:UTF-8&rfr_id=info:sid/ProQuest+Dissertations+%26+Theses+A%26I&rft_val_fmt=info:ofi/fmt:kev:mtx:dissertation&rft.genre=dissertations+%26+theses&rft.jtitle=&rft.atitle=&rft.au=Jensen%2C+Mike&rft.aulast=Jensen&rft.aufirst=Mike&rft.date=2008-01-01&rft.volume=&rft.issue=&rft.spage=&rft.isbn=9780494365908&rft.btitle=&rft.title=Creating+individualized+services+for+people+with+developmental+disabilities&rft.issn=&rft_id=info:doi/>

<http://fh6xn3yd3x.search.serialssolutions.com/?genre=article&sid=ProQ:&atitle=Creating+individualized+services+for+people+with+developmental+disabilities&title=Creating+individualized+services+for+people+with+developmental+disabilities&issn=&date=2008-01-01&volume=&issue=&spage=&author=Jensen%2C+Mike> ProQuest Dissertations & Theses A&I database.

Jones, K., Caiels, J., Forder, J., Windle, K., Welch, E., Dolan, P., . . . King, D. (2010). *Early experiences of implementing personal health budgets*. Retrieved from <http://www.pssru.ac.uk/pdf/dp2726-2.pdf>

Jones, K., Forder, J., Caiels, J., Welch, E., Glendinning, C., & Windle, K. (2013). Personalization in the health care system: do personal health budgets have an impact on outcomes and cost? *Journal of Health Services Research & Policy, 18*(s2), 59-67 59p. doi:10.1177/1355819613503152

Jones, K., Forder, J., Caiels, J., Welch, E., Windle, K., Davidson, J., . . . King, D. (2011). *The cost of implementing personal health budgets*. Retrieved from UK:

Jones, K., & Netten, A. (2010). The costs of change: a case study of the process of implementing individual budgets across pilot local authorities in England. *Health & Social Care in the Community, 18*(1), 51-58 58p. doi:10.1111/j.1365-2524.2009.00873.x

Judd, S. (2014). *The NDIS one year in: experiences of carers in the Hunter trial site*. Retrieved from <http://apo.org.au/resource/ndis-one-year-experiences-carers-hunter-trial-site>

Kaiser Family Foundation. (2006). National survey of enrollees in consumer directed health plans : chartpack. Retrieved from <https://kaiserfamilyfoundation.files.wordpress.com/2013/01/7596.pdf>

Kassner, E., Coleman, B., & Milne, D. (2008). *A balancing act: State long-term care reform*: AARP, Public Policy Institute.

Keigher, S. M. (2000). The interests of three stakeholders in independent personal care for disabled elders. *Journal of Health and Human Services Administration, 23*(2), 136-160.

Kelly, C. (2014). *Re/Moving care: Making care accessible through the Ontario Direct Funding program.* (AAINR87741). Retrieved from <http://search.proquest.com/docview/1520323557?accountid=12309>

<http://fh6xn3yd3x.search.serialssolutions.com/?ctx_ver=Z39.88-2004&ctx_enc=info:ofi/enc:UTF-8&rfr_id=info:sid/Sociological+Abstracts&rft_val_fmt=info:ofi/fmt:kev:mtx:dissertation&rft.genre=dissertations+%26+theses&rft.jtitle=&rft.atitle=&rft.au=Kelly%2C+Christine&rft.aulast=Kelly&rft.aufirst=Christine&rft.date=2013-01-01&rft.volume=&rft.issue=&rft.spage=&rft.isbn=9780494877418&rft.btitle=&rft.title=Re%2FMoving+care%3A+Making+care+accessible+through+the+Ontario+Direct+Funding+program&rft.issn=&rft_id=info:doi/>

<http://fh6xn3yd3x.search.serialssolutions.com/?genre=article&sid=ProQ:&atitle=Re%2FMoving+care%3A+Making+care+accessible+through+the+Ontario+Direct+Funding+program&title=Re%2FMoving+care%3A+Making+care+accessible+through+the+Ontario+Direct+Funding+program&issn=&date=2013-01-01&volume=&issue=&spage=&author=Kelly%2C+Christine> Sociological Abstracts database.

Kendall, S., & Cameron, A. (2014). Personalisation of adult social care: self-directed support and the choice and control agenda. *British Journal of Learning Disabilities, 42*(4), 264-271 268p. doi:10.1111/bld.12069

Kestenbaum, A. (1992). Cash for care A report on the experience of Independent Living Fund clients: Independent Living Fund, Nottingham (United Kingdom) ;.

Kim, K. M., White, G. W., & Fox, M. H. (2006). Comparing outcomes of persons choosing consumer-directed or agency-directed personal assistance services. *Journal of Rehabilitation, 72*(2), 32-43 12p.

Laragy, C. (2010). Snapshot of flexible funding outcomes in four countries. *Health & Social Care in the Community, 18*(2), 129-138. doi:10.1111/j.1365-2524.2009.00880.x

Laragy, C., David, C., & Moran, N. (2015). A framework for providing information in individualised funding programmes. *Qualitative Social Work*, 1473325015589402.

Larkin, M. (2015). Developing the knowledge base about carers and personalisation: contributions made by an exploration of carers' perspectives on personal budgets and the carer-service user relationship. *Health & Social Care in the Community, 23*(1), 33-41 39p. doi:10.1111/hsc.12131

Larsen, J., Ainsworth, E., Harrop, C., Patterson, S., Hamilton, S., Szymczynska, P., . . . Pinfold, V. (2013). Implementing personalisation for people with mental health problems: A comparative case study of four local authorities in England. *Journal of Mental Health, 22*(2), 174-182 179p. doi:10.3109/09638237.2012.734658

Larson, T. (2006). *Choosing Independence: An Overview of the Cash & Counseling Model of Self-Directed Personal Assistance Services*. Retrieved from USA:

Lavelle, L. (2009). Transforming adult social care: Personalisation and brokerage.

Lawson, S., Pearman, G., & Waters, J. (2010). *Finding Our Way: The story of Self-Directed Support in Barnsley*. Retrieved from Barnsley, UK:

Leece, D., & Leece, J. (2006). Direct Payments: Creating a Two-Tiered System in Social Care? *The British Journal of Social Work, 36*(8), 1379-1393. doi:<http://dx.doi.org/10.1093/bjsw/bch394>

Leece, J. (2010). Paying the piper and calling the tune: power and the direct payment relationship. *British Journal of Social Work, 40*(1), 188-206 119p. doi:bjsw/bcn097

Leece, J. P., Sheila. (2010). Developing New Understandings of Independence and Autonomy in the Personalised Relationship. *The British Journal of Social Work, 40*(6), 1847-1865. doi:<http://dx.doi.org/10.1093/bjsw/bcp105>

Lombe, M. (2016). Understanding Effects of Flexible Spending Accounts on People with Disabilities: The Case of a Consumer-Directed Care Program. *Journal of Social Work in Disability & Rehabilitation, 15*(1), 62-75 14p. doi:10.1080/1536710X.2016.1124255

Lombe, M., Putnam, M., & Huang, J. (2008). Exploring effects of institutional characteristics on saving outcome: the case of the cash and counseling program. *Journal of Policy Practice, 7*(4), 260-279 220p.

Lord, J., & Hutchison, P. (2003). Individualised support and funding: Building blocks for capacity building and inclusion. *Disability and Society, 18*(1), 71-86.

Low, L.-F., Chilko, N., Gresham, M., Barter, S., & Brodaty, H. (2012). An update on the pilot trial of consumer-directed care for older persons in Australia. *Australasian Journal on Ageing, 31*(1), 47-51 45p. doi:10.1111/j.1741-6612.2011.00572.x

Maglajlic, R. (1999). The silent treatment. *OpenMind*(99), 12-13.

Mahoney, K. J., Desmond, S. M., Simon-Rusinowitz, L. L., D. M.; , & Squillace, M. R. (2002). Consumer preferences for a cash option versus traditional services: telephone survey results from New Jersey elders and adults. *Journal of Disability Policy Studies, 13*(2), 74-86 13p.

Mansell, J. (2010). Raising our sights: services for adults with profound intellectual and multiple disabilities. *Tizard Learning Disability Review, 15*(3), 5-12 18p. doi:10.5042/tldr.2010.0399

Manthorpe, J., & Stevens, M. (2008). *The personalisation of adult social care in rural areas*. Retrieved from Cheltenham UK:

Manthorpe, J., Stevens, M., Rapaport, J., Jacobs, S., Challis, D., Wilberforce, M., . . . Glendinning, C. (2010). Gearing up for personalisation: training activities commissioned in the English pilot individual budgets sites 2006–2008. *Social Work Education, 29*(3), 319-331.

Matthias, R. E. B., A. E. (2003). Abuse and neglect of clients in agency-based and consumer-directed home care. *Health & Social Work, 28*(3), 174-184 111p.

McConkey, R., Bunting, B., Ferry, F., Garcia-Iriarte, E., & Stevens, R. (2013). *Personalised Supports to Individuals with Disabilities and Mental Health Difficulties*. Retrieved from <http://www.genio.ie/files/Evaluation_Personalised_Supports_UU2013_0.pdf>

McWilliam, C. L., Stewart, M., Vingilis, E., Hoch, J. S., Ward-Griffin, C., Donner, A., . . . Anderson, K. (2004). Flexible client-driven in-home case management: an option to consider. *Care Manag J, 5*(2), 73-86.

Meng, H., Friedman, B., Wamsley, B. R., Van Nostrand, J. F., & Eggert, G. M. (2010). Choice of personal assistance services providers by medicare beneficiaries using a consumer-directed benefit: Rural-urban differences. *Journal of Rural Health, 26*(4), 392-401.

Meyer, D. (2005). Personal budget for persons in need of care. A socio-economic change with new perspectives for supply and demand. [Das personengebundene Budget bei Pflegebedürftigkeit. Systemwechsel eröffnet Chancen für Anbieter und Nachfrager]. *Pflege Zeitschrift, 58*(11), suppl 2-8.

Mitchell, W., Brooks, J., & Glendinning, C. (2015). Carers' Roles in Personal Budgets: Tensions and Dilemmas in Front Line Practice. *British Journal of Social Work, 45*(5), 1433-1450 1418p. doi:bjsw/bcu018

Morse, A. (2011). *Oversight of user choice and provider competition in care markets*. Retrieved from London:

National Council on Disability. (2004). *Consumer-Directed Health Care: How Well Does It Work?* Retrieved from Washington, DC:

Needham, C. T., J. (2010). Commissioning for Personalisation: From the fringes to the mainstream. *London: Public Management and Policy Association*.

Neville, S. (2010). *Delivering personal budgets for adult social care: Reflections from Essex*. Retrieved from London:

Nolan, A. R., Colm. (2003). Direct Payment Schemes for People with Disabilities: A New and Innovative Policy Approach to Providing Services to Disabled People in Ireland, 42. Retrieved from <http://digitalcommons.ilr.cornell.edu/cgi/viewcontent.cgi?article=1321&context=gladnetcollect>

Norrie, C., Weinstein, J., Jones, R., Hood, R., & Bhanbro, S. (2014). Early experiences in extending personal budgets in one local authority. *Working with Older People: Community Care Policy & Practice, 18*(4), 176-185 110p. doi:10.1108/WWOP-07-2014-0019

O'Brien, D., Ford, L., & Malloy, J. M. (2005). Person centered funding: using vouchers and personal budgets to support recovery and employment for people with psychiatric disabilities. *Journal of Vocational Rehabilitation, 23*(2), 71-79 79p.

O’Keeffe, J. (2009). *Implementing Self-Direction Programs with Flexible Individual Budgets: Lessons Learned From the Cash and Counseling Replication States*. Retrieved from Macon, NC, USA:

O’Keeffe, J., O’Keeffe, C., Wiener, J. M., & Siebenaler, K. (2007). *Increasing Options for Self-Directed Services Initiatives of the FY 2003 Independence Plus Grantees*. Retrieved from Baltimore, MD: <http://www.nasuad.org/sites/nasuad/files/hcbs/files/130/6482/IPpaper.pdf>

Orion Marketing Research. (2007). *Evaluation of Disability Support Program Pilot Project - Final Report*. Retrieved from New Brunswick, Canada:

Ottmann, G., Laragy, C., & Haddon, M. (2009). Experiences of disability consumer-directed care users in Australia: results from a longitudinal qualitative study. *Health & Social Care in the Community, 17*(5), 466-475 410p. doi:10.1111/j.1365-2524.2009.00851.x

Ottmann, G., & Mohebbi, M. (2014). Self-directed community services for older Australians: a stepped capacity-building approach. *Health & Social Care in the Community, 22*(6), 598-611 514p. doi:10.1111/hsc.12111

Peak, M., & Waters, J. (2008). *My Budget, My Choice: Implementing Self-Directed Support in the City of London*. Retrieved from London:

Pearson, C. (2004). Keeping the Cash under Control: What's the Problem with Direct Payments in Scotland? *Disability & Society, 19*(1), 3-14. doi:<http://dx.doi.org/10.1080/0968759032000155596>

Phillips, B., Mahoney, K., Simon-Rusinowitz, L., Schore, J., Barrett, S., Ditto, W., . . . Doty, P. (2003). Lessons from the Implementation of Cash and Counseling in Arkansas, Florida, and New Jersey Retrieved from <http://www.mathematica-mpr.com/~/media/publications/PDFs/3state.pdf>

Phillips, V. L. (1996). The role of case managers in a United Kingdom experiment with self-directed care. *Journal of Case Management, 5*(4), 142-145 144p.

Pitts, J., Soave, V., & Waters, J. (2009). Doing it your way. *Worcester, Worcestershire County Council*.

Poll, C., Duffy, S., Hatton, C., Sanderson, H., & Routledge, M. (2006). *A report on In Control's first phase 2003-2005*. Retrieved from LondonI:

Presland, J. R. (2013). The reality of rights, independence, choice and inclusion for adults with learning disabilities.

Priestley, M., Jolly, D., Pearson, C., Ridell, S., Barnes, C., & Mercer, G. (2007). Direct Payments and Disabled People in the UK: Supply, Demand and Devolution. *The British Journal of Social Work, 37*(7), 1189-1204. doi:<http://dx.doi.org/10.1093/bjsw/bcl063>

Prince, J. M., Manley, M. S., & Whiteneck, G. G. (1995). Self-managed versus agency-provided personal assistance care for individuals with high level tetraplegia. *Archives of Physical Medicine & Rehabilitation, 76*(10), 919-923 915p.

Prince, M. J. (2011). Integrated and individualized service provision for people with disabilities: Promising practices in liberal welfare states. *Journal of Comparative Policy Analysis: Research and Practice, 13*(5), 545-560.

Quach, E. D. O. C., D.; McGaffigan, E. (2010). Supporting people with disabilities in managing individual budgets: the role of support brokers. *Professional Case Management, 15*(1), 29-37 29p. doi:10.1097/NCM.0b013e3181b5ecc4

Ramakers, C., de Graauw, K., Sombekke, E., Vierke, H., Doesborgh, J., & Woldringh, C. (2007). *Evaluatie persoonsgebonden budget nieuwe stijl 2005-2006*. Retrieved from

Richards, S., Waters, J., & Frisby, B. *This time it’s personal: Making self-directed support a reality for people with learning disabilities in Northamptonshire*. Retrieved from Northamptonshire, UK:

Richmond, G. W., Beatty, P., Tepper, S., & DeJong, G. (1997). The effect of consumer-directed personal assistance services on the productivity outcomes of people with disabilities. *Journal of Rehabilitation Outcomes Measurement, 1*(4), 48-51 44p.

Riddell, S., Priestley, M., Pearson, C., Mercer, G., Barnes, C., Jolly, D., & Williams, V. (2006). Disabled people and direct payments: a UK comparative study. *ESRC Award RES‐000‐23, 263*.

Ridley, J., & Jones, L. (2002). *'Direct what?' - A study of direct payments to mental health service users*. Retrieved from Edinburgh, Scotland:

Robert Wood Johnson Foundation. (2013). RWJF Program Results Report—Cash & Counseling. Retrieved from <http://www.rwjf.org/content/dam/farm/reports/program_results_reports/2015/rwjf406468>

Robinson, S. (2012). Evaluation of the self directed support pilot second report for children and young adults with a physical disability.

Robinson, S., Gendera, S., Fisher, K. R., Clements, N., Eastman, C., & Oprea, I. (2010). Evaluation of the Self Directed Support Pilot for Children and Young Adults with a Physical Disability.

Romano, M. J. (2009). *Public long term care models: Possibilities for replication to maximize consumer satisfaction and cost effectiveness among at-risk elders and special populations.* (AAI1478374). Retrieved from <http://search.proquest.com/docview/758131888?accountid=12309>

<http://fh6xn3yd3x.search.serialssolutions.com/?ctx_ver=Z39.88-2004&ctx_enc=info:ofi/enc:UTF-8&rfr_id=info:sid/Sociological+Abstracts&rft_val_fmt=info:ofi/fmt:kev:mtx:dissertation&rft.genre=dissertations+%26+theses&rft.jtitle=&rft.atitle=&rft.au=Romano%2C+Michael+J&rft.aulast=Romano&rft.aufirst=Michael&rft.date=2010-01-01&rft.volume=&rft.issue=&rft.spage=&rft.isbn=9781109565942&rft.btitle=&rft.title=Public+long+term+care+models%3A+Possibilities+for+replication+to+maximize+consumer+satisfaction+and+cost+effectiveness+among+at-risk+elders+and+special+populations&rft.issn=&rft_id=info:doi/>

<http://fh6xn3yd3x.search.serialssolutions.com/?genre=article&sid=ProQ:&atitle=Public+long+term+care+models%3A+Possibilities+for+replication+to+maximize+consumer+satisfaction+and+cost+effectiveness+among+at-risk+elders+and+special+populations&title=Public+long+term+care+models%3A+Possibilities+for+replication+to+maximize+consumer+satisfaction+and+cost+effectiveness+among+at-risk+elders+and+special+populations&issn=&date=2010-01-01&volume=&issue=&spage=&author=Romano%2C+Michael+J> Sociological Abstracts database.

Rowell, D., & Connelly, L. B. (2008). Personal assistance, income and employment: the spinal injuries survey instrument (SISI) and its application in a sample of people with quadriplegia. *Spinal Cord, 46*(6), 417-424 418p.

Rutter, D., Tyrer, P., Emmanuel, J., Weaver, T., Byford, S., Hallam, A., . . . Ferguson, B. (2004). Internal vs. external care management in severe mental illness: randomized controlled trial and qualitative study. *Journal of Mental Health, 13*(5), 453-466 414p.

San Antonio, P., Simon-Rusinowitz, L., Loughlin, D., Eckert, J. K., Mahoney, K. J., & Depretis Ruben, K. A. (2010). Lessons From the Arkansas Cash and Counseling Program: How the Experiences of Diverse Older Consumers and Their Caregivers Address Family Policy Concerns. *Journal of Aging & Social Policy, 22*(1), 1-17. doi:<http://dx.doi.org/10.1080/08959420903385544>

Schore, J., Foster, L., & Phillips, B. (2007). Consumer Enrollment and Experiences in the Cash and Counseling Program. *Health Services Research, 42*(1,part2), 446-466. doi:10.1111/j.1475-6773.2006.00679.x

Schore, J., & Phillips, B. (2004). *Consumer and Counselor Experiences in the Arkansas Independent Choices Program*. Retrieved from Princeton, NJ:

Sciegaj, M., Crisp, S., DeLuca, C., & Mahoney, K. J. (2013). *Participant-Directed Services in Managed Long-Term Services and Supports Programs: A Five State Comparison*. Retrieved from Washinton, USA:

Scope. (2003). *The Direct Approach: Disabled people’s experience of direct payments*. Retrieved from

Shen, C., Smyer, M. A., Mahoney, K. J., Loughlin, D. M., Simon-Rusinowitz, L., & Mahoney, E. K. (2008). Does mental illness affect consumer direction of community-based care? Lessons from the Arkansas Cash and Counseling program. *Gerontologist, 48*(1), 93-104 112p.

Simon-Rusinowitz, L., Bochniak, A. M., Mahoney, K. J., Marks, L. N., & Hecht, D. (2000). Implementation issues for consumer-directed programs: A survey of policy experts. *Generations, 24*(3), 34-40.

Simon-Rusinowitz, L., Mahoney, K. J., Loughlin, D. M., & Sadler, M. D. (2005). Paying family caregivers: An effective policy option in the Arkansas Cash and Counseling Demonstration and Evaluation. *Marriage and Family Review, 37*(1-2), 83-105. doi:10.1300/J002v37n01_07

Simon-Rusinowitz, L., Mahoney, K. J., Marks, L. N., Zacharias, B. L., & Loughlin, D. M. (2005). The Cash and Counseling Demonstration and Evaluation: focus groups inform design of a consumer-directed cash option. *Care Management Journals, 6*(2), 56-65 10p.

Simon-Rusinowitz, L., Mahoney, K. J., Shoop, D. M., Desmond, S. M., Squillace, M. R., & Sowers, J. A. (2001). Consumer and surrogate preferences for a cash option versus traditional services: Florida adults with developmental disabilities. *Mental Retardation, 39*(2), 87-103. doi:10.1352/0047-6765(2001)039<0087:CASPFA>2.0.CO;2

Simon-Rusinowitz, L., Marks, L. N., Loughlin, D. M., Desmond, S. M., Mahoney, K. J., Zacharias, B. L., . . . Allison, A. M. (2002). Implementation Issues for Consumer-Directed Programs: Comparing Views of Policy Experts, Consumers, and Representatives. *Journal of Aging & Social Policy, 14*(3-4), 95-118. doi:10.1300/J031v14n03_06

Slay, J. (2011). *Budgets and Beyond: Interim Report A review of the literature on personalisation and a framework for understanding co-production in the ‘Budgets and Beyond’ project*. Retrieved from London, UK: <http://www.thinklocalactpersonal.org.uk/_library/Resources/Personalisation/TLAP/Budgets_and_Beyond_November_2011_Final.pdf>

Social Interface. (2007). *A survey on the implementation of the current Direct Payments Scheme in Wales - Final Report*. Retrieved from Wales:

Solovieva, T. I., Wallsh, R. T., Hendricks, D. J., & Dowler, D. L. (2010). Workplace Personal Assistance Services for People with Disabilities: Making Productive Employment Possible. *Journal of Rehabilitation, 76*(4), 3-8 6p.

Spall, P., McDonald, C., & Zetlin, D. (2005). Fixing the system? the experience of service users of the quasi-market in disability services in Australia. *Health and Social Care in the Community, 13*(1), 56-63.

Spaulding-Givens, J. (2011). *Florida Self-Directed Care: An exploratory study of participants' characteristics, goals, service utilization, and outcomes.* (3483613 Ph.D.), The Florida State University, Ann Arbor. Retrieved from <http://search.proquest.com/docview/902623972?accountid=12309>

<http://fh6xn3yd3x.search.serialssolutions.com/?ctx_ver=Z39.88-2004&ctx_enc=info:ofi/enc:UTF-8&rfr_id=info:sid/ProQuest+Dissertations+%26+Theses+A%26I&rft_val_fmt=info:ofi/fmt:kev:mtx:dissertation&rft.genre=dissertations+%26+theses&rft.jtitle=&rft.atitle=&rft.au=Spaulding-Givens%2C+Jennifer&rft.aulast=Spaulding-Givens&rft.aufirst=Jennifer&rft.date=2011-01-01&rft.volume=&rft.issue=&rft.spage=&rft.isbn=9781124982854&rft.btitle=&rft.title=Florida+Self-Directed+Care%3A+An+exploratory+study+of+participants%27+characteristics%2C+goals%2C+service+utilization%2C+and+outcomes&rft.issn=&rft_id=info:doi/>

<http://fh6xn3yd3x.search.serialssolutions.com/?genre=article&sid=ProQ:&atitle=Florida+Self-Directed+Care%3A+An+exploratory+study+of+participants%27+characteristics%2C+goals%2C+service+utilization%2C+and+outcomes&title=Florida+Self-Directed+Care%3A+An+exploratory+study+of+participants%27+characteristics%2C+goals%2C+service+utilization%2C+and+outcomes&issn=&date=2011-01-01&volume=&issue=&spage=&author=Spaulding-Givens%2C+Jennifer> ProQuest Dissertations & Theses A&I database.

Spaulding-Givens, J. C., & Lacasse, J. R. (2015). Self-directed care: participants' service utilization and outcomes. *Psychiatric Rehabilitation Journal, 38*(1), 74-80.

Stainton, T., Asgarova, S., & Feduck, M. (2013). *A Comparison of Cost and Service Utilization Across Individualized and Traditional Funding Options Through Community Living British Columbia*. Retrieved from Vancouver, Canada:

Stainton, T., Boyce, S., & Phillips, C. J. (2009). Independence pays: A cost and resource analysis of direct payments in two local authorities. *Disability & Society, 24*(2), 161-172. doi:10.1080/09687590802652439

Stevens, M., Glendinning, C., Jacobs, S., Moran, N., Challis, D., Manthorpe, J., . . . Wilberforce, M. (2011). Assessing the Role of Increasing Choice in English Social Care Services. *Journal of Social Policy, 40*(2), 257-274. doi:<http://dx.doi.org/10.1017/S004727941000111X>

Sullivan, A. (2006). *Empowerment Initiatives Brokerage Service Quality and Outcome Evaluation Evaluation Report;* . Retrieved from Oregon, USA:

Tattrie, D., Stuart, C., Hanes, R., Ford, R., & Gyarmati, D. (2003). The Disability Supports Feasibility Study.

Tilly, J. (2007). *Consumer-directed, home and community services for adults with dementia*. Retrieved from Illinois, USA:

Tilly, J., & Wiener, J. M. (2001). Consumer-directed home and community services programs in eight states: policy issues for older people and government. *Journal of Aging & Social Policy, 12*(4), 1-26.

Timonen, V., Convery, J., & Cahill, S. (2006). Care Revolutions in the Making? A Comparison of Cash-for-Care Programmes in Four European Countries. *Ageing & Society, 26*(3), 455-474. doi:<http://dx.doi.org/10.1017/S0144686X0600479X>

Tyson, A., Brewis, R., Crosby, N., Hatton, C., Stanfield, J., Tomlinson, C. W., John; , & Wood, A. (2010). *A report on In Control's Third Phase: Evaluation and learning 2008-2009*. Retrieved from London:

Ungerson, C. (1999). Personal assistants and disabled people: An examination of a hybrid form of work and care. *Work, Employment and Society, 13*(4), 583-600.

Ungerson, C. (2004). Whose Empowerment and Independence? A Cross-National Perspective on 'Cash for Care' Schemes. *Ageing & Society, 24*(2), 189-212. doi:<http://dx.doi.org/10.1017/S0144686X03001508>

Ungerson, C. (2006). *The Management of Risk in Consumer Directed Care Schemes: A Cross National Analysis*. Paper presented at the International Sociological Association. <http://search.proquest.com/docview/61775023?accountid=12309>

<http://fh6xn3yd3x.search.serialssolutions.com/?ctx_ver=Z39.88-2004&ctx_enc=info:ofi/enc:UTF-8&rfr_id=info:sid/ProQ%3Asocabsshell&rft_val_fmt=info:ofi/fmt:kev:mtx:journal&rft.genre=conference&rft.jtitle=International+Sociological+Association&rft.atitle=The+Management+of+Risk+in+Consumer+Directed+Care+Schemes%3A+A+Cross+National+Analysis&rft.au=Ungerson%2C+Clare&rft.aulast=Ungerson&rft.aufirst=Clare&rft.date=2006-01-01&rft.volume=&rft.issue=&rft.spage=&rft.isbn=&rft.btitle=&rft.title=International+Sociological+Association&rft.issn=&rft_id=info:doi/>

<http://fh6xn3yd3x.search.serialssolutions.com/?genre=article&sid=ProQ:&atitle=The+Management+of+Risk+in+Consumer+Directed+Care+Schemes%3A+A+Cross+National+Analysis&title=International+Sociological+Association&issn=&date=2006-01-01&volume=&issue=&spage=&author=Ungerson%2C+Clare>

Vadapalli, D. K. (2009). Barriers and challenges in accessing social transfers and role of social welfare services in improving targeting efficiency: A study of conditional cash transfers. *Vulnerable Children and Youth Studies, 4*(Suppl 1), 41-54. doi:10.1080/17450120903111883

Valios, N. (2000). Wanted: caring employees. *Community Care, 1*, 20-21.

Victorian Auditor-General's, O. (2011). *Individualised funding for disability services*. Retrieved from <http://apo.org.au/resource/individualised-funding-disability-services>

Wadensten, B., & Ahlström, G. (2009). Ethical values in personal assistance: narratives of people with disabilities. *Nursing Ethics, 16*(6), 759-774 716p. doi:10.1177/0969733009341913

Walker, P., Hewitt, A., Bogenschutz, M., & Hall-Lande, J. (2009). Implementation of Consumer-Directed Services for Persons with Intellectual or Developmental Disabilities: A National Study. *Policy Research Brief, 20*.

Waters, J., & Hay, M. (2009). *Steering My Own Course: The introduction of Self-Directed Support in Cambridgeshire*. Retrieved from Cambridgeshire, UK:

Weech, S. (2009). Personalising health -- the West Sussex story. *Journal of Integrated Care, 17*(5), 26-30 25p.

Weinbach, H. (2010). 'Die haben uns sehr bestärkt in der sache, dass wir das schaffen...' Kriterien für die gestaltung von guten beratungsangeboten zum persönlichen budget. = 'We got utterly efficient encouragement to be able to succeed...' Criteria for the organisation of effective counselling on personal budget matters. *Vierteljahresschrift für Heilpädagogik und ihre Nachbargebiete, 79*(3), 212-223.

Wiesel, I., Laragy, C., Gendera, S., Fisher, K., Jenkinson, S., Hill, T., . . . Bridge, C. (2015). Moving to my home: housing aspirations, transitions and outcomes of people with disability.

Wilberforce, M., Glendinning, C., Challis, D., Fernandez, J. L., Jacobs, S., Jones, K., . . . Netten, A. (2011). Implementing Consumer Choice in Long‐term Care: The Impact of Individual Budgets on Social Care Providers in England. *Social Policy & Administration, 45*(5), 593-612.

Williams, V., Ponting, L., & Ford, K. (2009). 'I do like the subtle touch': Interactions between people with learning difficulties and their personal assistants. *Disability & Society, 24*(7), 815-828. doi:<http://dx.doi.org/10.1080/09687590903283407>

Williams, V., & Porter, S. (2015). The Meaning of 'choice and control' for People with Intellectual Disabilities who are Planning their Social Care and Support. *Journal of Applied Research in Intellectual Disabilities*. doi:10.1111/jar.12222

Zhang, H. (2015). The emergence of personalized service for disabled people in Sichuan Province, China. *International Social Work*, 0020872815574134.
