# Supplementary material for: Individualized funding interventions to improve health and social care outcomes for people with a disability: A mixed‐methods systematic review
Source: Campbell Syst Rev. 2019 Jul 19;15(1-2):e1008. doi: 10.4073/csr.2019.3 (PMC8356501; doi:10.4073/csr.2019.3)
Supplement: Supplementary file 6 — Supporting information [file CL2-15-e1008-s006.docx]

# Appendix 6: Risk of bias in included quantitative studies

| \| **Study:** Beatty (1998) \| **Quality and Risk of bias scores for Cross-Sectional Studies** \| \| --- \| --- \| | | | |
| --- | --- | --- | --- | --- | --- |
| **Criteria** | **Yes** | **No** | **Other (CD, NR, NA)*** |
| 1. Was the research question or objective in this paper clearly stated? | x |  |  |
| 2. Was the study population clearly specified and defined? | x |  |  |
| 3. Was the participation rate of eligible persons at least 50%? | x |  |  |
| 4. Were all the subjects selected or recruited from the same or similar populations (including the same time period)? Were inclusion and exclusion criteria for being in the study prespecified and applied uniformly to all participants? | x |  |  |
| 5. Was a sample size justification, power description, or variance and effect estimates provided? |  | x |  |
| 6. For the analyses in this paper, were the exposure(s) of interest measured prior to the outcome(s) being measured? |  | x | As per guidance |
| 7. Was the timeframe sufficient so that one could reasonably expect to see an association between exposure and outcome if it existed? | x |  |  |
| 8. For exposures that can vary in amount or level, did the study examine different levels of the exposure as related to the outcome (e.g., categories of exposure, or exposure measured as continuous variable)? |  |  | NA |
| 9. Were the exposure measures (independent variables) clearly defined, valid, reliable, and implemented consistently across all study participants? |  |  | NA |
| 10. Was the exposure(s) assessed more than once over time? |  |  | NA |
| 11. Were the outcome measures (dependent variables) clearly defined, valid, reliable, and implemented consistently across all study participants? | x |  | . |
| 12. Were the outcome assessors blinded to the exposure status of participants? |  | x |  |
| 13. Was loss to follow-up after baseline 20% or less? |  |  | NA |
| 14. Were key potential confounding variables measured and adjusted statistically for their impact on the relationship between exposure(s) and outcome(s)? |  | x |  |

| **Quality Rating (Good, Fair, or Poor) (see guidance)** |
| --- |
| Rater #1 initials: 6/10 (4 NA) = 60% - Fair |
| Rater #2 initials: |
| Additional Comments (If POOR, please state why): |

*CD, cannot determine; NA, not applicable; NR, not reported

| **Quality and Risk of bias scores (Cochrane)** | **Study Name** Personal assistance for people with physical disabilities: consumer-direction and satisfaction with services | |
| --- | --- | --- |
|  | Score: (High, Moderate, Low) | Support for judgement:  (Quote, Email communication, Comment) |
| Selection bias.  Random sequence generation  Allocation concealment | Low  NA (low)  NA (low) | Participants self-selected by returning contact details to research team |
| Performance bias.  Blinding of participants and personnel Assessments should be made for each main outcome (or class of outcomes) | Low | Not blinded |
| Detection bias.  Blinding of outcome assessment  Assessments should be made for each main outcome (or class of outcomes) | Low | Not blinded |
| Attrition bias.  Incomplete outcome data  Assessments should be made for each main outcome (or class of outcomes) | Low | Response rate not reported. |
| Reporting bias.  Selective reporting | High | All variables on the PASI satisfaction tool were reported. |
| Other bias / limitations.  Inappropriate influence from funders  Cross over of between intervention and control  Other | High | A declaration was made on the lack of a financial conflict of interest. |

| \| **Study:** Benjamin (2000) \| **Quality and Risk of bias scores for Cross-Sectional Studies** \| \| --- \| --- \| | | | |
| --- | --- | --- | --- | --- | --- |
| **Criteria** | **Yes** | **No** | **Other (CD, NR, NA)*** |
| 1. Was the research question or objective in this paper clearly stated? | x |  |  |
| 2. Was the study population clearly specified and defined? | x |  |  |
| 3. Was the participation rate of eligible persons at least 50%? | x |  |  |
| 4. Were all the subjects selected or recruited from the same or similar populations (including the same time period)? Were inclusion and exclusion criteria for being in the study prespecified and applied uniformly to all participants? | x |  |  |
| 5. Was a sample size justification, power description, or variance and effect estimates provided? |  | x |  |
| 6. For the analyses in this paper, were the exposure(s) of interest measured prior to the outcome(s) being measured? |  | x | As per guidance for x-sectional |
| 7. Was the timeframe sufficient so that one could reasonably expect to see an association between exposure and outcome if it existed? |  |  | CD |
| 8. For exposures that can vary in amount or level, did the study examine different levels of the exposure as related to the outcome (e.g., categories of exposure, or exposure measured as continuous variable)? |  |  | NA |
| 9. Were the exposure measures (independent variables) clearly defined, valid, reliable, and implemented consistently across all study participants? |  |  | NA |
| 10. Was the exposure(s) assessed more than once over time? |  |  | NA |
| 11. Were the outcome measures (dependent variables) clearly defined, valid, reliable, and implemented consistently across all study participants? |  |  |  |
| 12. Were the outcome assessors blinded to the exposure status of participants? |  | x |  |
| 13. Was loss to follow-up after baseline 20% or less? |  |  | NA |
| 14. Were key potential confounding variables measured and adjusted statistically for their impact on the relationship between exposure(s) and outcome(s)?  All analyses incorporated sampling weights and accounted for design effects (Kish 1967) using the Stata statistical software package (StataCorp 1997). | x |  |  |

| **Quality Rating (Good, Fair, or Poor) (see guidance)** |
| --- |
| Rater #1 initials: 7/10 (4 NA) = 70% - Good |
| Rater #2 initials: |
| Additional Comments (If POOR, please state why): |

*CD, cannot determine; NA, not applicable; NR, not reported

| **Quality and Risk of bias scores (Cochrane)** | **Study Name** | |
| --- | --- | --- |
|  | Score: (High, Moderate, Low) | Support for judgement:  (Quote, Email communication, Comment) |
| Selection bias.  Random sequence generation  Allocation concealment | Moderate | While the researchers could not control for who was assigned PAM or CDM, the sample was randomly selected from the two cohorts  This was not an RCT so NA  This was not an RCT so NA. Not possible to conceal as clients already knew which package they were receiving. |
| Performance bias.  Blinding of participants and personnel Assessments should be made for each main outcome (or class of outcomes) | High risk | Clients knew which model they were receiving |
| Detection bias.  Blinding of outcome assessment  Assessments should be made for each main outcome (or class of outcomes) | High risk | Assessors knew which model the participants were in receipt of |
| Attrition bias.  Incomplete outcome data  Assessments should be made for each main outcome (or class of outcomes) | Low risk | Data only collected once so we can assume dataset complete since it was not reported otherwise |
| Reporting bias.  Selective reporting | Low risk | It appears that all intended outcomes were reported on. |
| Other bias / limitations.  Inappropriate influence from funders  Cross over of between intervention and control  Other | Low risk |  |

| \| **Study:** Conroy (2002) \| **Quality and Risk of bias scores for Cross-Sectional Studies** \| \| --- \| --- \| | | | |
| --- | --- | --- | --- | --- | --- |
| **Criteria** | **Yes** | **No** | **Other (CD, NR, NA)*** |
| 1. Was the research question or objective in this paper clearly stated? | x |  |  |
| 2. Was the study population clearly specified and defined? |  | x |  |
| 3. Was the participation rate of eligible persons at least 50%? | x |  |  |
| 4. Were all the subjects selected or recruited from the same or similar populations (including the same time period)? Were inclusion and exclusion criteria for being in the study prespecified and applied uniformly to all participants? | x |  |  |
| 5. Was a sample size justification, power description, or variance and effect estimates provided? |  | x |  |
| 6. For the analyses in this paper, were the exposure(s) of interest measured prior to the outcome(s) being measured? |  |  | NA |
| 7. Was the timeframe sufficient so that one could reasonably expect to see an association between exposure and outcome if it existed? | x |  |  |
| 8. For exposures that can vary in amount or level, did the study examine different levels of the exposure as related to the outcome (e.g., categories of exposure, or exposure measured as continuous variable)? |  |  | NA |
| 9. Were the exposure measures (independent variables) clearly defined, valid, reliable, and implemented consistently across all study participants? |  |  | NA |
| 10. Was the exposure(s) assessed more than once over time? |  |  | NA |
| 11. Were the outcome measures (dependent variables) clearly defined, valid, reliable, and implemented consistently across all study participants? | x |  | They were well described and where available reliability scores provided |
| 12. Were the outcome assessors blinded to the exposure status of participants? |  | x |  |
| 13. Was loss to follow-up after baseline 20% or less? |  | x | 31% |
| 14. Were key potential confounding variables measured and adjusted statistically for their impact on the relationship between exposure(s) and outcome(s)? |  | x |  |

| **Quality Rating (Good, Fair, or Poor) (see guidance)** |
| --- |
| Rater #1 initials: 6/10 (4 NA) 60% Fair |
| Rater #2 initials: |
| Additional Comments (If POOR, please state why): |

*CD, cannot determine; NA, not applicable; NR, not reported

| \| **Study:** Brown (2007) \| **Quality and Risk of bias scores for Cross-Sectional Studies** \| \| --- \| --- \| | | | |
| --- | --- | --- | --- | --- | --- |
| **Criteria** | **Yes** | **No** | **Other (CD, NR, NA)*** |
| 1. Was the research question or objective in this paper clearly stated? | x |  |  |
| 2. Was the study population clearly specified and defined? | x |  |  |
| 3. Was the participation rate of eligible persons at least 50%? | x |  |  |
| 4. Were all the subjects selected or recruited from the same or similar populations (including the same time period)? Were inclusion and exclusion criteria for being in the study prespecified and applied uniformly to all participants? | x |  |  |
| 5. Was a sample size justification, power description, or variance and effect estimates provided? | x |  |  |
| 6. For the analyses in this paper, were the exposure(s) of interest measured prior to the outcome(s) being measured? |  | x | As per guidance |
| 7. Was the timeframe sufficient so that one could reasonably expect to see an association between exposure and outcome if it existed? | x |  | 9 months |
| 8. For exposures that can vary in amount or level, did the study examine different levels of the exposure as related to the outcome (e.g., categories of exposure, or exposure measured as continuous variable)? |  |  | NA |
| 9. Were the exposure measures (independent variables) clearly defined, valid, reliable, and implemented consistently across all study participants? |  |  | NA |
| 10. Was the exposure(s) assessed more than once over time? |  |  | NA |
| 11. Were the outcome measures (dependent variables) clearly defined, valid, reliable, and implemented consistently across all study participants? | x |  |  |
| 12. Were the outcome assessors blinded to the exposure status of participants? |  | x |  |
| 13. Was loss to follow-up after baseline 20% or less? |  | x |  |
| 14. Were key potential confounding variables measured and adjusted statistically for their impact on the relationship between exposure(s) and outcome(s)? | x |  |  |

| **Quality Rating (Good, Fair, or Poor) (see guidance)** |
| --- |
| Rater #1 initials: 9/11 (3 NA) = 82% - Good |
| Rater #2 initials: |
| Additional Comments (If POOR, please state why): |

*CD, cannot determine; NA, not applicable; NR, not reported

| **Quality and Risk of bias scores (Cochrane)** | **Study Name** | |
| --- | --- | --- |
|  | Score: (High, Moderate, Low) | Support for judgement:  (Quote, Email communication, Comment) |
| **Selection bias.**  Random sequence generation  Allocation concealment | Moderate risk  Unclear risk | No details of random sequence generation. But randomisation was undertaken. In general, the states used a combination of direct mailings, telephone calls, and home visits to inform all eligible beneficiaries about the opportunity to participate in the demonstration. Generally, within about one week of each beneficiary’s enrollment, MPR conducted a baseline telephone interview with the beneficiary (or with a knowledgeable proxy respondent) and then randomly assigned the beneficiary to the treatment group (with the opportunity to participate in Cash and Counseling) or to the control group (to rely on PCS or HCBS as usual).  No such detail reported |
| Performance bias.  Blinding of participants and personnel Assessments should be made for each main outcome (or class of outcomes) | High risk | Not possible |
| Detection bias.  Blinding of outcome assessment  Assessments should be made for each main outcome (or class of outcomes) | High risk | No such detail reported but not likely to be possible to blind assessors due to nature of intervention |
| Attrition bias.  Incomplete outcome data  Assessments should be made for each main outcome (or class of outcomes) | Unclear risk | At 12 months after enrolment 21.2% in Arkansas, 33.6% in Florida and 31.8% in New Jersey had disenrolled.  The majority of these voluntary disenrollees in all three states  had never started receiving an allowance. |
| Reporting bias.  Selective reporting | Low risk | “Only differences that were statistically significant at the .05 level were considered to be evidence of program effects.  Patterns of results across measures and subgroups also were used to assess whether statistically significant differences were likely to reflect true program effects or chance differences.” |
| Other bias / limitations.  Inappropriate influence from funders  Cross over of between intervention and control  Other |  |  |

| \| **Study:** Caldwell (2007) \| **Quality and Risk of bias scores for Cross-Sectional Studies** \| \| --- \| --- \| | | | |
| --- | --- | --- | --- | --- | --- |
| **Criteria** | **Yes** | **No** | **Other (CD, NR, NA)*** |
| 1. Was the research question or objective in this paper clearly stated? | x |  |  |
| 2. Was the study population clearly specified and defined? | x |  |  |
| 3. Was the participation rate of eligible persons at least 50%? |  | x | Control – 21% |
| 4. Were all the subjects selected or recruited from the same or similar populations (including the same time period)? Were inclusion and exclusion criteria for being in the study prespecified and applied uniformly to all participants? | x |  |  |
| 5. Was a sample size justification, power description, or variance and effect estimates provided? |  | x |  |
| 6. For the analyses in this paper, were the exposure(s) of interest measured prior to the outcome(s) being measured? |  | x | As per guidance |
| 7. Was the timeframe sufficient so that one could reasonably expect to see an association between exposure and outcome if it existed? | x |  | For intervention group |
| 8. For exposures that can vary in amount or level, did the study examine different levels of the exposure as related to the outcome (e.g., categories of exposure, or exposure measured as continuous variable)? |  |  | NA |
| 9. Were the exposure measures (independent variables) clearly defined, valid, reliable, and implemented consistently across all study participants? |  |  | NA |
| 10. Was the exposure(s) assessed more than once over time? |  |  | NA |
| 11. Were the outcome measures (dependent variables) clearly defined, valid, reliable, and implemented consistently across all study participants? | x |  |  |
| 12. Were the outcome assessors blinded to the exposure status of participants? |  | x |  |
| 13. Was loss to follow-up after baseline 20% or less? |  |  | NA |
| 14. Were key potential confounding variables measured and adjusted statistically for their impact on the relationship between exposure(s) and outcome(s)? | x |  | See notes on potential effects of attrition |

| **Quality Rating (Good, Fair, or Poor) (see guidance)** |
| --- |
| Rater #1 initials: 6/10 (4 NA) = 60% - Fair |
| Rater #2 initials: |
| Additional Comments (If POOR, please state why): |

*CD, cannot determine; NA, not applicable; NR, not reported

| **Study:** Glendinning (2008) | **Cochrane - Quality and Risk of bias scores for Randomised Controlled Trial** | |
| --- | --- | --- |
|  | Score: (Low) | Support for judgement:  Due to the complex social intervention the usual standards expected in a clinical RCT were not feasible. However every effort was made to adjust for potential bias. |
| Selection bias.  Random sequence generation  Allocation concealment | Yes  Not reported | An analysis of sample representative and other bias can be seen section 4.4 (page 43) “no significant differences between the IB and comparison groups, with the sole exceptions of prior receipt of carer support (where levels of service receipt were in any case very low in both groups) and whether the user posed a risk to others” |
| Performance bias.  Blinding of participants and personnel Assessments should be made for each main outcome (or class of outcomes) | No (not possible) |  |
| Detection bias.  Blinding of outcome assessment  Assessments should be made for each main outcome (or class of outcomes) | No (not possible) |  |
| Attrition bias.  Incomplete outcome data  Assessments should be made for each main outcome (or class of outcomes) | Both incomplete data and data by proxy respondents were considered during the analysis.  Proxy responses were removed from analysis to see if results affected. | A subgroup called ‘IB-accepted group’ was created to represent those within the intervention group who accepted the IB (n – 458). Comparisons were drawn between this group and the comparison group where possible.  Number of respondents were always highlighted for each outcome being reported and these varied considerably.  Section c.2 (page 285 – 286) details the impact of proxy responses.  In Chapter 6 we identified a number of associations between outcomes and IBs which ceased to be statistically significant once proxies were excluded. In the sample as a whole the proportion who reported feeling in control of their daily lives was 48 per cent in the IB group (n=493) and 41 per cent in the comparison group  (n=437). Excluding proxies the proportion who reported feeling in control was 55 per cent in the IB group (n=287) and 49 per cent in the comparison group (n=299). |
| Reporting bias.  Selective reporting | All intended outcomes were reported for whole sample and by subgroup. |  |
| Other bias / limitations.  Inappropriate influence from funders  Cross over of between intervention and control  Other | There was cross over between control and intervention (approximately 52 people) | There are several reasons why our sample may not be representative of the population of community (i.e. excluding those in residential care) social care service users as a whole. In particular this could be through pilot sites selecting or excluding specific types of service users to be put forward for the trial15. An unrepresentative sample may also result from non-response and sample attrition discussed above. For more detail see pages 43 -45. |

| **Study:** Glendinning (2008) | **Quality and Risk of bias scores for Cross-Sectional Studies** |
| --- | --- |

| **Criteria** | **Yes** | **No** | **Other (CD, NR, NA)*** |
| --- | --- | --- | --- |
| 1. Was the research question or objective in this paper clearly stated? | x |  |  |
| 2. Was the study population clearly specified and defined? | x |  |  |
| 3. Was the participation rate of eligible persons at least 50%? | x |  |  |
| 4. Were all the subjects selected or recruited from the same or similar populations (including the same time period)? Were inclusion and exclusion criteria for being in the study prespecified and applied uniformly to all participants? | x |  |  |
| 5. Was a sample size justification, power description, or variance and effect estimates provided? | x |  |  |
| 6. For the analyses in this paper, were the exposure(s) of interest measured prior to the outcome(s) being measured? |  | x | As per guidance |
| 7. Was the timeframe sufficient so that one could reasonably expect to see an association between exposure and outcome if it existed? | x |  | 6 months |
| 8. For exposures that can vary in amount or level, did the study examine different levels of the exposure as related to the outcome (e.g., categories of exposure, or exposure measured as continuous variable)? |  |  | NA |
| 9. Were the exposure measures (independent variables) clearly defined, valid, reliable, and implemented consistently across all study participants? |  |  | NA |
| 10. Was the exposure(s) assessed more than once over time? |  |  | NA |
| 11. Were the outcome measures (dependent variables) clearly defined, valid, reliable, and implemented consistently across all study participants? | x |  |  |
| 12. Were the outcome assessors blinded to the exposure status of participants? |  | x |  |
| 13. Was loss to follow-up after baseline 20% or less? |  | x |  |
| 14. Were key potential confounding variables measured and adjusted statistically for their impact on the relationship between exposure(s) and outcome(s)? | x |  |  |

| **Quality Rating (Good, Fair, or Poor) (see guidance)** |
| --- |
| Rater #1 initials: 8/11 (3 NA) = 73% - Good |
| Rater #2 initials: |
| Additional Comments (If POOR, please state why): |

*CD, cannot determine; NA, not applicable; NR, not reported

| \| **Study:** Woolham (2013) \| **Quality and Risk of bias scores for Cross-Sectional Studies** \| \| --- \| --- \| | | | |
| --- | --- | --- | --- | --- | --- |
| **Criteria** | **Yes** | **No** | **Other (CD, NR, NA)*** |
| 1. Was the research question or objective in this paper clearly stated? |  | x |  |
| 2. Was the study population clearly specified and defined? | x |  |  |
| 3. Was the participation rate of eligible persons at least 50%? | x |  |  |
| 4. Were all the subjects selected or recruited from the same or similar populations (including the same time period)? Were inclusion and exclusion criteria for being in the study prespecified and applied uniformly to all participants? | x |  |  |
| 5. Was a sample size justification, power description, or variance and effect estimates provided? |  | x |  |
| 6. For the analyses in this paper, were the exposure(s) of interest measured prior to the outcome(s) being measured? |  | x | As per guidance |
| 7. Was the timeframe sufficient so that one could reasonably expect to see an association between exposure and outcome if it existed? |  |  | NR |
| 8. For exposures that can vary in amount or level, did the study examine different levels of the exposure as related to the outcome (e.g., categories of exposure, or exposure measured as continuous variable)? |  |  | NA |
| 9. Were the exposure measures (independent variables) clearly defined, valid, reliable, and implemented consistently across all study participants? |  |  | NA |
| 10. Was the exposure(s) assessed more than once over time? |  |  | NA |
| 11. Were the outcome measures (dependent variables) clearly defined, valid, reliable, and implemented consistently across all study participants? |  | x | Not for costs |
| 12. Were the outcome assessors blinded to the exposure status of participants? |  | x |  |
| 13. Was loss to follow-up after baseline 20% or less? |  |  | NA |
| 14. Were key potential confounding variables measured and adjusted statistically for their impact on the relationship between exposure(s) and outcome(s)? |  | x | Broadly comparable groups |

| **Quality Rating (Good, Fair, or Poor) (see guidance)** |
| --- |
| Rater #1 initials: 3/10 (4 NA) = 30% - Poor |
| Rater #2 initials: |
| Additional Comments (If POOR, please state why):  The aims of the study are not clearly stated. While random assignment was used, the definition of the control group is ill-defined. There is no discussion of statistical power in relation to sample size. The two groups were considered broadly comparable on a number of demographic factors but no statistical data is presented. |

*CD, cannot determine; NA, not applicable; NR, not reported
