# Supplementary material for: Individualized funding interventions to improve health and social care outcomes for people with a disability: A mixed‐methods systematic review
Source: Campbell Syst Rev. 2019 Jul 19;15(1-2):e1008. doi: 10.4073/csr.2019.3 (PMC8356501; doi:10.4073/csr.2019.3)
Supplement: Supplementary file 7 — Supporting information [file CL2-15-e1008-s007.docx]

# Appendix 7: Risk of Bias and Quality in included qualitative studies

| **First Author (year)** | **CASP score** | **CerQual confidence score**  (High, Moderate, Low, Very low) |
| --- | --- | --- |
| Oliver (1992) | 3/10 | Moderate - This study was methodologically poor, the aims and outcomes are relevant to the review, the findings are well grounded in the data presented and the data was limited in quantity (1^st^ quartile of participants and 1^st^ quartile of coded data). However, the study was conducted when very little was known about personal budgets in the UK – rich data |
| Zarb  (1994) | 7/10 | Moderate - This study was methodologically sound, the aims and outcomes are relevant to the review, the findings are well grounded in the data presented and the data was rich in meaning and quantity (3^rd^ quartile of participants and 3^rd^ quartile of coded data), although some data was related to ineligible population and was excluded from analysis. |
| Malette (1996) | 7/10 | Moderate - This study was methodologically sound, the aims and outcomes are relevant to the review, the findings are well grounded in the data presented and the data was rich in meaning and quantity (3^rd^ quartile of participants and 4^th^ quartile of coded data). |
| Walker (1996) | 5/10 | Low - This study was fair methodologically, the aims and outcomes are relevant to the review, the discussion of findings lacks detail and the data was weak in terms of quantity and representativeness (2^nd^ quartile of participants and 1^st^ quartile of coded data). However, the study does give an early insight into consumer directed programmes. |
| Holman (1999) | 3.5/10 | Low - This study was poor methodologically (insufficient detail to assess), the aims and outcomes are relevant to the review, it was difficult to determine what findings related to what (limited raw) data, however the discussion was rich and provided useful insights into implementation issues at the early stage (2^nd^ quartile of participants and 1^st^ quartile of coded data). |
| Olmstead (1999) | 0.5/10 | Low - This study was very poor methodologically (insufficient detail to assess), the aims and outcomes are relevant to the review, the data was rich and findings are well grounded in the data, providing insights into the implementation of self-directed programs in the US at the very early stage of national roll-out, although limited in quantity and representativeness (1^st^ quartile of participants (no PwD directly involved) and 2^nd^ quartile of coded data). |
| Blumberg (2000) | 2/10 | Very Low - This study was poor methodologically (insufficient detail to assess), the aims and outcomes are relevant to the review, the data was rich although largely descriptive in nature and not linked well to other evidence and only represents one individual case (1^st^ quartile of participants and 1^st^ quartile of coded data). |
| Dawson (2000) | 4/10 | Low - This study was poor methodologically, the aims and outcomes are partly relevant to the review, although a lot of logistical and descriptive data was reported, although the design was not primarily qualitative, the findings seem to be grounded in the qualitative data with a particular focus on implementation, the data also makes a considerable contribution overall (2^nd^ quartile of participants and 3^rd^ quartile of coded data). |
| Glendinning (2000) | 7/10 | Moderate - This study was largely methodologically sound, the aims and outcomes are relevant to the review, the findings are well grounded in the data presented and the data was rich in meaning and quantity, providing early insight into direct payment schemes in the UK when relevant data was scarce (3^rd^ quartile of participants and represents the median number of coded text n = 148). |
| Leece (2000) | 3/10 | Low - This study was very poor methodologically, the aims and outcomes are somewhat relevant to the review, the findings however are well grounded in the data presented and the data provides early insight into direct payment schemes in the UK when relevant data was scarce, although it was limited in quantity and representativeness (1^st^ quartile of participants and 1^st^ quartile of coded data). |
| Pearson (2000) | 6.5/10 | Moderate - This study was methodologically sound, the aims and outcomes are relevant to the review. While the paper was somewhat disjointed it provides insightful findings into user experiences of using direct payments under different governing structures albeit limited in quantity and representativeness (2^nd^ quartile of participants and 1^st^ quartile of coded data). |
| Witcher (2000) | 8/10 | Moderate - This study was methodologically robust, the aims and outcomes are relevant to the review, the findings are well grounded in the data presented and the data was rich in meaning (4^th^ quartile of coded data), deciphering a very complex set of arrangements across Scotland, although the study was limited in terms of representativeness (2^nd^ quartile of participants). |
| Smith  (2001) | 3/10 | Low - This study was very poor methodologically (insufficient information to assess), while the aims and outcomes are somewhat relevant to the review, the findings are presented in an inaccessible manner. Although much of the data presented was not relevant, the relevant data was sizeable (3^rd^ quartile of coded data) with sizeable participation (2^nd^ quartile of participants). |
| Carmichael (2002) | 6.5/10 | Moderate - This study has methodological limitations (lacking detail) but the aims and outcomes are relevant to the review. Furthermore the findings are well grounded in the data presented and the data was rich in meaning and quantity (3^rd^ quartile of participants and 2^nd^ quartile of coded data). |
| Conroy (2002) | 7/10 | Moderate - This study was methodologically sound, the aims and outcomes are relevant to the review, the findings are well grounded in the data presented, although it was difficult at times to disaggregate quantitative and qualitative data. The data was rich in meaning and quantity (3^rd^ quartile of participants and 3^rd^ quartile of coded data). |
| Eckert (2002) | 6.5/10 | High – Although there are some methodological limitations (for example insufficient information on ethics) this study represents a large number of titles reporting on the Cash and Counseling demonstration sites. The aims and outcomes are relevant to the review, the findings are well grounded in the data presented, and the data was extremely rich in meaning and quantity (4^th^ quartile of participants and 4^th^ quartile of coded data). |
| Young (2003) | 8/10 | Moderate - This study was methodologically sound, the aims were unclear but the outcomes are relevant to the review, the findings are well grounded in the data presented. The sample was considerable with a moderate richness in data presented (3^rd^ quartile of participants and 2^nd^ quartile of coded data). |
| Breda (2004) | 7.5/10 | Moderate - This study was methodologically sound, the aims were broad but the specific research questions were relevant to the review, the findings are well grounded in the data presented. The data was rich in meaning and quantity (3^rd^ quartile of participants and 4^th^ quartile of coded data) providing rich insights into the experience of people from Belgium. |
| Jordan (2004) | 1.5/10 | Very low - This study was very poor methodologically (insufficient information to assess), while the aims were not particularly relevant, the reported findings were relevant to the review. The number of study participants were considerable (3^rd^ quartile of participants), although no PwD were involved. Furthermore, the richness and quantity of data was limited (1^st^ quartile of coded data). |
| Stainton (2004) | 7.5/10 | Moderate - This study was methodologically sound, the aims and outcomes are relevant to the review, the findings are well grounded in the data presented and the data was rich in meaning and quantity (2^nd^ quartile of participants and 2^nd^ quartile of coded data), conducted within two years of the introduction of personal budgets, these findings were very insightful at the time, particularly in Wales and the UK. |
| Emslie (2005) | 7/10 | Moderate - This study was methodologically sound, the aims were not completely in line with those of the review but the findings were relevant and were well grounded in the data presented. The data was rich in meaning and quantity (4^th^ quartile of participants and 4^th^ quartile of coded data) particularly around implementation. However the data was difficult to code due to the nature of reporting and not all data was eligible for analysis (for example, data relating to minors). |
| Rosenberg (2005) | 5.5/10 | Moderate - This study was fair methodologically; the aims were in line with the implementation element of the review and were well grounded in the data presented. The data was rich in meaning and quantity (4^th^ quartile of participants and 3^rd^ quartile of coded data) particularly around implementation of personal budgets in the UK at the early stages. No PwD were involved in the research directly. |
| Butler (2006) | 8.5/10 | Moderate - This study was methodologically robust, the aims were not entirely in line with the reviews but the findings were relevant and were well grounded in the data presented. While the focus was mainly on family members as representative for people with developmental disabilities, the data was rich in meaning and quantity (4^th^ quartile of participants and 3^rd^ quartile of coded data). |
| Sanderson (2006) | 4.5/10 | Low - This study was poor methodologically (insufficient information to assess), while the aims and outcomes are somewhat relevant to the review, the findings are limited in richness and representativeness (1^st^ quartile of coded data and 2^nd^ quartile of participants). |
| Speed (2006) | 6.5/10 | Low - This study was moderate methodologically, the aims and outcomes were in line with the review, while the findings provided a unique and rich insight into the first-hand experience of one individual case study, however the representativeness and quantity of data was limited (1^st^ quartile of participants and 1^st^ quartile of coded data). |
| Alakeson (2007) | 2/10 | Very low - This study was very poor methodologically (insufficient information to assess), while the aims were somewhat relevant, the reported findings were relevant to the review. The number of study participants were considerable (3^rd^ quartile of participants) and it highlighted the unique perspective of mental health users. However, the richness and quantity of data was limited (1^st^ quartile of coded data). |
| Caldwell (2007) | 7.5/10 | Moderate - This study was methodologically sound, the aims were in line with the reviews and the findings were relevant and were well grounded in the data presented. The focus was mainly on family members as representative for people with intellectual/developmental disabilities, the data was limited in meaning and quantity (1^st^ quartile of participants and 1^st^ quartile of coded data). |
| Dimitriadis (2007) | 6/10 | Moderate - This study was moderate methodologically, the aims were largely in line with the reviews and the findings were relevant. While much of the data presented was descriptive, the relevant findings were well grounded in the data presented, and were rich in quantity (3^rd^ quartile of coded data). However the representativeness was limited (1^st^ quartile of participants). |
| Adams (2008) | 5.5/10 | Moderate - This study was fair methodologically, the aims were and outcomes were relevant to the review, the findings are well grounded in the data presented. The sample was considerable, as were the richness and quantity (4^th^ quartile of participants and 3^rd^ quartile of coded data). |
| Daly  (2008) | 8/10 | Moderate - This study was methodologically rigorous, the aims and outcomes are relevant to the review and the findings are well grounded in the rich data presented. However the representativeness and quantity was limited and this cohort of people were already researched as part of the large scale IBSEN study (2^nd^ quartile of participants and 1^st^ quartile of coded data). |
| Dinora (2008) | 8/10 | Low – As a mixed methods study the methodology was robust, the aims and outcomes were relevant to the review, however the qualitative findings were limited in terms of richness (based solely on open-ended questions) and while the reach was considerable, the quantity of relevant data was minimal (4^th^ quartile of participants and 1^st^ quartile of coded data). |
| Glendinning (2008) | 9/10 | High - This study is methodologically rigorous, the aims and outcomes are relevant to the review, the findings are well grounded in the data presented and the data is rich in meaning and quantity (4^th^ quartile of participants and 4^th^ quartile of coded data). |
| Homer (2008) | 6.5/10 | Moderate - This study was methodologically sound, the aims and outcomes were relevant to the review and the findings were well grounded in the data presented. The sample was considerable (even with ineligible data removed), as were the richness and quantity (2^nd^ quartile of participants and 3^rd^ quartile of coded data), particularly for an under-researched part of the UK (Scotland). |
| Lord  (2008) | 6.5/10 | Moderate - This study was methodologically sound, the aims and outcomes were relevant to the review and the findings were well grounded in the data presented. The sample was considerable (given the document analysis component), while the quantity was limited (4^th^ quartile of participants and 2^nd^ quartile of coded data). |
| Shaw (2008) | 2.5/10 | Low - This study was very poor methodologically, the aims and outcomes were not particularly in line with the review, however the data does represent an under-researched population (deafblind) and the findings were grounded in rich qualitative data (2^nd^ quartile of participants and 3^rd^ quartile of coded data). |
| Coyle (2009) | 6.5/10 | Moderate - This study was methodologically sound, the aims and outcomes were relevant to the review and the findings were well grounded in the data presented. However the sample and quantity of data was limited (1^st^ quartile of participants and 2^nd^ quartile of coded data). |
| Rogers (2009) | 6/10 | Moderate - This study was moderate methodologically, the aims and outcomes were relevant to the review and the findings were well grounded in the data presented. However the sample and quantity of data was limited (1^st^ quartile of participants and 2^nd^ quartile of coded data), however the sample did represent an under-researched group (mental health users). |
| Sayles Wallace (2009) | 7.5/10 | Moderate - This study was methodologically robust, the aims were in line with those of the review, the findings were well grounded in the data presented. While the sample was small (1^st^ quartile of participants), capturing the parent representative perspective for adult children with a severe intellectual disability, the data was rich in meaning and quantity (3^rd^ quartile of coded data). |
| Eost- Telling (2010) | 6.5/10 | High - This study was methodologically sound, the aims and outcomes were relevant to the review and the findings were well grounded in the data presented. The sample was considerable, representing both user experience and staff implementation perspective. Furthermore, data was rich in quality and quantity (3^rd^ quartile of participants and 4^th^ quartile of coded data). |
| Kinnaird (2010) | 7/10 | Moderate - This study was methodologically robust, the aims and outcomes were relevant to the review and the findings were well grounded in the data presented. While the sample was limited, it represented an under-researched cohort (people with dementia) and staff implementation perspective. The data was rich in quality and quantity (2^nd^ quartile of participants and 4^th^ quartile of coded data). |
| Leahy  (2010) | 6/10 | Moderate - This study was moderate methodologically, the aims and outcomes were relevant to the review and the findings were well grounded in the data presented. The sample was limited, but the data was rich in quality and quantity (2^nd^ quartile of participants and 4^th^ quartile of coded data). |
| Priestley (2010) | 4.5/10 | Moderate - This study was poor methodologically, the aims and outcomes were not particularly in line with the review and a lot of descriptive and 3^rd^ party perspectives were reported. However, where relevant findings were reported, they were grounded in the data, represented a sizeable number of participants and were rich in quantity (4^th^ quartile of participants and 4^th^ quartile of coded data). |
| Vinton (2010) | 8/10 | Low - This study was methodologically sound, the aims and outcomes were relevant to the review and the findings were well grounded in the data presented. Although the sample was considerable, there was limited qualitative data in both richness and quantity (3^rd^ quartile of participants and 1^st^ quartile of coded data). |
| Williams (2010) | 6/10 | Very low – Although this study is methodologically moderate, the aims and outcomes only partially meet the inclusion criteria, however the social workers interviewed do discuss implementation issues, and findings are grounded in the data collected. The data is limited in richness and quantity (1^st^ quartile of participants and 1^st^ quartile of coded data). |
| Wilson (2010) | 5/10 | Moderate - This study was fair methodologically; the aims and outcomes were in line with the review; however the data is limited in richness, since it simply represents responses to open-ended questions and other comments. However, the sample was considerable and the data plentiful (4^th^ quartile of participants and 4^th^ quartile of coded data). |
| Campbell (2011) | 8.5/10 | Moderate - This study was methodologically sound, the aims and outcomes were relevant to the review. The findings were difficult to code however, i.e. determining what was based on empirical data vs researcher interpretation OR lived experiences vs potential implementation challenges/facilitators. However the sample and quantity of data was plentiful (3^rd^ quartile of participants and 4^th^ quartile of coded data). |
| Hatton (2011) | 2.5/10 | Low - This study was very poor methodologically, but the aims and outcomes were in line with the review. The qualitative element of this study is limited in richness (comments and responses to open end questions), however the data does represent a large sample therefore captured a broad spectrum of views (4^th^ quartile of participants and 2^nd^ quartile of coded data). |
| Lambert (2011) | 7/10 | Moderate - This study was methodologically sound, the aims and outcomes were largely in line with the review, the findings were grounded in the data, which was rich in meaning and quantity (3^rd^ quartile of participants and 3^rd^ quartile of coded data). |
| Newbronner (2011) | 9/10 | High - This study was methodologically robust, the aims and outcomes were in line with the review, the findings were grounded in the data, which was rich in meaning and quantity (3^rd^ quartile of coded data). Half the sample had to be excluded but the remaining sample of mental health users was considerable (3^rd^ quartile of participants) |
| Ridley  (2011) | 6.5/10 | Moderate - This study was moderate methodologically, the aims and outcomes were largely in line with the review, the findings were grounded in the data, which was rich in meaning and quantity, with a considerable sample (4^th^ quartile of participants and 3^rd^ quartile of coded data). |
| Secker  (2011) | 2.5/10 | Very low - This study was very poor methodologically (insufficient information to assess). While the aims and outcomes were relevant to the review and the findings were limited in terms of richness, mainly based on survey comments. The sample was also modest in size (1^st^ quartile of participants and 2^nd^ quartile of coded data). |
| Rummery (2012) | 5/10 | Low - This study was fair methodologically. The aims and outcomes were not particularly in line with the review but the qualitative findings were relevant, particularly the focus group data. The sample size was bolstered by the mixed method approach (3^rd^ quartile of participants and 2^nd^ quartile of coded data). |
| Sheikh (2012) | 7/10 | High - This study was methodologically sound, the aims and outcomes were in line with the review, the findings were grounded in the data, which was rich in meaning and quantity, having been captured over a prolonged period of time, capturing changes over time (3^rd^ quartile of participants and 4^th^ quartile of coded data). |
| Gross  (2013) | 7.5/10 | Moderate - This study was methodologically sound, while the aims and outcomes were not particularly in line with the review, the findings were relevant, grounded in the data, and relatively rich in meaning and quantity (2^nd^ quartile of participants and 2^nd^ quartile of coded data). |
| Hatton (2013) | 2.5/10 | Moderate - This study was very poor methodologically, but the aims and outcomes were in line with the review. The qualitative element of this study is limited in richness (comments and responses to open end questions), however the data does represent a large sample therefore captured a broad spectrum of views (4^th^ quartile of participants and 3^rd^ quartile of coded data). |
| Rees  (2013) | 7/10 | Moderate - This study was methodologically sound, the aims and outcomes were in line with the review, the findings were relevant and grounded in the data, the sample was relatively modest but the data was rich in meaning and quantity (2^nd^ quartile of participants and 4^th^ quartile of coded data). |
| Bola  (2014) | 6/10 | Moderate - This study was moderate methodologically, the aims and outcomes were largely in line with the review although with a particular focus on uptake, the findings were relevant and grounded in the data, the sample represented an under researched group (mental health users) and the data was rich in meaning and quantity (3^rd^ quartile of participants and 4^th^ quartile of coded data). |
| Junne  (2014) | 6/10 | Moderate - This study was moderate methodologically, the aims and outcomes were largely in line with the review although with a particular focus on risk, the findings were relevant and grounded in the data, which was moderate in meaning and quantity (2^nd^ quartile of participants and 2^nd^ quartile of coded data). |
| Buchanan (2014) | 6.5/10 | Moderate - This study was moderate methodologically, the aims and outcomes were in line with the review although with a particular focus on risk. While the sample was small (1^st^ quartile of participants), the findings were relevant and grounded in the data, which was moderate in meaning and quantity (3^rd^ quartile of coded data). |
| Waters (2014) | 2/10 | Very low - This study was very poor methodologically. While the aims and outcomes were relevant to the review and the sample size considerable (4^th^ quartile of participants), the findings were limited in terms of richness, mainly based on survey comments. Very little qualitative data was presented (1^st^ quartile of coded data). |
| Coles  (2015) | 7.5/10 | Low - This study was methodologically sound, the aims and outcomes were not particularly in line with the review (focusing on parents managing budget on behalf of adult child with a disability). However relevant data was presented although it was limited meaning and quantity (1^st^ quartile of participants and 1^st^ quartile of coded data). |
| Glendinning (2015) | 9.5/10 | Moderate - This study was methodologically robust, the aims and outcomes were somewhat in line with the review although with a particular focus on the carers role, the findings were relevant and grounded in the data, but represented a relatively small sample and were moderate in meaning and quantity (2^nd^ quartile of participants and 1^st^ quartile of coded data). |
| Hamilton (2015) | 9.5/10 | High - This study was methodologically robust, the aims and outcomes were in line with the review, with a particular focus on mental health users, the findings were relevant and grounded in the data, which was rich in meaning and quantity (4^th^ quartile of participants and 3^rd^ quartile of coded data). |
| Jepson (2015) | 8.5/10 | Moderate - This study was methodologically robust, the aims and outcomes were in line with the review, with a particular focus on dementia and implementation issues when providing funding to recipients that do not have capacity to consent. The findings were relevant and grounded in the data, representing a considerable sample (4^th^ quartile of participants). The richness of data was somewhat limited however in depth and quantity (2^nd^ quartile of coded data). |
| Jones  (2015) | 8/10 | High - This study was methodologically robust, the aims and outcomes were largely in line with the review although the funding had a particular ‘accommodation’ focus. The findings were relevant and grounded in the data, which were rich in meaning and quantity (4^th^ quartile of participants and 4^th^ quartile of coded data). |
| Laragy (2015) | 7/10 | Moderate - This study was methodologically sound, the aims and outcomes were in line with the review with a particular focus on implementation. The findings were relevant and grounded in the data, however the sample size was limited and the richness of data was moderate in meaning and quantity (1^st^ quartile of participants and 2^nd^ quartile of coded data). |
| O’Brien (2015) | 6.5/10 | Moderate - This study was moderate methodologically, the aims and outcomes were in line with the review and the findings were relevant and grounded in the data. While the sample size was limited, it represented an under-researched population (Irish), the data was rich in meaning and quantity (1^st^ quartile of participants and 3^rd^ quartile of coded data). |
| Fleming (2016) | 9/10 | High - This research is extremely valuable, it utilises rigorous qualitative methodology to provide detailed assessment of individualised funding in Ireland. It provides pragmatic recommendations and how structures and process can be improved. In addition to highlighting how important these services are to people with a disability. (MH) While the sample size represented the median number of participants (n = 44), the data were rich in meaning and quantity (3^rd^ quartile of coded data). (PF) |
| McGuigan (2016) | 5/10 | Low - This study was fair methodologically, the aims and outcomes were in line with the review and the findings were relevant and grounded in the data. The sample size was moderate and the richness of data was limited due to the more quantitative approach to data collection (survey) (2^nd^ quartile of participants and 2^nd^ quartile of coded data). |

## Details of CASP Score

| **Oliver (1992)**  1 Was there a clear statement of the aims of the research? Yes  See table above  2 Is a qualitative methodology appropriate? Yes  3 Was the research design appropriate to address the aims of the research? Can’t tell  The authors do not give any justification for the chosen design. We do not know if they considered another approach.  4 Was the recruitment strategy appropriate to the aims of the research? Can’t tell  There is no detail provided on the recruitment strategy.  5 Was the data collected in a way that addressed the research issue? No  There is a real lack on detail on methods used.  6 Has the relationship between researcher and participants been adequately considered? Yes 0.5  “While the evaluators are completely independent, it was considered inappropriate for the evaluation to be carried out without close and continuous consultation with GAD and local Personal Assistant Scheme users. Consequently, rather than simply produce periodic reports, the evaluators have held regular meetings with GAD with a view to feeding issues arising from the evaluation into their own work, to monitor progress of the evaluation itself, and to review priorities.” (page 5) While the relationship was considered, there is a lack of detail provided on how consultation influenced data collection or analysis.  7 Have ethical issues been taken into consideration? No  Ethical considerations not reported.  8 Was the data analysis sufficiently rigorous? No  There is a lack of detail on analysis. It is stated on page 6 that another report “Greenwich Personal Assistance Schemes: Findings from Personal Assistance Scheme Users Survey (September, 1992)” provides more detail on analysis, however this paper was not located.  9 Is there a clear statement of findings? No  Policy implications are discussed but reference to findings from current study are very brief.  10 How valuable is the research? Moderate 0.5  This study was conducted in 1992 when very little was known about personal budgets in the UK, this study provided a unique and useful insight into the use of PA advisors and how their role potentially strengthened the potential success of self-directed supports.  Overall score – 3/10 |
| --- |
| **Zarb (1994)**  1 Was there a clear statement of the aims of the research? Yes  See table above  2 Is a qualitative methodology appropriate? Yes  3 Was the research design appropriate to address the aims of the research? Yes  There is detailed rationale for the research design and method.  4 Was the recruitment strategy appropriate to the aims of the research? Yes  The authors gave detailed description of the recruitment strategy aiming to have a representative sample that was matched with a comparative group.  5 Was the data collected in a way that addressed the research issue? Yes  The interview data was collected by disabled researchers and based on semi-structured interview schedules.  6 Has the relationship between researcher and participants been adequately considered? Yes 0.5  Freelance disabled interviewers were recruited which indicates consideration given to the relationship between researchers and participants. However the rationale is not explicitly detailed. Some detail is provided on efforts to validate the findings with research participants.  7 Have ethical issues been taken into consideration? No  Ethical considerations not reported.  8 Was the data analysis sufficiently rigorous? No  There is a lack of detail on the qualitative analysis.  9 Is there a clear statement of findings? Yes  Chapter 8 is dedicated to summarising the findings, with final conclusions outlined on pages 188 – 190.  10 How valuable is the research? Moderate  This is a detailed study comparing personal budgets and traditional service provision. Rich and detailed qualitative data is provided.  Overall score – 7/10 |
| **Malette (1996)**  1 Was there a clear statement of the aims of the research? Yes  2 Is a qualitative methodology appropriate? Yes  3 Was the research design appropriate to address the aims of the research? Yes  4 Was the recruitment strategy appropriate to the aims of the research? Yes  5 Was the data collected in a way that addressed the research issue?” Yes  Various methods were employed.  6 Has the relationship between researcher and participants been adequately considered? Yes 0.5  Yes, this is considered particularly in relation to the observations, trying to be as unobtrusive as possible. Also through a participatory follow-up session participants were able to contribute and validate the initial findings. However, although the author took these steps the relationship between researcher and participant is not explicitly explored.  7 Have ethical issues been taken into consideration? Can’t tell  Not reported  8 Was the data analysis sufficiently rigorous? Yes  9 Is there a clear statement of findings? Yes  Through discussion  10 How valuable is the research? Moderate (0.5)  Overall score – 7/10 |
| **Walker (1996)**  1 Was there a clear statement of the aims of the research? No  The purpose of the study was to evaluate the consumer directed program but there was not a clear statement of aims.  2 Is a qualitative methodology appropriate? Yes  3 Was the research design appropriate to address the aims of the research? Yes 0.5  There is evidence of methodological rigour, for example, interviewing general informants in order to refine interview schedule before meeting program participants. However the author has not explicitly justified the methods used.  4 Was the recruitment strategy appropriate to the aims of the research? Yes  The authors gave detailed description of the recruitment strategy using but a random and targeted strategy in order to ensure diverse group / experiences of SDS.  5 Was the data collected in a way that addressed the research issue? Yes  The appendix is dedicated to providing methodological detail.  6 Has the relationship between researcher and participants been adequately considered? No  This is not explicitly reported in the document.  7 Have ethical issues been taken into consideration? No  Ethical considerations not reported.  8 Was the data analysis sufficiently rigorous? No  There is a lack of detail on the qualitative analysis.  9 Is there a clear statement of findings? Yes  Conclusion on page 23 is clear. The discussion acknowledges the many challenges for implementation of self-directed support. However is does lack detail regarding discussion for and against the findings, or triangulation etc.  10 How valuable is the research? Moderate  This is gives an early insight into such consumer directed programmes when such data was scarce. In that sense is it quite valuable despite some of the limitations,  Overall score – 5/10 |
| **Holman (1999)**  1 Was there a clear statement of the aims of the research? Yes  2 Is a qualitative methodology appropriate? Yes  3 Was the research design appropriate to address the aims of the research? Yes  4 Was the recruitment strategy appropriate to the aims of the research? Unclear  Not reported  5 Was the data collected in a way that addressed the research issue? Unclear  6 Has the relationship between researcher and participants been adequately considered? Can’t tell  7 Have ethical issues been taken into consideration? Can’t tell  Not reported.  8 Was the data analysis sufficiently rigorous? Can’t tell  9 Is there a clear statement of findings? No.  10 How valuable is the research? Moderate (0.5)  This data was collected at the very early stages of personal budgets in the UK. The report provides very useful insights into implementation issues at the early stage.  Overall score – 3.5/10 |
| **Olmstead (1999)**  1 Was there a clear statement of the aims of the research? No  2 Is a qualitative methodology appropriate? NA  3 Was the research design appropriate to address the aims of the research? Can’t tell  Not discussed  4 Was the recruitment strategy appropriate to the aims of the research? Can’t tell  Not reported  5 Was the data collected in a way that addressed the research issue? Can’t tell  6 Has the relationship between researcher and participants been adequately considered? Can’t tell  This is not discussed in the paper.  7 Have ethical issues been taken into consideration? Can’t tell  Not reported  8 Was the data analysis sufficiently rigorous? Can’t tell  9 Is there a clear statement of findings? No  10 How valuable is the research? Moderate (0.5)  Despite the methodological limitations, this paper presents qualitative insights into the implementation of self-directed programmes in the US at the very early stage of national roll out.  Overall score – 0.5/10 |
| **Blumberg (2000)**  1 Was there a clear statement of the aims of the research? Yes  2 Is a qualitative methodology appropriate? Yes  3 Was the research design appropriate to address the aims of the research? Can’t tell  The design is not discussed in much detail other than to say the individual’s story was used as an informal case study.  4 Was the recruitment strategy appropriate to the aims of the research? Can’t tell  No information given on recruitment strategy.  5 Was the data collected in a way that addressed the research issue? Can’t tell  Method is not discussed in any detail  6 Has the relationship between researcher and participants been adequately considered? No  Two of the authors are the individuals parents but due consideration is not given to any potential bias  7 Have ethical issues been taken into consideration? No  8 Was the data analysis sufficiently rigorous? No  9 Is there a clear statement of findings? No  10 How valuable is the research? Low  This is one individual case and the robustness of the study design and methods are questionable, having not been reported in any detail. Having said that, it does give a unique perspective, written by the family members themselves.  Overall score - 2/10 |
| **Dawson (2000)**  1 Was there a clear statement of the aims of the research? Yes  2 Is a qualitative methodology appropriate? No (0.5)  A mixed methods approach was taken. The aims were no specifically to gain an in-depth insight into the pilots, however it is clear that qualitative findings were critical to the monitoring and reporting of findings.  3 Was the research design appropriate to address the aims of the research? Yes 0.5  While the mixed methods design worked well in terms of monitoring the pilots, the rationale is not clearly outlined.  4 Was the recruitment strategy appropriate to the aims of the research? Can’t tell  5 Was the data collected in a way that addressed the research issue? Yes (0.5)  The detail on data collection is minimal.  6 Has the relationship between researcher and participants been adequately considered? Yes 0.5  A project advisory group was set up included disabled people, but it is unclear how much they contributed to the design of research.  7 Have ethical issues been taken into consideration? Can’t tell  8 Was the data analysis sufficiently rigorous? Can’t tell  The detail on data analysis is not reported  9 Is there a clear statement of findings? Yes 0.5  Yes but the findings are not discussed in relation to international evidence  10 How valuable is the research? Moderate (0.5)  Overall score – 4/10 |
| **Glendinning (2000)**  1 Was there a clear statement of the aims of the research? Yes  There was a clear statement of aims listed in the linked study report (Glendinning 2000)  2 Is a qualitative methodology appropriate? Yes  3 Was the research design appropriate to address the aims of the research? Yes  The design was guided by an export advisory group including disabled researchers. Mixed methods were used to triangulate findings but only the qualitative findings are captured in this review.  4 Was the recruitment strategy appropriate to the aims of the research? Yes  5 Was the data collected in a way that addressed the research issue? Yes  The appendix of Glendinning 2000 is dedicated to providing methodological detail.  6 Has the relationship between researcher and participants been adequately considered? Yes 0.5  This is not explicitly reported in the document but participants were given the opportunity to review findings before publication.  7 Have ethical issues been taken into consideration? No  Ethical considerations not reported.  8 Was the data analysis sufficiently rigorous? No  There is a lack of detail on the qualitative analysis.  9 Is there a clear statement of findings? Yes  10 How valuable is the research? Moderate 0.5  This is gives an early insight into such direct payment schemes when such data was scarce.  Overall score – 7/10 |
| **Leece (2000)**  1 Was there a clear statement of the aims of the research? No  2 Is a qualitative methodology appropriate? Yes  While the aims are not explicitly listed, it is clear that the type of data sought in qualitative in nature, requiring in-depth and insightful responses.  3 Was the research design appropriate to address the aims of the research? No  A self completed postal survey is not the best method of gathering the type of data required,  4 Was the recruitment strategy appropriate to the aims of the research? No  Recruitment strategy is unclear. We do not know if all pilot participants were sent survey.  5 Was the data collected in a way that addressed the research issue? Yes 0.5  While method of data collection was not ideal, all participants who were initially sent a questionnaire were provided with the opportunity to comment on the draft findings, this allowed for an increased response rate and more in-depth qualitative findings.  6 Has the relationship between researcher and participants been adequately considered? No  This is not explicitly reported in the document.  7 Have ethical issues been taken into consideration? No  Ethical considerations not reported.  8 Was the data analysis sufficiently rigorous? No  There is a lack of detail on the qualitative analysis.  9 Is there a clear statement of findings? Yes  Conclusions are well laid out with recommendations explicitly outlined.  10 How valuable is the research? Moderate  This is gives an early insight into an early pilot of direct payments when such data was scarce. In that sense is it quite valuable despite some of the limitations.  Overall score – 3/10 |
| **Pearson (2000)**  1 Was there a clear statement of the aims of the research? No  It was difficult at first to clearly outline what the author aimed to achieve.  2 Is a qualitative methodology appropriate? Yes  3 Was the research design appropriate to address the aims of the research? Yes  Although several study populations were used to outline the policy and practice landscape, service users were used to inform the lived experience of using direct payments.  4 Was the recruitment strategy appropriate to the aims of the research? Yes  The author attempted to get a representative sample by reportedly giving clear directions to the gate keepers.  5 Was the data collected in a way that addressed the research issue? Yes  6 Has the relationship between researcher and participants been adequately considered? No  This is not explicitly reported in the document.  7 Have ethical issues been taken into consideration? No  Ethical considerations not reported.  8 Was the data analysis sufficiently rigorous? Yes  There is detail provided in terms of software used, theoretical framework, and the findings are dispersed with researcher reflexivity.  9 Is there a clear statement of findings? Yes  Conclusions are well laid out with recommendations explicitly outlined.  10 How valuable is the research? Moderate  While the paper is ambitious in its scope resulting in a somewhat disjointed paper, it does provide insightful findings into user experiences of using direct payments under different governing structures.  Overall score – 6.5/10 |
| **Witcher (2000)**  1 Was there a clear statement of the aims of the research? Yes  2 Is a qualitative methodology appropriate? Yes  3 Was the research design appropriate to address the aims of the research? Yes  Given the variation across local authorities, the level of confusion on the ground regarding implementation of policy and the complexity of the intervention, the qualitative approach was most appropriate.  4 Was the recruitment strategy appropriate to the aims of the research? Yes  The researchers worked closely with the LAs in order to access the sample. The strategy was generally successful.  5 Was the data collected in a way that addressed the research issue? Yes  6 Has the relationship between researcher and participants been adequately considered? Yes  Disabled researchers were employed and workshops to illicit feedback on the draft findings.  7 Have ethical issues been taken into consideration? No  Ethical considerations not reported.  8 Was the data analysis sufficiently rigorous? Yes 0.5  While details were provided on the general approach, it lacked detail.  9 Is there a clear statement of findings? Yes  Findings were summarised at the end of each section, with chapter 7 dedicated to implications for policy and practice.  10 How valuable is the research? Moderate 0.5  This paper was successful in (as far as possible) deciphering a very complex set of arrangements across Scotland. This paper would certainly have shed light on an otherwise confused landscape. It was also timely.  Overall score – 8/10 |
| **Smith (2001)**  1 Was there a clear statement of the aims of the research? Yes  2 Is a qualitative methodology appropriate? Yes  3 Was the research design appropriate to address the aims of the research? Yes  A case study approach was chosen to highlight exemplar states implementing person centred approaches.  4 Was the recruitment strategy appropriate to the aims of the research? Can’t tell  5 Was the data collected in a way that addressed the research issue? Can’t tell  Very little detail provided  6 Has the relationship between researcher and participants been adequately considered? Can’t tell  This is not discussed in the paper  7 Have ethical issues been taken into consideration? Can’t tell  Not reported.  8 Was the data analysis sufficiently rigorous? Not reported  9 Is there a clear statement of findings? No  10 How valuable is the research? Low (0)  There is not enough methodological information to assess the robustness of this study. Also the findings are not presented in a very accessible manner, nor are the findings discussed in relation to international evidence.  Overall score – 3/10 |
| **Carmichael (2002)**  1 Was there a clear statement of the aims of the research? Yes  2 Is a qualitative methodology appropriate? Yes  It was a mixed methods review but multiple qualitative methods were adopted which were appropriate  3 Was the research design appropriate to address the aims of the research? Yes  4 Was the recruitment strategy appropriate to the aims of the research? Yes  5 Was the data collected in a way that addressed the research issue? No. Can’t tell  6 Has the relationship between researcher and participants been adequately considered? Yes  As user-led action research, the whole design from beginning through to send actively sought to consider the relationship between researcher and participant.  7 Have ethical issues been taken into consideration? Can’t tell  8 Was the data analysis sufficiently rigorous? Can’t tell  There is no information provided on the analysis of qualitative data.  9 Is there a clear statement of findings? Yes  10 How valuable is the research? (Moderate) (0.5)  Overall score – 6.5/10 |
| **Conroy (2002)**  1. Did the study address a clearly focused issue? Yes  2. Was the cohort recruited in an acceptable way? Yes  3. Was the exposure accurately measured to minimise bias? Yes  4. Was the outcome accurately measured to minimise bias? Yes  Blinding of assessors is not possible due to the nature of the research; it is clear who is in the intervention group and who is not.  5. (a) Have the authors identified all important confounding factors? List the ones you think might be important, that the author missed. No.  (b) Have they taken account of the confounding factors in the design and/or analysis? No  There is a general lack of detail on the analysis of quantitative data.  6. (a) Was the follow up of subjects complete enough? Yes  (b) Was the follow up of subjects long enough? Yes  7. What are the results of this study?  The evidence supports a policy to move the self-determination initiative the next level, beyond a small set of “pilot projects,” and toward larger scale system efforts.  8. How precise are the results? Can’t tell  Confidence intervals not reported.  9. Do you believe the results?  Yes.  10. Can the results be applied to the local population?  Yes  11. Do the results of this study fit with other available evidence?  Yes  12. What are the implications of this study for practice?  The study adds to the growing evidence at the time to provide a self-determination option for all.  **6/10** |
| **Eckert (2002)**  1 Was there a clear statement of the aims of the research? Yes  2 Is a qualitative methodology appropriate? Yes  This qualitative data supplements extensive quantitative data collected throughout the Cash and Counseling demonstration sites.  3 Was the research design appropriate to address the aims of the research? Yes  4 Was the recruitment strategy appropriate to the aims of the research? Yes  Yes, it appears that all people meeting the eligibility criteria were invited to participate.  5 Was the data collected in a way that addressed the research issue? Yes  Yes, the data collection process was described in detail. Proxy respondents were used where appropriate. Data was triangulated by getting the perspectives of three stakeholders directly involved in process.  6 Has the relationship between researcher and participants been adequately considered? Can’t tell  This is not discussed in the paper.  7 Have ethical issues been taken into consideration? Can’t tell  Not reported  8 Was the data analysis sufficiently rigorous? Yes  The use of stories is carefully described within the methods, giving a transparent overview of how the data was used and presented. Raw data is extensively reported within the paper.  9 Is there a clear statement of findings? No  Findings are summarised and crosscutting themes discussed, although the implications of the research and recommendations are not clearly outlined.  10 How valuable is the research? Moderate (0.5)  There is a lot of raw data presented here which provides rich data for meta synthesis. This project was also repeated in a number of other demonstration sites.  Overall score – 6.5/10 |
| **Young (2003)**  1 Was there a clear statement of the aims of the research? No  2 Is a qualitative methodology appropriate? Yes  3 Was the research design appropriate to address the aims of the research? Yes  A number of the variables of interest were complex and could best be understood in the context where they occurred. Some potential participants did not have the ability to respond to pencil-and-paper surveys effectively and accurately.  4 Was the recruitment strategy appropriate to the aims of the research? Yes  Yes, the researchers purposely selected participants to get a diverse sample by region, type of care needs and intensity of SDC.  5 Was the data collected in a way that addressed the research issue? Yes  6 Has the relationship between researcher and participants been adequately considered? Yes  The researchers engaged with a working group with representatives from several community based agencies and disability organisations to guide the design  7 Have ethical issues been taken into consideration? Yes  8 Was the data analysis sufficiently rigorous? Yes 0.5  While details were provided on the general approach, it lacked detail.  9 Is there a clear statement of findings? Yes  10 How valuable is the research? Moderate 0.5  This study seemed to have been conducted in a robust manner, with a sizeable sample for the qualitative element, using various stakeholder perspectives to triangulate the findings. An expert group also guided the design and commented on preliminary findings.  Overall score – 8/10 |
| **Breda (2004)**  1 Was there a clear statement of the aims of the research? Yes  The overall aim was quite general, but there was considerable detail given around the research questions, the importance of conducting the research and the insight to be gained by undertaking mixed methods  2 Is a qualitative methodology appropriate? Yes  Yes, there was detailed consideration given to how case studies, with multiple stakeholders would add context to the quant findings and provide a richer insight generally.  3 Was the research design appropriate to address the aims of the research?  Yes  4 Was the recruitment strategy appropriate to the aims of the research?  Yes, the sample was recruited from within the larger study sample, but consideration given to accessing a diverse sample across a number of criteria.  5 Was the data collected in a way that addressed the research issue?  Yes  6 Has the relationship between researcher and participants been adequately considered?  Can’t tell  7 Have ethical issues been taken into consideration?  Can’t tell  8 Was the data analysis sufficiently rigorous?  Yes  9 Is there a clear statement of findings?  Yes and implications for policy outlined clearly.  10 How valuable is the research? Moderate 0.5  The research report provides a lot of raw data in the form of direct quotes which give rich insights into the experience of people from Belgium, a jurisdiction not widely reported on.  Overall score – 7.5/10 |
| **Jordan (2004)**  1 Was there a clear statement of the aims of the research? Yes  2 Is a qualitative methodology appropriate? NA  Quant methods used although the majority of findings presented were of a qualitative nature.  3 Was the research design appropriate to address the aims of the research? No  A more qualitative approach from the outset would have been more appropriate, and gained more in-depth responses.  4 Was the recruitment strategy appropriate to the aims of the research? Yes 0.5  All target sample was offered opportunity to respond. However there is little detail on how population was contacted, reminded etc.  5 Was the data collected in a way that addressed the research issue? Can’t tell  There was little or no data provided on data collection.  6 Has the relationship between researcher and participants been adequately considered? Can’t tell  This is not discussed in the paper.  7 Have ethical issues been taken into consideration? Can’t tell  Not reported  8 Was the data analysis sufficiently rigorous? Can’t tell  There is a lack of detail on analysis of data.  9 Is there a clear statement of findings? No  The findings, implications and context within wider evidence base are not discussed  10 How valuable is the research? Low  There is a lot of raw data presented here which provides rich data for meta synthesis. This project was also repeated in a number of other demonstration sites.  Overall score – 1.5/10 |
| **Stainton (2004)**  1 Was there a clear statement of the aims of the research? Yes  2 Is a qualitative methodology appropriate? Yes  A relatively new, dynamic and changing landscape with a relatively small sample of respondents. Qualitative in-depth study was  3 Was the research design appropriate to address the aims of the research? Yes  4 Was the recruitment strategy appropriate to the aims of the research? Yes  Yes, over 50% of the eligible population was interviewed.  5 Was the data collected in a way that addressed the research issue? Yes  6 Has the relationship between researcher and participants been adequately considered?  Cannot tell  7 Have ethical issues been taken into consideration? Cannot tell  8 Was the data analysis sufficiently rigorous? Yes  9 Is there a clear statement of findings? Yes  10 How valuable is the research? Moderate 0.5  This study seemed to have been conducted in a robust manner, with a sizeable sample for the qualitative element. Given the study was conducted within two years of the introduction of personal budgets, these findings were very insightful at the time, particularly in Wales and the UK.  Overall score – 7.5/10 |
| **Emslie (2005)**  1 Was there a clear statement of the aims of the research? Yes  2 Is a qualitative methodology appropriate? Yes  3 Was the research design appropriate to address the aims of the research? Yes  4 Was the recruitment strategy appropriate to the aims of the research? Yes 0.5  For the end use participant pool: while there is some indication as to how participants were recruited, there is a lack of detail to determine whether the group selected were representative.  The level of detail on other stakeholders contributing to the evaluation is quite detailed and thorough.  5 Was the data collected in a way that addressed the research issue? Yes  Yes, the data collection process was described in detail. Proxy respondents were used where appropriate. Data was triangulated by getting the perspectives of various stakeholders.  6 Has the relationship between researcher and participants been adequately considered? Can’t tell  This is not discussed in the paper.  7 Have ethical issues been taken into consideration? Yes  8 Was the data analysis sufficiently rigorous? Can’t tell  While much of the points discussed are backed up with relevant quotes, there is a lack of detail generally on the analysis.  9 Is there a clear statement of findings? Yes  Key findings are summarised after each section with a more detailed discussion towards the end of the report.  10 How valuable is the research? Moderate (0.5)  This is a very useful report, specifically for the level of detail provided on implementation. The report is split between process and implementation information and participant experience, which allows for a thorough and full picture for the reader.  Overall score – 7/10 |
| **Rosenberg (2005)**  1 Was there a clear statement of the aims of the research? Yes  2 Is a qualitative methodology appropriate? Yes  3 Was the research design appropriate to address the aims of the research? Can’t tell  4 Was the recruitment strategy appropriate to the aims of the research? Yes  Appears to be purposeful.  5 Was the data collected in a way that addressed the research issue? Yes  6 Has the relationship between researcher and participants been adequately considered? Can’t tell  7 Have ethical issues been taken into consideration? Can’t tell  A protocol was reviewed by Review Board of University of Wisconsin-Madison, but it is unclear if this was an ethical application or ethics review board.  8 Was the data analysis sufficiently rigorous? Yes 0.5  Details on analysis are provided but not enough to determine sufficient robustness.  9 Is there a clear statement of findings? Yes 0.5  Findings could have been tied in with international evidence, however clear recommendations are made.  10 How valuable is the research? Moderate (0.5)  This data was collected at the very early stages of personal budgets in the UK. The report provides very useful insights into implementation issues at the early stage.  Overall score – 5.5/10 |
| **Butler (2006)**  1 Was there a clear statement of the aims of the research? Yes  2 Is a qualitative methodology appropriate? Yes  3 Was the research design appropriate to address the aims of the research? Yes  4 Was the recruitment strategy appropriate to the aims of the research? Yes  5 Was the data collected in a way that addressed the research issue? Yes  6 Has the relationship between researcher and participants been adequately considered? Yes  7 Have ethical issues been taken into consideration? No  However ethics was carefully considered and reported  8 Was the data analysis sufficiently rigorous? Yes  9 Is there a clear statement of findings? Yes  10 How valuable is the research? Moderate (0.5)  Overall score – 8.5/10 |
| **Sanderson (2006)**  1 Was there a clear statement of the aims of the research? Yes  2 Is a qualitative methodology appropriate? Yes 0.5  The rationale for including qualitative methods was not detailed in the paper. However it is clear that the results are strengthened with rich qualitative data.  3 Was the research design appropriate to address the aims of the research? Yes 0.5  4 Was the recruitment strategy appropriate to the aims of the research? Yes  5 Was the data collected in a way that addressed the research issue? Yes 0.5  There is a lack of detail provided on the method of data collection. There is no detail on setting, justification for methods used, no indication of interview schedule used (for qualitative), although the key areas covered in quantitative is specified which clearly influenced qualitative data collection also.  6 Has the relationship between researcher and participants been adequately considered? Can’t tell  This is not discussed in the paper.  7 Have ethical issues been taken into consideration? Can’t tell  Not reported  8 Was the data analysis sufficiently rigorous? Can’t tell  There is a lack of detail on analysis conducted.  9 Is there a clear statement of findings? Yes 0.5  Findings are summarised, however implications of research in policy and practice are limited.  10 How valuable is the research? Moderate (0.5)  Overall score – 4.5/10 |
| **Speed (2006)**  1 Was there a clear statement of the aims of the research? Yes  2 Is a qualitative methodology appropriate? Yes  This is a biographical piece. There is no real alternative to presenting the data.  3 Was the research design appropriate to address the aims of the research? Yes  4 Was the recruitment strategy appropriate to the aims of the research? Yes  5 Was the data collected in a way that addressed the research issue? Yes  6 Has the relationship between researcher and participants been adequately considered? Yes  The author acknowledges the inherent biases and the non-generalisability of findings.  7 Have ethical issues been taken into consideration? No  Not reported  8 Was the data analysis sufficiently rigorous? No  There was no data analysis as such.  9 Is there a clear statement of findings? No  10 How valuable is the research? Moderate (0.5)  This is a unique study in that the author has ten years first-hand experience. While it is only a sample of one, the insight and detail provided is considerable. Also, the author has made recommendations for both policy and practice.  Overall score – 6.5/10 |
| **Alakeson (2007)**  1 Was there a clear statement of the aims of the research? No  2 Is a qualitative methodology appropriate? Yes 0.5  Yes it is but the authors do not highlight the rationale for including qualitative findings.  3 Was the research design appropriate to address the aims of the research? Can’t tell  There is no detail provided on the justification for qualitative methods.  4 Was the recruitment strategy appropriate to the aims of the research? Can’t tell  There is very little detail on the recruitment strategy utilised for consumers.  5 Was the data collected in a way that addressed the research issue? Can’t tell  We do not know how participants were selected or if they are representative of wider population.  6 Has the relationship between researcher and participants been adequately considered? Can’t tell  This is not discussed in the paper.  7 Have ethical issues been taken into consideration? Can’t tell  Not reported  8 Was the data analysis sufficiently rigorous? Can’t tell  There is very little detail on analysis used.  9 Is there a clear statement of findings? yes  The findings are discussed in terms of considerations for the future.  10 How valuable is the research? Moderate (0.5)  It provides the perspective of mental health users who are under-represented in the research up until the time of study.  Overall score – 2/10 |
| **Caldwell (2007)**  1 Was there a clear statement of the aims of the research? Yes  2 Is a qualitative methodology appropriate? Yes  The current study provides a voice to individuals with disabilities and their  families to elucidate oppressive forces against consumer direction and the support of  families.  3 Was the research design appropriate to address the aims of the research? Yes  While few qualitative studies have explored consumer-directed programs, such an  approach offers a rich environment in which to explore experiences concerning redistribution  of financial resources  4 Was the recruitment strategy appropriate to the aims of the research? Yes  Purposeful sampling was used from a cohort of people who participated in a previous quantitative evaluation.  5 Was the data collected in a way that addressed the research issue?” Yes  Interviews were conducted in the family home and lasted approximately 1 hour. A semi-structured interview guide was used. Interviews were tape recorded and the tapes were transcribed. Following the interviews, memos were recorded to note observations and make initial reflections concerning the interview. A mother of an individual with intellectual disabilities co-facilitated three of the interviews. This provided further reflection on and insight into the interviews, initial development of themes and ideas for further exploration in subsequent interviews.  6 Has the relationship between researcher and participants been adequately considered? Can’t tell  Not reported  7 Have ethical issues been taken into consideration? Can’t tell  Not reported  8 Was the data analysis sufficiently rigorous? Yes  A grounded theory approach guided data analysis in the current study (Taylor & Bogdan, 1998).  Transcripts were read and tape recordings were reviewed several times during initial coding. Coding schemes were further modified, combined and split as new data was added. A portion of the data was coded independently by the family co-facilitator to allow for comparison and refinement of the coding. Member checks were conducted with all interview participants, which provided opportunities for them to comment on initial themes. Finally, previously conducted quantitative research allowed for triangulation among different data sources.  9 Is there a clear statement of findings? Yes  Through discussion  10 How valuable is the research? Moderate (0.5)  Overall score – 7.5/10 |
| **Dimitriadis (2007)**  1 Was there a clear statement of the aims of the research? Yes  2 Is a qualitative methodology appropriate? No (0.5)  While one objective i.e. to ‘actively engage people with disability, family and carers in decision making processes’ lends itself well to qualitative research, the outcomes related to measurable differences, i.e. expected increase in independence and autonomy would be better measured with a quantitative approach.  3 Was the research design appropriate to address the aims of the research? Yes  The research design involved an ‘action research’ approach and this was discussed and justified.  4 Was the recruitment strategy appropriate to the aims of the research? Can’t tell  5 Was the data collected in a way that addressed the research issue? Can’t tell (0.5)  The detail on data collection is minimal.  6 Has the relationship between researcher and participants been adequately considered? No  No details of such consideration are reported.  7 Have ethical issues been taken into consideration? Yes  8 Was the data analysis sufficiently rigorous? Can’t tell (0.5)  The detail on data analysis is minimal.  9 Is there a clear statement of findings? Yes  Through conclusions and recommendations  10 How valuable is the research? Moderate (0.5)  Overall score – 6/10 |
| **Adams (2008)**  1 Was there a clear statement of the aims of the research? Yes  2 Is a qualitative methodology appropriate? Yes  3 Was the research design appropriate to address the aims of the research? No  While opened ended questions were included and qualitative outcomes listed as those of interest (e.g. Experience of employers using DPs) the authors specifically stated they sought factual rather than qualitative data. However rich qualitative data were used to contextualise quant findings.  4 Was the recruitment strategy appropriate to the aims of the research? Yes  A lot of detail was provided on the recruitment strategy. A very inclusive strategy was adopted although response rate was low. However the number of face-to-face interviews conducted were considerable.  5 Was the data collected in a way that addressed the research issue? Yes 0.5  A more qualitative approach could have garnered richer data related to experiences of employers. However the open ended questions did produce detailed and rich data.  6 Has the relationship between researcher and participants been adequately considered? Yes 0.5  The researchers were very conscious of who was present in the room during interviews and adapted data collection in line with potential adverse reactions (e.g. For sensitive questions related to PAs, respondents could write their answers rather than verbally respond).  7 Have ethical issues been taken into consideration? Yes 0.5  However there is no evidence of an official ethics process.  8 Was the data analysis sufficiently rigorous? No  There is no information provided on the analysis of qualitative data.  9 Is there a clear statement of findings? Yes (0.5)  The executive summary serves as a statement of findings. A specific section outlines recommendations for future research.  10 How valuable is the research? (Moderate) (0.5)  The quantitative data would have been useful had a control group been included. However, the qualitative data is useful in that it represents a sizeable number of respondents and presents the lived experiences of people employing PAs under direct payment scheme.  Overall score – 5.5/10 |
| **Daly (2008)**  1 Was there a clear statement of the aims of the research? Yes  See table above  2 Is a qualitative methodology appropriate? Yes  3 Was the research design appropriate to address the aims of the research? Yes  4 Was the recruitment strategy appropriate to the aims of the research? Can’t tell  5 Was the data collected in a way that addressed the research issue? Yes  A lot of thought went into data collection, including the active participation of PWD. It was an iterative approach which evolved throughout research project.  6 Has the relationship between researcher and participants been adequately considered? Yes  “In an attempt to address intrinsic power imbalances between researchers and respondents (Law *et al*, 2005), researchers aimed to secure agreement between the service user and the researcher about the information that the service user wished to convey.”  7 Have ethical issues been taken into consideration? Yes  8 Was the data analysis sufficiently rigorous? Yes  “in practice, analysis of emerging themes occurred during each contact between the service user and researcher. Tentative ideas for themes for the individual stories were explored at each meeting, and the service users or relatives were able to tell the researchers whether they needed to be adjusted. … Following the service user-led dissemination event, a more detailed generation of reports was undertaken of each data analysis category.  Content analysis was employed (Denzin & Lincoln, 1998; Huberman & Miles, 2002; Patton,  2002) to enable individual accounts to be analysed for over-arching themes. Analysis included familiarisation and annotation of the data notes, creation”  9 Is there a clear statement of findings? Yes (0.5)  The findings are presented clearly in the Conclusion section but there is a lack of discussion of the evidence both for and against the researcher’s findings.  10 How valuable is the research? (Moderate) (0.5)  While the quality of this study is high, it is unclear how much value this adds to the IBSEN study which already evaluated this cohort of people.  Overall score – 8/10 |
| **Dinora (2008)**  1 Was there a clear statement of the aims of the research? Yes  2 Is a qualitative methodology appropriate? Yes  The open ended questions were included in a larger quantitative study to gather detailed information on the respondent’s experiences.  3 Was the research design appropriate to address the aims of the research? No  A more qualitative approach could have been adopted to gain more in-depth insight for the qualitative element of the study.  4 Was the recruitment strategy appropriate to the aims of the research? Yes  5 Was the data collected in a way that addressed the research issue? Yes  6 Has the relationship between researcher and participants been adequately considered? Yes  The authors carefully considered the impact of having proxy respondents. Steps were taken to encourage self-completion of questionnaires where possible. Data collectors had specific training prior to data collection.  7 Have ethical issues been taken into consideration? Yes  8 Was the data analysis sufficiently rigorous? Yes  Responses to open-ended questions were analysed through content analysis. This method was clearly explained and fit well within the larger quantitative study.  9 Is there a clear statement of findings? Yes  Through conclusions and recommendations  10 How valuable is the research? Low  As qualitative data is concerned, the reported data does not provide a particularly deep understanding of the experiences of respondents.  Overall score – 8/10 |
| **Glendinning (2008)**  1 Was there a clear statement of the aims of the research? Yes  2 Is a qualitative methodology appropriate? Yes  3 Was the research design appropriate to address the aims of the research? Yes  4 Was the recruitment strategy appropriate to the aims of the research? Yes  5 Was the data collected in a way that addressed the research issue? Yes  6 Has the relationship between researcher and participants been adequately considered? Can’t tell  7 Have ethical issues been taken into consideration? Yes  8 Was the data analysis sufficiently rigorous? Yes  9 Is there a clear statement of findings? Yes  A set of recommendations are outlined based on the study findings  10 How valuable is the research? Moderate (0.5)  The sample is small and only half had received PB at time of interview although they had started the assessment and planning stage.  Overall score – 8.5/10 |
| **Homer (2008)**  1 Was there a clear statement of the aims of the research? Yes  2 Is a qualitative methodology appropriate? Yes  The methodology is appropriate for the aims, as outlined.  3 Was the research design appropriate to address the aims of the research? Yes  4 Was the recruitment strategy appropriate to the aims of the research? Yes 0.5  While information on recruitment was provided, it lacked detail to determine if it was representative, or biased in some way.  5 Was the data collected in a way that addressed the research issue? Yes  6 Has the relationship between researcher and participants been adequately considered? No  No details of such consideration are reported.  7 Have ethical issues been taken into consideration? Can’t tell  8 Was the data analysis sufficiently rigorous? Yes 0.5  While the authors had developed a framework / typology with which to analyse the data (presented in Annex 4), this framework was abandoned during project due to concerns with robustness. Details for alternative analysis are not provided, but it is clear that themes emerged within the data.  9 Is there a clear statement of findings? Yes  A set of recommendations are outlined based on the study findings  10 How valuable is the research? Moderate (0.5)  The research provides insight into an under-researched area of the UK and provides detailed data particularly relevant to the systematic review.  Overall score – 6.5/10 |
| **Lord (2008)**  1 Was there a clear statement of the aims of the research? Yes  2 Is a qualitative methodology appropriate? Yes  3 Was the research design appropriate to address the aims of the research? Yes  4 Was the recruitment strategy appropriate to the aims of the research? Yes  A purposeful sampling for qualitative study aimed to capture a diverse and representative group.  5 Was the data collected in a way that addressed the research issue? Can’t tell  There is a lack of detail provided on justification, settings, guide development, recording of data, saturation.  6 Has the relationship between researcher and participants been adequately considered? Can’t tell  There is a lack of detail provided.  7 Have ethical issues been taken into consideration? Yes 0.5  But it is unclear whether an ethical application was supported to an organisational or university ethics committee.  8 Was the data analysis sufficiently rigorous? Yes 0.5  While we know how the qualitative data was analysed, the report lacks detail.  9 Is there a clear statement of findings? Yes  10 How valuable is the research? (Moderate) (0.5)  This study provides insight into an under-researched population, especially considering individualised funding was available in Canada for several decades before other jurisdictions.  Overall score – 6.5/10 |
| **Shaw (2008)**  1 Was there a clear statement of the aims of the research? No  The aims seem to be about the Deafblind information initiative but the results are primarily about experience of deafblind people using DPs. Successes and Challenges.  2 Is a qualitative methodology appropriate? Yes  3 Was the research design appropriate to address the aims of the research? Can’t tell  The research design was not justified in the report.  4 Was the recruitment strategy appropriate to the aims of the research? No  It is unclear how many initial questionnaires were sent out. This was a self-selecting group. A more representative group may have been accessed in another manner. Having said that, the response rate for the subgroup of DP users was quite high. Furthermore efforts were made to include a wide range of respondents through wider consultations.  5 Was the data collected in a way that addressed the research issue? Yes 0.5  A more qualitative approach could have garnered richer data related to experiences of employers. However the open ended questions did produce detailed and rich data.  6 Has the relationship between researcher and participants been adequately considered? Can’t tell  7 Have ethical issues been taken into consideration? Can’t tell  8 Was the data analysis sufficiently rigorous? No  There is no information provided on the analysis of qualitative data.  9 Is there a clear statement of findings? Yes (0.5)  A short conclusion section makes recommendations based on overall study. Respondent recommendations are also reported for the wide DP scheme and for the Sense scheme.  10 How valuable is the research? (Moderate) (0.5)  This is not a very high quality piece of research, however it does access a very under-represented cohort of disabled people and therefore it was deemed prudent to include the findings.  Overall score – 2.5/10 |
| **Coyle (2009)**  1 Was there a clear statement of the aims of the research? Yes  2 Is a qualitative methodology appropriate? Yes  3 Was the research design appropriate to address the aims of the research? Yes  4 Was the recruitment strategy appropriate to the aims of the research? Can’t tell  5 Was the data collected in a way that addressed the research issue?” Yes  6 Has the relationship between researcher and participants been adequately considered? Can’t tell  7 Have ethical issues been taken into consideration? Yes  8 Was the data analysis sufficiently rigorous? Yes  9 Is there a clear statement of findings? No  There is not adequate discussion of findings or triangulation of findings. Discussion lacks support from wider research arena.  10 How valuable is the research? Moderate (0.5)  Overall score – 6.5/10 |
| **Rogers (2009)**  1 Was there a clear statement of the aims of the research? Yes  There were many aims listed at the outset.  2 Is a qualitative methodology appropriate? Yes  3 Was the research design appropriate to address the aims of the research? Yes 0.5  There is very little information provided on the evaluation design process. However a Service User Reference Group (potential service users) did contribute to the design of data collection tools.  4 Was the recruitment strategy appropriate to the aims of the research? Yes  All people participating in pilot were invited to participate.  5 Was the data collected in a way that addressed the research issue? Yes 0.5  In-depth interviews were used for service users and carers but open-ended feedback forms were used for staff members. In-depth interviews would have been more appropriate for both groups.  6 Has the relationship between researcher and participants been adequately considered? Yes  Service User Reference Group (potential service users) did contribute to the design of data collection tools. They also met monthly during the pilots to contribute to the ongoing evaluation.  7 Have ethical issues been taken into consideration? Can’t tell  Not reported  8 Was the data analysis sufficiently rigorous? No  There is no information provided on the analysis of qualitative data.  9 Is there a clear statement of findings? Yes (0.5)  The executive summary serves as a statement of findings. A specific section outlines recommendations for future research.  10 How valuable is the research? (Moderate) (0.5)  While much of this evaluation was guided by the national IBSEN evaluation, the focus here was on mental health users. Therefore this research offers a unique perspective for an under represented cohort.  Overall score – 6/10 |
| **Sayles Wallace (2009)**  1 Was there a clear statement of the aims of the research? Yes  2 Is a qualitative methodology appropriate? Yes  3 Was the research design appropriate to address the aims of the research? Yes  4 Was the recruitment strategy appropriate to the aims of the research? Yes 0.5  As acknowledged by the authors, selecting a small group of ‘Pioneer Families’ may be representative of the most creative but not the general population. However this is acknowledged within the dissertation.  5 Was the data collected in a way that addressed the research issue? Yes  6 Has the relationship between researcher and participants been adequately considered? Can’t tell  Ethical considerations were made, as is standard with such research, but it is unclear if the researcher considered the impact on the relationship between researcher and respondent.  The author acknowledges the inherent biases and the non-generalisability of findings.  7 Have ethical issues been taken into consideration? Yes 0.5  Yes but no official ethics process is reported.  8 Was the data analysis sufficiently rigorous? Yes  9 Is there a clear statement of findings? Yes  10 How valuable is the research? Moderate (0.5)  This study provides an in-depth insight into self-directed support for families of adult children with severe intellectual disabilities. It also involves (mostly) families who work in the sector and therefore would have greater insight into how to make the system work best to meet your needs.  Overall score – 7.5/10 |
| **Eost- Telling (2010)**  1 Was there a clear statement of the aims of the research? Yes  2 Is a qualitative methodology appropriate? Yes  3 Was the research design appropriate to address the aims of the research? Yes  4 Was the recruitment strategy appropriate to the aims of the research? Yes  5 Was the data collected in a way that addressed the research issue? Yes  6 Has the relationship between researcher and participants been adequately considered? Can’t tell  Not reported  7 Have ethical issues been taken into consideration? Can’t tell  Not reported  8 Was the data analysis sufficiently rigorous? No  We know that themes were identified using MAX QDA but there is very little detail provided on the qualitative data analysis.  9 Is there a clear statement of findings? Yes  Findings are clearly summarised at the end of the report, with key recommendations for future focus outlined.  10 How valuable is the research? Moderate (0.5)  This report contains both user experience and implementation considerations from staff and brokers. There is an abundance of raw data available for meta-synthesis.  Overall score –6.5/10 |
| **Kinnaird (2010)**  1 Was there a clear statement of the aims of the research? Yes  2 Is a qualitative methodology appropriate? Yes  3 Was the research design appropriate to address the aims of the research? Yes  4 Was the recruitment strategy appropriate to the aims of the research? Yes  Efforts were made to get a representative group from across the participating local authorities but recruitment relied on one gatekeeper organisation. There was a mix of gender, urban rural and a wide age group from 60s to 80s.  5 Was the data collected in a way that addressed the research issue? Yes  6 Has the relationship between researcher and participants been adequately considered? Can’t tell  This is not discussed in the paper.  7 Have ethical issues been taken into consideration? Yes 0.5  Ethical approval was sought from local authorities for staff participating. It is unclear if ethical approval was sought for the carers participating  8 Was the data analysis sufficiently rigorous? Can’t tell  9 Is there a clear statement of findings? Yes  Yes. A clear statement of findings is made at beginning of chapter 3. Clear recommendations are made in Chapter 5  10 How valuable is the research? Moderate (0.5)  This research offers the under-researched perspective of people with dementia (via proxy carers). Implementation issues are also presented from perspective of staff.  Overall score – 7/10 |
| **Leahy (2010)**  1 Was there a clear statement of the aims of the research? Yes  2 Is a qualitative methodology appropriate? Yes  The qualitative data appears to be the main source of data reported, giving context and depth to the survey findings. In fact data from open-ended survey questions also reported.  3 Was the research design appropriate to address the aims of the research? Yes  4 Was the recruitment strategy appropriate to the aims of the research? Yes  The researchers attempted to get a representative group based on a number of criteria.  5 Was the data collected in a way that addressed the research issue? Yes 0.5  The authors took particular care to be inclusive of people previously excluded from earlier study i.e. those living in supported accommodation. However there is a lack of detail on who provided the data, where it was collected, the age etc. Notes were taken but no indication if audio recordings were taken.  6 Has the relationship between researcher and participants been adequately considered? Can’t tell  The author acknowledges the inherent biases and the non-generalisability of findings but relationship between researcher and respondents not explicitly discussed  7 Have ethical issues been taken into consideration? Can’t tell  Not reported  8 Was the data analysis sufficiently rigorous? No  There is very little information provided on qualitative analysis, other than to say the data was entered into excel.  9 Is there a clear statement of findings? Yes  Conclusions are drawn after each section and at the end of the report. Recommendations are made based on the evaluation both for the scheme in general and for service providers.  10 How valuable is the research? Moderate (0.5)  This is a unique perspective of the Australian Self-Managed Model with rich qualitative data presented throughout.  Overall score – 6/10 |
| **Priestley (2010)**  1 Was there a clear statement of the aims of the research? Yes  2 Is a qualitative methodology appropriate? Yes  3 Was the research design appropriate to address the aims of the research? Yes  “This multi-stakeholder approach allowed us to gain a broad organisational view, to  access individual experience and to triangulate perspectives on local implementation”  4 Was the recruitment strategy appropriate to the aims of the research? Can’t tell  5 Was the data collected in a way that addressed the research issue?” Yes 0.5  While we know how the data was collected there is little detail provided on setting, justification for chosen method, use of topic guides etc.  6 Has the relationship between researcher and participants been adequately considered? Can’t tell  7 Have ethical issues been taken into consideration? Can’t tell  Not reported  8 Was the data analysis sufficiently rigorous? Can’t tell  9 Is there a clear statement of findings? Yes 0.5  While the study findings are discussed in relation to the original research question and in relation to other evidence but there are no clear recommendations drawn.  10 How valuable is the research? Moderate (0.5)  Overall score – 4.5/10 |
| **Vinton (2010)**  1 Was there a clear statement of the aims of the research? Yes  2 Is a qualitative methodology appropriate? Yes  It was used as part of a mixed methods approach.  3 Was the research design appropriate to address the aims of the research? Yes  4 Was the recruitment strategy appropriate to the aims of the research? Yes 0.5  While we know how the respondents were recruited “Program participants  (consumers, caregivers, other representatives, support coordinators, and service providers) were invited to attend by the program administrator.”, there is very little detail as to how many accepted, why some were chosen over others etc.  5 Was the data collected in a way that addressed the research issue?” Yes  6 Has the relationship between researcher and participants been adequately considered? Can’t tell  7 Have ethical issues been taken into consideration? Yes  8 Was the data analysis sufficiently rigorous? Yes  9 Is there a clear statement of findings? Yes  10 How valuable is the research? Moderate (0.5)  Overall score – 8/10 |
| **Williams (2010)**  1 Was there a clear statement of the aims of the research? Yes  2 Is a qualitative methodology appropriate? Yes  3 Was the research design appropriate to address the aims of the research? Yes  4 Was the recruitment strategy appropriate to the aims of the research? Yes  Although the sample was very limited to colleagues working in one London borough  5 Was the data collected in a way that addressed the research issue? Yes  6 Has the relationship between researcher and participants been adequately considered? Can’t tell  7 Have ethical issues been taken into consideration? Can’t tell  8 Was the data analysis sufficiently rigorous? Can’t tell  9 Is there a clear statement of findings? Yes  10 How valuable is the research? Low (0)  This research only partially meets the inclusion criteria; however the respondents do discuss implementation issues which are relevant.  Overall score – 6/10 |
| **Wilson (2010)**  1 Was there a clear statement of the aims of the research? Yes  2 Is a qualitative methodology appropriate? NA  Yes but a qualitative method was not used other than open ended questions and comments sections  3 Was the research design appropriate to address the aims of the research? Yes  There is little detail provided on the rationale for including comments section and open ended questions, although the report does extensively report these findings. A lot of thought went into the research design with a consultation process with service user representatives and a pilot, and feedback sessions incorporated into the design.  4 Was the recruitment strategy appropriate to the aims of the research? Can’t tell  There is very little detail provided other than to state that “The Research, Intelligence and Consultation Team worked closely with the Adult Social Care Team to produce a sample of individuals who held an active personal budget from April 2010 to October 2010.”  5 Was the data collected in a way that addressed the research issue? Yes  The researchers endeavoured to capture the voice of service users and where this was not possible to capture their voices through a representative. Also efforts were made to capture carer perspectives separately to avoid bias.  6 Has the relationship between researcher and participants been adequately considered? Yes  This appears to have been considered as part of the extensive research design and pilot process.  7 Have ethical issues been taken into consideration? Can’t tell  Not reported  8 Was the data analysis sufficiently rigorous? Yes 0.5  Analysis of qual data is not reported although it is evident that comments were divided into themes. All of the raw qual data is presented in Appendix A.  9 Is there a clear statement of findings? Yes (0.5)  While the findings are not summarised nor are they within the context of national or international evidence, the intended use of evaluative data is clearly outlined, including the value of the research findings.  10 How valuable is the research? Low  Given the extensive reviews that have taken place in the UK, this data does not add a considerable contribution.  Overall score – 5/10 |
| **Campbell (2011)**  1 Was there a clear statement of the aims of the research? Yes  2 Is a qualitative methodology appropriate? Yes  To document, and understand, the mechanisms which support disabled people to achieve greater choice and control in their lives - a key outcome of independent living - with a specific focus on people moving on to personal budgets. Quantitative methods supplemented the qualitative data collection.  3 Was the research design appropriate to address the aims of the research? Yes  The transfer of support planning and brokerage from local authority to user-led organisations is intended to increase the choice and control of the disabled people involved. The qualitative research set out to discover whether, and how, this happened in practice.  4 Was the recruitment strategy appropriate to the aims of the research? Yes  Samples were drawn from Local Authority and User Led Organisations to compare experiences and outcomes.  5 Was the data collected in a way that addressed the research issue?” Yes  Data was collected in a number of ways, both qualitatively and quantitatively (where more appropriate) in order to address the research questions fully. Qual - “Data was collected via semi-structured in-depth interviews, with time spent to get to know each person, before working through a topic guide in a way that suited the individual participant. The aim was to provide a context for discussion of issues and reflection, rather than a scheduled list of specific questions”  6 Has the relationship between researcher and participants been adequately considered? Can’t tell  Not reported  7 Have ethical issues been taken into consideration? Yes  Since the qualitative research included participants who lacked capacity to consent to research, approval was gained from a national research ethics committee flagged for proposals relating to mental capacity. Where appropriate, carers and other close individuals were asked to act as consultees in relation to consent matters. 42 participants also had carers acting as supporters in interviews, evenly spread over the LA and ULO groups.   1. Was the data analysis sufficiently rigorous? Yes   All data were analysed using an interpretative methodology grounded in what participants said, so that the themes were both set by the research topic guide, but also reflected what was important in the lives of the eighty people who took part in this study.  A software package (NVivo 8) was used in order both to organise the data, construct a coding scheme for analysis, and review data for particular groups of individuals.  9 Is there a clear statement of findings? Yes  10 How valuable is the research? Moderate (0.5)  This research seems to be well thought through and reported. Various methods of data collection are used to triangulate the findings. Limitations are clearly presented and considered.  Overall score – 8.5/10 |
| **Hatton (2011)**  1 Was there a clear statement of the aims of the research? Yes  2 Is a qualitative methodology appropriate? NA  This qualitative data was not the primary method of data collections but is used to supplement quantitative data collected  3 Was the research design appropriate to address the aims of the research? Yes  4 Was the recruitment strategy appropriate to the aims of the research? Can’t tell  While we know how many responded we don’t know the population.  5 Was the data collected in a way that addressed the research issue? No  In terms of qualitative outcomes, i.e. views and experiences of servicer users and carers, a more qualitative approach would have been more suitable.  6 Has the relationship between researcher and participants been adequately considered? Can’t tell  This is not discussed in the paper.  7 Have ethical issues been taken into consideration? No  8 Was the data analysis sufficiently rigorous? Can’t tell  Qualitative data analysis is not discussed.  9 Is there a clear statement of findings? No  Findings are summarised but they are not discussed in terms of other international evidence.  10 How valuable is the research? Moderate (0.5)  While the qualitative data is limited, the sample is quite large and therefore a broad spectrum of views have been captured.  Overall score – 2.5/10 |
| **Lambert (2011)**  1 Was there a clear statement of the aims of the research? Yes  2 Is a qualitative methodology appropriate? Yes  3 Was the research design appropriate to address the aims of the research? Yes  4 Was the recruitment strategy appropriate to the aims of the research? Yes  5 Was the data collected in a way that addressed the research issue? Yes  6 Has the relationship between researcher and participants been adequately considered? Can’t tell  This is not discussed in the paper.  7 Have ethical issues been taken into consideration? Can’t tell  Not reported  8 Was the data analysis sufficiently rigorous? Yes  9 Is there a clear statement of findings? Yes 0.5  A statement of findings is outlined at the beginning of the report. Conclusions are drawn at the end of the report, however the findings are not discussed in relation to international evidence.  10 How valuable is the research? Moderate (0.5)  There is a lot of raw data presented here which provides rich data for meta synthesis. It is also useful to compare different types of personal budgets models and how these affect the perceived improvements.  Overall score – 7/10 |
| **Newbronner (2011)**  1 Was there a clear statement of the aims of the research? Yes  2 Is a qualitative methodology appropriate? Yes  3 Was the research design appropriate to address the aims of the research? Yes  4 Was the recruitment strategy appropriate to the aims of the research? Yes  5 Was the data collected in a way that addressed the research issue?” Yes  6 Has the relationship between researcher and participants been adequately considered? Yes  7 Have ethical issues been taken into consideration? Yes  8 Was the data analysis sufficiently rigorous? Yes  9 Is there a clear statement of findings? Yes 0.5  Executive summary clearly outlines the findings however they are not discussed in relation to international evidence / policy  10 How valuable is the research? Moderate (0.5)  Overall score – 9/10 |
| **Ridley (2011)**  1 Was there a clear statement of the aims of the research? Yes  2 Is a qualitative methodology appropriate? Yes  3 Was the research design appropriate to address the aims of the research? Yes  4 Was the recruitment strategy appropriate to the aims of the research? Yes 0.5  While there is some detail provided on the recruitment of 30 cases, and efforts to get a representative sample across various criteria, it is not reported how they went about this.  5 Was the data collected in a way that addressed the research issue? Yes  6 Has the relationship between researcher and participants been adequately considered? Can’t tell  This is not discussed in the paper.  7 Have ethical issues been taken into consideration? Can’t tell  Not reported  8 Was the data analysis sufficiently rigorous? Yes 0.5  While there is some information provided on analysis, it is not thorough enough to determine robustness.  9 Is there a clear statement of findings? Yes  Chapter 6 outlines the key lessons learned and makes 24 recommendations based on these. Recommendations are broken down into various themes, reflective of the findings.  10 How valuable is the research? Moderate (0.5)  Overall score – 6.5/10 |
| **Secker (2011)**  1 Was there a clear statement of the aims of the research? Yes  2 Is a qualitative methodology appropriate? Yes  3 Was the research design appropriate to address the aims of the research? Can’t tell  No justification given to research design.  4 Was the recruitment strategy appropriate to the aims of the research? Can’t tell  5 Was the data collected in a way that addressed the research issue?” Can’t tell  6 Has the relationship between researcher and participants been adequately considered? Can’t tell  7 Have ethical issues been taken into consideration? Can’t tell  Not reported  8 Was the data analysis sufficiently rigorous? Can’t tell  Information on data analysis is not provided  9 Is there a clear statement of findings? Yes 0.5  10 How valuable is the research? Low  There is very little methodological information provided.  Overall score – 2.5/10 |
| **Rummery (2012)**  1 Was there a clear statement of the aims of the research? Yes  2 Is a qualitative methodology appropriate? NA  This qualitative data was not the primary method of data collections but is used to supplement quantitative data collected  3 Was the research design appropriate to address the aims of the research? Yes  4 Was the recruitment strategy appropriate to the aims of the research? Yes  5 Was the data collected in a way that addressed the research issue? No  In terms of qualitative outcomes, i.e. views and experiences of servicer users and carers, a more qualitative approach would have been more suitable.  6 Has the relationship between researcher and participants been adequately considered? Can’t tell  This is not discussed in the paper.  7 Have ethical issues been taken into consideration? Yes  8 Was the data analysis sufficiently rigorous? Can’t tell  Qualitative data analysis is not discussed.  9 Is there a clear statement of findings? Yes  Chapter 7 provides detailed discussion of findings in relation to policy and practice along with issues for consideration by various stakeholders  10 How valuable is the research? Low  The aims of this particular report are not consistent with aims of systematic review. Furthermore, the majority of data presented is not relevant to the systematic review. However, the qualitative data of service users was deemed appropriate to include.  Overall score – 5/10 |
| **Sheikh (2012)**  1 Was there a clear statement of the aims of the research? Yes  2 Is a qualitative methodology appropriate? Yes  3 Was the research design appropriate to address the aims of the research? Yes  4 Was the recruitment strategy appropriate to the aims of the research? Yes 0.5  It is not clear how the service users were recruited for rounds one and two.  5 Was the data collected in a way that addressed the research issue? Yes  6 Has the relationship between researcher and participants been adequately considered? Can’t tell  This is not discussed in the paper.  7 Have ethical issues been taken into consideration? Can’t tell  Not reported  8 Was the data analysis sufficiently rigorous? Yes  A specific framework was developed for which to analyse the qualitative data.  9 Is there a clear statement of findings? Yes  Chapters 8 and 9 are dedicated to summarising the findings and putting recommendations forward for various processes and stakeholders.  10 How valuable is the research? Moderate (0.5)  There is a lot of raw data presented here which provides rich data for meta synthesis. Furthermore the data was gathered over a long period of time, allowing for capture of changes over time.  Overall score – 7/10 |
| **Gross (2013)**  1 Was there a clear statement of the aims of the research? Yes  2 Is a qualitative methodology appropriate? Yes  Rationale was presented  3 Was the research design appropriate to address the aims of the research? Yes  Clearly outlined in detail  4 Was the recruitment strategy appropriate to the aims of the research? Yes  5 Was the data collected in a way that addressed the research issue? Yes  While the data is expected to be representative, the expectations of the data are well described, as are the limitations.  6 Has the relationship between researcher and participants been adequately considered? Can’t tell  This is not discussed in the paper however, it is clear that the researchers gave a lot of thought in terms of design and how best to represent the four cases who could not participate themselves  7 Have ethical issues been taken into consideration? Can’t tell  Not reported. Consent was sought from all participants  8 Was the data analysis sufficiently rigorous? Yes  Very detailed section on analysis. (page 92)  9 Is there a clear statement of findings? Yes  Findings were summarised including implications for the research, policy and practice.  10 How valuable is the research? Moderate (0.5)  Overall score – 7.5/10 |
| **Hatton (2013)**  1 Was there a clear statement of the aims of the research? Yes  2 Is a qualitative methodology appropriate? NA  This qualitative data was not the primary method of data collections but is used to supplement quantitative data collected  3 Was the research design appropriate to address the aims of the research? Yes  4 Was the recruitment strategy appropriate to the aims of the research? Can’t tell  While we know how many responded we don’t know the population.  5 Was the data collected in a way that addressed the research issue? No  In terms of qualitative outcomes, i.e. views and experiences of servicer users and carers, a more qualitative approach would have been more suitable.  6 Has the relationship between researcher and participants been adequately considered? Can’t tell  This is not discussed in the paper.  7 Have ethical issues been taken into consideration? No  8 Was the data analysis sufficiently rigorous? Can’t tell  Qualitative data analysis is not discussed.  9 Is there a clear statement of findings? No  Findings are summarised but they are not discussed in terms of other international evidence.  10 How valuable is the research? Moderate (0.5)  While the qualitative data is limited, the sample is quite large and therefore a broad spectrum of views has been captured.  Overall score – 2.5/10 |
| **Rees (2013)**  1 Was there a clear statement of the aims of the research? Yes  2 Is a qualitative methodology appropriate? Yes  3 Was the research design appropriate to address the aims of the research? Yes  4 Was the recruitment strategy appropriate to the aims of the research? Yes  Rationale for sampling was clearly outlined  5 Was the data collected in a way that addressed the research issue? Yes  6 Has the relationship between researcher and participants been adequately considered? Can’t tell  This is not discussed in the paper.  7 Have ethical issues been taken into consideration? Yes 0.5  Ethical considerations are outlined clearly, including how they were addressed. However, an official ethical application process was not followed.  8 Was the data analysis sufficiently rigorous? Can’t tell  There is a lack of detail on analysis of data.  9 Is there a clear statement of findings? Yes  Findings are summarised and discussed; the implications for policy and practice outlined.  10 How valuable is the research? Moderate (0.5)  There is a lot of raw data presented here which provides rich data for meta synthesis. This project was also repeated in a number of other demonstration sites.  Overall score – 7/10 |
| **Bola (2014)**  1 Was there a clear statement of the aims of the research? Yes  See table above  2 Is a qualitative methodology appropriate? Yes  3 Was the research design appropriate to address the aims of the research? Can’t tell  The authors do not give any justification for the chosen design. We do not know if they considered another approach.  4 Was the recruitment strategy appropriate to the aims of the research? Yes  While there was an informal strategy utilised i.e. opportunistic and snowballing, the opportunistic sampling was done by contacting experts in the field, within the research steering committee. While selection bias is a real possibility, it appears the researchers did attempt to select an appropriate sample.  5 Was the data collected in a way that addressed the research issue? Yes / 0.5  Positives: Details provided in terms of forms of data collection i.e. interviews and focus groups. The methods chosen however or the setting for data collection was not justified. Lines of enquiry / topic guide were included. The methods were not modified during study. Interviews and focus groups were audio recorded. Saturation point was not discussed. Overall, I would give a tentative Yes to this question (0.5 out of 1).  6 Has the relationship between researcher and participants been adequately considered? No  No detail of such consideration is reported.  7 Have ethical issues been taken into consideration? Yes  8 Was the data analysis sufficiently rigorous? No  The description of analysis is very brief. There is room for improvement i.e. explaining how certain quotes were selected over others, critically examining the role of the researcher in the analysis and selection of data.  9 Is there a clear statement of findings? Yes  Discussion of findings and study recommendations included.  10 How valuable is the research? Moderate (0.5)  A more systematic study design would be beneficial. Differentiating the results by those in receipt of PB and those not would have been insightful  Overall score – 6/10 |
| **Junne (2014)**  1 Was there a clear statement of the aims of the research? Yes  2 Is a qualitative methodology appropriate? Yes  3 Was the research design appropriate to address the aims of the research? Yes  4 Was the recruitment strategy appropriate to the aims of the research? Can’t tell  While a random sample was indicated, we do not know what the participation rate was. Also for such a small sample, random sampling is unlikely to produce a representative sample. Perhaps purposeful sampling would have been more appropriate.  5 Was the data collected in a way that addressed the research issue? Yes  6 Has the relationship between researcher and participants been adequately considered? Can’t tell  This is not discussed in the paper.  7 Have ethical issues been taken into consideration? Can’t tell  Not reported  8 Was the data analysis sufficiently rigorous? Yes 0.5  It is unclear how themes were distilled. More detail would have been helpful.  9 Is there a clear statement of findings? Yes  F10 How valuable is the research? Moderate (0.5)  This study offers a unique perspective by focussing on risks associated with PBs. While it does not fit the inclusion criteria in the perfect sense, it was deemed appropriate to include.  Overall score – 6/10 |
| **Buchanan (2014)**  1 Was there a clear statement of the aims of the research? Yes  2 Is a qualitative methodology appropriate? Yes  3 Was the research design appropriate to address the aims of the research? Can’t tell  A lot of data had already been collected so it made sense to make use of this data rather than repeating data collection.  4 Was the recruitment strategy appropriate to the aims of the research? Can’t tell  Very little information is provided on recruitment strategy other than to say ‘self-selected’  5 Was the data collected in a way that addressed the research issue? Yes  Sufficient detail is provided for the various ways in which data was collected. “Consumers of the SPS service, who self-selected into the study, consented for researchers to have access to de-identified data of their lived experiences while undertaking SPS services.  Lived experiences reflected consumers’ accounts of what life was like, while living with and beyond mental illness [15]. The data were held by the service provider and an independent evaluator. Overall, the data on 473 documents related to consumers’ lived experiences prior to and while they were undertaking services (Tables 1 and 2). This data had been developed by three sources including the consumers, two Guides and another evaluator independent of the researchers/authors. The data developed by sixteen consumers were held by the service provider. The Guides provided the researchers with consumers’ action plans, two  questionnaires (‘Most Important Changes (MIC) to My Life’ completed one to four times and  ‘Recovery’) that had been completed for the independent evaluator prior to the commencement of this study, and PCP reunion speeches. The Guides also provided the researchers with documents they had developed on their personal learning and reflections of consumers’ progress and service aspects (completed four to seven times per group), meeting minutes, and own PCP reunion speeches. The independent evaluator who had evaluated the  PCP process three times for the service provider gave the researchers interview (of consumers and staff) and report (three key note summaries and full evaluations) data. The data developed by the three sources varied in the type (e.g., background, experiences around  living with mental illness, hopes, and experience of the SPS service) and style of information  that was captured (e.g., bullet point, short answer, Likert scale, or narrative) as described in  Table 1.”  6 Has the relationship between researcher and participants been adequately considered? No  Not applicable as secondary data is used. However, the relationship between the data collectors of original data does not seem to be considered.  7 Have ethical issues been taken into consideration? Yes  Ethical approval was gained and therefore ethical issues would have been considered  8 Was the data analysis sufficiently rigorous? Yes  Sufficient detail is provided on the data analysis process. Limitations are also considered and implications presented.  9 Is there a clear statement of findings? Yes  “Most consumers at varied levels experienced four over-arching categories of positive recovery experiences. This seemed directly related to them having access to individualised funds, high quality shared management and person-centred relationships, and the chance to self-direct. In these SPS service experiences, consumers had improved emotional encounters and control over all aspects of their recovery that seemed to lead to behavioural, cognitive and/or emotional change and growth of quality of life. Thus, these results showed that newly provided SPS services in WA met most consumers’ unique recovery needs across all aspects of their lives in a meaningful, effective and timely manner.”  10 How valuable is the research? Moderate (0.5)  This seems to be high quality research given the limitations discussed. However, the author acknowledges the limited transferability of findings and need for further research.  Overall score – 6.5/10 |
| **Waters (2014)**  1 Was there a clear statement of the aims of the research? Yes  2 Is a qualitative methodology appropriate? NA  This qualitative data was not the primary method of data collections but is used to supplement quantitative data collected  3 Was the research design appropriate to address the aims of the research? Yes  4 Was the recruitment strategy appropriate to the aims of the research? Can’t tell  While we know how many responded we don’t know the population.  5 Was the data collected in a way that addressed the research issue? No  In terms of qualitative outcomes, i.e. views and experiences of servicer users and carers, a more qualitative approach would have been more suitable.  6 Has the relationship between researcher and participants been adequately considered? Can’t tell  This is not discussed in the paper.  7 Have ethical issues been taken into consideration? No  8 Was the data analysis sufficiently rigorous? Can’t tell  Qualitative data analysis is not discussed.  9 Is there a clear statement of findings? No  Findings are summarised but they are not discussed in terms of other international evidence.  10 How valuable is the research? Low  Very little qualitative data is presented in this version of the InControl report. The quantitative data cannot be used either.  Overall score – 2/10 |
| **Coles (2015)**  1 Was there a clear statement of the aims of the research? No.  The overall aim of the paper is not outlined within the main text of the article. The purpose outlined in the accessible summary only partially identifies the aim of the paper. However for the purposes of quality review, the aim will be taken as examining the experiences of parents managing a direct payment.  2 Is a qualitative methodology appropriate? Yes  3 Was the research design appropriate to address the aims of the research? NA  The research design focuses more about ‘insider research’ than on capturing experiences of parents.  4 Was the recruitment strategy appropriate to the aims of the research? Yes  The author acknowledges that the sample is not representative and was a convenience sample, but for the purposes of the paper, this seems appropriate.  5 Was the data collected in a way that addressed the research issue? Yes  6 Has the relationship between researcher and participants been adequately considered? Yes  This is one of the key aims of the paper and is therefore discussed in great detail.  7 Have ethical issues been taken into consideration? Yes  8 Was the data analysis sufficiently rigorous? Yes  9 Is there a clear statement of findings? Yes  Findings are summarised and discussed; the implications for policy and practice considered  10 How valuable is the research? Moderate (0.5)  Although this paper does not set out to address the outcomes of interest in the current systematic review, it does offer a unique perspective of managing personal budgets on behalf of a disabled person.  Overall score – 7.5/10 |
| **Glendinning (2015)**  1 Was there a clear statement of the aims of the research? Yes  2 Is a qualitative methodology appropriate? Yes  3 Was the research design appropriate to address the aims of the research? Yes  4 Was the recruitment strategy appropriate to the aims of the research? Yes  Rationale for sampling was clearly outlined  5 Was the data collected in a way that addressed the research issue? Yes  6 Has the relationship between researcher and participants been adequately considered? Yes  The researchers used talking mats to take into account the potential limitations of participants. Carers and participants were interviewed separately where requested.  7 Have ethical issues been taken into consideration? Yes  8 Was the data analysis sufficiently rigorous? Yes  9 Is there a clear statement of findings? Yes  10 How valuable is the research? Moderate (0.5)  This paper offers a unique perspective of carers and budget users where carers manage the budget on behalf of their disabled relative.  Overall score – 9.5/10 |
| **Hamilton (2015)**  1 Was there a clear statement of the aims of the research? Yes  2 Is a qualitative methodology appropriate? Yes  3 Was the research design appropriate to address the aims of the research? Yes  4 Was the recruitment strategy appropriate to the aims of the research? Yes  5 Was the data collected in a way that addressed the research issue?” Yes  6 Has the relationship between researcher and participants been adequately considered? Yes  7 Have ethical issues been taken into consideration? Yes  8 Was the data analysis sufficiently rigorous? Yes  9 Is there a clear statement of findings? Yes  10 How valuable is the research? Moderate (0.5)  Overall score – 9.5/10 |
| **Jepson (2015)**  1 Was there a clear statement of the aims of the research? Yes  2 Is a qualitative methodology appropriate? Yes  3 Was the research design appropriate to address the aims of the research? Yes  4 Was the recruitment strategy appropriate to the aims of the research? Yes  Rationale for sampling was clearly outlined  5 Was the data collected in a way that addressed the research issue? Yes  6 Has the relationship between researcher and participants been adequately considered? Can’t tell  This is not discussed in the paper.  7 Have ethical issues been taken into consideration? Yes  8 Was the data analysis sufficiently rigorous? Yes  9 Is there a clear statement of findings? Yes  10 How valuable is the research? Moderate (0.5)  This is very unique perspective of a third party receiving payment on behalf of person with dementia who does not have the capacity to manage a direct payment themselves.  Overall score – 8.5/10 |
| **Jones (2015)**  1 Was there a clear statement of the aims of the research? Yes  2 Is a qualitative methodology appropriate? Yes  3 Was the research design appropriate to address the aims of the research? Yes  4 Was the recruitment strategy appropriate to the aims of the research? Yes  5 Was the data collected in a way that addressed the research issue? Yes  6 Has the relationship between researcher and participants been adequately considered? Yes  Researcher with disability involved to inform all stages of research.  7 Have ethical issues been taken into consideration? Can’t tell  8 Was the data analysis sufficiently rigorous? Yes 0.5  There is a lack of detail provided on analysis of qualitative data.  9 Is there a clear statement of findings? Yes  See implications and conclusions  10 How valuable is the research? Moderate (0.5)  Overall score – 8/10 |
| **Laragy (2015)**  1 Was there a clear statement of the aims of the research? Yes  2 Is a qualitative methodology appropriate? Yes  3 Was the research design appropriate to address the aims of the research? Can’t tell  4 Was the recruitment strategy appropriate to the aims of the research? Yes 0.5  The authors based selection on advice from prominent advocates and selected progressive and innovative service providers. Unclear why less progressive service providers were not included.  5 Was the data collected in a way that addressed the research issue?” Yes  6 Has the relationship between researcher and participants been adequately considered? Can’t tell  7 Have ethical issues been taken into consideration? Yes  8 Was the data analysis sufficiently rigorous? Yes  9 Is there a clear statement of findings? Yes  Through discussion and conclusion  10 How valuable is the research? Moderate (0.5)  Overall score – 7/10 |
| **O’Brien (2015)**  1 Was there a clear statement of the aims of the research? Yes  2 Is a qualitative methodology appropriate? Yes  The research was exploratory in nature and therefore qualitative methods were appropriate.  3 Was the research design appropriate to address the aims of the research? Yes  4 Was the recruitment strategy appropriate to the aims of the research? Yes  “Purposeful sampling was the most relevant type of sampling for this research as the research is based specifically on individuals with a disability that are in receipt of direct payments in Ireland.”  5 Was the data collected in a way that addressed the research issue?” Yes  6 Has the relationship between researcher and participants been adequately considered? Can’t tell  7 Have ethical issues been taken into consideration? Can’t tell  Not reported  8 Was the data analysis sufficiently rigorous? Can’t tell  Information on data analysis is not provided  9 Is there a clear statement of findings? Yes  Through recommendations  10 How valuable is the research? Moderate (0.5)  The report lacks the detail required to tell if this was a robust study in terms of analysis, ethics etc. Therefore it cannot be scored any higher than moderate.  Overall score – 6.5/10 |
| **Fleming (2016) Assessed by by independent reviewer (MH)**  1 Was there a clear statement of the aims of the research? Yes  The objectives of the study were to assess whether individualised funding was: perceived to be effective at improving health and social care outcomes in Ireland; acceptable and feasible within the Irish context; and an appropriate mechanism for supporting disabled people to gain independence and self-determined lives, fully integrated within the community.  2 Is a qualitative methodology appropriate? Yes  3 Was the research design appropriate to address the aims of the research? Yes  Exploratory mixed methods utilising a critical realist framework was used as there were small numbers of participants. Additionally, little is known about the processes, structures, successes and challenges and in-depth mixed methods methodology  is most appropriate to meet objectives  4 Was the recruitment strategy appropriate to the aims of the research? Yes  5 Was the data collected in a way that addressed the research issue? Yes  See participants and methods section and Methods section  - audio recorded interviews, document analysis  -Purposive sampling  -Interview schedules developed based on document review  - Table 1 presents those who were recruited as a percentage of those are availing of services.  -Data collection ceased once saturation was reached and no new  themes were emerging  6 Has the relationship between researcher and participants been adequately considered? No  Researcher does not appear to describe the relationship between researcher and participant.  The framework used is described, however the specific relationship is not  7 Have ethical issues been taken into consideration? Yes  Ethical was sought and approved from the appropriate source  Informed consent was sought  Information was regarding the study was provided using an information pack and participants could have an advocate present if desired.  8 Was the data analysis sufficiently rigorous? Yes  Detailed thematic analysis was conducted using a critical realist perspective, as well as an implementation science framework.  All major themes are presented with quotes to illustrate the identification of the theme as well as the source from which the quote came.  Data was analysed using a qualitative analysis package.  Validity of the data was tested by soliciting feedback from key stakeholders using a participatory workshop   1. Is there a clear statement of findings? Yes   The findings are described in detail in the findings section along with illustrative quotes  Findings are also discussed in detail in relation to international research  Credibility of the findings was assessed using a participatory workshop however analysis has not been sub-grouped based on interview findings or workshop findings  Findings are discussed in relation to the original objectives - perceived efficacy, acceptability and applicability was explored   1. How valuable is the research? High (1)   This research is extremely valuable, it utilises rigorous qualitative methodology to provide a detailed assessment of IF in Ireland. It provides pragmatic recommendations and how structures and processes can be improved. In addition to highlighting how important these services are for PwD  Overall score – 9/10 |
| **McGuigan (2016)**  1 Was there a clear statement of the aims of the research? Yes  2 Is a qualitative methodology appropriate? Yes  3 Was the research design appropriate to address the aims of the research? Yes 0.5  While the qualitative element of the study is appropriate and constitutes the majority of findings presented, the methods are primarily presented as a cross-sectional survey. There is a lack of detail for rationale.  4 Was the recruitment strategy appropriate to the aims of the research? No  It is unclear why the authors decided to sample 10% of total population. Also all DP users were invited to participate but only the first 30 that presented themselves were selected. This may not have been a representative sample and may also have been the more proactive participants.  5 Was the data collected in a way that addressed the research issue? Yes  6 Has the relationship between researcher and participants been adequately considered? Can’t tell  7 Have ethical issues been taken into consideration? Can’t tell  8 Was the data analysis sufficiently rigorous? Can’t tell  9 Is there a clear statement of findings? Yes  Findings are summarised and discussed; the implications for social work practice considered  10 How valuable is the research? Moderate (0.5)  Overall score – 5/10 |
