# Supplementary material for: Individualized funding interventions to improve health and social care outcomes for people with a disability: A mixed‐methods systematic review
Source: Campbell Syst Rev. 2019 Jul 19;15(1-2):e1008. doi: 10.4073/csr.2019.3 (PMC8356501; doi:10.4073/csr.2019.3)
Supplement: Supplementary file 8 — Supporting information [file CL2-15-e1008-s008.docx]

# Appendix 8: Changes to full-text screening tool (capturing outcome and methodological detail)

| **Individualised funding interventions to improve health and social care outcomes for people with a disability: a mixed-methods systematic review**  **Study ID: ________ Coder: ________ Date: ____**  **APA Citation:** *(PF to insert)***__________________________________** | |
| --- | --- |
| **Section A: Full Text Eligibility Screening Form** | **Study Name** |

Has a state funded personal budgeting intervention been utilised for a minimum of 6 months?

Yes

Majority (state %)

Unsure

No, then STOP!

Is the study population aged over 18 years of age?

Yes

Majority (state %)

Unsure

No, then STOP!

Does the study population have any form of physical, sensory, intellectual or developmental disability, dementia or mental health problem, disorder or illness?

Yes

Unsure

No, then STOP!

Has a study design been adopted which collected and analysed empirical data, including outcomes of interest?

Yes

Unsure

No, then STOP!

List the outcomes reported:

Is the study:

Quantitative

Qualitative

Mixed methods (including open ended questions in a quantitative study)

If the study design is quantitative (ONLY), it should be EXCLUDED on the basis of: single-case design, pre-post study without a control group, non-matched control groups, or groups matched post-hoc after results were known.

EXCLUDED ON THIS BASIS

Any other comments:
