# Supplementary material for: Individualized funding interventions to improve health and social care outcomes for people with a disability: A mixed‐methods systematic review
Source: Campbell Syst Rev. 2019 Jul 19;15(1-2):e1008. doi: 10.4073/csr.2019.3 (PMC8356501; doi:10.4073/csr.2019.3)
Supplement: Supplementary file 9 — Supporting information [file CL2-15-e1008-s009.docx]

# Appendix 9 – Description of primary, secondary, adverse and other outcomes reported

Contents

[Appendix 9 – Description of primary, secondary, adverse and other outcomes reported 1](#_Toc490050633)

[Table A9.1: Measures of quality of life in each study 2](#_Toc490050634)

[Table A9.2: Measures of client satisfaction in each study 3](#_Toc490050635)

[Table A9.3: Measures of physical functioning in each study 4](#_Toc490050636)

[Table A9.4: Measures of costs data in each study 5](#_Toc490050637)

[Table A9.5: Measures of adverse outcomes in each study 6](#_Toc490050638)

[Table A9.6: Measures of other health and social care outcomes in each study 7](#_Toc490050639)

### Table A9.1: Measures of quality of life in each study

| Study | Outcome measure used | Reliability | Validity |
| --- | --- | --- | --- |
| Brown et al. (2007) | Single question on perceived QoL  ‘How Satisfied with Way Spending Life These Days?’ | Not reported | Not reported |
| Conroy et al. (2002) | “Quality of Life Changes” Scale asks each person to rate his/her quality of life “A Year Ago” and “Now.” Ratings are given on 5 point, Likert scales, and covers 14 domains including health, friendships, safety, and comfort. | Interrater reliability was found to be .76 | Not reported |
| Glendinning et al. (2008) | Perceived Quality of life – using a seven-point scale ranging from ‘So good, it could not be better’ to ‘So bad, it could not be worse’.  In addition Psychological well-being was measured using General Health Questionnaire. | GHQ - Cronbach’s Alpha was 0.92 | Not reported |
| Woolham (2013) | Psychological well-being was measured using General Health Questionnaire. | Not reported | Not reported |

### Table A9.2: Measures of client satisfaction in each study

| Study | Outcome measure used | Reliability | Validity |
| --- | --- | --- | --- |
| Beatty (1998) | Personal Assistance Satisfaction Index - The items cover a range of issues regarding satisfaction with the delivery of personal assistance services, including cost of services, control over assistants' schedule, availability of assistants, safety, and consumer-assistant interactions. | Internal consistency high, with a Cronbach's alpha of .88 | Not reported |
| Benjamin (2000) | Client satisfaction measures were adapted from those previously developed on medical outcomes and on home care. Variables included: Technical quality, service impact, general satisfaction and interpersonal manner. | Not reported | Not reported |
| Brown et al. (2007) | Satisfaction with paid care received was measured based on 1. the way paid caregiver helped with personal care, household activities and routine health care, 2. time of day paid worker helped, 3. level of difficulty in changing caregiver schedule and 4. satisfaction with overall care (transportation & use of care-related equipment). | Not reported | Not reported |
| Caldwell (2007) | Service satisfaction. Service satisfaction consisted  of five items. Examples of these items are: ‘‘To what degree do you get the kind of services you want?’’ and ‘‘In an overall sense, how satisfied are you with the services you receive?’’ A 5-point scale was used for each item, from 1 (not at all) to 5 (very much). | Alpha reliability of the scale for total sample at Time 3 was .96 | Not reported |
| Glendinning et al. (2008) | Satisfaction and quality of services. Measures of satisfaction and quality of care were based on quality indicators derived from the extensions to national User Experience Surveys for older home care service users and younger adults. | Cronbach’s Alpha of 0.80 | Not reported |

### Table A9.3: Measures of physical functioning in each study

| Study | Outcome measure used | Reliability | Validity |
| --- | --- | --- | --- |
| Benjamin (2000) | While functional status (Katz and Akpom 1976; Lawton 1971) was recorded in telephone interview, this was only reported with regards to unmet needs (see section 4.4.6). | NA | NA |
| Brown et al. (2007) | Within the overall ‘Health and Functioning’ category physical functioning was measured by asking respondents if they were ‘Not independent in last week: 1) getting in or out of bed, 2) bathing and 3) using toilet/diapers. These ADL were used a coefficients in the effectiveness analysis. | NA | NA |
| Glendinning et al. (2008) | Difficulties with Activities of Daily Living Scale (ADL) were collected at baseline to act as a coefficient within a multiple regression. Therefore individual ADL data are not presented. | NA | NA |
| Woolham (2013) | Activities of Daily Living Scale (ADL) - a simple measure by which the ability of those taking part to carry out everyday activities of daily living could be assessed | Not reported | Not reported |

### Table A9.4: Measures of costs data in each study

| Study | Outcome measure used | Reliability | Validity |
| --- | --- | --- | --- |
| Brown et al. (2007)  Dale & Brown (2005) | 1. Monthly costs presented for intervention and control groups.  2. Costs presented mean treatment and control with associated Treatment-Control Differences. | Not reported | Not reported |
| Glendinning et al. (2008) | The cost of social care packages are presented as weekly costs. Mean costs (per week) presented for intervention and control groups for 1. Health Care Costs and 2. Care management costs. Incremental cost effectiveness ratios for ASCOT and GHQ outcomes measures are also presented (using pre/post design). | GHQ - Cronbach’s Alpha was 0.92 | Not reported |
| Woolham (2013) | Mean package costs (per week) by care group for intervention and control groups. Costs represent costs of care management for the control groups and of staff time plus advocacy and support service time for intervention group. Infrastructure costs excluded for both groups. Scatterplots are used to examine intervention and control differences based. | Not reported | Not reported |

### Table A9.5: Measures of adverse outcomes in each study

| Study | Outcome measure used | Reliability | | Validity |
| --- | --- | --- | --- | --- |
| Benjamin (2000) | Unmet service needs measured by 1. Number of ADL needs unmet due to not needs (0-6) having help and 2. IADL unmet Number of IADL needs unmet due to not needs (0-5) having help.  ‘Physical and psychological risk’ whereby client yelled at, stolen from, pushed, shoved, neglected, ignored, injured while assisted or received unwanted sexual advances or carer under the influence | Not reported  Not reported | | Not reported  Not reported |
| Brown et al. (2007) | Comparisons drawn for intervention / control reporting 1. Unmet needs for help with daily living activity 2. Paid caregiver was rude or disrespectful and 3. Unmet needs for person assistance with household activities, personal care and transportation. 4. Care-related problems and events including ‘had a fall’, ‘Contractures Developed/Worsened’, ‘Bedsores Developed/Worsened’ and ‘had urinary tract infection’ | | Not reported | Not reported |
| Caldwell (2007) | A modified version of the Family Support Index (Heller & Factor, 1993; Heller et al., 1999) was used to measure unmet service needs. This index included a list of 28 common types of services used by individuals with disabilities and families. Families were asked whether they used each service. If families were not using a service, they were asked whether they needed it. Unmet needs for each service were totalled. | Not reported | | Not reported |
| Conroy (2002) | **Challenging behaviour** scale is complementary to the Adaptive Behaviour scale. It is composed of 14 items detailing various maladaptive behaviours on a 100-point scale, with higher scores indicating less challenging behaviour. | Not reported | | Not reported |
| Glendinning et al. (2008) | Psychological ill-health using GHQ-12. By scoring each item as 0 or 1, sums them, and then calculates the proportion of people with a total score of 4 or higher, which is conventionally interpreted as indicating that they are at risk of psychological ill-health. | Cronbach’s Alpha was 0.92 | | Not reported |

### Table A9.6: Measures of other health and social care outcomes in each study

| Study | Outcome measure used | Reliability | Validity |
| --- | --- | --- | --- |
| Benjamin (2000) | ‘Sense of security’ – How safe client feel with provider and how well they get along with provider. | Not reported | Not reported |
| Caldwell (2007) | Community participation of individuals with developmental disabilities Community participation was measured using the Community Integration Scale  (Heller & Factor, 1991), which measures frequency  of participation in eight common community activities | Alpha reliability at Time 3 was .66. | Not reported |
| Conroy (2002) | Choice making – ‘Decision control inventory’ – 10 point scale of 35 decision making ratings where 0 denotes a choice is made entirely by paid staff and 10 denotes a choice made entirely by the focus person (and/or unpaid trusted others)  Integration – ‘Harris poll of Americans with and without disabilities’ - measuring how often people visit with friends, go shopping, go to a place of worship, engage in recreation, and so on, in the presence of citizens without a disability.  The Individual Planning Process – includes a scale to measure the “Elements of the Planning Process”, designed to reflect the degree to which planning is carried out in a “person-centred” manner. | Interrater reliability of .86  Interrater reliability (.97)  Not reported | Not reported  Not reported  Not reported |
| Glendinning et al. (2008) | Self-perceived health - based on a (previously used) five point scale that asks respondents to rate their health in general according to five categories ranging from ‘Very good’ to ‘Very bad’.  Social care outcomes using Adult Social Care Outcomes Toolkit (ASCOT) is a preference weighted indicator that reflects need for help and outcome gain from services across seven domains ranging from basic areas of need such as personal care and food and nutrition to social participation and involvement and control over daily life. | Not reported  ASCOT – Cronbachs alpha was 0.74. | Not reported  Not reported |
