# Supplementary material for: Individualized funding interventions to improve health and social care outcomes for people with a disability: A mixed‐methods systematic review
Source: Campbell Syst Rev. 2019 Jul 19;15(1-2):e1008. doi: 10.4073/csr.2019.3 (PMC8356501; doi:10.4073/csr.2019.3)
Supplement: Supplementary file 10 — Supporting information [file CL2-15-e1008-s005.docx]

# Appendix 10: Complete list of qualitative themes, subthemes and levels of coding

|  | **MACRO** | | | **MESO** | | | **MICRO** | | | **Coded pieces of text** |
| --- | --- | --- | --- | --- | --- | --- | --- | --- | --- | --- |
|  | **LEVEL 1** | **LEVEL 2** | | **LEVEL 3** | **LEVEL 4** | | **LEVEL 5** | **LEVEL 6** | |  |
| 1 | **Contributing factors** |  | |  |  | |  |  | |  |
| 2 |  | 3rd parties | |  |  | |  |  | |  |
| 3 |  |  | |  |  | |  |  | |  |
| 4 |  |  | | Local Authority / Funders |  | |  |  | | **62** |
| 5 |  |  | | Organisational attributes |  | |  |  | | **5** |
| 6 |  |  | |  | Org. cultural practices | |  |  | | **4** |
| 7 |  |  | |  |  | | Pay-rise |  | | **1** |
| 8 |  |  | | State government |  | |  |  | | **5** |
| 9 |  |  | |  | Political power | |  |  | | **5** |
| 10 |  |  | |  |  | |  |  | |  |
| 11 |  | Access to funds | |  |  | |  |  | | **105** |
| 12 |  |  | | How money was used |  | |  |  | | **116** |
| 13 |  |  | |  | ADL | |  |  | | **108** |
| 14 |  |  | |  | Attend courses / classes / clubs | |  |  | | **31** |
| 15 |  |  | |  | Childcare | |  |  | | **6** |
| 16 |  |  | |  | Health and fitness | |  |  | | **29** |
| 17 |  |  | |  | Home improvement | |  |  | | **21** |
| 18 |  |  | |  | Hours of Trad Care | |  |  | | **18** |
| 19 |  |  | |  |  | | Day Centre |  | | **15** |
| 20 |  |  | |  | Household goods | |  |  | | **3** |
| 21 |  |  | |  | intimate care | |  |  | | **43** |
| 22 |  |  | |  | Medical Equipment | |  |  | | **6** |
| 23 |  |  | |  | Medicine / medical supplies | |  |  | | **12** |
| 24 |  |  | |  | Paid assistance | |  |  | | **155** |
| 25 |  |  | |  | Personal care | |  |  | | **32** |
| 26 |  |  | |  | Respite hours | |  |  | | **55** |
| 27 |  |  | |  | Technology / assistive tech | |  |  | | **56** |
| 28 |  |  | |  | Therapy | |  |  | | **23** |
| 29 |  |  | |  | Transport | |  |  | | **51** |
| 30 |  |  | | Spending criteria/ restriction |  | |  |  | | **102** |
| 31 |  |  | |  | Want vs. need | |  |  | | **9** |
| 32 |  | Conditions / arrangements | |  |  | |  |  | |  |
| 33 |  |  | | accountability |  | |  |  | | **31** |
| 34 |  |  | | Communication |  | |  |  | | **72** |
| 35 |  |  | |  | different forms of expression | |  |  | | **17** |
| 36 |  |  | |  | Fam. expect to be consulted | |  |  | | **13** |
| 37 |  |  | |  |  | | Ask questions |  | | **2** |
| 38 |  |  | |  | Word of mouth | |  |  | | **27** |
| 39 |  |  | |  |  | | School |  | | **3** |
| 40 |  |  | | Dedicating time |  | |  |  | | **21** |
| 41 |  |  | | Engagement |  | |  |  | | **6** |
| 42 |  |  | | Information |  | |  |  | | **177** |
| 43 |  |  | |  | guidance / advice | |  |  | | **113** |
| 44 |  |  | | Org. support |  | |  |  | | **26** |
| 45 |  |  | | Practical support |  | |  |  | | **30** |
| 46 |  |  | | Promotion of I.F. |  | |  |  | | **23** |
| 47 |  |  | | Training |  | |  |  | | **136** |
| 48 |  |  | |  | Rekindle ability to choose | |  |  | | **1** |
| 49 |  | HR | |  |  | |  |  | | **56** |
| 50 |  |  | | advocacy |  | |  |  | | **35** |
| 51 |  |  | | Carer |  | |  |  | | **28** |
| 52 |  |  | | Centralised staff |  | |  |  | | **16** |
| 53 |  |  | |  | in-house | |  |  | | **5** |
| 54 |  |  | | Frontline staff |  | |  |  | | **21** |
| 55 |  |  | | GP |  | |  |  | | **7** |
| 56 |  |  | | health and safety |  | |  |  | | **12** |
| 57 |  |  | |  | Home is workplace | |  |  | | **2** |
| 58 |  |  | | Independent provider / facilitator |  | |  |  | | **8** |
| 59 |  |  | | New recruits |  | |  |  | | **6** |
| 60 |  |  | |  | Job benefits | |  |  | | **17** |
| 61 |  |  | | No previous exp. |  | |  |  | | **10** |
| 62 |  |  | | one-to-one |  | |  |  | | **26** |
| 63 |  |  | | PA skills / role |  | |  |  | | **100** |
| 64 |  |  | |  | Healthcare | |  |  | | **55** |
| 65 |  |  | | Previous exp |  | |  |  | | **243** |
| 66 |  |  | | Supervision |  | |  |  | | **38** |
| 67 |  |  | | Supporter attribute |  | |  |  | | **6** |
| 68 |  | Interpersonal relationships | |  |  | |  |  | | **93** |
| 69 |  |  | | Consumer attributes |  | |  |  | | **10** |
| 70 |  |  | |  | articulate | |  |  | | **1** |
| 71 |  |  | |  | Modest | |  |  | | **4** |
| 72 |  |  | |  | Open to new ideas | |  |  | | **12** |
| 73 |  |  | |  | proactive | |  |  | | **18** |
| 74 |  |  | | Hands on |  | |  |  | | **11** |
| 75 |  |  | | sense of obligation |  | |  |  | | **17** |
| 76 |  |  | | Staff attitudes |  | |  |  | | **6** |
| 77 |  |  | | tension |  | |  |  | | **8** |
| 78 |  | Limitations | |  |  | |  |  | | **52** |
| 79 |  | Location / Setting | |  |  | |  |  | | **3** |
| 80 |  |  | | Informal setting |  | |  |  | | **33** |
| 81 |  |  | | Living alone |  | |  |  | | **3** |
| 82 |  |  | | Own home |  | |  |  | | **54** |
| 83 |  |  | | residential settings |  | |  |  | | **11** |
| 84 |  | The system | |  |  | |  |  | | **30** |
| 85 |  |  | | Challenge the system |  | |  |  | | **18** |
| 86 |  |  | | No change in practice |  | |  |  | | **44** |
| 87 |  |  | |  | based on existing system | |  |  | | **3** |
| 88 |  |  | | Rigorous systems |  | |  |  | | **4** |
| 89 |  |  | | Social benefits system |  | |  |  | | **2** |
| 90 |  |  | | two tier system |  | |  |  | | **3** |
| 91 |  | User attributes | |  |  | |  |  | |  |
| 92 |  |  | | Changing preferences |  | |  |  | | **14** |
| 93 |  |  | |  | Avoid segregation / group based | |  |  | | **9** |
| 94 |  |  | |  | Does not want trad. ser | |  |  | | **37** |
| 95 |  |  | |  | Needs change | |  |  | | **40** |
| 96 |  |  | | cultural, language & religious factors |  | |  |  | | **18** |
| 97 |  |  | | Loudest voice |  | |  |  | | **3** |
| 98 |  |  | | Need for proxy |  | |  |  | | **8** |
| 99 |  |  | | Older people |  | |  |  | | **12** |
| 100 |  |  | | Severity / type of disability |  | |  |  | | **84** |
| 101 |  |  | |  |  | |  |  | |  |
| 102 | **Implementation Challenges** |  | |  |  | |  |  | |  |
| 103 |  | Imp, Challenge - Staff / Org. Perspective | |  |  | |  |  | | **404** |
| 104 |  |  | | Fear |  | |  |  | | **94** |
| 105 |  |  | |  | Flood the system | |  |  | | **5** |
| 106 |  |  | |  | Impact on existing services | |  |  | | **40** |
| 107 |  |  | |  |  | | Expands workforce |  | | **1** |
| 108 |  |  | |  |  | | Large caseloads |  | | **2** |
| 109 |  |  | |  |  | | Privatisation of care |  | | **3** |
| 110 |  |  | |  |  | |  | Competing services | | **3** |
| 111 |  |  | |  |  | |  | Disjointed services / supports | | **13** |
| 112 |  |  | |  |  | |  | Economies of scale?? | | **4** |
| 113 |  |  | |  | Misuse | |  |  | | **23** |
| 114 |  |  | |  |  | | Fraud |  | | **10** |
| 115 |  |  | |  |  | | Reviewing Receipts not important |  | | **1** |
| 116 |  |  | |  |  | | self-destructive to misuse |  | | **2** |
| 117 |  |  | |  | Safeguarding | |  |  | | **49** |
| 118 |  |  | |  |  | | risk aversion |  | | **9** |
| 119 |  |  | |  |  | |  | bankrupt | | **3** |
| 120 |  |  | | Staff scepticism |  | |  |  | | **14** |
| 121 |  |  | |  | Prof. avoidance | |  |  | | **4** |
| 122 |  |  | |  | Pressure to promote | |  |  | | **5** |
| 123 |  |  | | Accommodating different levels of need |  | |  |  | | **1** |
| 124 |  |  | |  | Different backgrounds / life experience | |  |  | | **6** |
| 125 |  |  | |  | High support needs | |  |  | | **45** |
| 126 |  |  | |  | Little support required | |  |  | | **5** |
| 127 |  |  | |  | required ongoing support | |  |  | | **33** |
| 128 |  | Perceived Negative / Challenging Aspects | |  |  | |  |  | | **820** |
| 129 |  |  | | Cross-cutting challenges |  | |  |  | |  |
| 130 |  |  | |  | increased workload | |  |  | | **39** |
| 131 |  |  | |  | Inequitable distribution of funds | |  |  | | **25** |
| 132 |  |  | |  | intrusive | |  |  | | **6** |
| 133 |  |  | |  | Lack of trust | |  |  | | **38** |
| 134 |  |  | |  | Not inclusive | |  |  | | **39** |
| 135 |  |  | |  | Relinquish control | |  |  | | **28** |
| 136 |  |  | |  | Risk | |  |  | | **69** |
| 137 |  |  | |  | Stressful | |  |  | | **102** |
| 138 |  |  | |  | Too complex | |  |  | | **69** |
| 139 |  |  | | External factors |  | |  |  | |  |
| 140 |  |  | |  | 3rd party | |  |  | |  |
| 141 |  |  | |  |  | | Abuse |  | | **12** |
| 142 |  |  | |  |  | | Bad attitude / Hostile |  | | **41** |
| 143 |  |  | |  |  | |  | Avoidance | | **6** |
| 144 |  |  | |  |  | |  | Discouraged | | **10** |
| 145 |  |  | |  |  | | Overskilled |  | | **3** |
| 146 |  |  | |  |  | | Paternalistic |  | | **36** |
| 147 |  |  | |  |  | |  | Authoritarian | | **3** |
| 148 |  |  | |  |  | |  | Controlled by regime | | **5** |
| 149 |  |  | |  |  | |  | Patronising / demeaning | | **13** |
| 150 |  |  | |  |  | | Relationship balance |  | | **22** |
| 151 |  |  | |  |  | | Serve own interests |  | | **13** |
| 152 |  |  | |  |  | |  | Designed to benefit others | | **4** |
| 153 |  |  | |  |  | | Staff turnover / retention |  | | **79** |
| 154 |  |  | |  |  | |  | Finding competent staff | | **10** |
| 155 |  |  | |  |  | |  | Rurality | | **24** |
| 156 |  |  | |  |  | | Toll on carer |  | | **91** |
| 157 |  |  | |  |  | |  | assumption | | **5** |
| 158 |  |  | |  |  | | unresponsive |  | | **20** |
| 159 |  |  | |  |  | | Weak network of support |  | | **36** |
| 160 |  |  | |  | Change unsettling | |  |  | | **35** |
| 161 |  |  | |  | Conflated publicity | |  |  | | **3** |
| 162 |  |  | |  |  | | failure to promote |  | | **33** |
| 163 |  |  | |  |  | |  | Not publicised | | **19** |
| 164 |  |  | |  | Delay in process | |  |  | | **121** |
| 165 |  |  | |  | Financial issues | |  |  | | **93** |
| 166 |  |  | |  |  | | Disappointment with level of funding |  | | **47** |
| 167 |  |  | |  |  | | high costs |  | | **9** |
| 168 |  |  | |  |  | |  | Unit pricing | | **3** |
| 169 |  |  | |  |  | | Lack of work benefits |  | | **3** |
| 170 |  |  | |  |  | | Low pay / wage |  | | **52** |
| 171 |  |  | |  |  | |  | Working hours + vol | | **27** |
| 172 |  |  | |  |  | | No transitionary money |  | | **3** |
| 173 |  |  | |  |  | | Payroll & Tax |  | | **87** |
| 174 |  |  | |  |  | |  | Money to pay for infrastructure | | **2** |
| 175 |  |  | |  | increased bureaucracy | |  |  | | **70** |
| 176 |  |  | |  | Lack of clarity | |  |  | | **91** |
| 177 |  |  | |  |  | | Unclear roles |  | | **36** |
| 178 |  |  | |  | No time | |  |  | | **18** |
| 179 |  |  | |  |  | | Time consuming |  | | **40** |
| 180 |  |  | | Individual factors |  | |  |  | | **0** |
| 181 |  |  | |  | Fear of IF ending | |  |  | | **40** |
| 182 |  |  | |  |  | | Lose services |  | | **4** |
| 183 |  |  | |  | Lack of independence | |  |  | | **6** |
| 184 |  |  | |  | Negative emotions | |  |  | | **3** |
| 185 |  |  | |  |  | | Burnt out |  | | **5** |
| 186 |  |  | |  |  | | lack of motivation |  | | **11** |
| 187 |  |  | |  |  | | lonely and isolated |  | | **20** |
| 188 |  |  | |  |  | | not coping |  | | **5** |
| 189 |  |  | |  | No employment opportunities | |  |  | | **3** |
| 190 |  |  | |  | Not for everyone | |  |  | | **18** |
| 191 |  |  | |  |  | | DP not appropriate |  | | **4** |
| 192 |  |  | |  |  | | not for the faint hearted |  | | **2** |
| 193 |  |  | |  | personal issues | |  |  | | **2** |
| 194 |  |  | |  |  | | Behavioural |  | | **29** |
| 195 |  |  | |  |  | | Managing ill health |  | | **21** |
| 196 |  |  | |  |  | | Self-neglect |  | | **7** |
| 197 |  | Potential problem / Area for improvement | |  |  | |  |  | |  |
| 198 |  |  | | Disabling practices |  | |  |  | | **3** |
| 199 |  |  | |  | Disability Awareness (soc. oppression) | |  |  | | **11** |
| 200 |  |  | |  |  | | A wish, not a right |  | | **4** |
| 201 |  |  | |  |  | | Public perception |  | | **22** |
| 202 |  |  | |  | Facility-based (segregated) activities | |  |  | | **7** |
| 203 |  |  | |  | Override PwD / Funds gatekeeper | |  |  | | **42** |
| 204 |  |  | |  |  | | 3rd party pressure |  | | **18** |
| 205 |  |  | |  |  | | Suggestions ignored |  | | **2** |
| 206 |  |  | |  |  | | Only option / no alternatives |  | | **36** |
| 207 |  |  | |  |  | |  | Don't have control | | **4** |
| 208 |  |  | |  |  | |  | Hands tied | | **3** |
| 209 |  |  | |  |  | |  | More restricted now | | **5** |
| 210 |  |  | |  |  | |  | no choice | | **2** |
| 211 |  |  | |  | Perceived inability (3rd party) | |  |  | | **42** |
| 212 |  |  | | Financial issues |  | |  |  | |  |
| 213 |  |  | |  | Budget cuts | |  |  | | **21** |
| 214 |  |  | |  |  | | claw back funds |  | | **4** |
| 215 |  |  | |  |  | | Freeze expansion |  | | **2** |
| 216 |  |  | |  |  | | IF end when Goal achieved |  | | **5** |
| 217 |  |  | |  | Charges for PwD | |  |  | | **18** |
| 218 |  |  | |  | Keeping funding source separate | |  |  | | **27** |
| 219 |  |  | |  |  | | Conflating funding sources |  | | **6** |
| 220 |  |  | |  | Not cost saving | |  |  | | **8** |
| 221 |  |  | |  | Unsustainable | |  |  | | **33** |
| 222 |  |  | |  |  | | Hidden costs |  | | **30** |
| 223 |  |  | |  |  | |  | out of pocket | | **12** |
| 224 |  |  | | Human Resources |  | |  |  | | **1** |
| 225 |  |  | |  | Available support | |  |  | | **224** |
| 226 |  |  | |  |  | | Over/under-estimate needs |  | | **15** |
| 227 |  |  | |  |  | |  | less hours than needed | | **17** |
| 228 |  |  | |  |  | |  | Subjective | | **2** |
| 229 |  |  | |  |  | | Need additional help |  | | **11** |
| 230 |  |  | |  |  | | conflict of interest |  | | **3** |
| 231 |  |  | |  |  | | Less contact with services |  | | **5** |
| 232 |  |  | |  |  | | rely on informal supports |  | | **24** |
| 233 |  |  | |  |  | | over-reliant on 1 person |  | | **4** |
| 234 |  |  | |  |  | | Finding flatmate |  | | **2** |
| 235 |  |  | |  | Behaviour Change | |  |  | | **19** |
| 236 |  |  | |  |  | | accepting help |  | | **6** |
| 237 |  |  | |  |  | | Avoid preconceived ideas |  | | **2** |
| 238 |  |  | |  |  | | Learned passivity |  | | **35** |
| 239 |  |  | |  |  | | Struggle to let go |  | | **4** |
| 240 |  |  | |  |  | | unrealistic expectations |  | | **27** |
| 241 |  |  | |  | minimum level of training | |  |  | | **15** |
| 242 |  |  | |  |  | | need skills and knowledge |  | | **72** |
| 243 |  |  | |  |  | | No formal training |  | | **27** |
| 244 |  |  | |  |  | |  | Disciplinary role as employer | | **2** |
| 245 |  |  | |  | Respecting boundaries | |  |  | | **19** |
| 246 |  |  | |  |  | | conflict |  | | **36** |
| 247 |  |  | | Negative emotions / perceptions |  | |  |  | |  |
| 248 |  |  | |  | Big / more responsibility | |  |  | | **29** |
| 249 |  |  | |  |  | | daunting |  | | **22** |
| 250 |  |  | |  |  | |  | apprehensive | | **2** |
| 251 |  |  | |  |  | | struggle |  | | **6** |
| 252 |  |  | |  | Burden | |  |  | | **12** |
| 253 |  |  | |  |  | | Ask too much |  | | **6** |
| 254 |  |  | |  |  | | Guilt |  | | **8** |
| 255 |  |  | |  | Suspicious | |  |  | | **7** |
| 256 |  |  | |  |  | | destroy informal supports / familism |  | | **4** |
| 257 |  |  | |  |  | | Paying lip service to I.F. |  | | **2** |
| 258 |  |  | |  |  | | penalised for honesty |  | | **1** |
| 259 |  |  | |  |  | | Penalised for working |  | | **7** |
| 260 |  |  | |  |  | | Set up to fail |  | | **4** |
| 261 |  |  | |  | vulnerability | |  |  | | **21** |
| 262 |  |  | |  |  | | reluctant to ‘rock the boat’ |  | | **5** |
| 263 |  |  | |  |  | | What happens when parents die? |  | | **7** |
| 264 |  |  | | Operational |  | |  |  | |  |
| 265 |  |  | |  | Cumbersome systems | |  |  | | **13** |
| 266 |  |  | |  |  | | duplication |  | | **23** |
| 267 |  |  | |  |  | |  | repeatedly explain | | **2** |
| 268 |  |  | |  |  | | Fire fighting |  | | **9** |
| 269 |  |  | |  |  | |  | focus on crisis / acute / deficit | | **4** |
| 270 |  |  | |  |  | | Inappropriate / wrong focus |  | | **28** |
| 271 |  |  | |  |  | |  | fit for purpose | | **3** |
| 272 |  |  | |  |  | |  | Medical model | | **8** |
| 273 |  |  | |  |  | |  | Not a priority | | **8** |
| 274 |  |  | |  |  | |  | Targets / costs vs. quality | | **10** |
| 275 |  |  | |  |  | | inflexible / too rigid |  | | **56** |
| 276 |  |  | |  | Inconsistent approaches | |  |  | | **49** |
| 277 |  |  | |  |  | | Unclear procedure / legislation |  | | **2** |
| 278 |  |  | |  | Info needs | |  |  | | **305** |
| 279 |  |  | |  |  | | Inaccessible |  | | **12** |
| 280 |  |  | |  |  | | Inaccurate information |  | | **14** |
| 281 |  |  | |  |  | |  | Too much | | **3** |
| 282 |  |  | |  |  | |  | outdated | | **2** |
| 283 |  |  | |  |  | | Mixed messages |  | | **30** |
| 284 |  |  | |  |  | | unaware |  | | **5** |
| 285 |  |  | |  | Legal challenges | |  |  | | **14** |
| 286 |  |  | |  |  | | liability |  | | **2** |
| 287 |  |  | |  | Transitionary period | |  |  | | **8** |
| 288 |  |  | |  |  | |  |  | |  |
| 289 | **Implementation facilitators** |  | |  |  | |  |  | |  |
| 290 |  | Imp. Facilitator - Staff / Org. perspective | |  |  | |  |  | | **292** |
| 291 |  | Mechanisms of success | |  |  | |  |  | |  |
| 292 |  |  | | Active community member |  | |  |  | | **24** |
| 293 |  |  | |  | existing community resources / mainstream | |  |  | | **37** |
| 294 |  |  | | Buy-in |  | |  |  | | **15** |
| 295 |  |  | |  | commitment | |  |  | | **24** |
| 296 |  |  | | control and choice |  | |  |  | | **104** |
| 297 |  |  | |  | control of family | |  |  | | **3** |
| 298 |  |  | |  | control of their disability / life | |  |  | | **45** |
| 299 |  |  | | early intervention |  | |  |  | | **10** |
| 300 |  |  | |  | preventative | |  |  | | **3** |
| 301 |  |  | | Employment considerations |  | |  |  | |  |
| 302 |  |  | |  | Good employer / employment practice | |  |  | | **32** |
| 303 |  |  | |  |  | | higher / appropriate rate of pay |  | | **18** |
| 304 |  |  | |  |  | | Treat well |  | | **4** |
| 305 |  |  | |  | Hire family | |  |  | | **125** |
| 306 |  |  | |  |  | | Siblings involved |  | | **4** |
| 307 |  |  | |  |  | | Knows what needs to be done |  | | **43** |
| 308 |  |  | |  |  | | Would do anything |  | | **7** |
| 309 |  |  | |  | I'm the Boss / Power shift | |  |  | | **29** |
| 310 |  |  | |  |  | | Influence purchasing power |  | | **16** |
| 311 |  |  | |  |  | | Power to sack |  | | **23** |
| 312 |  |  | |  |  | | Set terms of employment |  | | **37** |
| 313 |  |  | |  | Known to PwD/Family - Familiarity | |  |  | | **22** |
| 314 |  |  | |  |  | | Hire non-family |  | | **4** |
| 315 |  |  | |  |  | | Hire friend |  | | **42** |
| 316 |  |  | | flexibility |  | |  |  | | **177** |
| 317 |  |  | | Future planning/ Purpose |  | |  |  | | **74** |
| 318 |  |  | |  | Aspirations | |  |  | | **25** |
| 319 |  |  | |  |  | | Exceeded expectations |  | | **4** |
| 320 |  |  | |  | Have a plan | |  |  | | **2** |
| 321 |  |  | |  | Have long term view / vision | |  |  | | **26** |
| 322 |  |  | |  |  | | Aim High |  | | **3** |
| 323 |  |  | |  |  | | Short term / achievable goals |  | | **26** |
| 324 |  |  | |  | Recovery plan | |  |  | | **3** |
| 325 |  |  | | Hands-off approach |  | |  |  | | **41** |
| 326 |  |  | | Holistic approach / comprehensive |  | |  |  | | **18** |
| 327 |  |  | | Inclusive |  | |  |  | | **27** |
| 328 |  |  | | increased knowledge |  | |  |  | | **25** |
| 329 |  |  | |  | knowing how much money | |  |  | | **16** |
| 330 |  |  | |  | Understanding I.F. | |  |  | | **79** |
| 331 |  |  | |  |  | | support hours vs. services |  | | **1** |
| 332 |  |  | | Integration of services |  | |  |  | | **6** |
| 333 |  |  | |  | Integrating information | |  |  | | **2** |
| 334 |  |  | | Needs led |  | |  |  | | **135** |
| 335 |  |  | | Outcome focussed |  | |  |  | | **25** |
| 336 |  |  | |  | Health, social care outcome | |  |  | | **30** |
| 337 |  |  | |  | Mental health or emotional wellbeing | |  |  | | **58** |
| 338 |  |  | |  | QoL | |  |  | | **28** |
| 339 |  |  | | Positive risk taking |  | |  |  | | **7** |
| 340 |  |  | | Quick and Easy / Convenient |  | |  |  | | **26** |
| 341 |  |  | |  | simplify / user-friendly | |  |  | | **4** |
| 342 |  |  | | Range of services |  | |  |  | | **17** |
| 343 |  |  | |  | quality of services | |  |  | | **88** |
| 344 |  |  | |  | tailored | |  |  | | **15** |
| 345 |  |  | |  |  | | Supporting differently |  | | **4** |
| 346 |  |  | |  | Variety is spice of life | |  |  | | **5** |
| 347 |  |  | | Relationships |  | |  |  | |  |
| 348 |  |  | |  | (Financial) recognition for vol work | |  |  | | **41** |
| 349 |  |  | |  | active listening skills | |  |  | | **4** |
| 350 |  |  | |  |  | | felt heard |  | | **7** |
| 351 |  |  | |  | Better understanding | |  |  | | **46** |
| 352 |  |  | |  | collaborative relationships | |  |  | | **52** |
| 353 |  |  | |  |  | | Shared understanding |  | | **32** |
| 354 |  |  | |  |  | | Shared learning |  | | **20** |
| 355 |  |  | |  | Deeper engagement | |  |  | | **7** |
| 356 |  |  | |  | Dignity / Respect | |  |  | | **47** |
| 357 |  |  | |  | Friendships from trad. services | |  |  | | **5** |
| 358 |  |  | |  | Manage expectations / ppl management | |  |  | | **28** |
| 359 |  |  | |  | Meaningful activity | |  |  | | **3** |
| 360 |  |  | |  |  | | encourage active role |  | | **1** |
| 361 |  |  | |  | Moral support | |  |  | | **43** |
| 362 |  |  | |  | Network of support | |  |  | | **306** |
| 363 |  |  | |  | PA attribute | |  |  | | **95** |
| 364 |  |  | |  |  | | Good disposition |  | | **8** |
| 365 |  |  | |  |  | | Live close by |  | | **14** |
| 366 |  |  | |  |  | | Proactive staff |  | | **2** |
| 367 |  |  | |  |  | | responsive |  | | **24** |
| 368 |  |  | |  |  | | shared interests / life-stage |  | | **26** |
| 369 |  |  | |  |  | |  | Age appropriate | | **5** |
| 370 |  |  | |  | satisfaction with staff | |  |  | | **19** |
| 371 |  |  | |  | Shift focus to positive | |  |  | | **6** |
| 372 |  |  | |  | Strong leadership | |  |  | | **3** |
| 373 |  |  | |  | trust | |  |  | | **82** |
| 374 |  |  | |  | Use humour | |  |  | | **4** |
| 375 |  |  | | Smooth transition |  | |  |  | | **4** |
| 376 |  |  | | tangible examples |  | |  |  | | **8** |
| 377 |  |  | | Thinking innovatively / creatively |  | |  |  | | **80** |
| 378 |  |  | | Transparency |  | |  |  | | **12** |
| 379 |  | Perceived benefit | |  |  | |  |  | | **662** |
| 380 |  |  | | <dependent on supports |  | |  |  | | **57** |
| 381 |  |  | | Avoid institutionalisation |  | |  |  | | **18** |
| 382 |  |  | | Back to / remain in work |  | |  |  | | **47** |
| 383 |  |  | | Community integration |  | |  |  | | **151** |
| 384 |  |  | | Complement existing vol. supports |  | |  |  | | **14** |
| 385 |  |  | | Continuity of Care / Service / Life |  | |  |  | | **121** |
| 386 |  |  | |  | Reliability | |  |  | | **19** |
| 387 |  |  | | Contribute to family life |  | |  |  | | **24** |
| 388 |  |  | | Enhance skills |  | |  |  | | **50** |
| 389 |  |  | |  | Continue self-improvement | |  |  | | **10** |
| 390 |  |  | |  | Life skills training | |  |  | | **30** |
| 391 |  |  | | enhanced relationship |  | |  |  | | **91** |
| 392 |  |  | | Formalise alternative supports |  | |  |  | | **9** |
| 393 |  |  | | Freedom |  | |  |  | |  |
| 394 |  |  | |  | Freedom (to choose) / individualisation | |  |  | | **84** |
| 395 |  |  | |  |  | | how you're supported |  | | **107** |
| 396 |  |  | |  |  | | When you're supported |  | | **87** |
| 397 |  |  | |  |  | | Where you're supported |  | | **36** |
| 398 |  |  | |  |  | | Who supports you |  | | **149** |
| 399 |  |  | |  |  | |  | Find the best fit | | **9** |
| 400 |  |  | |  |  | |  | request specific people | | **16** |
| 401 |  |  | |  | Personal freedom / I have my life back | |  |  | | **14** |
| 402 |  |  | |  |  | | Autonomy |  | | **10** |
| 403 |  |  | |  |  | | Freedom to make mistakes |  | | **4** |
| 404 |  |  | |  |  | | Self-determined |  | | **82** |
| 405 |  |  | |  |  | | Self-directed |  | | **98** |
| 406 |  |  | |  |  | | self-reliance |  | | **23** |
| 407 |  |  | |  |  | | Sense of empowerment |  | | **46** |
| 408 |  |  | |  |  | | Space and freedom |  | | **8** |
| 409 |  |  | | greater appreciation for money |  | |  |  | | **6** |
| 410 |  |  | | greater efficiency |  | |  |  | | **3** |
| 411 |  |  | | Improve family life |  | |  |  | | **128** |
| 412 |  |  | | improved self-image /self-belief / self-esteem |  | |  |  | | **53** |
| 413 |  |  | |  | Adulthood recognition | |  |  | | **3** |
| 414 |  |  | |  | build confidence | |  |  | | **5** |
| 415 |  |  | |  |  | | confidence |  | | **82** |
| 416 |  |  | |  | hope / positive outlook | |  |  | | **48** |
| 417 |  |  | |  |  | | enhanced self-awareness |  | | **8** |
| 418 |  |  | |  |  | | improved mood |  | | **4** |
| 419 |  |  | |  |  | | Less stress / anxiety |  | | **37** |
| 420 |  |  | |  |  | | Resilient |  | | **4** |
| 421 |  |  | |  |  | |  | Self-managing behaviour | | **21** |
| 422 |  |  | |  |  | | Self-worth |  | | **12** |
| 423 |  |  | |  | Increased vitality | |  |  | | **2** |
| 424 |  |  | |  | motivated | |  |  | | **18** |
| 425 |  |  | |  | positive emotional experiences | |  |  | | **13** |
| 426 |  |  | |  |  | | Benefits outweigh negatives |  | | **6** |
| 427 |  |  | |  |  | | Peace of mind |  | | **6** |
| 428 |  |  | |  |  | |  | felt cared for | | **2** |
| 429 |  |  | |  |  | |  | Safe and secure | | **14** |
| 430 |  |  | |  |  | |  | Fears alleviated | | **24** |
| 431 |  |  | |  |  | | Trickle-down effect (happiness) |  | | **3** |
| 432 |  |  | |  |  | |  | unexpected | | **8** |
| 433 |  |  | |  |  | |  | I'm happy if they're happy | | **6** |
| 434 |  |  | |  | Richer life | |  |  | | **16** |
| 435 |  |  | |  | Successful | |  |  | | **7** |
| 436 |  |  | | In-tune with needs |  | |  |  | | **14** |
| 437 |  |  | | independence |  | |  |  | | **85** |
| 438 |  |  | |  | independent as possible | |  |  | | **33** |
| 439 |  |  | | More bang for buck / Can do more |  | |  |  | |  |
| 440 |  |  | |  | Better / new opportunities | |  |  | | **58** |
| 441 |  |  | |  |  | | Civic participation / volunteering |  | | **32** |
| 442 |  |  | |  |  | | Get outdoors |  | | **22** |
| 443 |  |  | |  |  | | Recreational Opps |  | | **90** |
| 444 |  |  | |  |  | | Social opps |  | | **142** |
| 445 |  |  | |  | choosing cheaper option / value for money | |  |  | | **40** |
| 446 |  |  | | New friendships |  | |  |  | | **30** |
| 447 |  |  | | Not a burden |  | |  |  | | **29** |
| 448 |  |  | | Org. / Gov't cost saving |  | |  |  | | **16** |
| 449 |  |  | | privacy |  | |  |  | | **13** |
| 450 |  |  | |  | own bedroom | |  |  | | **2** |
| 451 |  |  | | Reduced cost overheads |  | |  |  | | **5** |
| 452 |  |  | | Reduced medication / hospitalisation |  | |  |  | | **13** |
| 453 |  |  | | To stay at home |  | |  |  | | **29** |
| 454 |  |  | |  | in-home support | |  |  | | **27** |
| 455 |  |  | |  |  | |  |  | |  |
| 456 | **Process** |  | |  |  | |  |  | | **25** |
| 457 |  | Admin / Management | |  |  | |  |  | | **235** |
| 458 |  |  | | Shared management |  | |  |  | | **10** |
| 459 |  |  | | Separate funding streams |  | |  |  | | **3** |
| 460 |  |  | | Governance |  | |  |  | | **17** |
| 461 |  |  | |  | Developing SOPs | |  |  | | **3** |
| 462 |  |  | |  | Develop policy | |  |  | | **2** |
| 463 |  |  | |  |  | | Adult protection policy |  | | **5** |
| 464 |  |  | | Annual vs monthly budget |  | |  |  | | **3** |
| 465 |  |  | | Forms / Paperwork |  | |  |  | | **198** |
| 466 |  | Logistics | |  |  | |  |  | |  |
| 467 |  |  | | Audit |  | |  |  | | **4** |
| 468 |  |  | | Banking |  | |  |  | | **2** |
| 469 |  |  | |  | Additional bank account(s) | |  |  | | **22** |
| 470 |  |  | | Basic system of organising |  | |  |  | | **6** |
| 471 |  |  | | Centralised services |  | |  |  | | **10** |
| 472 |  |  | | client data management |  | |  |  | | **12** |
| 473 |  |  | | Monitoring |  | |  |  | | **136** |
| 474 |  |  | |  | risk panel | |  |  | | **4** |
| 475 |  |  | |  | Complaints procedure | |  |  | | **9** |
| 476 |  |  | |  | Review | |  |  | | **64** |
| 477 |  |  | |  |  | | lack of / absent |  | | **8** |
| 478 |  |  | | PA / staff recruitment |  | |  |  | | **174** |
| 479 |  |  | |  | Vetting supports | |  |  | | **20** |
| 480 |  |  | |  | Switching agency | |  |  | | **3** |
| 481 |  |  | |  | Rostering | |  |  | | **9** |
| 482 |  |  | |  | Roles clearly specified | |  |  | | **14** |
| 483 |  |  | |  | Poach agency staff | |  |  | | **5** |
| 484 |  |  | |  | Place adverts | |  |  | | **18** |
| 485 |  |  | |  | Multiple assistants/ providers | |  |  | | **28** |
| 486 |  |  | |  | Dismissing PA | |  |  | | **25** |
| 487 |  |  | |  |  | | fire staff |  | | **4** |
| 488 |  |  | |  | Employ directly | |  |  | | **78** |
| 489 |  |  | |  |  | | Setting wage |  | | **2** |
| 490 |  |  | |  |  | | employment law |  | | **18** |
| 491 |  |  | |  |  | | Employment contract |  | | **10** |
| 492 |  |  | | streamline |  | |  |  | | **3** |
| 493 |  |  | |  | fast track process | |  |  | | **2** |
| 494 |  |  | |  | Standardisation | |  |  | | **7** |
| 495 |  | Setup | |  |  | |  |  | |  |
| 496 |  |  | | Uptake |  | |  |  | | **38** |
| 497 |  |  | |  | Drop-out | |  |  | | **10** |
| 498 |  |  | | Network building |  | |  |  | | **49** |
| 499 |  |  | |  | room mate | |  |  | | **6** |
| 500 |  |  | |  | Power of attorney / trustee | |  |  | | **9** |
| 501 |  |  | |  | PA's children | |  |  | | **5** |
| 502 |  |  | | Allocation calculation |  | |  |  | | **51** |
| 503 |  |  | |  | RAS | |  |  | | **4** |
| 504 |  |  | | Application/ enrolment |  | |  |  | | **19** |
| 505 |  |  | | Assessment |  | |  |  | | **127** |
| 506 |  |  | |  | Capacity | |  |  | | **25** |
| 507 |  |  | |  | estimating hours needed | |  |  | | **2** |
| 508 |  |  | |  | self-assessment | |  |  | | **15** |
| 509 |  |  | |  | Community care assessment | |  |  | | **2** |
| 510 |  |  | | determine eligibility |  | |  |  | | **22** |
| 511 |  |  | | How money can be used |  | |  |  | | **130** |
| 512 |  |  | |  | Pooling resources | |  |  | | **8** |
| 513 |  |  | |  | Contingency funds / plan | |  |  | | **18** |
| 514 |  |  | |  | extra transition costs | |  |  | | **3** |
| 515 |  |  | |  | Use up allocation | |  |  | | **16** |
| 516 |  |  | |  | Negotiations | |  |  | | **10** |
| 517 |  |  | |  | Sign-off plans / budget / spend | |  |  | | **41** |
| 518 |  |  | | Initial set up |  | |  |  | | **46** |
| 519 |  |  | |  | set up as a company / business | |  |  | | **4** |
| 520 |  |  | | Journey of discovery |  | |  |  | | **7** |
| 521 |  |  | |  | Decision Making | |  |  | | **32** |
| 522 |  |  | |  | prioritise requests | |  |  | | **2** |
| 523 |  |  | |  | learn to dream | |  |  | | **4** |
| 524 |  |  | |  | identify / organise resources | |  |  | | **48** |
| 525 |  |  | |  | Identify goals | |  |  | | **33** |
| 526 |  |  | |  | Choosing I.F. | |  |  | | **138** |
| 527 |  |  | | Letter of agreement |  | |  |  | | **3** |
| 528 |  |  | |  | consent vs understand | |  |  | | **4** |
| 529 |  |  | | PCP / Support plan |  | |  |  | | **135** |
| 530 |  |  | | Planning and outreach |  | |  |  | | **16** |
| 531 |  |  | | referral routes / mechanisms |  | |  |  | | **5** |
| 532 |  |  | | Tendering process |  | |  |  | | **5** |
| 533 |  | Types of supports | |  |  | |  |  | |  |
| 534 |  |  | | Wide range of people |  | |  |  | | **3** |
| 535 |  |  | | Peer support |  | |  |  | | **55** |
| 536 |  |  | | Offer continuum of SDS |  | |  |  | | **2** |
| 537 |  |  | | disability led advisory councils |  | |  |  | | **25** |
| 538 |  |  | | Local Support Orgs |  | |  |  | | **145** |
| 539 |  |  | |  | User-led orgs. | |  |  | | **16** |
| 540 |  |  | | Emergency support |  | |  |  | | **71** |
| 541 |  |  | | Direct Payment |  | |  |  | | **4** |
| 542 |  |  | | Brokerage / managed model |  | |  |  | | **11** |
| 543 |  |  | | Agency involvement |  | |  |  | | **236** |
| 544 |  |  | |  | Professionals / Practitioners | |  |  | | **234** |
| 545 |  |  | |  |  | | Carer lead officer |  | | **4** |
| 546 |  |  | |  |  | | Case / care manager / coordinator |  | | **38** |
| 547 |  |  | |  | I.F. coordinators / Support Brokers | |  |  | | **256** |
| 548 |  |  | |  |  | | intermediary |  | | **25** |
| 549 |  |  | |  |  | | Coordinator attributes |  | | **14** |
|  |  |  | |  | **TOTAL CODED SECTIONS OF TEXT** | | | | | **17961** |
|  |  | | | | | | | | | |
|  | **Total Level 1 Themes** | | **Total Level 2 Themes** | **Total Level 3 Themes** | | **Total Level 4 Themes** | **Total Level 5 Themes** | | **Total Level 6 Themes** |  |
|  | **4** | | **19** | **142** | | **192** | **144** | | **43** | **544** |
